# Atom-economic generation of gold carbenes: gold-catalyzed formal [3+2] cycloaddition between ynamides and isoxazoles†
†Electronic supplementary information (ESI) available. CCDC 1009902. For ESI and crystallographic data in CIF or other electronic format see DOI: 10.1039/c4sc02596b



**DOI:** 10.1039/c4sc02596b

**Published:** 2014-11-12

**Authors:** Ai-Hua Zhou, Qiao He, Chao Shu, Yong-Fei Yu, Shuang Liu, Tian Zhao, Wei Zhang, Xin Lu, Long-Wu Ye

**Affiliations:** a State Key Laboratory for Physical Chemistry of Solid Surfaces , The Key Laboratory for Chemical Biology of Fujian Province and Department of Chemistry , College of Chemistry and Chemical Engineering , Xiamen University , Xiamen 361005 , Fujian , P. R. China . Email: longwuye@xmu.edu.cn ; Fax: +86-592-218-5833 ; Tel: +86-592-218-5833; b State Key Laboratory of Physical Chemistry of Solid Surfaces , Key Laboratory for Theoretical and Computational Chemistry of Fujian Province and Department of Chemistry , College of Chemistry and Chemical Engineering , Xiamen University , Xiamen , 361005 , Fujian , P. R. China . Email: xinlu@xmu.edu.cn ; Fax: +86-592-218-1600

## Abstract

The generation of gold carbenes *via* the gold-catalyzed intermolecular reaction of nucleophiles containing relatively labile N–O or N–N bonds with alkynes has received considerable attention during recent years. However, this protocol is not atom-economic as the reaction produces a stoichiometric amount of pyridine or quinoline waste, the cleaved part of the N–O or N–N bonds. In this article, we disclose an unprecedented gold-catalyzed formal [3+2] cycloaddition between ynamides and isoxazoles, allowing rapid and practical access to a wide range of synthetically-useful 2-aminopyrroles. Most importantly, mechanistic studies and theoretical calculations revealed that this reaction presumably proceeds *via* an α-imino gold carbene pathway, thus providing a strategically novel, atom-economic route to the generation of gold carbenes. Other significant features of this approach include the use of readily-available starting materials, high flexibility, simple procedure, mild reaction conditions, and in particular, no need to exclude moisture or air (“open flask”).

## Abstract 2

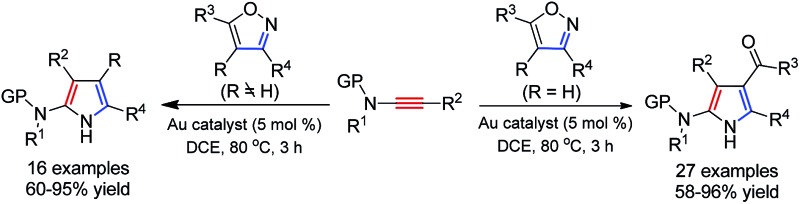
An unprecedented gold-catalyzed formal [3+2] cycloaddition between ynamides and isoxazoles has been developed, allowing rapid and practical access to a wide range of synthetically useful 2-aminopyrroles.

## Introduction

Catalytic transformations involving gold carbenes are arguably the most important aspect of homogeneous gold catalysis.^1^ Recently, the possibility of forming an α-oxo gold carbenoid species *via* gold-catalyzed intramolecular or intermolecular alkyne oxidation by a N–O bond oxidant (initially a sulfoxide), pioneered by Toste and Zhang,^2a,b^ represents a significant advance in gold carbene chemistry, and various efficient synthetic methods have been developed based on this strategy.^2^ Compared with intramolecular alkyne oxidation, the intermolecular approach offers much greater flexibility as no tethering of the oxidant is required, and therefore it is more synthetically useful.^3^ However, this intermolecular approach is obviously not atom-economic as the reaction produces a stoichiometric amount of pyridines or quinolines, the reduced form of the corresponding pyridine *N*-oxides or quinoline *N*-oxides, as waste (eqn (1)),^4^ which may even deactivate the gold catalyst *via* coordination.^5^
1
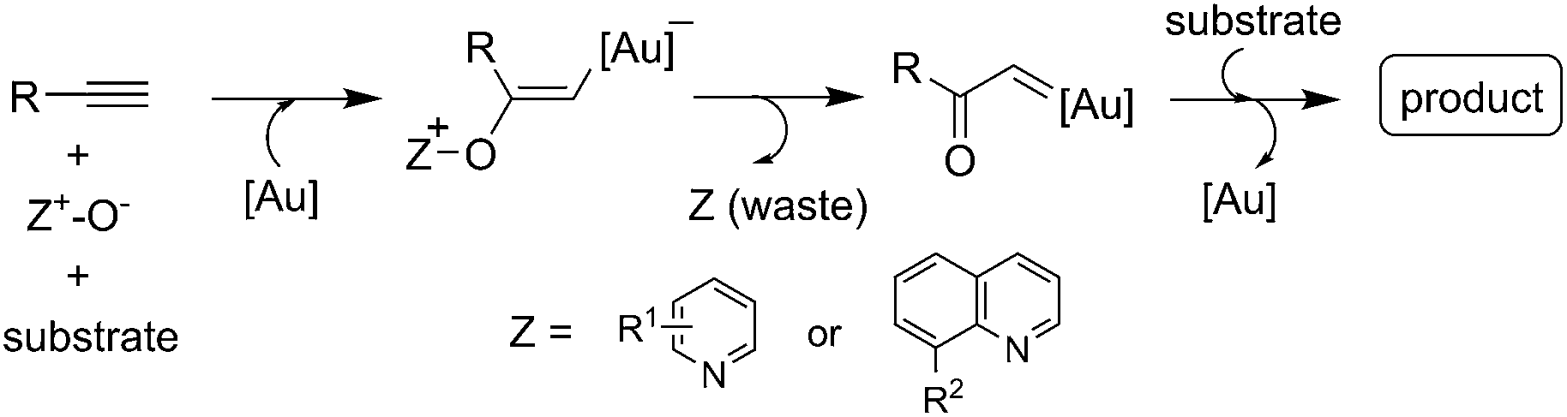



**Scheme 1:**
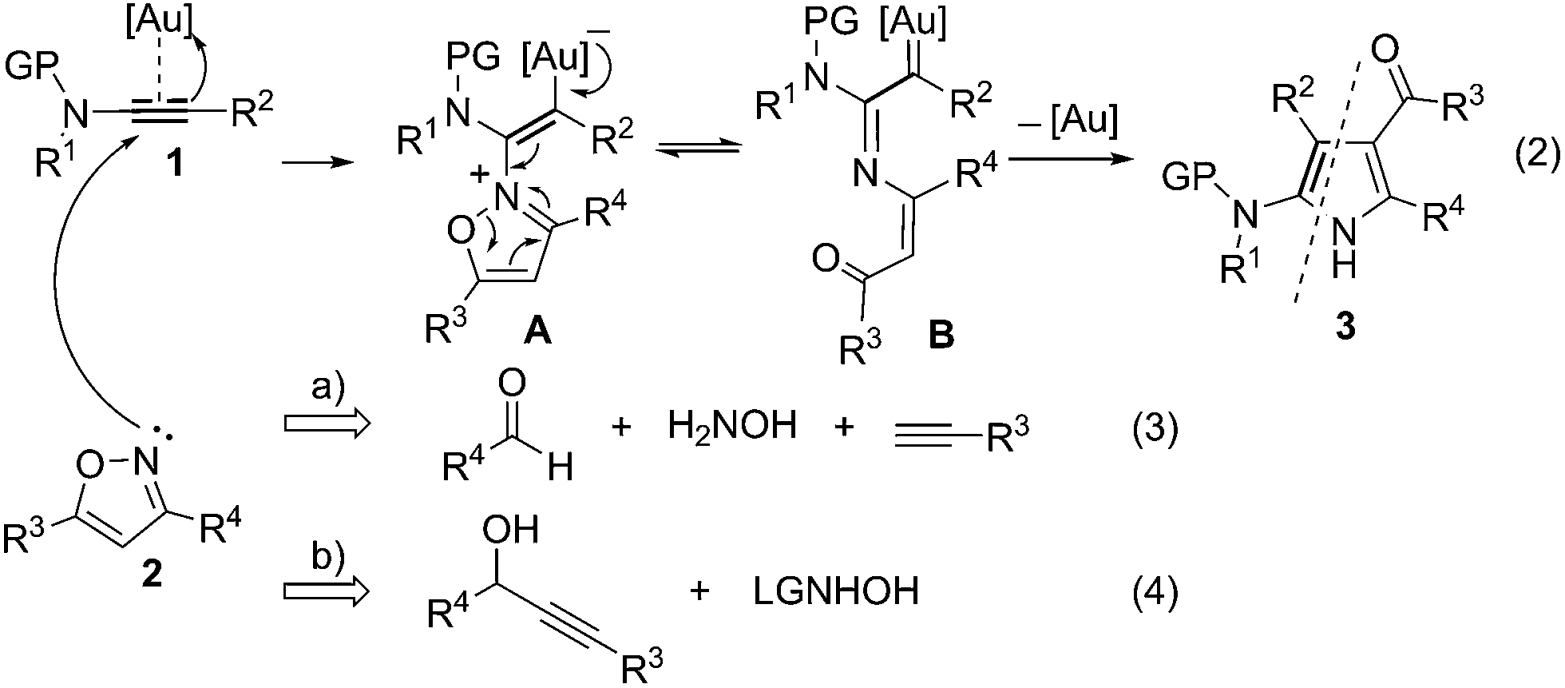
Synthetic design for the atom-economic generation of α-imino gold carbenes: formation of 2-aminopyrroles **3** through gold-catalyzed formal [3+2] cycloaddition between ynamides **1** and isoxazoles **2**.

**Fig. 1:**
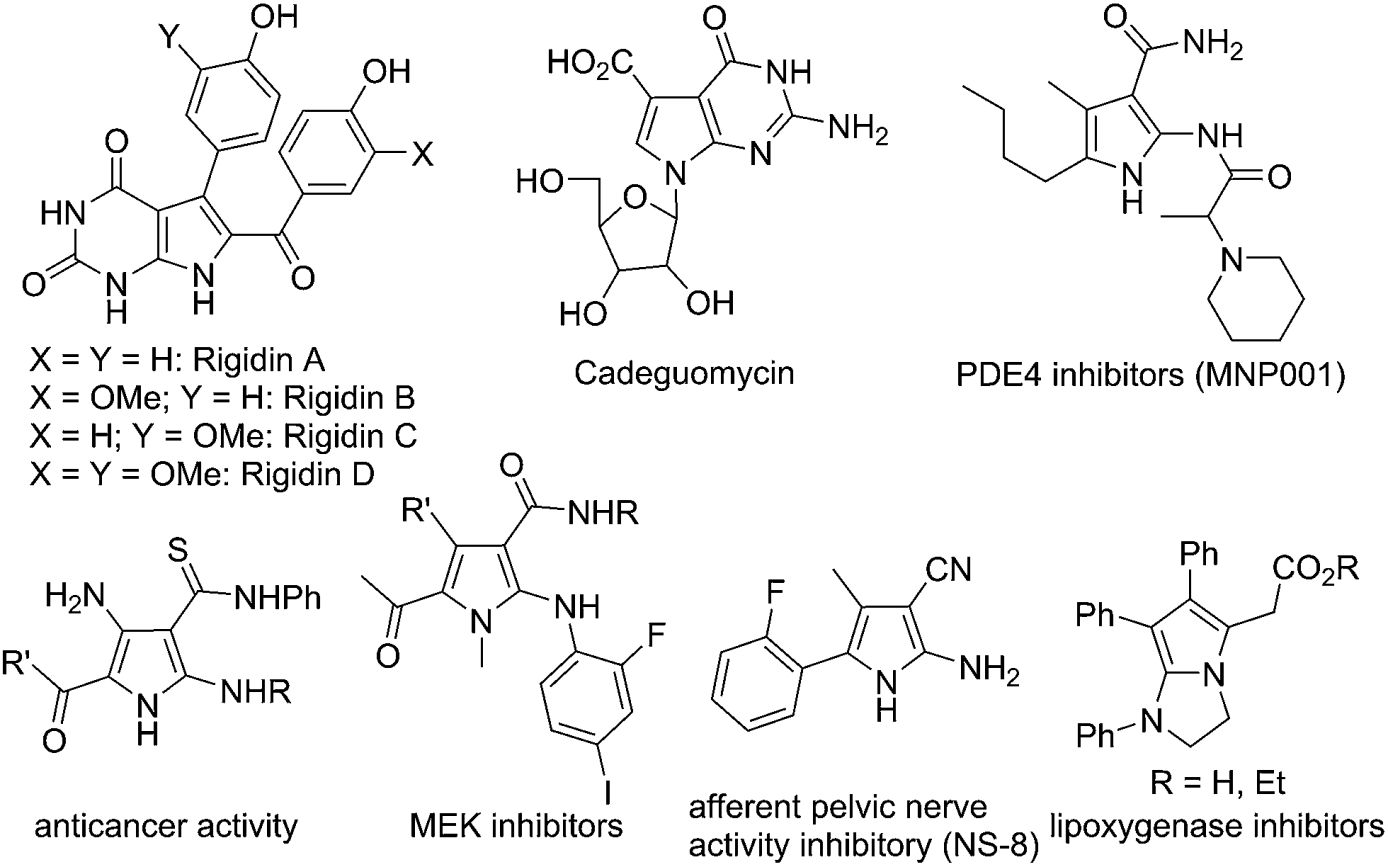
2-Aminopyrrole subunit in natural products and bioactive molecules.

Access to the related α-imino gold carbenes *via* gold-catalyzed nitrene transfer to alkynes, however, remains a highly challenging task. Here, it should be noted that: (1) the nitrene moiety is delivered *via* an outer sphere attack and no gold nitrene complex^6^ is involved in this case; this mode of nitrene transfer is distinctively different from many well-established nitrene transfer reactions;^7^ (2) this protocol would present alkynes as equivalents of α-diazo imines, which are difficult to access as α-diazo imines can readily cyclize into the corresponding 1,2,3-triazoles. To date, only limited success has been achieved in this type of gold-catalyzed nitrene transfer, mainly by the intramolecular reaction of alkyne and azide.^8^ For example, Toste and co-workers used azide as an effective nitrene equivalent and realized the first protocol for the generation of α-imino gold carbenes in 2005.^8*a*^ Later, elegant studies on the synthesis of indoles from alkynyl azides were demonstrated by Gagosz^8*c*^ and Zhang,^8*d*^ independently. Recently, several studies have invoked the intermolecular transfer of nitrene to alkynes by the use of iminopyridinium ylides as nitrene-transfer reagents, as disclosed by the groups of Zhang,^9*a*^ Davies,^9b,c^ and Liu.^9*d*^ However, similar to those of the above-mentioned gold-catalyzed intermolecular alkyne oxidations, a stoichiometric amount of pyridine was produced as the waste in these cases. Therefore, the exploration of intermolecular approaches to the generation of α-imino gold carbenes, especially in an atom-economic way, is very attractive to researchers. We envisioned that the α-imino gold carbene intermediate **B** might be generated through the gold-catalyzed intermolecular reaction of ynamides **1** ^10^ with isoxazoles **2**, which could be obtained in an efficient and modular manner following the synthetic routes shown in eqn (3) and (4) in Scheme 1.^11^ The carbene **B**, likely highly electrophilic, could then undergo an electrophilic cyclization to yield the final 2-aminopyrroles **3**, thus constituting a gold-catalyzed formal [3+2] cycloaddition (Scheme 1, eqn (2)). Herein, we report the successful implementation of this mechanistic design to a facile and practical synthesis of a wide range of polysubstituted 2-aminopyrroles, which are common structural motifs found in natural products and pharmacologically active molecules (Fig. 1)^12^ and are difficult to access *via* traditional methods for pyrrole synthesis.^13^ Most importantly, an α-imino gold carbene is most likely generated as the key intermediate on the basis of both mechanistic studies and theoretical calculations, thereby providing a strategically-novel, atom-economic route to the generation of gold carbenes.

## Results and discussion

At the outset, ynamide **1a** and 3,5-dimethylisoxazole **2a** were used as the reacting species and a series of experiments were performed in order to validate our approach. To our delight, the expected product **3a** was indeed formed in 70% ^1^H NMR yield in the presence of 5 mol% IPrAuNTf_2_ (Table 1, entry 1). Then, various typical gold catalysts with a range of electronic and steric characteristics were screened (Table 1, entries 2–7), and (**Ar**O)_3_PAuNTf_2_ (**Ar** = 2,4-di-*tert*-butylphenyl) gave the best yield of the desired product (Table 1, entry 7). Somewhat surprisingly, AgNTf_2_ could also catalyze this reaction in 50% yield (Table 1, entry 8). Notably, without a metal catalyst, the reaction failed to give even a trace of **3a**, and PtCl_2_ and Zn(OTf)_2_ were not effective in promoting this reaction (Table 1, entries 9–10).^14^ The reaction proved to be less efficient when it was performed at a reduced temperature (Table 1, entry 11). In addition, the use of 2 equiv. of **2a** also gave the desired pyrrole **3a** in 90% yield (Table 1, entry 12).

**Table 1:** Optimization of reaction conditions^*a*^

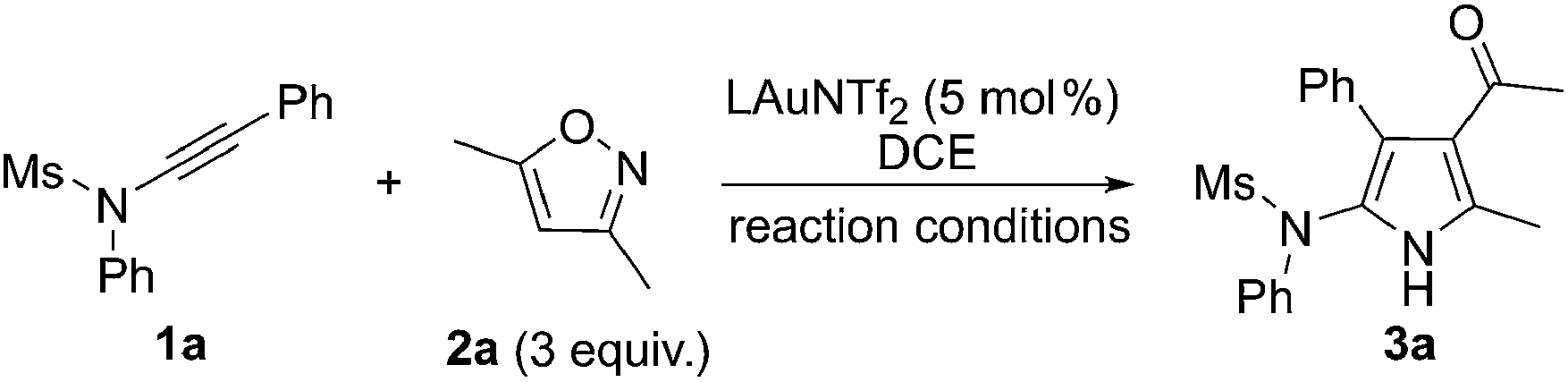			
Entry	Metal catalyst	Conditions	Yield^*b*^ (%)
1	IPrAuNTf_2_	DCE, 80 °C, 3 h	70
2	Ph_3_PAuNTf_2_	DCE, 80 °C, 3 h	69
3	Et_3_PAuNTf_2_	DCE, 80 °C, 3 h	54
4	Cy-JohnPhosAuNTf_2_	DCE, 80 °C, 3 h	71
5	BrettPhosAuNTf_2_	DCE, 80 °C, 12 h	27
6	Au(III)^*c*^	DCE, 80 °C, 3 h	34
7	(**Ar**O)_3_PAuNTf_2_ ^*d*^	DCE, 80 °C, 3 h	95
8	AgNTf_2_	DCE, 80 °C, 3 h	50
9^*e*^	PtCl_2_	toluene, 80 °C, 3 h	<5
10^*e*^	Zn(OTf)_2_ (10 mol%)	DCE, 80 °C, 3 h	<5
11	(**Ar**O)_3_PAuNTf_2_ ^*d*^	DCE, 60 °C, 5 h	75
12^*f*^	(**Ar**O)_3_PAuNTf_2_ ^*d*^	DCE, 80 °C, 3 h	90

^*a*^Reaction conditions: [**1a**] = 0.05 M; DCE = 1,2-dichloroethane.

^*b*^Measured by ^1^H NMR using diethyl phthalate as the internal standard.

^*c*^Dichloro(2-picolinato)gold(iii).

^*d*^ **Ar** = 2,4-di-*tert*-butylphenyl.

^*e*^ **1a** was decomposed.

^*f*^2.0 equiv. of **2a** was used.

**Fig. 2:**
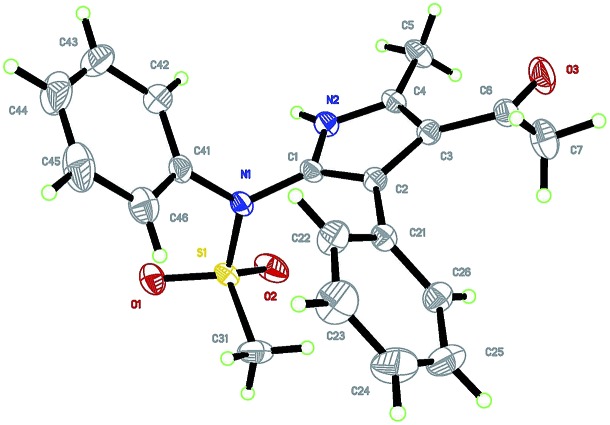
Crystal structure of compound **3a**.

With the optimized reaction conditions in hand, the scope of the transformation was explored. As seen from the results collected in Table 2, the reaction proceeded smoothly with various ynamide substrates **1**, and the yields ranged from 58% to 96%. For example, ynamides with different protecting groups, even the Ns group (Table 2, entries 4–5), readily gave the desired 2-aminopyrroles **3a–f** (Table 2, entries 1–6). Of note, an excellent yield could be achieved in the case of an ynamide with an oxazolidinone moiety and no dimerization reaction was observed (Table 2, entry 6).^15^ When R^1^ is an allyl group, the desired **3j** could also be formed in 86% yield, and no cyclopropanation product was formed (Table 2, entry 10).^16,5g^ Other aryl-substituted ynamides were also suitable substrates for this reaction, giving the corresponding functionalized pyrroles **3k–l** in excellent yields (Table 2, entries 11–12). Interestingly, for styryl or cyclopropyl-substituted ynamides, this reaction still led to 75% yield and 58% yield, respectively (Table 2, entries 13–14). The molecular structure of **3a** was further confirmed by X-ray diffraction (Fig. 2).^17^

**Table 2:** Reaction scope for different ynamides **1** ^*a*^

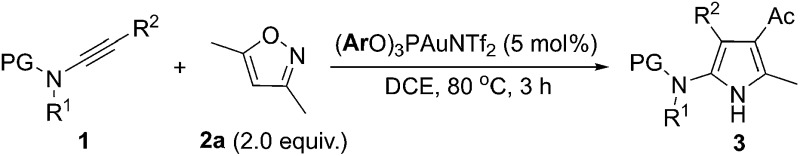
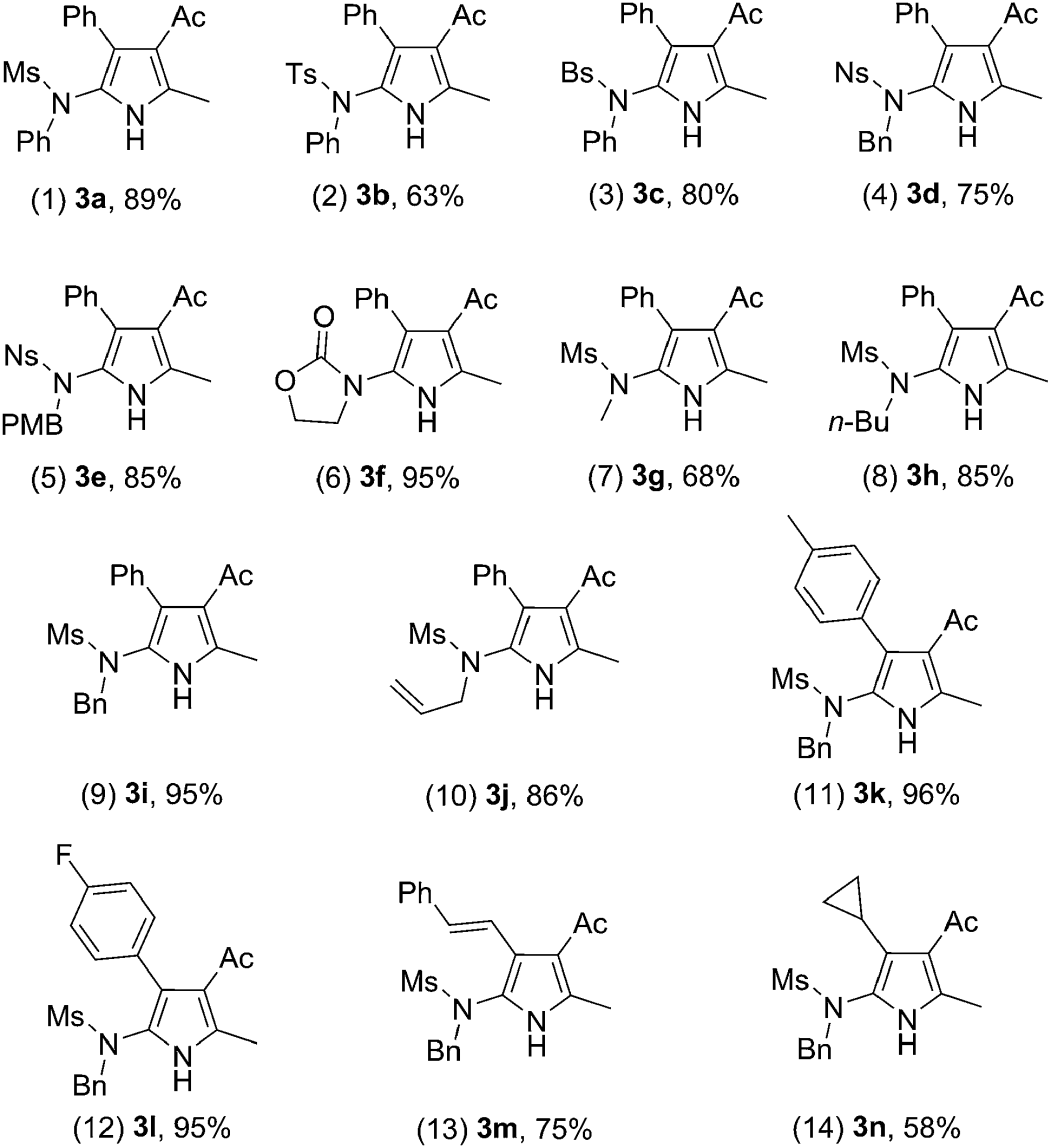

^*a*^Reactions run in vials; [**1**] = 0.05 M; isolated yields are reported.

We next extended the reaction to different 3,5-disubstituted isoxazoles **2**. To our delight, the reaction of ynamide **1i** with various isoxazole substrates **2** worked well under the above optimized reaction conditions, giving versatile polysubstituted 2-aminopyrroles **3o–z** in generally good to excellent yields. As summarized in Table 3, a range of aryl-substituted isoxazoles **2c–g** were successful (R^2^ = aryl), delivering the desired **3p–t** in 72–96% yield (Table 3, entries 2–6). In addition, when R^1^ is an aryl group, the reaction also worked well to afford the corresponding pyrroles **3v–w** in excellent yields (Table 3, entries 8–9). Pleasingly, methyl 3-pyrrolecarboxylate **3x** was formed in 90% yield from the corresponding isoxazole (Table 3, entry 10). It should be mentioned that 3-formylpyrroles **3y–z** could also be prepared in serviceable yields (Table 3, entries 11–12).

**Table 3:** Reaction scope for different isoxazoles **2** ^*a*^

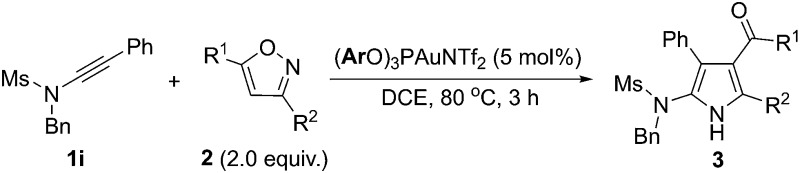
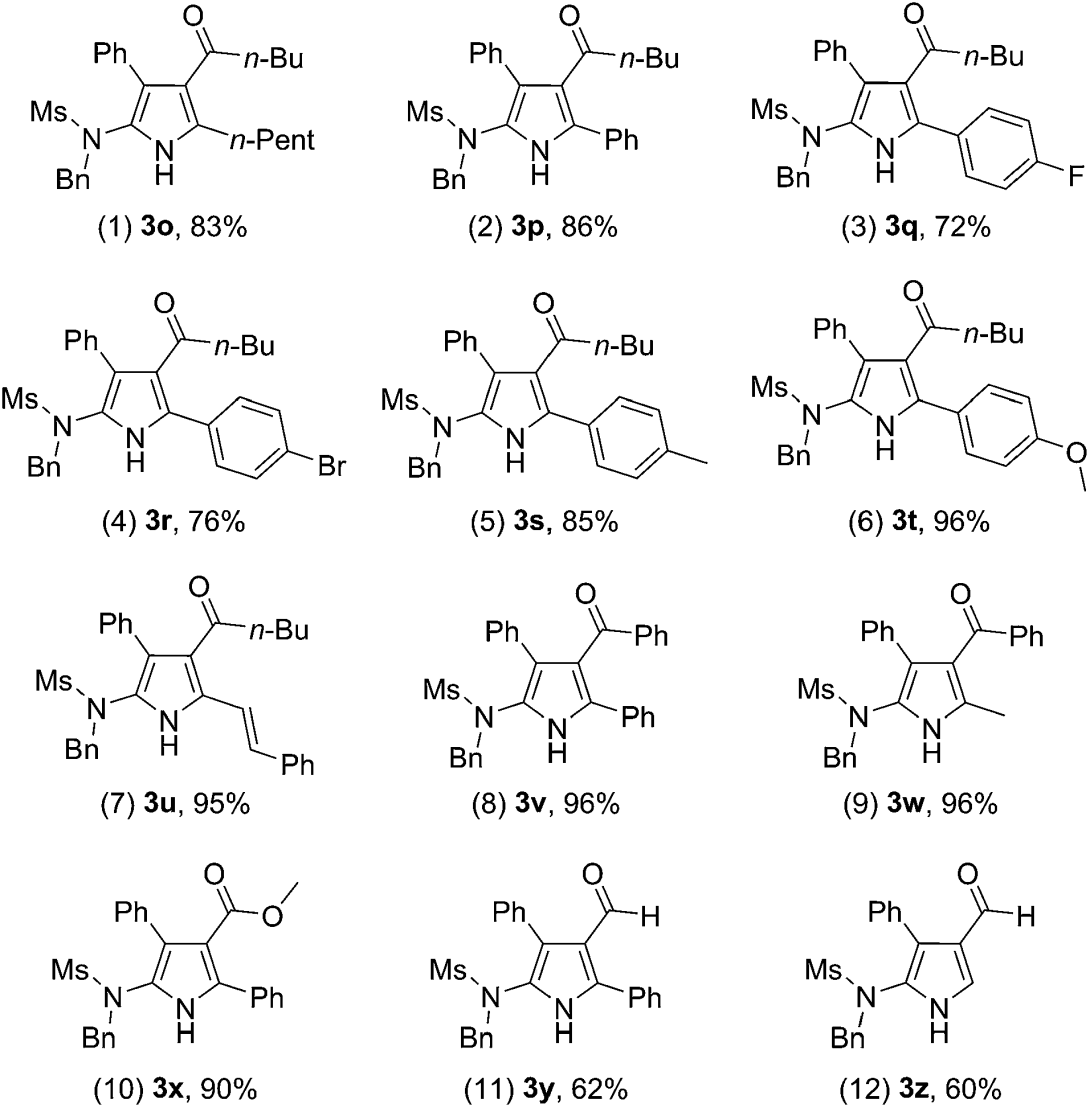

^*a*^Reactions run in vials; [**1i**] = 0.05 M; isolated yields are reported.

Interestingly, when the scope of the method was extended to fully-substituted isoxazoles **4**, the reaction also proceeded well, allowing the convenient synthesis of deacylated polysubstituted 2-aminopyrroles **5**. A series of readily-available substituted ynamides was first examined. The corresponding pyrroles **5a–d** were obtained in 72–85% yield (Table 4, entries 1–4). Then, isoxazoles **4** with substituents at the 4-position were also investigated, giving the products **5e–m** in mostly good to excellent yields (Table 4, entries 5–13). Notably, methyl 3-pyrrolecarboxylate **5n** could also be obtained in 77% yield from the corresponding 4-substituted isoxazole, which is complementary to the above protocol based on the 3,5-disubstituted isoxazoles **2** (Table 4, entry 14 *vs.* Table 3, entry 10). In particular, the 3,4-diphenyl substituted isoxazole also reacted smoothly, delivering the 3,4,5-triphenyl substituted pyrrole **5o** in a respectable 60% yield (Table 4, entry 15).

**Table 4:** Reaction scope for different ynamides **1** and 4-substituted isoxazoles **4** ^*a*^

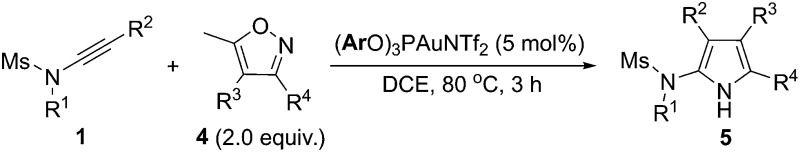
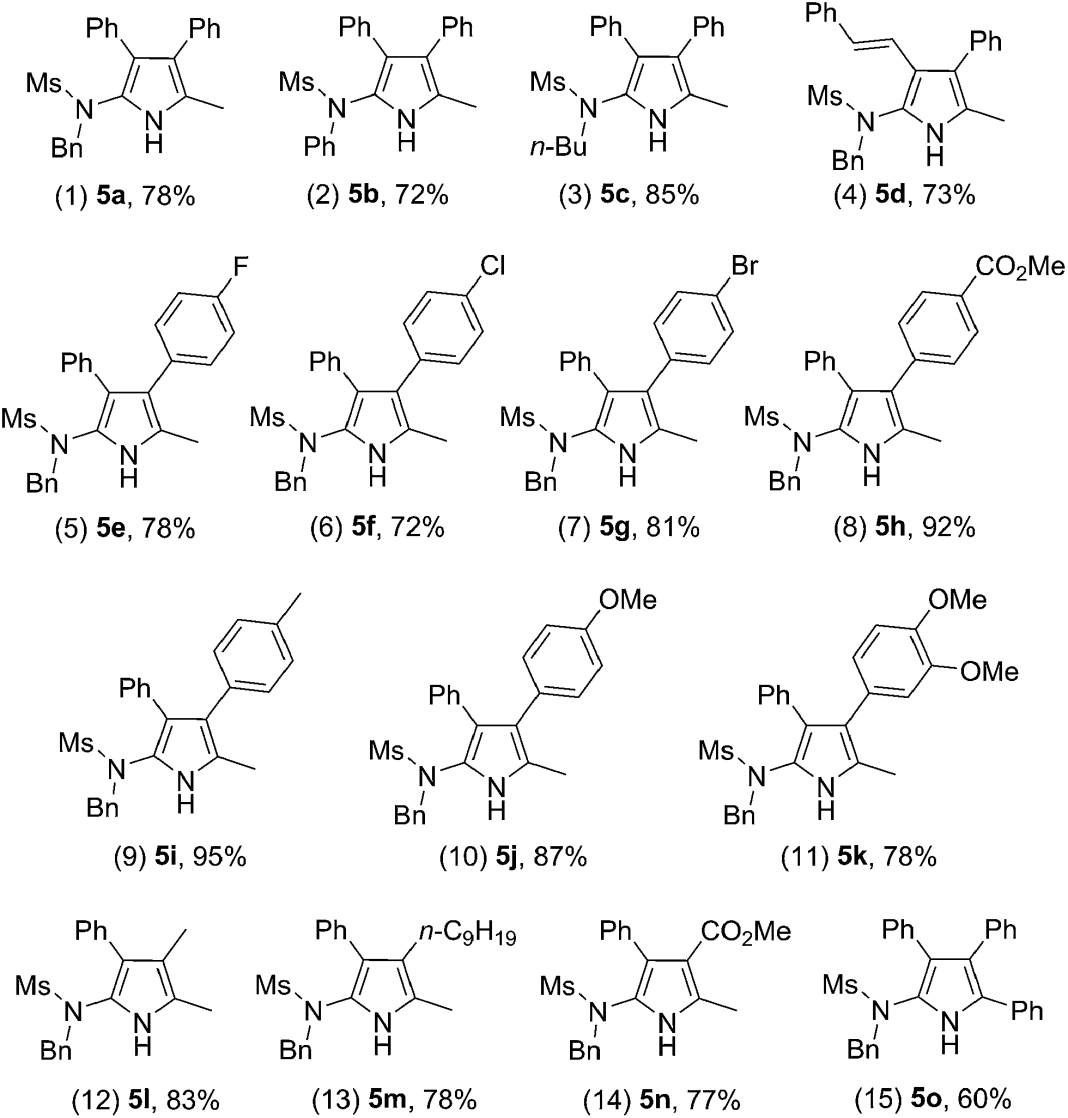

^*a*^Reactions run in vials; [**1**] = 0.05 M; isolated yields are reported.

To further test the practicality of the current catalytic system, a gram-scale reaction of 1.36 g of **1a** and 1.07 g of **2a** was carried out with a much lower catalyst loading (1 mol%), and 1.72 g of the desired pyrrole **3a** was formed in 85% yield, highlighting the synthetic utility of this chemistry (eqn (5)). Interestingly, the reaction could also be performed well even in water to afford the desired product **3a** in 80% yield and no hydration of the ynamide was observed (eqn (6)),^10a–c^ thus making this protocol more practical and environmentally benign.5
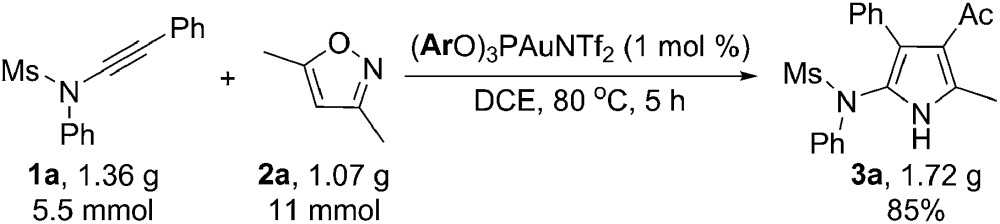

6
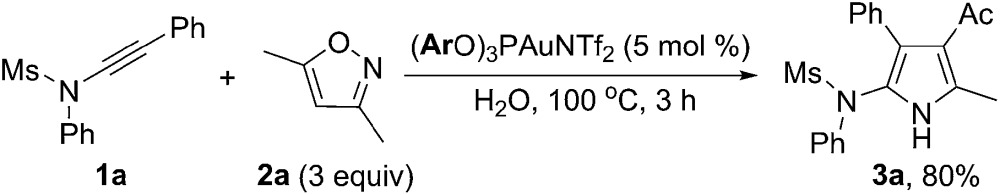



This chemistry can also be used to construct *N*-heteropyrrolizines, which are present in a variety of bioactive molecules.^18,12k^ For example, treatment of ynamide **1p** with isoxazole **4a** under the optimized reaction conditions gave the pyrrole **5p**, which could be converted into fused 2-aminopyrrole **6** in basic conditions in a one-pot process (63% two-step overall yield, eqn (7)). Compound **6** might serve as a precursor for the synthesis of lipoxygenase inhibitors (Fig. 1).^12*k*^
7
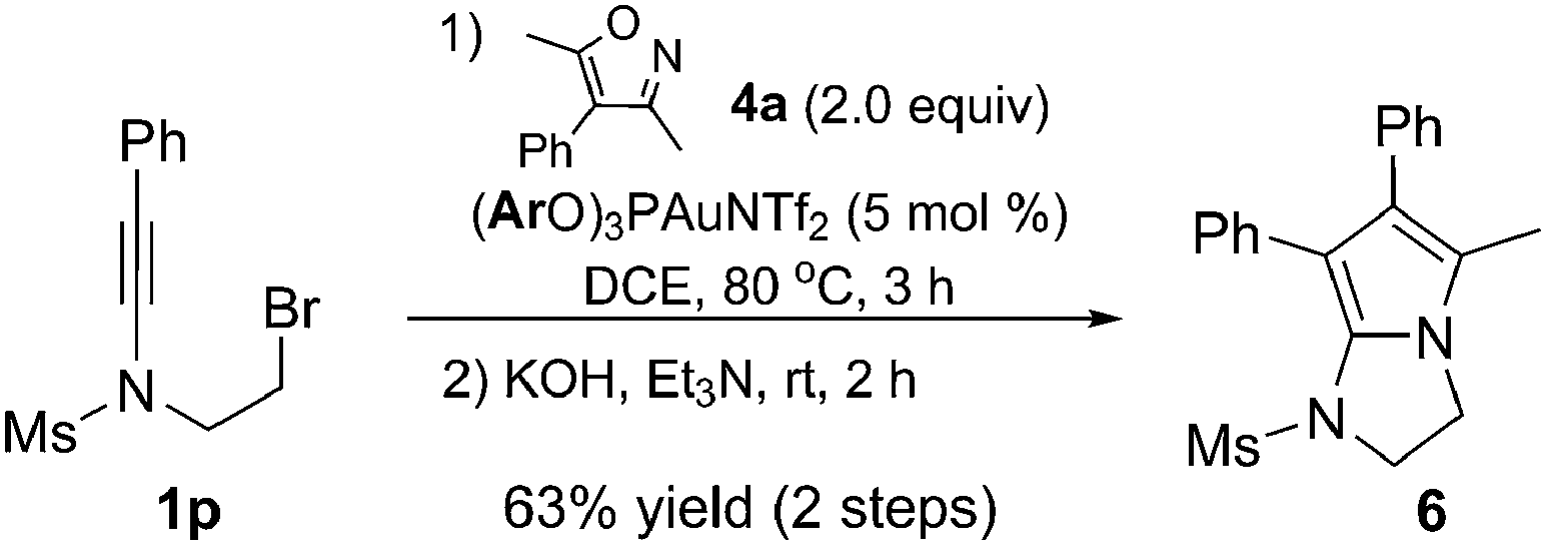



**Scheme 2:**
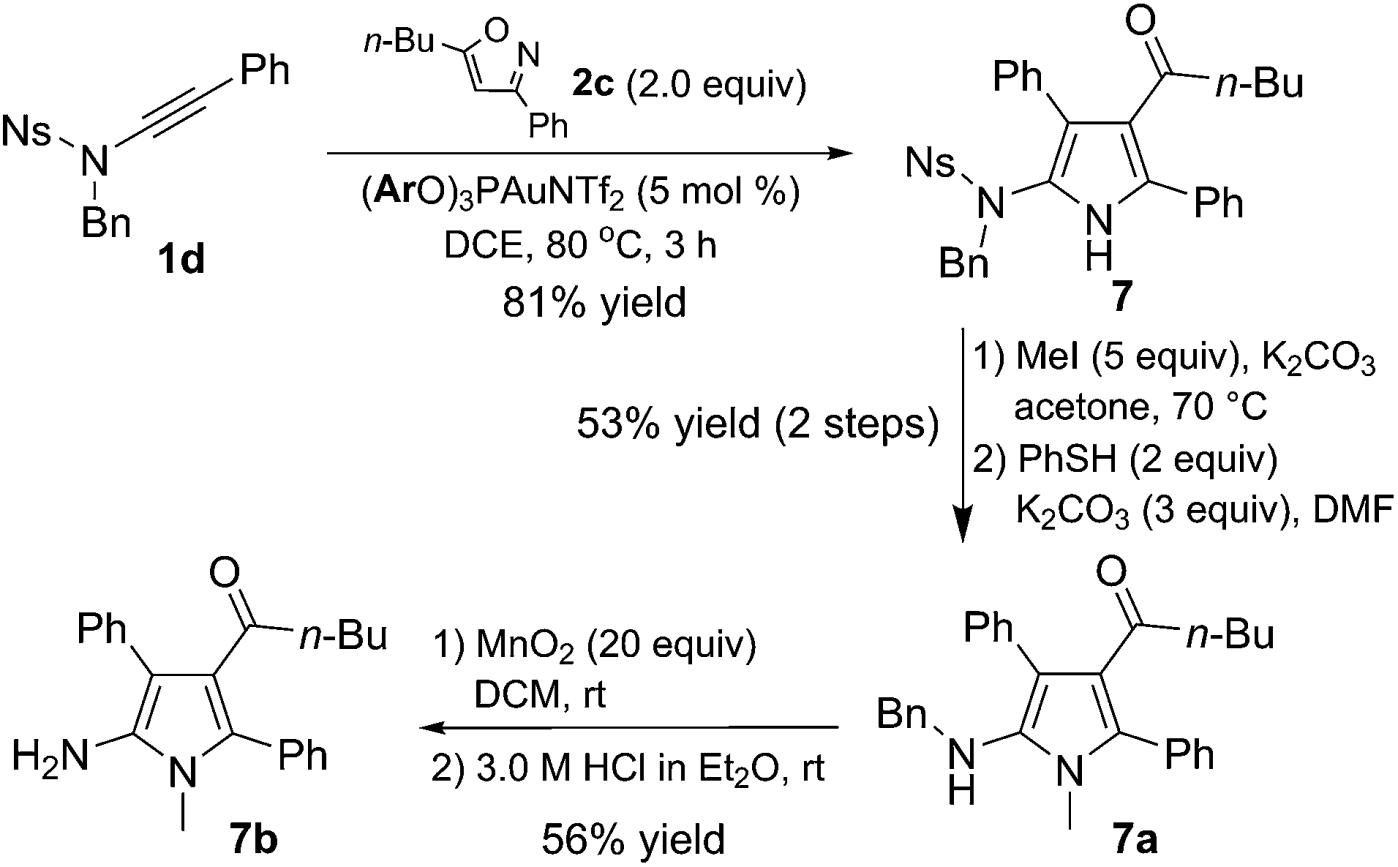
Transformation of a sulfonamide into an amine.

The sulfonamide could be readily transformed into a free amine (Scheme 2). For example, the reaction of ynamide **1d** with isoxazole **2c** under the optimized reaction conditions furnished pyrrole **7** in 81% yield. Nitrogen protection of **7** with a methyl group and subsequent removal of the Ns group using the standard conditions (PhSH, K_2_CO_3_) resulted in the formation of species **7a** (53% two-step overall yield). Subsequent deprotection of the benzyl group in **7a** could be realized by performing MnO_2_-mediated oxidation followed by hydrolysis to afford **7b** in 56% yield.^13*e*^

To probe the mechanism of this reaction, we first synthesized the alkyl-substituted ynamide **1q** as the alkyl-substituted gold carbene is well-known in the gold-catalyzed cycloisomerizations of enynes; [1,2] hydride shift followed by elimination of the gold catalyst was involved as the critical deauration step.^1e,19^ Indeed, as depicted in eqn (8), when ynamide **1q** reacted with **2a** under the standard reaction conditions, none of the desired pyrrole was detected and α,β-unsaturated amide **8** was isolated in 25% yield. Amide **8** is supposed to be derived from [1,2] hydride shift followed by elimination of the gold catalyst and subsequent hydrolysis. This result indicated that a gold carbene is most likely generated as the key intermediate in this process. On the other hand, the low chemoselectivity in the case of *n*-butyl substituted ynamide shows the importance of aryl substituents on the ynamides to keep a high reactivity for the reactions in Tables 2–4.^20^
8
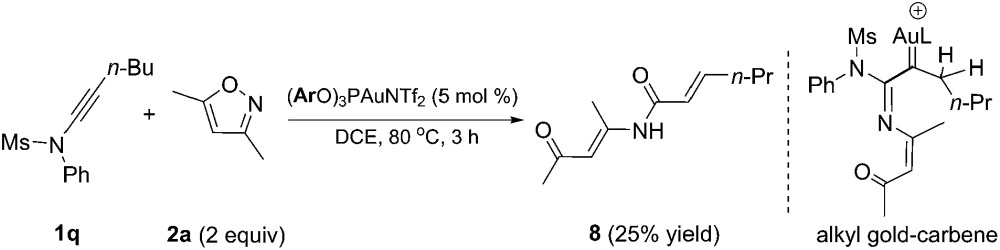



**Fig. 3:**
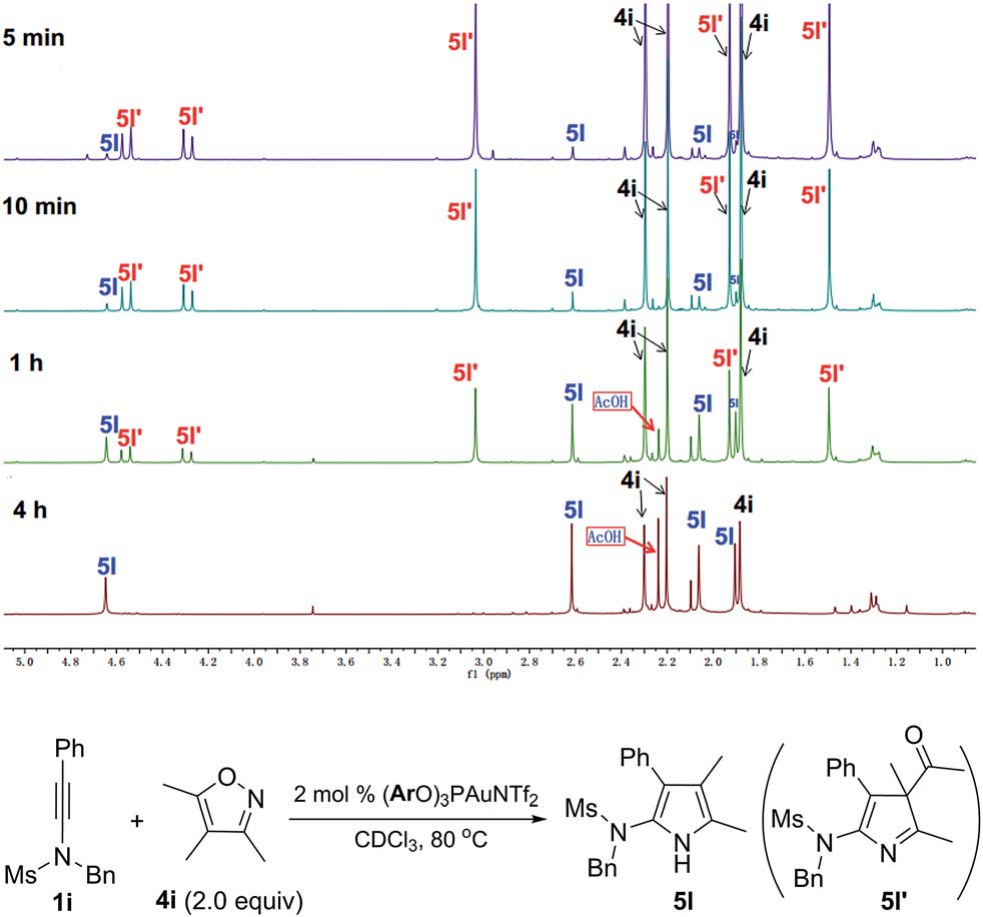
^1^H NMR monitoring of the reaction of ynamide **1i** with fully-substituted isoxazole **4i**.

In addition, it was found that a key intermediate 3*H*-pyrrole **5l′** could be detected and isolated in the case of the reaction of ynamide **1i** with fully-substituted isoxazole **4i** (Table 4, entry 12). To further demonstrate this process, we monitored the reaction by ^1^H NMR spectroscopy, as depicted in Fig. 3. Here, the reaction was performed in the presence of 2 mol% (**Ar**O)_3_PAuNTf_2_ in CDCl_3_ in order to better track the reaction intermediates. At the early stage of the reaction, we could clearly observe the formation of the 3*H*-pyrrole **5l′**, which was gradually transformed into the final 1*H*-pyrrole **5l**.

**Scheme 3:**
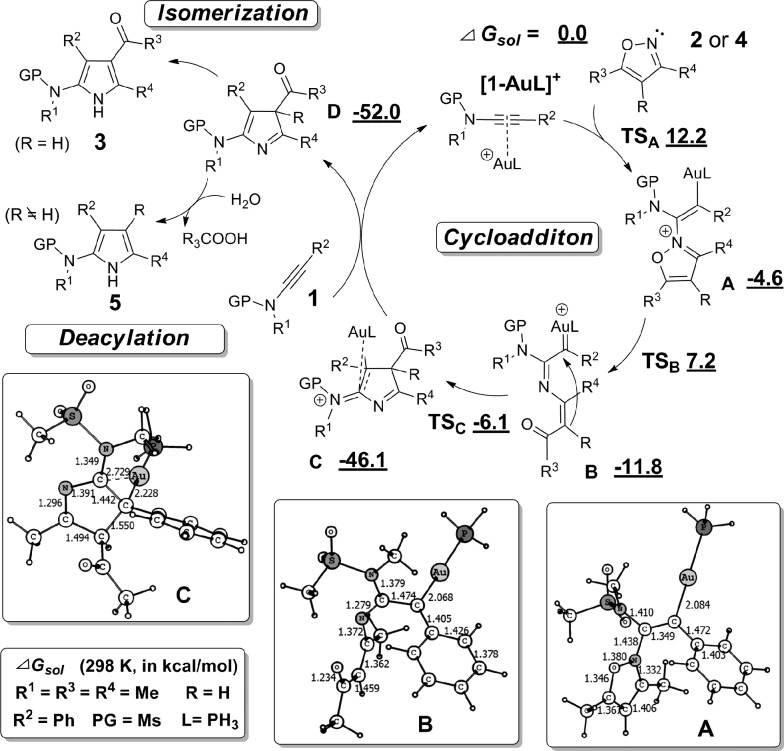
Plausible reaction mechanism. Theoretical investigations on the reaction pathways for the formation of product **3g** (Table 2, entry 7): relative free energies (Δ*G*_sol_, in kcal mol^–1^) of key intermediates and transition states were computed at the M06/6-31+G(d)/SDD level in 1,2-dichloroethane at 298 K.

A plausible mechanism to rationalize the formation of pyrrole **3** or **5** is illustrated in Scheme 3, in light of the above experimental observations and density functional theory (DFT) computations (see ESI† for details).^21^ Initially, nucleophilic attack of isoxazole **2** or **4** to the Au(i)-ligated alkyne of ynamide **1** forms vinyl gold intermediate **A** by overcoming a moderate barrier (12.2 kcal mol^–1^). Intermediate **A** isomerizes into the gold carbene intermediate **B** upon breakage of the isoxalic N–O bond,^22^ again requiring an activation energy around 12.0 kcal mol^–1^. Subsequent 1,5-cyclization^23^ within intermediate **B** readily occurs to afford the Au(i)-ligated 3*H*-pyrrole **C**, which upon ligand exchange with another ynamide **1** releases 3*H*-pyrrole **D**. The whole process is highly exothermic with free energy release amounting to 52 kcal mol^–1^. For 3,5-disubstituted isoxazoles **2**, 3*H*-pyrrole **D** readily isomerizes into the final aromatic 1*H*-pyrrole **3** by sigmatropic H-migrations.^24^ In the case of fully-substituted isoxazole substrates **4**, **D** is ultimately transformed into the final 1*H*-pyrrole **5**, presumably by a water-assisted deacylative aromatization.^25^

## Conclusions

In summary, we have developed a novel gold-catalyzed formal [3+2] cycloaddition between ynamides and isoxazoles, leading to the concise and flexible synthesis of polysubstituted 2-aminopyrroles. This methodology makes it possible to introduce four substituents onto a pyrrole ring very freely with high efficiency. Of particular interest, fully substituted isoxazoles also react under deacylation, closing a further gap in the reaction scope. Moreover, an α-imino gold carbene is the most likely intermediate based on both mechanistic studies and theoretical calculations, thus providing a new strategy for the generation of gold carbenes, especially in an atom-economic way. Studies to elucidate the detailed mechanism and further synthetic applications of the current protocol are in progress in our laboratory.
