# Supplementary material for: Atom-economic generation of gold carbenes: gold-catalyzed formal [3+2] cycloaddition between ynamides and isoxazoles
Source: Chem Sci. 2014 Nov 12;6(2):1265–71. doi: 10.1039/c4sc02596b (PMC5811108; doi:10.1039/c4sc02596b)
Supplement: Supplementary file 1 [file SC-006-C4SC02596B-s001.pdf]

Electronic Supplementary Material (ESI) for Chemical Science.
This journal is © The Royal Society of Chemistry 2014

Supporting Information

Atom-Economic Generation of Gold Carbenes: Gold-Catalyzed Formal [3+2] Cycloaddition between Ynamides and Isoxazoles

Ai-Hua Zhou, Qiao He, Chao Shu, Yong-Fei Yu, Shuang Liu, Tian Zhao, Wei Zhang, Xin Lu,* and Long-Wu Ye*

State Key Laboratory for Physical Chemistry of Solid Surfaces & The Key Laboratory for Chemical Biology of Fujian Province, Department of Chemistry, Xiamen University, Xiamen 361005, Fujian, P. R. China

**Content**	**Page number**
**General**	2
**Preparation of Starting Materials**	2
**General Procedure: Gold Catalysis**	15
**Computational Studies**	38
**^{1}H and ^{13}C NMR Spectra**	66

General Information. Ethyl acetate (ACS grade), hexanes (ACS grade) and anhydrous 1, 2-dichloroethane (ACS grade) were obtained commercially and used without further purification. Methylene chloride, tetrahydrofuran and diethyl ether were purified according to standard methods unless otherwise noted. Commercially available reagents were used without further purification. Reactions were monitored by thin layer chromatography (TLC) using silicycle pre-coated silica gel plates. Flash column chromatography was performed over silica gel (300-400 mesh). Infrared spectra were recorded on a Nicolet AVATER FTIR330 spectrometer as thin film and are reported in reciprocal centimeter (cm^{-1}). Mass spectra were recorded with Micromass QTOF2 Quadrupole/Time-of-Flight Tandem mass spectrometer using electron spray ionization.

^{1}H NMR spectra and ^{13}C NMR spectra were recorded on a Bruker AV-400 spectrometer and a Bruker AV-500 spectrometer in chloroform-d_3. For ^{1}H NMR spectra, chemical shifts are reported in ppm with the internal TMS signal at 0.0 ppm as a standard. For ^{13}C NMR spectra, chemical shifts are reported in ppm with the internal chloroform signal at 77.0 ppm as a standard.

Representative synthetic procedures for the preparation of ynamides 1 (1g-1m, 1o-p):[1]

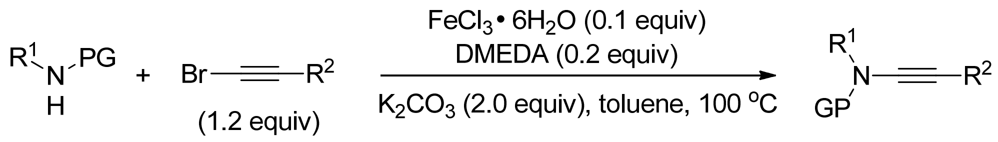

Representative synthetic procedures for the preparation of ynamides 1 (1d-1f, 1n):[2]

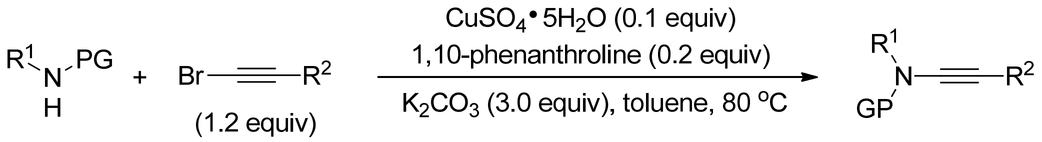

The data of the following ynamides **1a-1c** were reported in our previous work.[3]

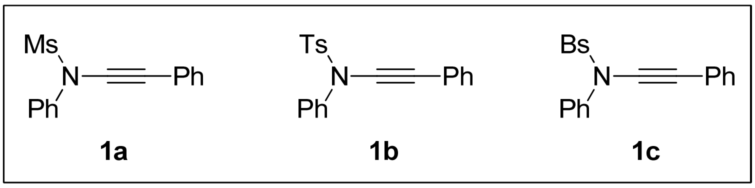

N-benzyl-2-nitro-N-(phenylethynyl)benzenesulfonamide (1d)

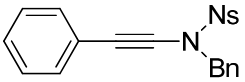

1d

^{1}H NMR (500 MHz, $CDCl_3$) δ 8.14 (dd, 1H, J = 1.0 Hz, J = 8.0 Hz), 7.78 – 7.58 (m, 3H), 7.44 – 7.38 (m, 2H), 7.38 – 7.27 (m, 3H), 7.27 – 7.15 (m, 5H), 4.81 (s, 2H); ^{13}C NMR (125 MHz, $CDCl_3$) δ 147.9, 134.6, 134.4, 131.9, 131.7, 131.2, 130.8, 128.8, 128.6, 128.5, 128.2, 128.0, 124.4, 122.1, 80.9, 72.5, 56.2; IR (neat): 2236, 1631, 1543, 1454, 1371 ,779, 752, 691; MS (ESI, m/z) 415 (M + Na^+); HRESIMS Calcd for $[C_{21}H_{16}N_2NaO_4S]^+$ (M + Na^+) 415.0723, found: 415.0728.

N-(4-methoxybenzyl)-2-nitro-N-(phenylethynyl)benzenesulfonamide (1e)

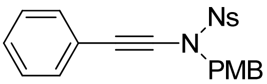

1e

^{1}H NMR (400 MHz, $CDCl_3$) δ 8.16 (dd, 1H, J = 1.2 Hz, J = 7.6 Hz), 7.80 – 7.68 (m, 3H), 7.39 – 7.34 (m, 2H), 7.30 – 7.19 (m, 5H), 6.91 – 6.86 (m, 2H), 4.75 (s, 2H), 3.81 (s, 3H); ^{13}C NMR (100 MHz, $CDCl_3$) δ 159.5, 147.5, 134.6, 131.7, 131.1, 130.8, 130.2(3), 130.1(7), 128.0, 127.8, 126.1, 124.1, 121.8, 113.7, 80.8, 72.3, 55.5, 54.8; IR (neat): 2916, 2239, 1611, 1543, 1513, 1371, 915, 851, 752, 691; MS (ESI, m/z) 445 (M + Na^+); HRESIMS Calcd for $[C_{22}H_{18}N_2NaO_5S]^+$ (M + Na^+) 445.0829, found: 445.0834.

3-(phenylethynyl)oxazolidin-2-one (1f)

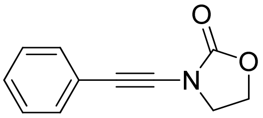

1f

This compound is known and the spectroscopic data match those reported.[1] ^{1}H NMR (400 MHz, $CDCl_3$) δ 7.48 – 7.39 (m, 2H), 7.30 – 7.25 (m, 3H), 4.49 (t, 2H, J = 8.0 Hz), 4.01 (t, 2H, J = 8.0 Hz); ^{13}C NMR (100 MHz, $CDCl_3$) δ 155.6, 131.0, 127.9, 127.7, 121.8, 79.0, 70.4, 62.9, 46.5.

N-methyl-N-(phenylethynyl)methanesulfonamide (1g)

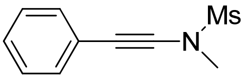

1g

This compound is known and the spectroscopic data match those reported.[4] ^{1}H NMR (500 MHz, $CDCl_3$) δ 7.44 – 7.38 (m, 2H), 7.32 – 7.28 (m, 3H), 3.30 (s, 3H), 3.12 (s, 3H); ^{13}C NMR (125 MHz, $CDCl_3$) δ 131.3, 128.2, 127.9, 122.2, 83.0, 69.2, 39.0, 36.5.

N-butyl-N-(phenylethynyl)methanesulfonamide (1h)

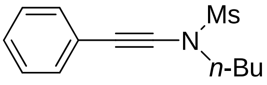

1h

This compound is known and the spectroscopic data match those reported.[1] ^{1}H NMR (400 MHz, $CDCl_3$) δ 7.42 – 7.39 (m, 2H), 7.33 – 7.28 (m, 3H), 3.54 (t, 2H, J = 7.2 Hz), 3.12 (s, 3H), 1.81 – 1.74 (m, 2H), 1.48 – 1.42 (m, 2H), 0.98 (t, 3H, J = 7.2 Hz); ^{13}C NMR (100 MHz, $CDCl_3$) δ 131.4, 128.2, 127.9, 122.6, 81.6, 70.9, 51.4, 38.1, 30.2, 19.4, 13.5.

N-benzyl-N-(phenylethynyl)methanesulfonamide (1i)

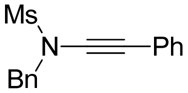

1i

This compound is known and the spectroscopic data match those reported.[1] ^{1}H NMR (400 MHz, $CDCl_3$) δ 7.50 – 7.48 (m, 2H), 7.43 – 7.34 (m, 5H), 7.29 – 7.26 (m, 3H), 4.71 (s, 2H), 2.93 (s, 3H); ^{13}C NMR (100 MHz, $CDCl_3$) δ 134.4, 131.2, 128.8, 128.7, 128.1, 127.9, 122.3, 81.9, 71.5, 55.7, 38.8.

N-allyl-N-(phenylethynyl)methanesulfonamide (1j)

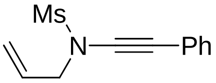

1j

This compound is known and the spectroscopic data match those reported.[5] ^{1}H NMR (400 MHz, $CDCl_3$) δ 7.45 – 7.36 (m, 2H), 7.36 – 7.28 (m, 3H), 6.00 (ddt, 1H, J = 6.4 Hz, J = 10.0 Hz, J = 16.8 Hz), 5.49 –

5.35 (m, 2H), 4.17 (dt, 2H, *J* = 1.2 Hz, *J* = 6.4 Hz), 3.14 (s, 3H); ^{13}C NMR (100 MHz, $CDCl_3$) δ 131.4, 130.8, 128.2, 127.9, 122.4, 120.4, 81.5, 71.0, 54.2, 38.8.

N-benzyl-N-(p-tolylethynyl)methanesulfonamide (1k)

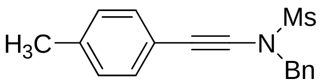

1k

This compound is known and the spectroscopic data match those reported.[1] ^{1}H NMR (400 MHz, $CDCl_3$) δ 7.48 – 7.46 (m, 2H), 7.41 – 7.33 (m, 3H), 7.25 – 7.23 (m, 2H), 7.08 (d, 2H, *J* = 7.6 Hz), 4.68 (s, 2H), 2.90 (s, 3H), 2.31 (s, 3H); ^{13}C NMR (100 MHz, $CDCl_3$) δ 138.1, 134.4, 131.3, 129.0, 128.9, 128.7, 128.6, 119.1, 81.1, 71.4, 55.7, 38.7, 21.3.

N-benzyl-N-((4-fluorophenyl)ethynyl)methanesulfonamide (1l)

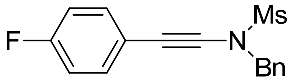

1l

^{1}H NMR (400 MHz, $CDCl_3$) δ 7.48 – 7.29 (m, 7H), 7.00 – 6.94 (m, 2H), 4.69 (s, 2H), 2.93 (s, 3H); ^{13}C NMR (100 MHz, $CDCl_3$) δ 162.3 (d, *J* = 247.8 Hz), 133.4 (d, *J* = 8.3 Hz), 133.4, 128.9, 128.8, 128.7,118.4 (d, *J* = 3.3 Hz), 115.5 (d, *J* = 21.9 Hz), 81.5, 70.4, 55.8, 38.9; IR (neat): 2916, 2237, 1600, 1509, 1358, 835, 785, 757; MS (ESI, m/z) 326 (M + Na^+); HRESIMS Calcd for $[C_{16}H_{14}FNNaO_2S]^+$ (M + Na^+) 326.0621, found: 326.0629.

(E)-N-benzyl-N-(4-phenylbut-3-en-1-yn-1-yl)methanesulfonamide (1m)

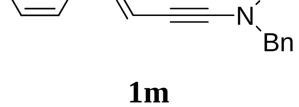

1m

^{1}H NMR (400 MHz, $CDCl_3$) δ 7.47 – 7.23 (m, 10H), 6.82 (d, 1H, *J* = 16.4 Hz), 6.21 (d, 1H, *J* = 16.0 Hz), 4.67 (s, 2H), 2.90 (s, 3H); ^{13}C NMR (100 MHz, $CDCl_3$) δ 139.8, 136.1, 134.4, 128.9, 128.8, 128.7, 128.6, 128.3, 126.0, 107.1, 83.9, 71.1, 55.8, 38.9; IR (neat): 2917, 2219 ,1630, 1493, 1357, 871, 749,

701; MS (ESI, m/z) 334 (M + Na$^+$); HRESIMS Calcd for $[C_{18}H_{17}NNaO_2S]^+$ (M + Na$^+$) 334.0872, found: 334.0875.

N-benzyl-N-(cyclopropylethynyl)methanesulfonamide (1n)

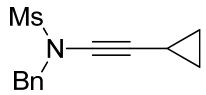

1n

This compound is known and the spectroscopic data match those reported.[4] ^{1}H NMR (400 MHz, $CDCl_3$) δ 7.46 – 7.30 (m, 5H), 4.56 (s, 2H), 2.86 (s, 3H), 1.33 – 1.27 (m, 1H), 0.82 – 0.74 (m, 2H), 0.66 – 0.58 (m, 2H); ^{13}C NMR (100 MHz, $CDCl_3$) δ 134.5, 128.4, 128.2, 128.1, 75.2, 68.3, 55.2, 37.8, 8.4, -1.2.

N-(2-bromoethyl)-N-(phenylethynyl)methanesulfonamide (1p)

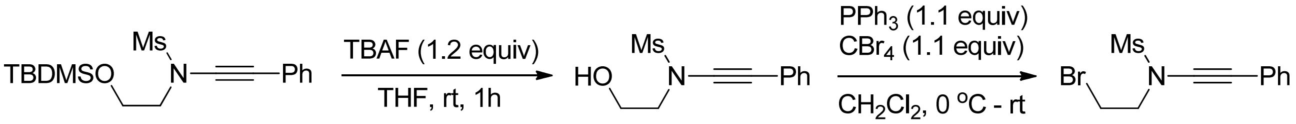

Compound **1p** was prepared according to the known procedures.[6] ^{1}H NMR (400 MHz, $CDCl_3$) δ 7.47 – 7.40 (m, 2H), 7.34 – 7.27 (m, 3H), 3.95 (t, 2H, *J* = 6.8 Hz), 3.65 (t, 2H, *J* = 6.8 Hz), 3.22 (s, 3H); ^{13}C NMR (100 MHz, $CDCl_3$) δ 131.6, 128.3, 122.0, 80.1, 71.8, 52.4, 39.0, 28.0; IR (neat): 2918, 2233, 1631, 1492, 1442, 1356, 862, 754, 690; MS (ESI, m/z) 324 (M + Na$^+$); HRESIMS Calcd for $[C_{11}H_{12}BrNNaO_2S]^+$ (M + Na$^+$) 323.9664, found: 323.9664.

N-(hex-1-yn-1-yl)-N-phenylmethanesulfonamide (1q)

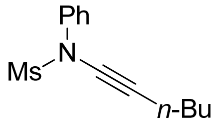

1q

This compound is known and the spectroscopic data match those reported.[5] ^{1}H NMR (400 MHz, $CDCl_3$) δ 7.55 – 7.47 (m, 2H), 7.46 – 7.38 (m, 2H), 7.37 – 7.29 (m, 1H), 3.06 (s, 3H), 2.35 (t, 2H, *J* = 7.2 Hz), 1.58 – 1.51 (m, 2H), 1.48 – 1.37 (m, 2H), 0.92 (t, 3H, *J* = 7.2 Hz); ^{13}C NMR (100 MHz, $CDCl_3$) δ 139.0, 129.1, 127.8, 125.2, 73.0, 70.9, 35.9, 30.7, 21.8, 18.0, 13.4.

Representative synthetic procedures for the preparation of isoxazoles 2:[7]

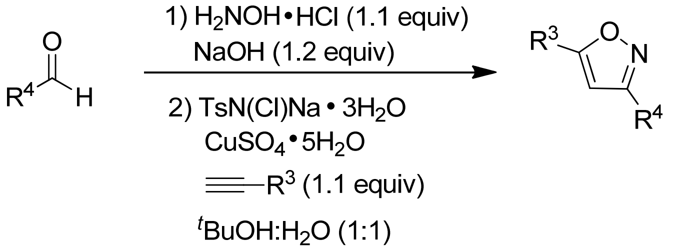

Compounds **2a, 2j** and **2m** are commercially available.

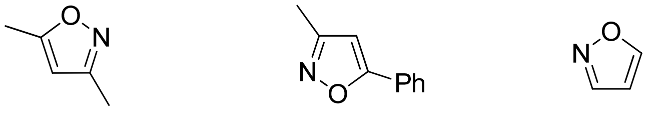

2a **2j** **2m**

5-butyl-3-pentylisoxazole (2b)

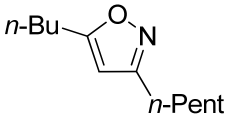

2b

^{1}H NMR (400 MHz, $CDCl_3$) δ 5.81 (s, 1H), 2.69 (t, 2H, *J* = 7.6 Hz), 2.62 (t, 2H, *J* = 7.6 Hz), 1.75 – 1.57 (m, 4H), 1.46 – 1.29 (m, 6H), 0.99 – 0.84 (m, 6H); ^{13}C NMR (100 MHz, $CDCl_3$) δ 173.2, 163.9, 100.1, 31.1, 30.3, 27.1, 26.5, 25.6, 22.2, 13.7, 13.6; IR (neat): 2918, 2849, 1632, 1602, 1275, 749; MS (ESI, m/z) 218 (M + Na^+); HRESIMS Calcd for $[C_{12}H_{21}NNaO]^+$ (M + Na^+) 218.1515, found: 218.1519.

5-butyl-3-phenylisoxazole (2c)

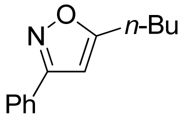

2c

This compound is known and the spectroscopic data match those reported.[8] ^{1}H NMR (400 MHz, $CDCl_3$) δ 7.83 – 7.75 (m, 2H), 7.47 – 7.41 (m, 3H), 6.29 (t, 1H, *J* = 0.8 Hz), 2.84 – 2.76 (m, 2H), 1.82 – 1.66 (m,

2H), 1.47 – 1.40 (m, 2H), 0.96 (t, 3H, J = 7.6 Hz); ^{13}C NMR (100 MHz, $CDCl_3$) δ 174.2, 162.2, 129.6, 128.7, 126.6, 98.7, 29.5, 26.4, 22.1, 13.6.

3-(4-florophenyl)-5-butylisoxazole (2d)

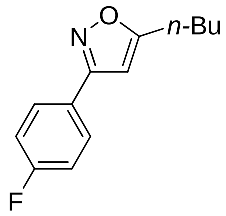

2d

This compound is known and the spectroscopic data match those reported.[8] ^{1}H NMR (500 MHz, $CDCl_3$) δ 7.81 – 7.74 (m, 2H), 7.13 (t, 2H, J = 8.5 Hz), 6.24 (s, 1H), 2.79 (t, 2H, J = 7.5 Hz), 1.77 – 1.69 (m, 2H), 1.48 – 1.39 (m, 2H), 0.96 (t, 3H, J = 7.5 Hz); ^{13}C NMR (100 MHz, $CDCl_3$) δ 174.5, 163.7 (d, J = 247.9 Hz), 128.6 (d, J = 8.3 Hz), 125.7 (d, J = 3.3 Hz), 115.9 (d, J = 21.7 Hz), 98.6, 29.6, 26.5, 22.2, 13.6.

3-(4-bromophenyl)-5-butylisoxazole (2e)

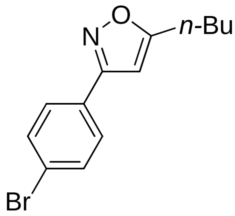

2e

This compound is known and the spectroscopic data match those reported.[8] ^{1}H NMR (400 MHz, $CDCl_3$) δ 7.68 – 7.62 (m, 2H), 7.57 – 7.53 (m, 2H), 6.25 (t, 1H, J = 0.8 Hz), 2.77 (t, 2H, J = 7.6 Hz), 1.75 – 1.68 (m, 2H), 1.45 – 1.39 (m, 2H), 0.95 (t, 3H, J = 7.6 Hz); ^{13}C NMR (100 MHz, $CDCl_3$) δ 174.5, 161.3, 131.9, 128.3, 128.1, 123.9, 29.5, 26.4, 22.1, 13.6.

5-butyl-3-(p-tolyl)isoxazole (2f)

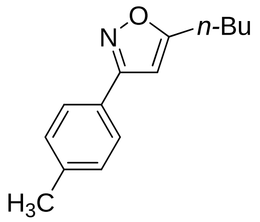

2f

^{1}H NMR (400 MHz, $CDCl_3$) δ 7.67 (d, 2H, J = 8.4 Hz), 7.24 (d, 2H, J = 8.0 Hz), 6.25 (s, 1H), 2.77 (t, 2H, J = 7.6 Hz), 2.38 (s, 3H), 1.75 – 1.68 (m, 2H), 1.45 – 1.39 (m, 2H), 0.95 (t, 3H, J = 7.2 Hz); ^{13}C NMR (100 MHz, $CDCl_3$) δ 174.0, 162.2, 139.7, 129.4, 126.5, 98.6, 29.5, 26.4, 22.1, 21.3, 13.6; IR (neat): 2958, 2933, 1613 ,1574, 1529, 1433, 870, 800; MS (ESI, m/z) 238 (M + Na^+), found: 238.1; HRESIMS Calcd for $[C_{14}H_{17}NNaO]^+$ (M + Na^+) 238.1202, found: 238.1205.

5-butyl-3-(4-methoxyphenyl)isoxazole (2g)

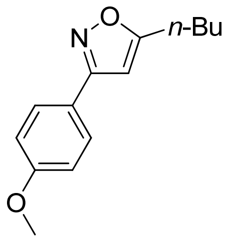

2g

This compound is known and the spectroscopic data match those reported.[8] ^{1}H NMR (500 MHz, $CDCl_3$) δ 7.82 – 7.70 (m, 2H), 7.05 – 6.90 (m, 2H), 6.24 (t, 1H, J = 1.0 Hz), 3.87 (s, 3H), 2.80 (t, 2H, J = 7.5 Hz), 1.81 – 1.71 (m, 2H), 1.51 – 1.38 (m, 2H), 0.98 (t, 3H, J = 7.5 Hz); ^{13}C NMR (100 MHz, $CDCl_3$) δ 174.0, 161.9, 160.8, 128.1, 1220, 114.2, 98.5, 55.3, 29.6, 26.4, 22.2, 13.6.

(E)-5-butyl-3-styrylisoxazole (2h)

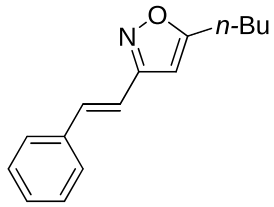

2h

^{1}H NMR (400 MHz, $CDCl_3$) δ 7.50 – 7.48 (m, 2H), 7.37 – 7.28 (m, 3H), 7.11 (s, 2H), 6.20 (s, 1H), 2.73 (t, 2H, J = 7.6 Hz), 1.73 – 1.65 (m, 2H), 1.45 – 1.37 (m, 2H), 0.94 (t, 3H, J = 7.2 Hz); ^{13}C NMR (100

MHz, $CDCl_3$) δ 173.4, 161.5, 135.8, 135.2, 128.7, 128.6, 126.8, 116.3, 97.6, 29.4, 26.2, 22.0, 13.6; IR (neat): 2960, 2931, 1643, 1596, 1444, 820, 754, 696; MS (ESI, m/z) 250 ($M + Na^+$); HRESIMS Calcd for $[C_{15}H_{17}NNaO]^+$ ($M + Na^+$) 250.1202, found: 250.1212.

3,5-diphenylisoxazole (2i)

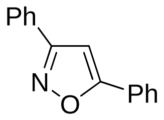

2i

This compound is known and the spectroscopic data match those reported.[9] ^{1}H NMR (400 MHz, $CDCl_3$) δ 7.88 – 7.83 (m, 4H), 7.51 – 7.43 (m, 6H), 6.83 (s, 1H); ^{13}C NMR (100 MHz, $CDCl_3$) δ 170.3, 162.9, 130.1, 129.9, 129.0, 128.9, 128.8, 127.3, 126.7, 125.7, 97.4.

5-methoxy-3-phenylisoxazole (2k)

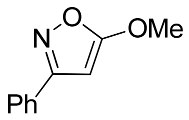

2k

Compound **2k** was prepared according to the known procedures.[9] This compound is known and the spectroscopic data match those reported.[9] ^{1}H NMR (400 MHz, $CDCl_3$) δ 7.75 – 7.73 (m, 2H), 7.51 – 7.38 (m, 3H), 5.52 (s, 1H), 4.01 (s, 3H); ^{13}C NMR (100 MHz, $CDCl_3$) δ 174.4, 164.0, 129.9, 129.4, 128.6, 126.3, 75.2, 58.7.

3-phenylisoxazole (2l)

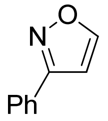

2l

Compound **2l** was prepared according to the known procedures.[10] This compound is known and the spectroscopic data match those reported.[10] ^{1}H NMR (400 MHz, $CDCl_3$) δ 8.44 (d, 1H, J = 1.6 Hz), 7.84 – 7.81 (m, 2H), 7.48 – 7.43 (m, 3H), 6.65 (d, 1H, J = 1.6 Hz); ^{13}C NMR (100 MHz, $CDCl_3$) δ 161.5, 158.8, 130.0, 128.9, 128.7, 126.8, 102.4.

Representative synthetic procedures for the preparation of isoxazoles 4:[11]

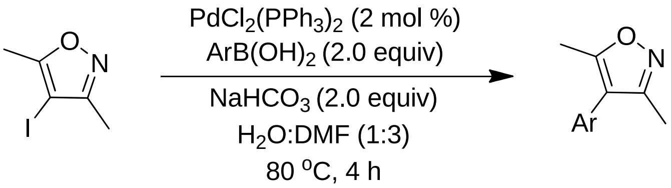

Compound **4i** (3,4,5-trimethylisoxazole) is commercially available.

3,5-dimethyl-4-phenylisoxazole (4a)

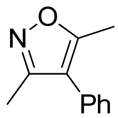

4a

This compound is known and the spectroscopic data match those reported.[12] ^{1}H NMR (400 MHz, $CDCl_3$) δ 7.46 – 7.41 (m, 2H), 7.40 – 7.31 (m, 1H), 7.28 – 7.22 (m, 2H), 2.41 (s, 3H), 2.28 (s, 3H); ^{13}C NMR (100 MHz, $CDCl_3$) δ 164.9, 158.3, 130.2, 128.8, 128.6, 127.2, 116.4, 11.2, 10.5.

4-(4-fluorophenyl)-3,5-dimethylisoxazole (4b)

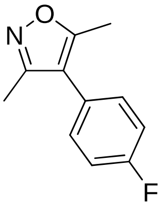

4b

This compound is known and the spectroscopic data match those reported.[13] ^{1}H NMR (400 MHz, $CDCl_3$) δ 7.26 – 7.19 (m, 2H), 7.19 – 7.10 (m, 2H), 2.39 (s, 3H), 2.25 (s, 3H); ^{13}C NMR (100 MHz, $CDCl_3$) δ 165.2, 162.2 (d, J = 235.7 Hz), 158.5, 130.8 (d, J = 8.1 Hz), 126.4 (d, J = 3.3 Hz), 115.8 (d, J = 21.5 Hz), 115.7, 11.4, 10.6.

4-(4-chlorophenyl)-3,5-dimethylisoxazole (4c)

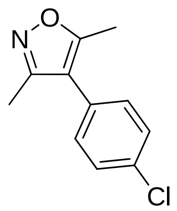

4c

^{1}H NMR (400 MHz, $CDCl_3$) δ 7.33 (d, 2H, J = 7.2 Hz), 7.11 (d, 2H, J = 7.2 Hz), 2.31 (s, 3H), 2.17 (s, 3H); ^{13}C NMR (100 MHz, $CDCl_3$) δ 164.9, 157.8, 133.0, 130.0, 128.6, 115.2, 11.0, 10.2; IR (neat): 1631, 1595, 1502, 1485, 1395, 890, 832; MS (ESI, m/z) 230 (M + Na^+); HRESIMS Calcd for $[C_{11}H_{10}ClNNaO]^+$ (M + Na^+) 230.0343, found: 230.0349.

4-(4-bromophenyl)-3,5-dimethylisoxazole (4d)

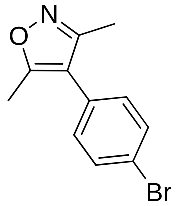

4d

This compound is known and the spectroscopic data match those reported.[11] ^{1}H NMR (400 MHz, $CDCl_3$) δ 7.57 (d, 2H, J = 8.8 Hz), 7.13 (d, 2H, J = 8.4 Hz), 2.39 (s, 3H), 2.25 (s, 3H); ^{13}C NMR (100 MHz, $CDCl_3$) δ 165.2, 158.3, 137.9, 131.9, 130.8, 130.6, 129.3, 121.6, 115.6, 11.5, 10.6.

4-(3,5-dimethylisoxazol-4-yl)benzoate (4e)

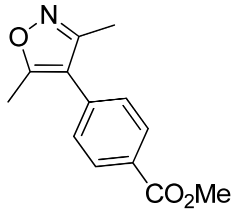

4e

This compound is known and the spectroscopic data match those reported.[13] ^{1}H NMR (400 MHz, $CDCl_3$) δ 8.11 (d, 2H, J = 8.0 Hz), 7.35 (d, 2H, J = 6.8 Hz), 3.95 (s, 3H), 2.44 (s, 3H), 2.30 (s, 3H); ^{13}C NMR (100 MHz, $CDCl_3$) δ 166.6, 165.7, 158.3, 135.2, 130.0, 129.2, 128.9, 115.9, 52.2, 11.6, 10.8.

4-(4-methylphenyl)-3,5-dimethylisoxazole (4f)

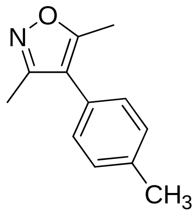

4f

This compound is known and the spectroscopic data match those reported.[13] ^{1}H NMR (400 MHz, $CDCl_3$) δ 7.25 (d, 2H, *J* = 8.4 Hz), 7.14 (d, 2H, *J* = 8.0 Hz), 2.40 (s, 3H), 2.39 (s, 3H), 2.26 (s, 3H); ^{13}C NMR (125 MHz, $CDCl_3$) δ 164.8, 158.6, 137.2, 129.4, 128.8, 127.3, 116.4, 21.0, 11.4, 10.6.

4-(4-methoxyphenyl)-3,5-dimethylisoxazole (4g)

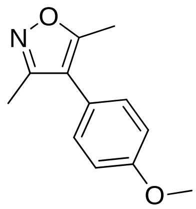

4g

This compound is known and the spectroscopic data match those reported.[13] ^{1}H NMR (400 MHz, $CDCl_3$) δ 7.17 (d, 2H, *J* = 8.4 Hz), 6.97 (d, 2H, *J* = 8.4 Hz), 3.85 (s, 3H), 2.38 (s, 3H), 2.25 (s, 3H); ^{13}C NMR (100 MHz, $CDCl_3$) δ 164.6, 158.8, 158.5, 130.0, 122.4, 116.0, 114.1, 55.0, 11.2, 10.5.

4-(3,4-dimethoxyphenyl)-3,5-dimethylisoxazole (4h)

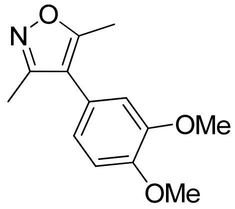

4h

^{1}H NMR (400 MHz, $CDCl_3$) δ 6.94 (d, 1H, *J* = 8.0 Hz), 6.80 (dd, 1H, *J* = 2.0 Hz, *J* = 8.0 Hz), 6.75 (d, 1H, *J* = 2.0 Hz), 3.92 (s, 3H), 3.90 (s, 3H), 2.40 (s, 3H), 2.26 (s, 3H); ^{13}C NMR (100 MHz, $CDCl_3$) δ 164.2, 158.0, 148.6, 148.0, 122.4, 121.1, 115.9, 111.9, 111.0, 55.4, 55.3, 10.8, 10.1; IR (neat): 2932, 1603, 1582, 1519, 1441, 1299, 863, 812, 765; MS (ESI, m/z) 256 (M + Na^+); HRESIMS Calcd for $[C_{13}H_{15}NNaO_3]^+$ (M + Na^+) 256.0944, found: 256.0945.

3,5-dimethyl-4-nonylisoxazole (4j)

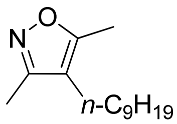

4j

Compound **4j** was prepared according to the known procedure.[14] ^{1}H NMR (400 MHz, $CDCl_3$) δ 2.32 – 2.24 (m, 5H), 2.20 (s, 3H), 1.50 – 1.38 (m, 2H), 1.37 – 1.19 (m, 12H), 0.88 (t, 3H, J = 6.8 Hz); ^{13}C NMR (100 MHz, $CDCl_3$) δ 164.3, 159.5, 113.6, 31.7, 29.6, 29.4, 29.3, 29.2, 29.1, 22.5, 22.0, 13.9, 10.7, 10.0; IR (neat): 2926, 2854, 1638, 1452, 1424, 1194, 747; MS (ESI, m/z) 246 (M + Na^+); HRESIMS Calcd for $[C_{14}H_{25}NNaO]^+$ (M + Na^+) 246.1828, found: 246.1838.

methyl 3,5-dimethylisoxazole-4-carboxylate (4k)

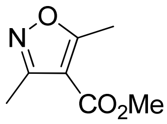

4k

Compound **4k** was prepared according to the known procedure.[15] This compound is known and the spectroscopic data match those reported.[15] ^{1}H NMR (400 MHz, $CDCl_3$) δ 3.86 (s, 3H), 2.65 (s, 3H), 2.42 (s, 3H); ^{13}C NMR (100 MHz, $CDCl_3$) δ 174.9, 162.5, 159.5, 108.3, 51.2, 12.9, 11.4.

5-methyl-3,4-diphenylisoxazole (4l)

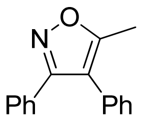

4l

Compound **4l** was prepared according to the known procedure.[16] This compound is known and the spectroscopic data match those reported.[16] ^{1}H NMR (400 MHz, $CDCl_3$) δ 7.47 – 7.41 (m, 2H), 7.39 – 7.27 (m, 6H), 7.19 – 7.14 (m, 2H), 2.44 (s, 3H); ^{13}C NMR (100 MHz, $CDCl_3$) δ 166.5, 161.1, 130.3, 129.7, 129.2, 129.1, 128.6, 128.4, 127.6, 115.7, 11.5.

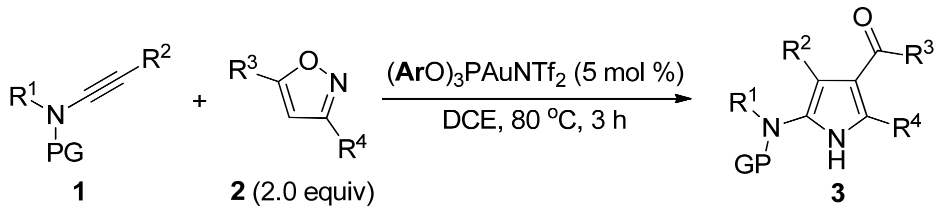

General procedure for the synthesis of 3:

Isoxazole **2** (0.60 mmol) and (**Ar**O)$_3$PAuNTf$_2$ (13.5 mg, 0.015 mmol) were added to a suspension of the ynamide **1** (0.30 mmol) in DCE (6.0 mL) at room temperature. The reaction mixture was then stirred at 80 ^{o}C and the progress of the reaction was monitored by TLC. The reaction typically took 3 h. Upon completion, the mixture was concentrated and the residue was purified by chromatography on silica gel (eluent: hexanes/ethyl acetate) to afford the desired product **3**.

N-(4-acetyl-5-methyl-3-phenyl-1H-pyrrol-2-yl)-N-phenylmethanesulfonamide (3a)

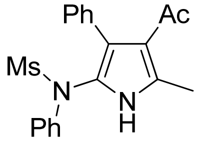

3a

Compound **3a** was prepared in 89% yield by the reaction of 3,5-dimethylisoxazole **2a** with ynamide **1a** according to the general procedure (Table 2, entry 1). ^{1}H NMR (400 MHz, CDCl$_3$) δ 8.84 (s, 1H), 7.35 – 7.27 (m, 5H), 7.23 – 7.18 (m, 1H), 7.16 – 7.04 (m, 4H), 2.76 (s, 3H), 2.54 (s, 3H), 1.84 (s, 3H); ^{13}C NMR (100 MHz, CDCl$_3$) δ 196.4, 141.0, 134.8, 134.5, 130.3, 129.1, 128.1, 127.6, 126.7, 125.4, 123.0, 121.8, 120.5, 40.2, 30.5, 14.1; IR (neat): 3317, 2926, 1632, 1605, 1526, 1377, 1152, 866, 788, 703; MS (ESI, m/z) 391 (M + Na$^+$); HRESIMS Calcd for [$C_{20}H_{20}N_2NaO_3S$]$^+$ (M + Na$^+$) 391.1087, found: 391.1087.

N-(4-acetyl-5-methyl-3-phenyl-1H-pyrrol-2-yl)-4-methyl-N-phenylbenzenesulfonamide (3b)

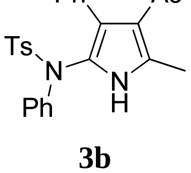

3b

Compound **3b** was prepared in 63% yield by the reaction of 3,5-dimethylisoxazole **2a** with ynamide **1b** according to the general procedure (Table 2, entry 2). ^{1}H NMR (400 MHz, $CDCl_3$) δ 8.97 (s, 1H), 7.51 (d, 2H, *J* = 8.4 Hz), 7.28 – 7.12 (m, 6H), 7.06 (t, 2H, *J* = 7.6 Hz), 6.89 – 6.85 (m, 2H), 6.60 – 6.55 (m, 2H), 2.56 (s, 3H), 2.46 (s, 3H), 1.74 (s, 3H); ^{13}C NMR (100 MHz, $CDCl_3$) δ 196.1, 144.2, 144.1, 135.9, 134.0, 133.7, 130.2, 129.5, 128.8, 127.9, 127.6, 127.0, 126.9, 126.7, 123.1, 122.1, 120.8, 30.5, 21.5, 14.3; IR (neat): 3332, 2922, 2850, 1632, 1352, 1164, 951, 696; MS (ESI, m/z) 467 (M + Na^+); HRESIMS Calcd for $[C_{26}H_{24}N_2NaO_3S]^+$ (M + Na^+) 467.1400, found: 467.1407.

N-(4-acetyl-5-methyl-3-phenyl-1H-pyrrol-2-yl)-4-bromo-N-phenylbenzenesulfonamide (3c)

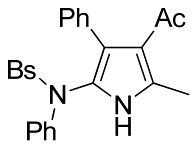

3c

Compound **3c** was prepared in 80% yield by the reaction of 3,5-dimethylisoxazole **2a** with ynamide **1c** according to the general procedure (Table 2, entry 3). ^{1}H NMR (400 MHz, $CDCl_3$) δ 9.18 (s, 1H), 7.53 – 7.51 (m, 2H), 7.44 – 7.41 (m, 2H), 7.25 – 7.18 (m, 4H), 7.14 – 7.10 (m, 2H), 6.96 – 6.92 (m, 2H), 6.63 – 6.61 (m, 2H), 2.55 (s, 3H), 1.75 (s, 3H); ^{13}C NMR (100 MHz, $CDCl_3$) δ 196.2, 140.9, 137.8, 134.2, 133.8, 132.2, 130.1, 129.3, 129.1, 128.3, 127.8, 127.4, 127.3, 126.8, 123.5, 121.5, 120.8, 30.6, 14.3; IR (neat): 3268, 2924, 2852, 1634, 1573, 1434, 1358, 1153, 852, 788, 701; MS (ESI, m/z) 531 (M + Na^+); HRESIMS Calcd for $[C_{25}H_{21}BrN_2NaO_3S]^+$ (M + Na^+) 531.0348, found: 531.0351.

N-(4-acetyl-5-methyl-3-phenyl-1H-pyrrol-2-yl)-N-benzyl-2-nitrobenzenesulfonamide (3d)

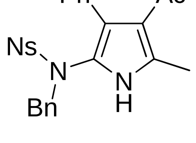

3d

Compound **3d** was prepared in 75% yield by the reaction of 3,5-dimethylisoxazole **2a** with ynamide **1d** according to the general procedure (Table 2, entry 4). ^{1}H NMR (500 MHz, $CDCl_3$) δ 8.80 (s, 1H), 7.69 (t, 2H, *J* = 7.5 Hz), 7.63 – 7.52 (m, 2H), 7.31 – 7.20 (m, 5H), 7.16 (t, 1H, *J* = 7.5 Hz), 7.07 (t, 2H, *J* = 7.5 Hz), 6.60 (d, 2H, *J* = 7.0 Hz), 4.68 (s, 2H), 2.36 (s, 3H), 1.70 (s, 3H); ^{13}C NMR (125 MHz, $CDCl_3$) δ 196.1, 147.5, 136.1, 134.4, 133.9, 133.6, 132.0, 131.8, 131.8, 129.5, 128.8, 128.6, 128.1, 127.8, 127.2,

124.6, 123.7, 120.4, 119.1, 56.7, 30.4, 14.0; IR (neat): 3231, 1639, 1543, 1438, 1367, 1150, 876, 763, 736, 700; MS (ESI, m/z) 512 (M + Na^+); HRESIMS Calcd for $[C_{26}H_{23}N_3NaO_5S]^+$ (M + Na^+) 512.1251, found: 512.1258.

N-(4-acetyl-5-methyl-3-phenyl-1H-pyrrol-2-yl)-N-(4-methoxybenzyl)-2-nitrobenzenesulfonamide (3e)

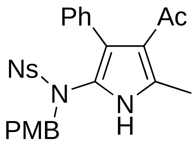

3e

Compound **3e** was prepared in 85% yield by the reaction of 3,5-dimethylisoxazole **2a** with ynamide **1e** according to the general procedure (Table 2, entry 5). ^{1}H NMR (400 MHz, $CDCl_3$) δ 8.41 (s, 1H), 7.77 – 7.66 (m, 2H), 7.66 – 7.55 (m, 2H), 7.22 – 7.11 (m, 3H), 7.07 (t, 2H, *J* = 7.6 Hz), 6.82 (d, 2H, *J* = 8.4 Hz), 6.62 (d, 2H, *J* = 7.2 Hz), 4.61 (s, 2H), 3.79 (s, 3H), 2.38 (s, 3H), 1.72 (s, 3H); ^{13}C NMR (100 MHz, $CDCl_3$) δ 196.12, 159.5, 147.6, 134.2, 133.8, 133.7, 132.3, 132.0, 131.7, 130.3, 129.6, 128.3, 127.9, 127.2, 124.7, 123.8, 120.6, 119.2, 114.1, 56.5, 55.2, 30.5, 14.1; IR (neat): 3316, 2927, 1634, 1612, 1513, 1435, 1339, 1152, 798, 764, 704; MS (ESI, m/z) 542 (M + Na^+); HRESIMS Calcd for $[C_{27}H_{25}N_3NaO_6S]^+$ (M + Na^+) 542.1356, found: 542.1359.

3-(4-acetyl-5-methyl-3-phenyl-1H-pyrrol-2-yl)oxazolidin-2-one (3f)

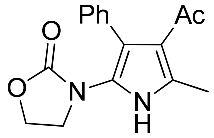

3f

Compound **3f** was prepared in 95% yield by the reaction of 3,5-dimethylisoxazole **2a** with ynamide **1f** according to the general procedure (Table 2, entry 6). ^{1}H NMR (400 MHz, $CDCl_3$) δ 10.17 (s, 1H), 7.42 – 7.32 (m, 5H), 4.29 – 4.25 (m, 2H), 3.47 – 3.43 (m, 2H), 2.36 (s, 3H), 1.89 (s, 3H); ^{13}C NMR (100 MHz, $CDCl_3$) δ 196.0, 158.3, 134.4, 132.6, 130.2, 128.3, 127.5, 120.4, 120.2, 63.0, 46.6, 30.6, 13.7; IR (neat): 3233, 2923, 1743(s), 1650, 1527, 1495, 1442, 1405, 1137, 762, 730, 705; MS (ESI, m/z) 307 (M + Na^+); HRESIMS Calcd for $[C_{16}H_{16}N_2NaO_3]^+$ (M + Na^+) 307.1053, found: 307.1053.

N-(4-acetyl-5-methyl-3-phenyl-1H-pyrrol-2-yl)-N-methylmethanesulfonamide (3g)

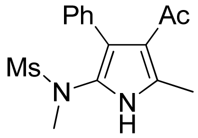

3g

Compound **3g** was prepared in 68% yield by the reaction of 3,5-dimethylisoxazole **2a** with ynamide **1g** according to the general procedure (Table 2, entry 7). ^{1}H NMR (400 MHz, $CDCl_3$) δ 8.66 (s, 1H), 7.47 – 7.29 (m, 5H), 3.12 (s, 3H), 2.56 (s, 3H), 2.51 (s, 3H), 1.86 (s, 3H); ^{13}C NMR (100 MHz, $CDCl_3$) δ 195.7, 134.9, 133.6, 130.3, 128.6, 127.9, 123.2, 121.6, 120.8, 39.4, 38.0, 30.6, 14.3; IR (neat): 3558, 2920, 2850, 1657, 1632, 1469, 1341, 1151, 795, 765, 704; MS (ESI, m/z) 329 (M + Na^+); HRESIMS Calcd for $[C_{15}H_{18}N_2NaO_3S]^+$ (M + Na^+) 329.0930, found: 329.0936.

N-(4-acetyl-5-methyl-3-phenyl-1H-pyrrol-2-yl)-N-butylmethanesulfonamide (3h)

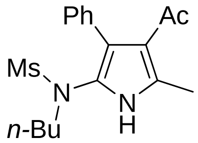

3h

Compound **3h** was prepared in 85% yield by the reaction of 3,5-dimethylisoxazole **2a** with ynamide **1h** according to the general procedure (Table 2, entry 8). ^{1}H NMR (400 MHz, $CDCl_3$) δ 9.09 (s, 1H), 7.43 – 7.35 (m, 3H), 7.33 – 7.30 (m, 2H), 3.33 – 3.29 (m, 2H), 2.64 (s, 3H), 2.50 (s, 3H), 1.85 (s, 3H), 1.51 – 1.41 (m, 2H), 1.29 – 1.19 (m, 2H), 0.86 (t, 3H, J = 6.8 Hz); ^{13}C NMR (100 MHz, $CDCl_3$) δ 196.0, 134.9, 134.0, 130.2, 128.5, 127.8, 122.1, 121.1, 120.8, 51.3, 39.0, 31.0, 30.6, 19.6, 14.3, 13.6; IR (neat): 3305, 2958, 2929, 1634, 1525, 1434, 1340, 1147, 965, 763, 704; MS (ESI, m/z) 371 (M + Na^+); HRESIMS Calcd for $[C_{18}H_{24}N_2NaO_3S]^+$ (M + Na^+) 371.1400, found: 371.1402.

N-(4-acetyl-5-methyl-3-phenyl-1H-pyrrol-2-yl)-N-benzylmethanesulfonamide (3i)

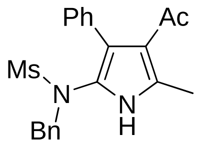

3i

Compound **3i** was prepared in 95% yield by the reaction of 3,5-dimethylisoxazole **2a** with ynamide **1i** according to the general procedure (Table 2, entry 9). ^{1}H NMR (400 MHz, $CDCl_3$) δ 8.71 (s, 1H), 7.42 – 7.36 (m, 3H), 7.29 – 7.27 (m, 3H), 7.24 – 7.18 (m, 4H), 4.49 (s, 2H), 2.66 (s, 3H), 2.34 (s, 3H), 1.82 (s, 3H); ^{13}C NMR (100 MHz, $CDCl_3$) δ 196.0, 135.9, 134.7, 134.1, 130.1, 128.7, 128.6, 128.4, 128.1, 127.7, 122.5, 121.0, 120.5, 55.2, 39.8, 30.5, 14.0; IR (neat): 3319, 2927, 1638, 1525, 1434, 1341, 1152, 955, 763, 700; MS (ESI, m/z) 405 (M + Na^+); HRESIMS Calcd for $[C_{21}H_{22}N_2NaO_3S]^+$ (M + Na^+) 405.1243, found: 405.1249.

N-(4-acetyl-5-methyl-3-phenyl-1H-pyrrol-2-yl)-N-allylmethanesulfonamide (3j)

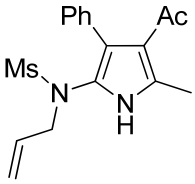

3j

Compound **3j** was prepared in 86% yield by the reaction of 3,5-dimethylisoxazole **2a** with ynamide **1j** according to the general procedure (Table 2, entry 10). ^{1}H NMR (400 MHz, $CDCl_3$) δ 9.21 (s, 1H), 7.45 – 7.32 (m, 5H), 5.85 – 5.75 (m, 1H), 5.20 – 5.18 (m, 1H), 5.16 (s, 1H), 3.99 (d, 2H, J = 6.4 Hz), 2.60 (s, 3H), 2.48 (s, 3H), 1.87 (s, 3H); ^{13}C NMR (100 MHz, $CDCl_3$) δ 196.0, 134.8, 134.1, 133.0, 130.2, 128.4, 127.8, 122.5, 121.4, 120.6, 119.7, 54.7, 39.8, 30.6, 14.2; IR (neat): 3306, 2923, 2851, 1640, 1433, 1340, 1153, 703; MS (ESI, m/z) 355 (M + Na^+); HRESIMS Calcd for $[C_{17}H_{20}N_2NaO_3S]^+$ (M + Na^+) 355.1087, found: 355.1089.

N-(4-acetyl-5-methyl-3-(p-tolyl)-1H-pyrrol-2-yl)-N-benzylmethanesulfonamide (3k)

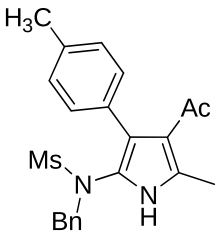

3k

Compound **3k** was prepared in 96% yield by the reaction of 3,5-dimethylisoxazole **2a** with ynamide **1k** according to the general procedure (Table 2, entry 11). ^{1}H NMR (400 MHz, $CDCl_3$) δ 8.42 (s, 1H), 7.30 – 7.27 (m, 3H), 7.23 – 7.19 (m, 4H), 7.14 – 7.12 (m, 2H), 4.48 (s, 2H), 2.70 (s, 3H), 2.40 (s, 3H), 2.33 (s,

3H), 1.83 (s, 3H); ^{13}C NMR (100 MHz, $CDCl_3$) δ 196.0, 137.5, 136.1, 133.9, 131.6, 130.0, 129.2, 128.7, 128.6, 128.1, 122.4, 120.9, 120.6, 55.2, 39.8, 30.6, 21.2, 14.1; IR (neat): 3321, 3030, 2925, 1634, 1531, 1437, 1341, 1152, 825, 698; MS (ESI, m/z) 419 (M + Na^+); HRESIMS Calcd for $[C_{22}H_{24}N_2NaO_3S]^+$ (M + Na^+) 419.1400, found: 419.1403.

N-(4-acetyl-3-(4-fluorophenyl)-5-methyl-1H-pyrrol-2-yl)-N-benzylmethanesulfonamide (3l)

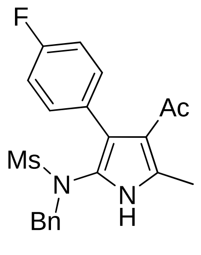

3l

Compound **3l** was prepared in 95% yield by the reaction of 3,5-dimethylisoxazole **2a** with ynamide **1l** according to the general procedure (Table 2, entry 12). ^{1}H NMR (400 MHz, $CDCl_3$) δ 8.28 (s, 1H), 7.31 – 7.30 (m, 3H), 7.20 – 7.17 (m, 4H), 7.13 – 7.08 (m, 2H), 4.48 (s, 2H), 2.71 (s, 3H), 2.36 (s, 3H), 1.84 (s, 3H); ^{13}C NMR (100 MHz, $CDCl_3$) δ 195.5, 162.4 (d, J = 248.5 Hz), 135.9, 134.0, 131.8, 130.6, 128.8, 128.4, 121.6, 121.2, 120.7, 115.6 (d, J = 21.4 Hz), 55.3, 40.0, 30.7, 14.2; IR (neat): 3307, 2926, 2853, 1636, 1529, 1438, 1341, 1152, 841, 740, 698; MS (ESI, m/z) 423 (M + Na^+); HRESIMS Calcd for $[C_{21}H_{21}FN_2NaO_3S]^+$ (M + Na^+) 423.1149, found: 423.1158.

(E)-N-(4-acetyl-5-methyl-3-styryl-1H-pyrrol-2-yl)-N-benzylmethanesulfonamide (3m)

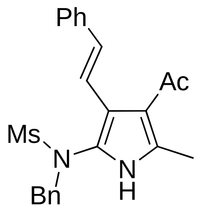

3m

Compound **3m** was prepared in 75% yield by the reaction of 3,5-dimethylisoxazole **2a** with ynamide **1m** according to the general procedure (Table 2, entry 13). ^{1}H NMR (400 MHz, $CDCl_3$) δ 8.72 (s, 1H), 7.41 – 7.31 (m, 10H), 7.28 – 7.20 (m, 1H), 6.69 (d, 1H, J = 16.4 Hz), 4.77 (s, 2H), 2.99 (s, 3H), 2.39 (s, 3H), 2.31 (s, 3H); ^{13}C NMR (100 MHz, $CDCl_3$) δ 195.8, 137.2, 136.0, 133.6, 131.2, 129.1, 128.7, 128.6, 128.2, 127.6, 126.1, 121.0, 120.3, 120.2, 119.1, 54.7, 40.4, 31.0, 14.4; IR (neat): 3314, 3028, 2926,

1630, 1443, 1337, 1151, 963, 801, 753, 698; MS (ESI, m/z) 431 (M + Na^+); HRESIMS Calcd for $[C_{23}H_{24}N_2NaO_3S]^+$ (M + Na^+) 431.1400, found: 431.1405.

N-(4-acetyl-3-cyclopropyl-5-methyl-1H-pyrrol-2-yl)-N-benzylmethanesulfonamide (3n)

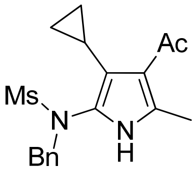

3n

Compound **3n** was prepared in 58% yield by the reaction of 3,5-dimethylisoxazole **2a** with ynamide **1n** according to the general procedure (Table 2, entry 14). ^1H NMR (400 MHz, $CDCl_3$) δ 8.12 (s, 1H), 7.30 – 7.29 (m, 3H), 7.24 – 7.22 (m, 2H), 4.70 (s, 2H), 3.05 (s, 3H), 2.52 (s, 3H), 2.27 (s, 3H), 1.75 – 1.68 (m, 1H), 0.91 – 0.88 (m, 2H), 0.64 – 0.60 (m, 2H); ^{13}C NMR (100 MHz, $CDCl_3$) δ 195.9, 136.2, 133.8, 129.1, 128.7, 128.3, 121.6, 121.3, 121.0, 55.3, 40.0, 31.1, 14.5, 8.0, 7.1; IR (neat): 3434, 2924, 2851, 1630, 1440, 1334, 1149, 954, 734, 699; MS (ESI, m/z) 369 (M + Na^+); HRESIMS Calcd for $[C_{18}H_{22}N_2NaO_3S]^+$ (M + Na^+) 369.1243, found: 369.1255.

N-benzyl-N-(4-pentanoyl-5-pentyl-3-phenyl-1H-pyrrol-2-yl)methanesulfonamide (3o)

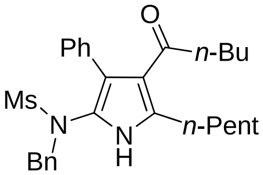

3o

Compound **3o** was prepared in 83% yield by the reaction of isoxazole **2b** with ynamide **1i** according to the general procedure (Table 3, entry 1). ^1H NMR (400 MHz, $CDCl_3$) δ 8.04 (s, 1H), 7.46 – 7.34 (m, 3H), 7.34 – 7.26 (m, 5H), 7.26 – 7.17 (m, 2H), 4.50 (s, 2H), 2.73 (t, 2H, J = 7.6 Hz), 2.65 (s, 3H), 2.04 (t, 2H, J = 7.2 Hz), 1.45 – 1.32 (m, 4H), 1.23 – 1.14 (m, 2H), 1.14 – 1.04 (m, 2H), 1.03 – 0.91 (m, 2H), 0.86 (t, 3H, J = 7.2 Hz), 0.77 (t, 3H, J = 7.2 Hz); ^{13}C NMR (100 MHz, $CDCl_3$) δ 199.1, 138.0, 136.2, 135.0, 130.1, 128.9, 128.7, 128.5, 128.2, 127.7, 122.0, 120.8, 120.2, 55.4, 42.3, 39.9, 31.3, 30.9, 27.1, 24.1, 22.3, 22.2, 13.8; IR (neat): 3321, 2955, 2929, 2858, 1649, 1605, 1522, 1456, 1339, 1152, 960, 762, 700; MS (ESI, m/z) 503 (M + Na^+); HRESIMS Calcd for $[C_{28}H_{36}N_2NaO_3S]^+$ (M + Na^+) 503.2339, found: 503.2339.

N-benzyl-N-(4-pentanoyl-3,5-diphenyl-1H-pyrrol-2-yl)methanesulfonamide (3p)

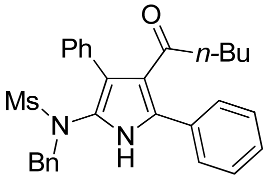

3p

Compound **3p** was prepared in 86% yield by the reaction of isoxazole **2c** with ynamide **1i** according to the general procedure (Table 3, entry 2). ^{1}H NMR (400 MHz, $CDCl_3$) δ 8.30 (s, 1H), 7.56 – 7.15 (m, 15H), 4.56 (s, 2H), 2.67 (s, 3H), 2.15 (t, 2H, J = 7.6 Hz), 1.41 – 1.30 (m, 2H), 1.08 – 0.92 (m, 2H), 0.66 (t, 3H, J = 7.6 Hz); ^{13}C NMR (100 MHz, $CDCl_3$) δ 200.1, 136.0, 133.9, 133.3, 131.5, 129.8, 128.9, 128.7, 128.7, 128.4, 128.3, 128.2, 127.6, 122.9, 122.7, 121.6, 55.3, 42.6, 39.9, 26.5, 22.0, 13.6; IR (neat): 3438, 2918, 2849, 1657, 1547, 1342, 1152, 760, 698; MS (ESI, m/z) 509 (M + Na^+); HRESIMS Calcd for $[C_{29}H_{30}N_2NaO_3S]^+$ (M + Na^+) 509.1869, found: 509.1875.

N-benzyl-N-(5-(4-fluorophenyl)-4-pentanoyl-3-phenyl-1H-pyrrol-2-yl)methanesulfonamide (3q)

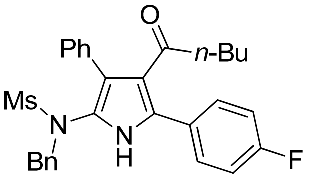

3q

Compound **3q** was prepared in 72% yield by the reaction of isoxazole **2d** with ynamide **1i** according to the general procedure (Table 3, entry 3). ^{1}H NMR (400 MHz, $CDCl_3$) δ 8.56 (s, 1H), 7.43 – 7.21 (m, 12H), 7.02 – 6.97 (m, 2H), 4.54 (s, 2H), 2.63 (s, 3H), 2.10 (t, 2H, J = 7.2 Hz), 1.36 – 1.28 (m, 2H), 0.99 – 0.95 (m, 2H), 0.65 (t, 3H, J = 7.2 Hz); ^{13}C NMR (100 MHz, $CDCl_3$) δ 199.8, 162.7 (d, J = 247.2 Hz), 136.0, 133.9, 132.4, 130.7 (d, J = 8.2 Hz), 129.8, 128.9, 128.7, 128.5, 128.3, 127.7, 127.6 (d, J = 3.2 Hz), 122.8, 122.7, 121.5, 115.2 (d, J = 21.5 Hz), 55.3, 42.6, 40.0, 26.4, 22.0, 13.6; IR (neat): 3295, 3063, 2957, 1659, 1606, 1500, 1451, 1342, 1227, 1153, 841, 785, 760, 700; MS (ESI, m/z) 527 (M + Na^+); HRESIMS Calcd for $[C_{29}H_{29}FN_2NaO_3S]^+$ (M + Na^+) 527.1775, found: 527.1768.

N-benzyl-N-(5-(4-bromophenyl)-4-pentanoyl-3-phenyl-1H-pyrrol-2-yl)methanesulfonamide (3r)

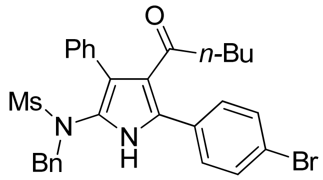

3r

Compound **3r** was prepared in 76% yield by the reaction of isoxazole **2e** with ynamide **1i** according to the general procedure (Table 3, entry 4). ^{1}H NMR (400 MHz, $CDCl_3$) δ 8.55 (s, 1H), 7.44 – 7.37 (m, 5H), 7.33 – 7.29 (m, 3H), 7.25 – 7.21 (m, 4H), 7.15 – 7.12 (m, 2H), 4.54 (s, 2H) , 2.61 (s, 3H), 2.10 (t, 2H, *J* = 7.2 Hz), 1.36 – 1.28 (m , 2H), 0.99 – 0.96 (m, 2H), 0.65 (t, 3H, *J* = 7.2 Hz); ^{13}C NMR (100 MHz, $CDCl_3$) δ 199.8, 135.9, 133.8, 131.9, 131.3, 130.3, 129.8, 128.9, 128.7, 128.5, 128.3, 127.8, 123.1, 122.7, 122.6, 121.7, 55.3, 42.6, 40.0, 26.4, 21.9, 13.6; IR (neat): 3297, 2956, 2870, 1658, 1605, 1492, 1450, 1341, 1152, 830, 760, 700; MS (ESI, m/z) 587 (M + Na$^+$); HRESIMS Calcd for $[C_{29}H_{29}BrN_2NaO_3S]^+$ (M + Na$^+$) 587.0974, found: 587.0987.

N-benzyl-N-(4-pentanoyl-3-phenyl-5-(p-tolyl)-1H-pyrrol-2-yl)methanesulfonamide (3s)

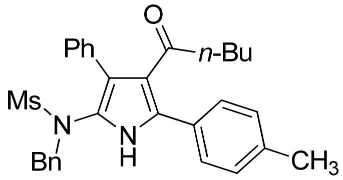

3s

Compound **3s** was prepared in 85% yield by the reaction of isoxazole **2f** with ynamide **1i** according to the general procedure (Table 3, entry 5). ^{1}H NMR (400 MHz, $CDCl_3$) δ 8.36 (s, 1H), 7.41 – 7.34 (m, 3H), 7.31 – 7.27 (m, 7H), 7.22 – 7.11 (m, 4H), 4.53 (s, 2H) , 2.64 (s, 3H), 2.33 (s, 3H), 2.14 (t, 2H, *J* = 7.2 Hz), 1.38 – 1.30 (m, 2H), 1.00 – 0.97 (m, 2H), 0.65 (t, 3H, *J* = 7.6 Hz); ^{13}C NMR (100 MHz, $CDCl_3$) δ 200.0, 138.3, 136.1, 134.0, 133.5, 129.7, 128.9, 128.8, 128.6, 128.5, 128.3, 128.2, 127.5, 122.6, 121.3, 55.3, 42.5, 39.8, 26.5, 22.0, 21.1, 13.6; IR (neat): 3296, 2957, 2870, 1642, 1606, 1502, 1449, 1343, 1153, 958, 822, 732, 700; MS (ESI, m/z) 523 (M + Na$^+$); HRESIMS Calcd for $[C_{30}H_{32}N_2NaO_3S]^+$ (M + Na$^+$) 523.2026, found: 523.2021.

N-benzyl-N-(5-(4-methoxyphenyl)-4-pentanoyl-3-phenyl-1H-pyrrol-2-yl)methanesulfonamide (3t)

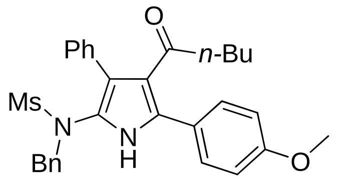

3t

Compound **3t** was prepared in 96% yield by the reaction of isoxazole **2g** with ynamide **1i** according to the general procedure (Table 3, entry 6). ^{1}H NMR (400 MHz, $CDCl_3$) δ 8.54 (s, 1H), 7.40 – 7.19 (m, 12H), 6.84 – 6.82 (m, 2H), 4.53 (s, 2H) , 3.77 (s, 3H), 2.62 (s, 3H), 2.12 (t, 2H, J = 7.6 Hz), 1.36 – 1.29 (m, 2H), 0.99 – 0.95 (m, 2H), 0.65 (t, 3H, J = 7.6 Hz); ^{13}C NMR (100 MHz, $CDCl_3$) δ 199.9, 159.6, 136.0, 134.1, 133.5, 130.0, 129.7, 128.8, 128.6, 128.3, 128.1, 127.4, 123.8, 122.6, 122.4, 121.0, 113.6, 55.3, 55.1, 42.4, 39.8, 26.5, 21.9, 13.5; IR (neat): 3297, 2957, 2870, 1643, 1610, 1502, 1452, 1342, 1153, 959, 836, 760, 700; MS (ESI, m/z) 539 (M + Na^+); HRESIMS Calcd for $[C_{30}H_{32}N_2NaO_4S]^+$ (M + Na^+) 539.1975, found: 539.1972.

(E)-N-benzyl-N-(4-pentanoyl-3-phenyl-5-styryl-1H-pyrrol-2-yl)methanesulfonamide (3u)

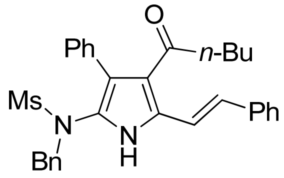

3u

Compound **3u** was prepared in 95% yield by the reaction of isoxazole **2h** with ynamide **1i** according to the general procedure (Table 3, entry 7). ^{1}H NMR (400 MHz, $CDCl_3$) δ 8.97 (s, 1H), 7.59 (d, 1H, J = 16.8 Hz), 7.42 – 7.13 (m, 15H), 6.61 (d, 1H, J = 16.8 Hz), 4.54 (s, 2H), 2.66 (s, 3H), 2.04 (t, 2H, J = 7.6 Hz), 1.41 – 1.33 (m, 2H), 1.01 – 0.97 (m, 2H), 0.67 (t, 3H, J = 7.6 Hz); ^{13}C NMR (100 MHz, $CDCl_3$) δ 199.6, 136.5, 135.7, 134.2, 132.5, 130.0, 129.6, 128.9, 128.7, 128.6, 128.5, 128.3, 127.9, 126.6, 123.4, 122.8, 121.7, 117.8, 55.2, 42.2, 40.1, 26.3, 22.0, 13.6; IR (neat): 3313, 2956, 2929, 1650, 1453, 1340, 1152, 804, 755, 700; MS (ESI, m/z) 535 (M + Na^+); HRESIMS Calcd for $[C_{31}H_{32}N_2NaO_3S]^+$ (M + Na^+) 535.2026, found: 535.2037.

N-(4-benzoyl-3,5-diphenyl-1H-pyrrol-2-yl)-N-benzylmethanesulfonamide (3v)

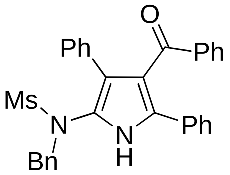

3v

Compound **3v** was prepared in 96% yield by the reaction of isoxazole **2i** with ynamide **1i** according to the general procedure (Table 3, entry 8). ^{1}H NMR (400 MHz, $CDCl_3$) δ 8.75 (s, 1H), 7.59 – 7.57 (m, 2H), 7.32 – 7.07 (m, 18H), 4.62 (s, 2H), 2.70 (s, 3H); ^{13}C NMR (100 MHz, $CDCl_3$) δ 193.6, 138.4, 136.0, 133.1, 133.0, 132.1, 130.4, 129.7, 129.4, 129.0, 128.6, 128.2, 128.1, 127.8, 127.6, 127.1, 123.8, 122.8, 119.6, 55.2, 40.0; IR (neat): 3305, 3061, 2929, 1735, 1641, 1596, 1495, 1456, 1343, 1152, 763, 739, 696; MS (ESI, m/z) 529 (M + Na^+); HRESIMS Calcd for $[C_{31}H_{26}N_2NaO_3S]^+$ (M + Na^+) 529.1556, found: 529.1546.

N-(4-benzoyl-5-methyl-3-phenyl-1H-pyrrol-2-yl)-N-benzylmethanesulfonamide (3w)

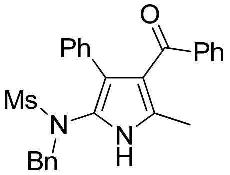

3w

Compound **3w** was prepared in 96% yield by the reaction of isoxazole **2j** with ynamide **1i** according to the general procedure (Table 3, entry 9). ^{1}H NMR (400 MHz, $CDCl_3$) δ 8.82 (s, 1H), 7.54 – 7.53 (m, 2H), 7.29 – 7.20 (m, 6H), 7.15 – 6.99 (m, 7H) , 4.59 (s, 2H), 2.64 (s, 3H), 2.16 (s, 3H); ^{13}C NMR (100 MHz, $CDCl_3$) δ 193.3, 139.3, 135.9, 133.5, 133.4, 131.5, 129.4, 129.3, 128.9, 128.5, 128.1, 127.9, 127.5, 126.6, 123.1, 120.8, 119.4, 55.3, 39.9, 12.9; IR (neat): 3309, 2918, 2848, 1629, 1527, 1449, 1341, 1150, 764, 738, 696; MS (ESI, m/z) 467 (M + Na^+); HRESIMS Calcd for $[C_{26}H_{24}N_2NaO_3S]^+$ (M + Na^+) 467.1400, found: 467.1406.

methyl 5-(N-benzylmethylsulfonamido)-2,4-diphenyl-1H-pyrrole-3-carboxylate (3x)

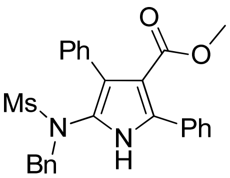

3x

Compound **3x** was prepared in 90% yield by the reaction of isoxazole **2k** with ynamide **1i** according to the general procedure (Table 3, entry 10). ^{1}H NMR (400 MHz, $CDCl_3$) δ 8.38 (s, 1H), 7.43 – 7.26 (m, 15H), 4.56 (s, 2H), 3.45 (s, 3H), 2.60 (s, 3H); ^{13}C NMR (100 MHz, $CDCl_3$) δ 165.0, 136.2, 135.3, 133.9, 131.3, 129.8, 128.9, 128.8, 128.3, 128.2, 128.0, 127.3, 123.8, 123.1, 110.7, 55.5, 50.7, 40.0; IR (neat): 3302, 1709, 1606, 1495, 1455, 1341, 807, 761, 698; MS (ESI, m/z) 483 (M + Na^+); HRESIMS Calcd for $[C_{26}H_{24}N_2NaO_4S]^+$ (M + Na^+) 483.1349, found: 483.1355.

N-benzyl-N-(4-formyl-3,5-diphenyl-1H-pyrrol-2-yl)methanesulfonamide (3y)

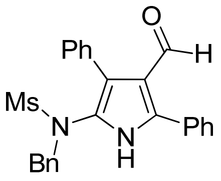

3y

Compound **3y** was prepared in 62% yield by the reaction of isoxazole **2l** with ynamide **1i** according to the general procedure (Table 3, entry 11). ^{1}H NMR (400 MHz, $CDCl_3$) δ 9.74 (s, 1H), 8.36 (s, 1H), 7.48 – 7.29 (m, 15H), 4.63 (s, 2H), 2.68 (s, 3H); ^{13}C NMR (100 MHz, $CDCl_3$) δ 186.2, 139.0, 136.3, 132.3, 130.0, 129.5, 129.3, 129.1, 129.0, 128.9, 128.7, 128.5, 128.5, 128.0, 124.0, 123.6, 118.5, 55.6, 40.1; IR (neat): 3434, 2919, 2849, 1641, 1345, 1150, 764, 697; MS (ESI, m/z) 453 (M + Na^+); HRESIMS Calcd for $[C_{25}H_{22}N_2NaO_3S]^+$ (M + Na^+) 453.1243, found: 453.1253.

N-benzyl-N-(4-formyl-3-phenyl-1H-pyrrol-2-yl)methanesulfonamide (3z)

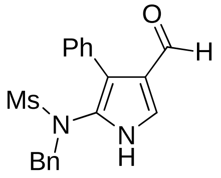

3z

Compound **3z** was prepared in 60% yield by the reaction of isoxazole **2m** with ynamide **1i** according to the general procedure (Table 3, entry 12). ^{1}H NMR (400 MHz, $CDCl_3$) δ 9.63 (s, 1H), 8.78 (s, 1H), 7.66 – 7.08 (m, 11H), 4.59 (s, 2H), 2.69 (s, 3H); ^{13}C NMR (100 MHz, $CDCl_3$) δ 186.1, 136.0, 131.7, 129.9, 128.9, 128.7, 128.4, 128.0, 124.2, 123.5, 123.3, 122.7, 55.4, 40.1; IR (neat): 3396, 2918, 2849, 1657,

1342, 1151, 796, 762, 699; MS (ESI, m/z) 377 (M + Na^+); HRESIMS Calcd for $[C_{19}H_{18}N_2NaO_3S]^+$ (M + Na^+) 377.0930, found: 377.0932.

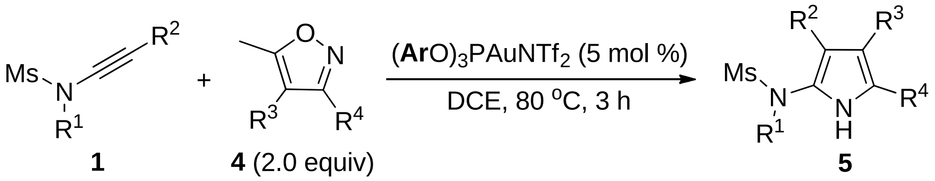

General procedure for the synthesis of 5:

Isoxazole **4** (0.60 mmol) and (**Ar**O)$_3$PAuNTf$_2$ (13.5 mg, 0.015 mmol) were added to a suspension of the ynamide **1** (0.30 mmol) in DCE (6.0 mL) at room temperature. The reaction mixture was then stirred at 80 °C and the progress of the reaction was monitored by TLC. The reaction typically took 3 h. Upon completion, the mixture was concentrated and the residue was purified by chromatography on silica gel (eluent: hexanes/ethyl acetate) to afford the desired product **5**.

N-benzyl-N-(5-methyl-3,4-diphenyl-1H-pyrrol-2-yl)methanesulfonamide (5a)

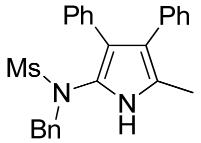

5a

Compound **5a** was prepared in 78% yield by the reaction of isoxazole **4a** with ynamide **1i** according to the general procedure (Table 4, entry 1). ^{1}H NMR (400 MHz, $CDCl_3$) δ 7.72 (s, 1H), 7.35 – 7.02 (m, 15H), 4.70 (s, 2H), 2.59 (s, 3H), 2.12 (s, 3H); ^{13}C NMR (100 MHz, $CDCl_3$) δ 137.0, 135.1, 134.6, 130.0, 129.8, 129.0, 128.7, 128.3, 128.1, 127.8, 126.5, 125.4, 124.0, 120.5, 120.4, 120.2, 56.1, 39.8, 11.9; IR (neat): 3372, 2921, 1641, 1327, 1146, 759, 700; MS (ESI, m/z) 439 (M + Na^+); HRESIMS Calcd for $[C_{25}H_{24}N_2NaO_2S]^+$ (M + Na^+) 439.1451, found: 439.1438.

N-(5-methyl-3,4-diphenyl-1H-pyrrol-2-yl)-N-phenylmethanesulfonamide (5b)

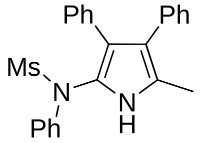

5b

Compound **5b** was prepared in 72% yield by the reaction of isoxazole **4a** with ynamide **1a** according to the general procedure (Table 4, entry 2). ^1H NMR (400 MHz, $CDCl_3$) δ 8.48 (s, 1H), 7.36 – 7.11 (m, 11H), 7.07 – 7.05 (m, 2H), 6.96 – 6.91 (m, 2H), 2.71 (s, 3H), 2.34 (s, 3H); ^{13}C NMR (100 MHz, $CDCl_3$) δ 142.3, 135.0, 134.2, 130.0, 129.2, 128.0, 127.9, 126.4, 126.0, 125.5, 124.6, 124.2, 121.4, 120.9, 120.8, 40.3, 12.1; IR (neat): 3359, 2921, 1640, 1602, 1492, 1342, 1153, 968, 770, 730, 700; MS (ESI, m/z) 425 (M + Na^+); HRESIMS Calcd for $[C_{24}H_{22}N_2NaO_2S]^+$ (M + Na^+) 425.1294, found: 425.1296.

N-butyl-N-(5-methyl-3,4-diphenyl-1H-pyrrol-2-yl)methanesulfonamide (5c)

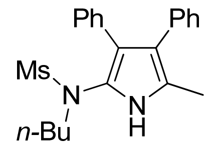

5c

Compound **5c** was prepared in 85% yield by the reaction of isoxazole **4a** with ynamide **1h** according to the general procedure (Table 4, entry 3). ^1H NMR (400 MHz, $CDCl_3$) δ 8.42 (s, 1H), 7.25 – 7.10 (m, 8H), 7.06 – 7.04 (m, 2H), 3.50 (t, 2H, J = 7.6 Hz), 2.58 (s, 3H), 2.26 (s, 3H), 1.66 – 1.58 (m, 2H), 1.31 – 1.26 (m, 2H), 0.90 (t, 3H, J = 7.6 Hz); ^{13}C NMR (100 MHz, $CDCl_3$) δ 135.2, 134.7, 130.0, 129.9, 128.1, 127.8, 126.4, 125.4, 123.9, 120.7, 120.5, 120.4, 52.3, 39.1, 31.7, 19.7, 13.7, 11.9; IR (neat): 3352, 2958, 2871, 1604, 1500, 1329, 1150, 967, 770, 700; MS (ESI, m/z) 405 (M + Na^+); HRESIMS Calcd for $[C_{22}H_{26}N_2NaO_2S]^+$ (M + Na^+) 405.1607, found: 405.1611.

(E)-N-benzyl-N-(5-methyl-4-phenyl-3-styryl-1H-pyrrol-2-yl)methanesulfonamide (5d)

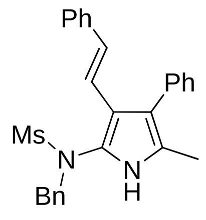

5d

Compound **5d** was prepared in 73% yield by the reaction of isoxazole **4a** with ynamide **1m** according to the general procedure (Table 4, entry 4). ^1H NMR (400 MHz, $CDCl_3$) δ 7.86 (s, 1H), 7.41 – 7.18 (m, 15H), 6.60 (dd, 2H, J = 1.6 Hz, J = 11.2 Hz), 4.84 (s, 2H), 3.03 (s, 3H), 2.06 (s, 3H); ^{13}C NMR (100 MHz, $CDCl_3$) δ 138.0, 136.38, 135.4, 130.5, 129.4, 128.7, 128.5, 128.2, 127.8, 127.0, 126.3, 125.7,

124.8, 121.0, 120.3, 119.7, 116.2, 45.1, 40.1, 11.5; IR (neat): 3444, 2923, 1635, 1495, 1455, 1323, 1151, 753, 700; MS (ESI, m/z) 465 (M + Na^+); HRESIMS Calcd for $[C_{27}H_{26}N_2NaO_2S]^+$ (M + Na^+) 465.1607, found: 465.1613.

N-benzyl-N-(4-(4-fluorophenyl)-5-methyl-3-phenyl-1H-pyrrol-2-yl)methanesulfonamide (5e)

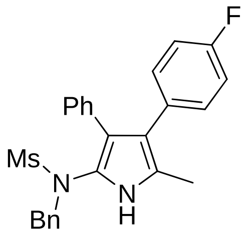

5e

Compound **5e** was prepared in 78% yield by the reaction of isoxazole **4b** with ynamide **1i** according to the general procedure (Table 4, entry 5). ^{1}H NMR (400 MHz, $CDCl_3$) δ 7.82 (s, 1H), 7.39 – 7.15 (m, 8H), 7.09 – 7.03 (m, 2H), 7.02 – 6.94 (m, 2H), 6.93 – 6.84 (m, 2H), 4.68 (s, 2H), 2.59 (s, 3H), 2.10 (s, 3H); ^{13}C NMR (100 MHz, $CDCl_3$) δ 161.0 (d, J = 233.0 Hz), 137.0, 134.5, 131.4 (d, J = 7.7 Hz), 131.1 (d, J = 3.2 Hz), 129.8, 129.0, 128.7, 128.3, 128.1, 126.6, 123.9, 120.6, 120.5, 119.3, 114.7 (d, J = 21.0 Hz), 56.0, 39.8, 11.8; IR (neat): 3356, 2916, 2849, 1711, 1508, 1334, 1265, 1151, 838, 740, 701; MS (ESI, m/z) 457 (M + Na^+); HRESIMS Calcd for $[C_{25}H_{23}FN_2NaO_2S]^+$ (M + Na^+) 457.1356, found: 457.1352.

N-benzyl-N-(4-(4-chlorophenyl)-5-methyl-3-phenyl-1H-pyrrol-2-yl)methanesulfonamide (5f)

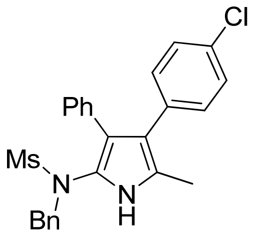

5f

Compound **5f** was prepared in 72% yield by the reaction of isoxazole **4c** with ynamide **1i** according to the general procedure (Table 4, entry 6). ^{1}H NMR (400 MHz, $CDCl_3$) δ 7.96 (s, 1H), 7.32 – 7.15 (m, 8H), 7.13 – 7.07 (m, 2H), 7.07 – 7.05 (m, 2H), 6.94 – 6.92 (m, 2H), 4.68 (s, 2H), 2.58 (s, 3H), 2.08 (s, 3H); ^{13}C NMR (100 MHz, $CDCl_3$) δ 136.8, 134.3, 133.6, 131.2, 129.7, 1128.9, 128.6, 128.4, 128.1, 128.0, 126.6, 124.1, 120.6, 120.4, 119.0, 56.0, 40.0, 11.7; IR (neat): 3357, 2924, 1638, 1496, 1331,

1149, 833, 764 , 699; MS (ESI, m/z) 473 (M + Na+); HRESIMS Calcd for $[C_{25}H_{23}ClN_2NaO_2S]^+$ (M + Na+) 473.1061, found: 473.1063.

N-benzyl-N-(4-(4-bromophenyl)-5-methyl-3-phenyl-1H-pyrrol-2-yl)methanesulfonamide (5g)

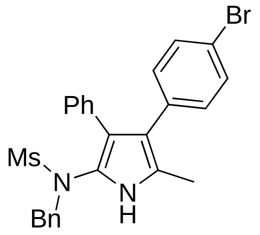

5g

Compound **5g** was prepared in 81% yield by the reaction of isoxazole **4d** with ynamide **1i** according to the general procedure (Table 4, entry 7). ^{1}H NMR (400 MHz, $CDCl_3$) δ 7.92 (s, 1H), 7.39 – 7.18 (m, 10H), 7.06 (d, 2H, *J* = 6.8 Hz), 6.88 (d, 2H, *J* = 8.4 Hz), 4.67 (s, 2H), 2.58 (s, 3H), 2.10 (s, 3H); ^{13}C NMR (100 MHz, $CDCl_3$) δ 136.9, 134.3, 134.2, 131.6, 131.0, 129.8, 129.0, 128.7, 128.4, 128.1, 126.7, 124.1, 120.67, 120.5, 119.4, 119.1, 56.0, 39.8, 11.8; IR (neat): 3352, 2922, 2851, 1603, 1495, 1331, 1149, 764, 699; MS (ESI, m/z) 517 (M + Na+); HRESIMS Calcd for $[C_{25}H_{23}BrN_2NaO_2S]^+$ (M + Na+) 517.0556, found: 517.0559.

methyl 4-(5-(N-benzylmethylsulfonamido)-2-methyl-4-phenyl-1H-pyrrol-3-yl)benzoate (5h)

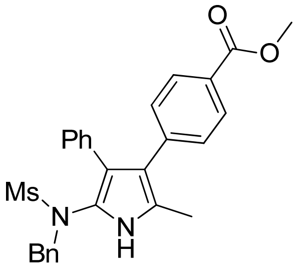

5h

Compound **5h** was prepared in 92% yield by the reaction of isoxazole **4e** with ynamide **1i** according to the general procedure (Table 4, entry 8). ^{1}H NMR (400 MHz, $CDCl_3$) δ 8.15 (s, 1H), 7.86 – 7.84 (m, 2H), 7.32 – 7.21 (m, 8H), 7.08 – 7.05 (m, 4H), 4.68 (s, 2H), 3.86 (s, 3H), 2.59 (s, 3H), 2.12 (s, 3H); ^{13}C NMR (100 MHz, $CDCl_3$) δ 167.2, 140.3, 136.7, 134.2, 129.8, 129.6, 129.1, 128.9, 128.6, 128.4, 128.1, 126.8, 126.7, 124.8, 120.9, 120.6, 119.2, 56.0, 51.9, 39.9, 11.9; IR (neat): 3347, 2926, 1716(s), 1607,

1436, 1333, 1278, 1150, 776, 735, 702; MS (ESI, m/z) 497 (M + Na^+); HRESIMS Calcd for $[C_{27}H_{26}N_2NaO_4S]^+$ (M + Na^+) 497.1505, found: 497.1515.

N-benzyl-N-(5-methyl-3-phenyl-4-(p-tolyl)-1H-pyrrol-2-yl)methanesulfonamide (5i)

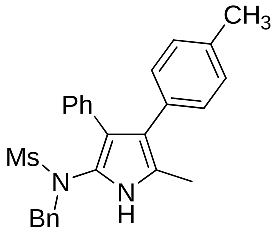

5i

Compound **5i** was prepared in 95% yield by the reaction of isoxazole **4f** with ynamide **1i** according to the general procedure (Table 4, entry 9). ^{1}H NMR (400 MHz, $CDCl_3$) δ 7.86 (s, 1H), 7.32 – 7.18 (m, 8H), 7.10 – 7.08 (m, 2H), 7.00 – 7.98 (m, 2H), 6.92 – 6.90 (m, 2H), 4.69 (s, 2H), 2.57 (s, 3H), 2.28 (s, 3H), 2.09 (s, 3H); ^{13}C NMR (100 MHz, $CDCl_3$) δ 137.0, 134.8, 134.7, 132.1, 129.8, 129.8, 129.0, 128.6, 128.5, 128.0, 126.3, 123.8, 120.5, 120.3, 120.0, 56.1, 39.8, 21.0, 11.8; IR (neat): 3365, 2918, 1657, 1631, 1510, 1453, 1330, 1151, 823, 764, 699; MS (ESI, m/z) 453 (M + Na^+); HRESIMS Calcd for $[C_{26}H_{26}N_2NaO_2S]^+$ (M + Na^+) 453.1607, found: 453.1603.

N-benzyl-N-(4-(4-methoxyphenyl)-5-methyl-3-phenyl-1H-pyrrol-2-yl)methanesulfonamide (5j)

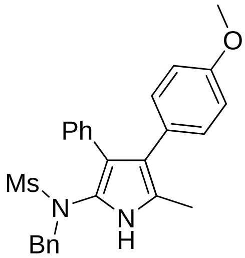

5j

Compound **5j** was prepared in 87% yield by the reaction of isoxazole **4g** with ynamide **1i** according to the general procedure (Table 4, entry 10). ^{1}H NMR (400 MHz, $CDCl_3$) δ 7.85 (s, 1H), 7.33 – 7.18 (m, 8H), 7.10 – 7.07 (m, 2H), 6.95 – 6.93 (m, 2H), 6.75 – 6.73 (m, 2H), 4.69 (s, 2H), 3.74 (s, 3H), 2.57 (s, 3H), 2.08 (s, 3H); ^{13}C NMR (100 MHz, $CDCl_3$) δ 157.4, 137.0, 134.7, 131.0, 129.8, 128.9, 128.6, 128.2, 128.0, 127.6, 126.3, 123.7, 120.4, 120.2, 119.7, 113.3, 56.1, 55.0, 39.8, 11.7; IR (neat): 3357, 2918,

1658, 1510, 1331, 1244, 1148, 834, 764, 700; MS (ESI, m/z) 469 (M + Na^+); HRESIMS Calcd for $[C_{26}H_{26}N_2NaO_3S]^+$ (M + Na^+) 469.1556, found: 469.1558.

N-benzyl-N-(4-(3,4-dimethoxyphenyl)-5-methyl-3-phenyl-1H-pyrrol-2-yl)methanesulfonamide (5k)

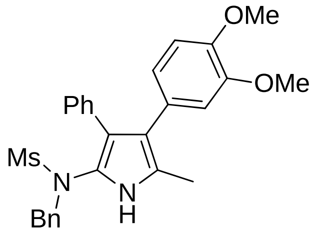

5k

Compound **5k** was prepared in 78% yield by the reaction of isoxazole **4h** with ynamide **1i** according to the general procedure (Table 4, entry 11). ^{1}H NMR (400 MHz, $CDCl_3$) δ 8.01 (s, 1H), 7.43 – 7.17 (m, 8H), 7.10 (d, 2H, J = 7.2 Hz), 6.74 (d, 1H, J = 8.4 Hz), 6.64 (dd, 1H, 2.0 Hz, J = 8.4 Hz), 6.46 (d, 1H, J = 2.0 Hz), 4.69 (s, 2H), 3.83 (s, 3H), 3.53 (s, 3H), 2.60 (s, 3H), 2.14 (s, 3H); ^{13}C NMR (100 MHz, $CDCl_3$) δ 148.0, 146.7, 136.9, 134.8, 129.9, 128.9, 128.7, 128.6, 128.2, 128.0, 127.9, 126.4, 123.5, 121.8, 120.5, 120.2, 119.9, 113.6, 110.7, 56.0, 55.6, 55.3, 39.8, 11.9; IR (neat): 3348, 2926, 1604, 1513, 1451, 1327, 1149, 960, 761, 700; MS (ESI, m/z) 499 (M + Na^+); HRESIMS Calcd for $[C_{27}H_{28}N_2NaO_4S]^+$ (M + Na^+) 499.1662, found: 499.1662.

N-benzyl-N-(4,5-dimethyl-3-phenyl-1H-pyrrol-2-yl)methanesulfonamide (5l)

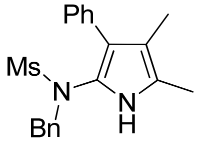

5l

Compound **5l** was prepared in 83% yield by the reaction of isoxazole **4i** with ynamide **1i** according to the general procedure (Table 4, entry 12). ^{1}H NMR (400 MHz, $CDCl_3$) δ 7.53 (s, 1H), 7.42 – 7.36 (m, 2H), 7.35 – 7.21 (m, 8H), 4.63 (s, 2H), 2.59 (s, 3H), 2.03 (s, 3H), 1.89 (s, 3H); ^{13}C NMR (100 MHz, $CDCl_3$) δ 137.2, 135.2, 129.6, 128.9, 128.6, 128.5, 128.0, 126.6, 123.1, 121.4, 119.4, 113.0, 55.9, 39.7, 11.1, 9.7; IR (neat): 3358, 2919, 2849, 1658, 1345, 1150, 700; MS (ESI, m/z) 377 (M + Na^+); HRESIMS Calcd for $[C_{20}H_{22}N_2NaO_2S]^+$ (M + Na^+) 377.1294, found: 377.1297.

N-(3-acetyl-2,3-dimethyl-4-phenyl-3H-pyrrol-5-yl)-N-benzylmethanesulfonamide (5l’)

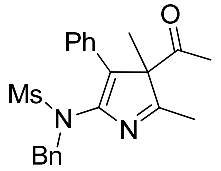

5l'

^{1}H NMR (400 MHz, $CDCl_3$) δ 7.49 – 7.43 (m, 2H), 7.42 – 7.37 (m, 1H), 7.37 – 7.33 (m, 2H), 7.29 – 7.24 (m, 3H), 7.13 – 7.08 (m, 2H), 4.54 (d, 1H, J = 15.2 Hz), 4.27 (d, 1H, J = 15.2 Hz), 3.02 (s, 3H), 1.91 (s, 3H), 1.86 (s, 3H), 1.48 (s, 3H); ^{13}C NMR (100 MHz, $CDCl_3$) δ 203.9, 167.6, 162.0, 135.2, 134.8, 131.6, 128.8, 128.7, 128.6, 128.5, 128.3, 128.2, 88.1, 52.1, 42.6, 24.9, 18.3, 11.7.; IR (neat): 2928, 1714, 1347, 1152, 773, 701; MS (ESI, m/z) 419 (M + Na^+); HRESIMS Calcd for $[C_{22}H_{24}N_2NaO_3S]^+$ (M + Na^+) 419.1400, found: 419.1402.

N-benzyl-N-(4-decanoyl-5-methyl-3-phenyl-1H-pyrrol-2-yl)methanesulfonamide (5m)

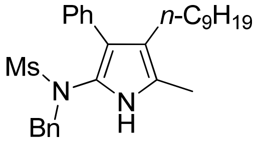

5m

Compound **5m** was prepared in 78% yield by the reaction of isoxazole **4j** with ynamide **1i** according to the general procedure (Table 4, entry 13). ^{1}H NMR (400 MHz, $CDCl_3$) δ 7.60 (s, 1H), 7.38 – 7.25 (m, 8H), 7.21 – 7.19 (m, 2H), 4.59 (s, 2H), 2.59 (s, 3H), 2.27 (t, 2H, J = 7.6 Hz), 2.03 (s, 3H), 1.28 – 1.14 (m, 14H), 0.87 (t, 3H, J = 6.8 Hz); ^{13}C NMR (100 MHz, $CDCl_3$) δ 137.1, 135.5, 129.8, 128.8, 128.5, 128.3, 127.9, 126.6, 122.7, 121.2, 119.2, 118.5, 55.8, 39.5, 31.8, 31.0, 29.5, 29.2, 24.2, 22.6, 14.1, 11.1; IR (neat): 3368, 2924, 2853, 1641, 1605, 1454, 1332, 1150, 799, 761, 700; MS (ESI, m/z) 489 (M + Na^+); HRESIMS Calcd for $[C_{28}H_{38}N_2NaO_2S]^+$ (M + Na^+) 489.2546, found: 489.2550.

methyl 5-(N-benzylmethylsulfonamido)-2-methyl-4-phenyl-1H-pyrrole-3-carboxylate (5n)

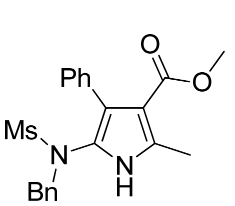

5n

Compound **5n** was prepared in 77% yield by the reaction of isoxazole **4k** with ynamide **1i** according to the general procedure (Table 4, entry 14). ^{1}H NMR (400 MHz, $CDCl_3$) δ 8.28 (s, 1H), 7.39 – 7.23 (m, 10H), 4.52 (s, 2H), 3.56 (s, 3H), 2.60 (s, 3H), 2.31 (s, 3H); ^{13}C NMR (100 MHz, $CDCl_3$) δ 165.3, 136.2, 134.4, 134.3, 129.8, 128.8, 128.7, 128.14, 127.8, 127.1, 123.1, 121.1, 110.1, 55.5, 50.5, 39.8, 13.6; IR (neat): 3319, 2924, 1692, 1678, 1606, 1449, 1336, 1150, 761, 699; MS (ESI, m/z) 421 (M + Na^+); HRESIMS Calcd for $[C_{21}H_{22}N_2NaO_4S]^+$ (M + Na^+) 421.1192, found: 421.1195.

N-benzyl-N-(3,4,5-triphenyl-1H-pyrrol-2-yl)methanesulfonamide (5o)

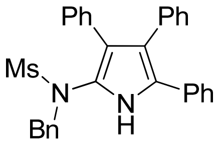

5o

Compound **5o** was prepared in 60% yield by the reaction of isoxazole **4l** with ynamide **1i** according to the general procedure (Table 4, entry 15). ^{1}H NMR (400 MHz, $CDCl_3$) δ 8.19 (s, 1H), 7.32 – 7.00 (m, 20H), 4.66 (s, 2H), 2.72 (s, 3H); ^{13}C NMR (100 MHz, $CDCl_3$) δ 136.7, 134.8, 134.2, 132.1, 130.8, 128.96, 128.7, 128.4, 128.2, 128.1, 127.9, 127.4, 127.3, 126.9, 126.8, 126.0, 122.6, 122.2, 120.9, 55.7, 40.0; IR (neat): 3330, 3060, 2927, 1603, 1587, 1493, 1332, 1149, 910, 792, 763, 698; MS (ESI, m/z) 501 (M + Na^+); HRESIMS Calcd for $[C_{30}H_{26}N_2NaO_2S]^+$ (M + Na^+) 501.1607, found: 501.1601.

5-methyl-1-(methylsulfonyl)-6,7-diphenyl-2,3-dihydro-1H-pyrrolo[1,2-a]imidazole (6)

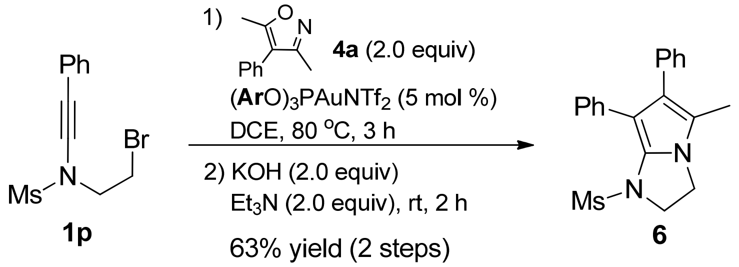

Isoxazole **4a** (103.9 mg, 0.60 mmol) and (**Ar**O)$_3$PAuNTf$_2$ (13.5 mg, 0.015 mmol) were added to a suspension of the ynamide **1p** (90.7 mg, 0.30 mmol) in DCE (6.0 mL) at room temperature. The reaction mixture was then stirred at 80 °C for 3 h. Upon completion, the mixture was treated with KOH (33.6 mg, 0.60 mmol) and Et_3N (83 uL, 0.60 mmol), and stirred at room temperature for another 2 h. The residue was then concentrated and purified by chromatography on silica gel (eluent: hexanes/ethyl acetate) to afford the desired product **6** (63% overall yield, eq 6). ^{1}H NMR (400 MHz, $CDCl_3$) δ 7.30 –

7.03 (m, 10H), 4.48 (t, 2H, J = 6.8 Hz), 3.99 (t, 2H, J = 6.8 Hz), 2.54 (s, 3H), 2.21 (s, 3H); ^{13}C NMR (100 MHz, $CDCl_3$) δ 135.3, 134.0, 130.2, 129.9, 129.2, 128.0, 127.8, 126.0, 125.6, 123.2, 119.7, 110.1, 53.2, 42.6, 38.6, 10.8; IR (neat): 2917, 2849, 1601, 1566, 1408, 1349, 781, 765, 698; MS (ESI, m/z) 375 (M + Na^+); HRESIMS Calcd for $[C_{20}H_{20}N_2NaO_2S]^+$ (M + Na^+) 375.1138, found: 375.1143.

N-benzyl-2-nitro-N-(4-pentanoyl-3,5-diphenyl-1H-pyrrol-2-yl)benzenesulfonamide (7)

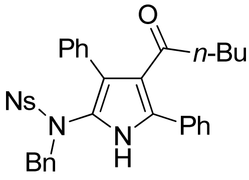

7

Compound **7** was prepared in 81% yield by the reaction of isoxazole **2c** with ynamide **1d** according to the general procedure (Scheme 2). ^{1}H NMR (400 MHz, $CDCl_3$) δ 8.35 (s, 1H), 7.74 – 7.66 (m, 2H), 7.63 – 7.61 (m, 1H), 7.55 (t, 1H, J = 6.0 Hz), 7.37 – 7.30 (m, 10H), 7.19 – 7.16 (m, 1H), 7.11 (t, 2H, J = 6.0 Hz), 6.75 (d, 2H, J = 6.0 Hz), 4.81 (s, 2H), 2.10 (t, 3H, J = 6.0 Hz), 1.36 – 1.30 (m, 2H), 1.02 – 0.94 (m, 2H), 0.66 (t, 3H, J = 6.0 Hz); ^{13}C NMR (100 MHz, $CDCl_3$) δ 200.5, 147.6, 136.4, 134.1, 133.4, 132.9, 132.2, 131.9, 131.4, 129.4, 129.1, 128.9, 128.7, 128.6, 128.5, 128.4, 128.0, 127.9, 127.2, 124.7, 123.7, 121.7, 121.2, 56.9, 42.9, 26.6, 22.0, 13.6; IR (neat): 3356, 2920, 1658, 1632, 1383, 755, 699; MS (ESI, m/z) 616 (M + Na^+); HRESIMS Calcd for $[C_{34}H_{31}N_3NaO_5S]^+$ (M + Na^+) 616.1877, found: 616.1873.

1-(5-(benzylamino)-1-methyl-2,4-diphenyl-1H-pyrrol-3-yl)pentan-1-one (7a)

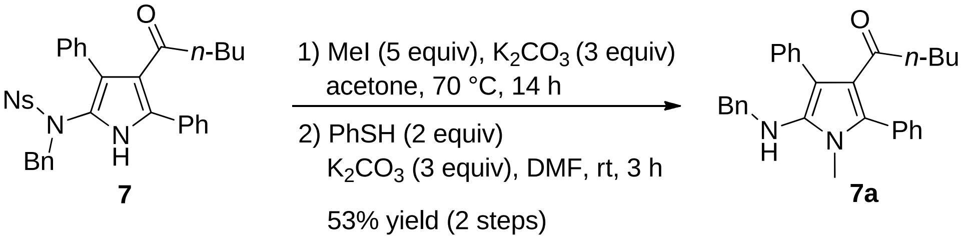

MeI (93.4 uL, 1.50 mmol) and K_2CO_3 (124.2 mg, 0.90 mmol) were added to a suspension of the pyrrole **7** (178.1 mg, 0.30 mmol) in acetone (6.0 mL) at room temperature. The reaction mixture was then stirred at 70 °C for 14 h. Upon completion, the mixture was then concentrated and purified by chromatography on silica gel (eluent: hexanes/ethyl acetate) to afford the desired product **7'**, which was then converted into the compound **7a** according to the known procedure (53% two-step overall yield, Scheme 2).[17] ^{1}H NMR (400 MHz, $CDCl_3$) δ 7.46 – 7.39 (m, 3H), 7.38 – 7.33 (m, 4H), 7.29 – 7.23 (m,

7H), 7.17 – 7.14 (m, 2H), 3.92 (s, 2H), 3.31 (s, 3H), 2.09 (t, 2H, *J* = 7.6 Hz), 1.39 – 1.31 (m, 2H), 1.03 – 0.94 (m, 2H), 0.65 (t, 3H, *J* = 7.2 Hz); ^{13}C NMR (100 MHz, $CDCl_3$) δ 199.3, 138.9, 135.9, 135.5, 132.8, 132.7, 130.6, 130.0, 128.4, 128.3, 128.2(4), 128.1(9), 128.1, 127.4, 126.4, 121.4, 114.7, 54.4, 42.3, 30.8, 26.8, 22.2, 13.7; IR (neat): 2957, 2929, 1660, 1543, 1366, 1167, 698; MS (ESI, m/z) 445 (M + Na^+); HRESIMS Calcd for $[C_{29}H_{30}N_2NaO]^+$ (M + Na^+) 445.2250, found: 445.2254.

1-(5-amino-1-methyl-2,4-diphenyl-1H-pyrrol-3-yl)pentan-1-one (7b)

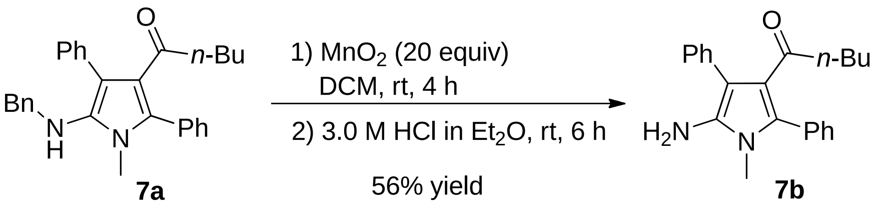

Compound **7b** was prepared in 56% yield according to the known procedure (Scheme 2).[18] ^{1}H NMR (400 MHz, $CDCl_3$) δ 7.44 – 7.33 (m, 7H), 7.30 – 7.21 (m, 3H), 3.28 – 3.26 (m, 5H), 2.11 (t, 2H, *J* = 7.6 Hz), 1.40 – 1.32 (m, 2H), 1.04 – 0.94 (m, 2H), 0.65 (t, 3H, *J* = 7.2 Hz); ^{13}C NMR (100 MHz, $CDCl_3$) δ 199.4, 135.6, 133.9, 132.4, 131.4, 130.8, 129.7, 128.4, 128.3, 128.1, 125.9, 121.2, 107.5, 42.2, 30.2, 26.9, 22.1, 13.6; IR (neat): 3435, 2924, 1687, 1625, 1450 , 698; MS (ESI, m/z) 355 (M + Na^+); HRESIMS Calcd for $[C_{22}H_{24}N_2NaO]^+$ (M + Na^+) 355.1781, found: 355.1782.

(E)-N-((E)-4-oxopent-2-en-2-yl)hex-2-enamide (8)

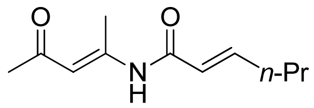

8

Compound **8** was prepared in 25% yield by the reaction of isoxazole **2a** with ynamide **1q** according to the general procedure (eq 7). ^{1}H NMR (400 MHz, $CDCl_3$) δ 12.49 (s, 1H), 6.96 (dt, 1H, *J* = 7.2 Hz, *J* = 15.2 Hz), 5.93 (dt, 1H, *J* = 1.2 Hz, *J* = 15.2 Hz), 5.36 (s, 1H), 2.42 (s, 3H), 2.24 – 2.17 (m, 2H), 2.15 (s, 3H), 1.57 – 1.46 (m, 2H), 0.94 (t, 3H, *J* = 7.2 Hz); ^{13}C NMR (100 MHz, $CDCl_3$) δ 199.6, 165.0, 155.9, 147.9, 124.8, 105.5, 34.2, 30.4, 21.9, 21.3, 13.6; IR (neat): 3361, 2920, 2850, 1704, 1641, 1595, 1478, 1256, 764, 749; MS (ESI, m/z) 218 (M + Na^+); HRESIMS Calcd for $[C_{11}H_{17}NNaO_2]^+$ (M + Na^+) 218.1151, found: 218.1150.

Reference:

1. Yao, B.; Liang, Z.; Niu, T.; Zhang, Y. *J. Org. Chem*. **2009**, *74*, 4630.
2. Zhang, Y.; Hsung, R. P.; Tracey, M. R.; Kurtz,K. C. M.; Vera, E. L.; *Org. Lett.* **2004**, *6*, 1151.
3. Li, L.; Shu, C.; Zhou, B.; Yu, Y.-F.; Xiao, X.-Y.; Ye, L.-W. *Chem. Sci.* **2014**, *5*, DOI: 10.1039/C4SC00983E.
4. Mukherjee, A.; Dateer, R. B.; Chaudhuri, R.; Bhunia, S.; Karad, S. N.; Liu, R.-S. *J. Am. Chem. Soc.* **2011**, *133*, 15372.
5. Davies, P. W.; Cremonesi, A.; Martin, N. *Chem. Commun.* **2011**, 379.
6. (a) Vo, C.-V. T.; Mitchell, T. A.; Bode, J. W. *J. Am. Chem. Soc.* **2011**, *133*, 14082. (b) Kienzler, M. A.; Reiner, A.; Trautman, E.; Yoo, S.; Trauner, D.; Isacoff, E. Y. *J. Am. Chem. Soc.* **2013**, *135*, 17683.
7. Hansen, T. V.; Wu, P.; Fokin, V. V. *J. Org. Chem.* **2005**, *70*, 7761.
8. Debleds, O.; Gayon, E.; Ostaszuk, E.; Vrancken, E.; Campagne, J.-M. *Chem. Eur. J.* **2010***, 16,* 12207.
9. Griesbeck, A. G.; Franke, M.; Neudörfl, J.; Kotaka, H. *Beilstein J. Org. Chem.* **2011***, 7,*127.
10. Tang, S.; He, J. ; Sun, Y. ; He, L. ; She, X. *Org. Lett.* **2009**, *17*, 3982.
11. John, B. A.; Javier, G.; Hui, L.; Angelca, L. M.; Howard, T. J. PCT Int. Appl. WO 2004074270A2, **2004**.
12. Zheng, Y.; Yang, C.; Zhang-Negrerie, D.; Du, Y.; Zhao, K. *Tetrahedron Lett.* **2013**, *54*, 6157.
13. Fall, Y.; Reynaud, C.; Doucet, H.; Santelli, M. *Eur. J. Org. Chem.* **2009**, 4041.
14. Cativiela, C.; Serrano, J. L.; Zurbano, M. M. *J. Org. Chem.* **1995**, *60*, 3074.
15. Lasri, J.; Mukhopadhyay, S.; Charmier, M. A. J.; Charmier, M. A. J. *J. Heterocycl. Chem.* **2008**, *45*, 1385.
16. Reddy, A. R.; Goverdhan, G.; Sampath, A.; Mukkanti, K.; Reddy, P. P.; Bandichhor, R. *Synth. Commun*. **2012**, *42*, 639.
17. Häkkinen, M. R.; Keinänen, T. A.; Khomutov, A. R.; Auriola, S.; Weisell, J.; Alhonen, L.; Jänne, J.; Vepsäläinen, J. *Tetrahedron* **2009**, *65*, 547.
18. Fontaine, P.; Masson, G.; Zhu, J. *Org. Lett.* **2009**, *11*, 1555.

1. Computational Details

All DFT (density functional theory) calculations were carried out using the Gaussian 09 program.[1] All structures were optimized with no constraints of freedom by using the hybrid density functional M06[2] in combination with double-ζ basis set plus polarization (DZP), i.e., the 6-31+G(d) basis set[3] for C, H, N, O, F, S and P and the Stuttgart/Dresden small-core RECP (relativistic effective core potential) plus valence double-ζ basis set (SDD)[4] for Au. DFT calculations at similar levels have been shown to give reasonable predictions on the mechanism and selectivity of some gold-catalyzed reactions.[5] Vibrational analyses at the same level of theory were performed to confirm each stationary point to be either a local minimum or a transition state (TS). The connection of each TS to its corresponding reactant and product was confirmed by IRC (intrinsic reaction coordinate)[6] calculations. Solvent effects were taken into account by the polarizable continuum model (PCM) with 1,2-dichloroethane (ε = 10.12) as solvent.[7] Natural Bond Orbital (NBO)[8] analyses were performed using the NBO 3.1 code[9] as implemented in Gaussian 09 program.

The catalytic species $(ArO)_3PAu^+$ is simplified as H_3PAu^+ in the computations.[10] Reported energies are relative Gibbs free energies in 1,2-dichloroethane (ΔG_{sol}, in kcal/mol) at 298 K.

2. Results and Discussion

Scheme S1

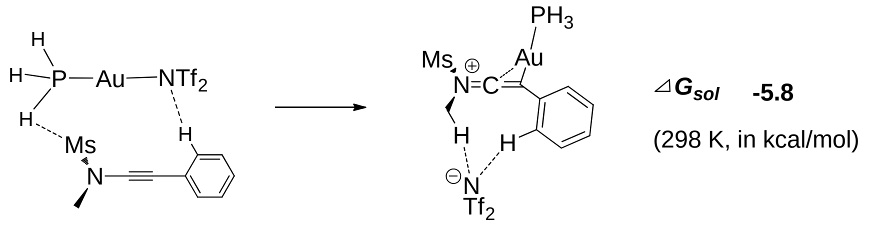

The formation of Au(I)-ligated ynamide **1** upon ligand exchange is predicted to be slightly exothermic, as shown in Scheme S1).

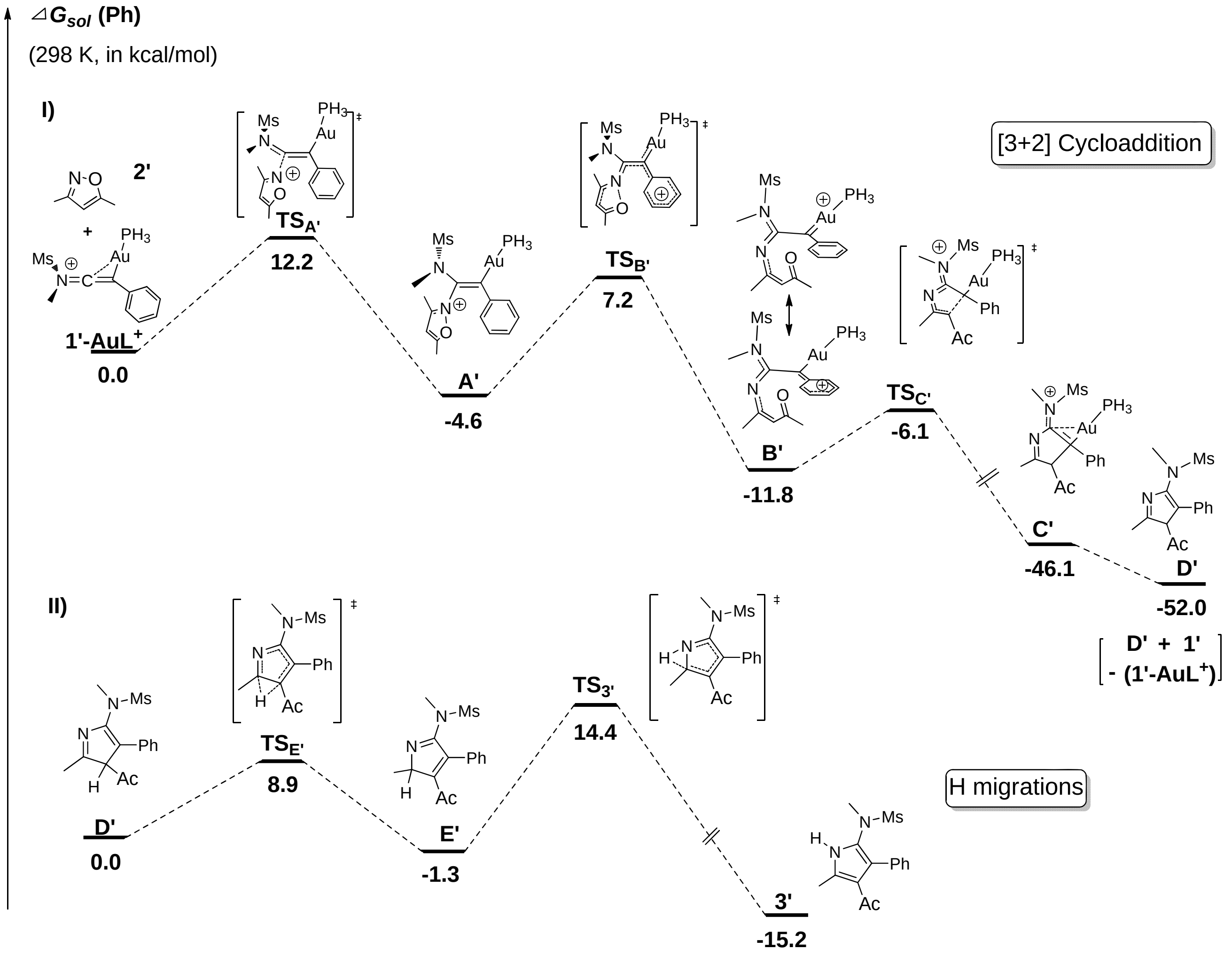

Figure S1. Energy profiles of the Au(I)-catalyzed [3+2] cycloaddition of phenyl ynamide with isoxazole (**I**) and H-migration on pyrrole (**II**).

Phenyl ynamide case. Then density functional theory (DFT) computations studies were performed to provide theoretical support to the proposed mechanism in the main test. The relative energies of all intermediates and transitions states along the reaction pathway are shown in figure S1. Substrate ynamide **1'** interacting with **[Au-PH$_3$]$^+$** generates allene-like organogold complex **[1'-Au-PH$_3$]$^+$**. Then nucleophilic attack of substrate 2' to the central carbon on the **[1'-Au-PH$_3$]$^+$** produce intermediate A' with a activation free energy 12.2 kcal/mol. Next, intermediate **A'** could transform to a more stable intermediate **B'** (Au-carbene) by overcoming an activation barrier 11.8 kcal/mol for breaking the N-O bond. From Au-carbene intermediate **B'**, stable intermediate **C'** containing 3-H pyrrole could be easily

achieved by an intramolecular cyclosation with small activation barrier (5.7 kcal/mol). A subsequent substrate exchanging reaction between **C'** and **1'** could obtain the 3-*H* pyrrole **D'** and regenerate the starting organogold complex **[1'-Au-PH$_3$]$^+$**. Eventually, 3-*H* pyrrole **D'** would transformed into 1-*H* pyrrole **3'** by two steps of H-migrations. The second step H-migration form 2-*H* pyrrole to 1-*H* pyrrole **3'** has a higher activation barrier 15.7 kcal/mol. Consequently, the gold-carbene involved [3+2] catalytic cycle is quite favored in thermodynamics and has no significantly high activation barrier to overcome.

Scheme S2

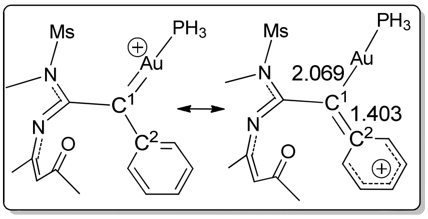

B'(Ph)	*Wiberg Bond index:*	B'(Me)
**0.57**	C^1-Au	**0.62**
**1.39**	C^1-C^2	**1.20**
	Natural Charges:	
**0.35**	Ph/Me	**0.11**

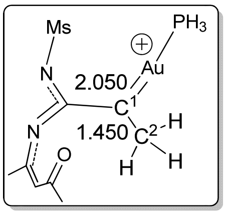

Additionally, we found that the phenyl group in ynamide could help to stabilize the gold-carbene moiety. As resonance structures for **B'** depicted in scheme S2 left, there is conjugation between phenyl group and gold-carbene. This could be proved by the C^1(carbene)-C^2(Ph) bond length, 1.405 Å which is nearly identical to C-C bond length in aryl compounds. NBO analyses shows that the C^1-C^2 bond index is 1.39 and the natural charges on phenyl group is 0.34. For a comparison, we use methyl to replace the phenyl group to study the alkyl gold-carbene (Scheme S2, right). The C1-C2 bond length in **B'** (Me) is 1.450 Å and its bond index is 1.20, which implies hyperconjugation between gold-carbene and C-H bond (Me). The natural charge on methyl group is 0.11. Obviously, the π conjugation of C^1 and Ph in **B'** **(Me)** is stronger than the hyperconjugation C^1 and C-H bond in **B' (Me)**, which results in relatively weaker electrophilicity of phenyl gold-carbene given verse of methyl gold-carbene. This difference as such in electrophilicity of gold-carbene might lead to different chemoselectivity of outputs.

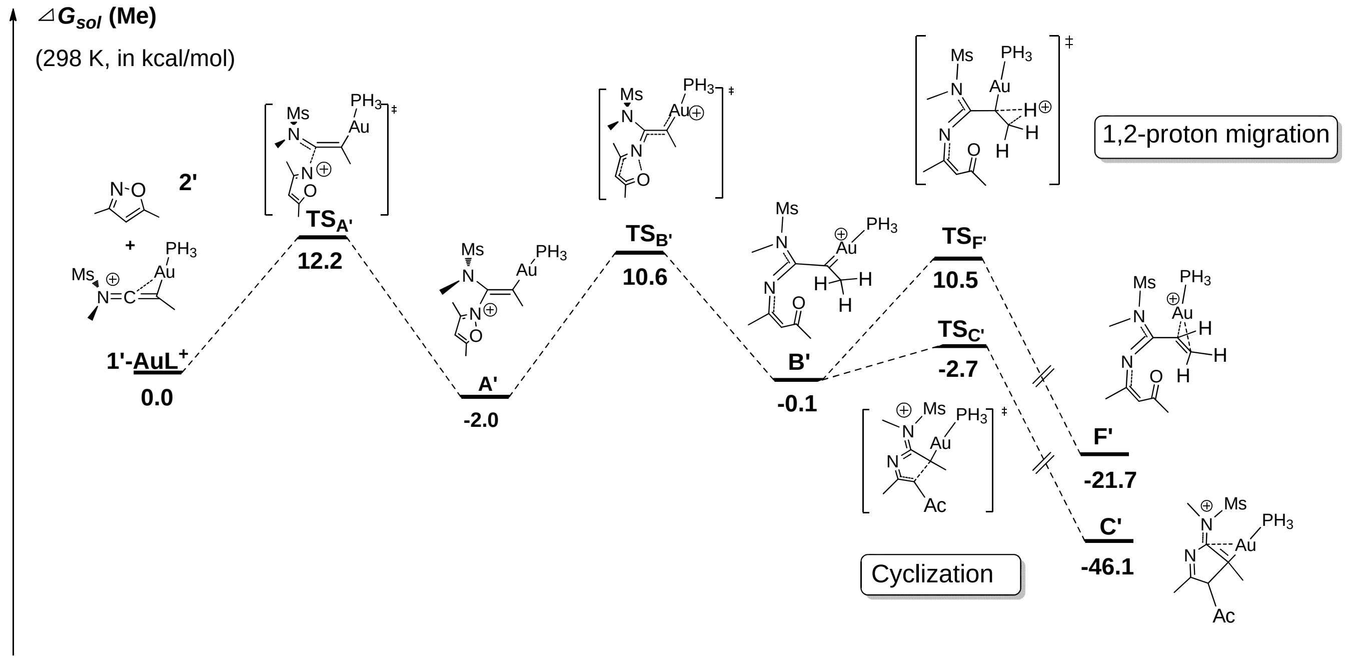

Figure S2. Energy profiles of the Au(I)-catalyzed reaction between methyl ynamide and isoxazole.

Methyl ynamide case. Hence, we also considered the energy diagram of the reaction between methyl ynamide and isoxazole (Figure S2). The formation of methyl gold-carbene needs to overcome an activation barrier 12.6 kcal/mol, slightly harder than the formation of phenyl gold-carbene (11.8 kcal/mol). The activation barrier for pyrrole formation from **B' (Me)** is smaller than it from **B' (Ph)**, 2.6 kcal/mol vs. 5.7 kcal/mol. This is in agreement with the aforementioned difference in electrophilicity of gold-carbene, **B' (Me)** > **B' (Ph)**. On the other hand, the intramolecular 1,2-proton migration of methyl gold carbene was also considered since those migrations were common in gold-carbene involved cycloisomerizations of enynes.[11] Due to the hyperconjugation between gold-carbene and C-H bond, 1,2-proton migration occurs only with a barrier of 10.6 kcal/mol. Though the 1,2-proton migration pathway is harder than the cyclization pathway, it is not the rate-limiting step in the whole pathway and it is quite irreversible. The C-H activation product might still as a minor side product in the final outputs. As such, other more nucleophilic functional groups (e.g. C=N) than C-H bond might be as stronger competitors against the desired cyclosation pathway. In this way, high electrophilicity of gold-carbene means low chemoselectivity of outputs in reaction system with many functional groups. On the other side, phenyl

group of ynamides in the present reaction system not only stabilizes the gold-carbene but also enhance the chemoselectivity of [3+2] cycloaddition products.

References:

1. A. F. Izmaylov, J. Bloino, G. Zheng, J. L. Sonnenberg, M. Hada, M. Ehara, K. Toyota, R. Fukuda, J. Hasegawa, M. Ishida, T. Nakajima, Y. Honda, O. Kitao, H. Nakai, T. Vreven, J. A. Montgomery, Jr., J. E. Peralta, F. Ogliaro, M. Bearpark, J. J. Heyd, E. Brothers, K. N. Kudin, V. N. Staroverov, T. Keith, R. Kobayashi, J. Normand, K. Raghavachari, A. Rendell, J. C. Burant, S. S. Iyengar, J. Tomasi, M. Cossi, N. Rega, J. M. Millam, M. Klene, J. E. Knox, J. B. Cross, V. Bakken, C. Adamo, J. Jaramillo, R. Gomperts, R. E. Stratmann, O. Yazyev, A. J. Austin, R. Cammi, C. Pomelli, J. W. Ochterski, R. L. Martin, K. Morokuma, V. G. Zakrzewski, G. A. Voth, P. Salvador, J. J. Dannenberg, S. Dapprich, A. D. Daniels, O. Farkas, J. B. Foresman, J. V. Ortiz, J. Cioslowski, and D. J. Fox, Gaussian, Inc., Wallingford CT, 2010. *Gaussian09 Rev. B.01,* Gaussian, Inc., Wallingford CT, **2010**.
2. Y. Zhao, D. G. Truhlar, *Acc. Chem. Res.* **2008**, *41*, 157.
3. W. J. Hehre, R. Ditchfield, J. A. Pople, *J. Chem. Phys.* **1972**, *56*, 2257.
4. P. Fuentealba, H. Preuss, H. Stoll, L. von Szentpaly, *Chem. Phys. Lett.* **1989**, *89*, 418.
5. (a) E.L. Noey, Y. Luo, L. Zhang, K. N. Houk, *J. Am. Chem. Soc.* **2012**, *134*, 1078. (b) D. Leboeuf, A. Simonneau, C. Aubert, M. Malacria, V. Gandon, L. Fensterbank, *Angew. Chem. Int. Ed.* **2011**, *50*, 6868. (c) R. Sanz, D. Miguel, M. Gohain, P. García-García, M. A. Fernández-Rodríguez, A. González-Pérez, O. Nieto-Faza, Á. R. de Lera, F. Rodríguez, *Chem. Eur. J.* **2010**, *16*, 9818. (d) A. S. Dudnik, Y. Xia, Y. Li, V. Gevorgyan, *J. Am. Chem. Soc.* **2010**, *132*, 7645. (e) Y. Xia, A. S. Dudnik, Y. Li, V. Gevorgyan, *Org. Lett.*, **2010**, *12*, 5538. (f) E. Seraya, E. Slack, A. Ariafard, B. F. Yates, C. J. T. Hyland, *Org. Lett.*, **2010**, *12*, 4768. (g) Y. Xia, G. Huang, *J. Org. Chem.*, **2010**, *75*, 7842. (h) G. Kovács, A. Lledós, G. Ujaque, *Organometallics* **2010**, *29*, 3252. (i) R. S. Paton, F. Maseras, *Org. Lett.*, **2009**, *11*, 2237. (j) G. Kovács, G. Ujaque, A. Lledós, *J. Am. Chem. Soc.* **2008**, *130*, 853. (k) P.

H. Cheong, P. Morganelli, M. R. Luzung, K. N. Houk, F. D. Toste, *J. Am. Chem. Soc.* **2008**, *130*, 4517.

6. C. Gonzalez, H. B. Schlegel, *J. Chem. Phys.* **1989**, *90*, 2154.

7. (a) M. T. Cancès, B. Mennucci, J. Tomasi, *J. Chem. Phys.*, **1997**, *107*, 3032. (b) M. Cossi, G. Scalmani, N. Rega, V. Barone, *J. Chem. Phys.*, **2002**, *117*, 43.

8. A. E. Reed, L. A. Curtis, F. Weinhold, *Chem. Rev.* **1988**, *88*, 899.

9. NBO Version 3.1, E. D. Glendening, A. E. Reed, J. E. Carpenter, and F. Weinhold.

10. (a) B. Lu, Y. Li, Y. Wang, D. H. Aue, Y. Luo, L. Zhang, *J. Am. Chem. Soc.* **2013**, *135*, 8512. (b) Y. Wang, A. Yepremyan, S. Ghorai, R. Todd, D. H. Aue, L. Zhang, *Angew. Chem. Int. Ed.* **2013**, *52*, 7795.

11. (a) E. Soriano J. Marco-Contelles, *J. Org. Chem.* **2012**, *77*, 6231. (b) N. Cabello, E. Jiménez-Núñez, E. Buñuel, D. J. Cárdenas, A. M. Echavarren, *Eur. J. Org. Chem.* **2007**, 4217. (c) E. Jiménez-Núñez, A. M. Echavarren, *Chem. Rev.* **2008**, *108*, 3326.

Cartesian Coordinates, Total Electronic Energies (E_{total}, in Hartree)

Scheme S1

yne-LAuX E_{total} = -3296.24003

C	-1.83071300	-0.45954500	0.24742700
C	-1.51347100	0.64826600	-0.30359400
N	-1.32625100	1.81471400	-0.80496400
S	-1.66460900	3.22700500	0.23247700
C	-0.18528100	3.31291700	1.20204800
H	0.66071100	3.42742200	0.51780100
H	-0.28789200	4.18005100	1.86243800
H	-0.10309400	2.38644500	1.77994700
C	-0.81810300	2.07524100	-2.16902800
H	0.13795600	2.60287800	-2.10296200
H	-0.66568600	1.10480900	-2.64608900
H	-1.55148000	2.66643300	-2.72248700
O	-1.74829200	4.34382700	-0.69204600
O	-2.79144700	2.84172700	1.06322300
C	-1.07646400	-1.44665600	1.01189300
C	0.31388900	-1.30428200	1.15039900
C	-1.71656000	-2.54379800	1.60338800
C	1.04682800	-2.24629300	1.85989000
H	0.82311100	-0.46050100	0.68236500
C	-0.98131700	-3.47720200	2.32662800
H	-2.79533100	-2.65894200	1.49443400
C	0.39930700	-3.33141600	2.45332700
H	2.12806300	-2.13493800	1.93818500
H	-1.48712500	-4.32359600	2.78801900
H	0.97461700	-4.06877600	3.01088800
Au	-3.90380900	-0.65116900	-0.23142900
P	-6.18624000	-0.95286400	-0.67317900
H	-7.03507300	0.12234700	-0.37730800
H	-6.54944900	-1.23784200	-1.99632800
H	-6.81612800	-1.99821600	0.01547300
N	3.28511800	-0.30429200	0.18653200
S	3.15360200	1.16789100	-0.42384800
S	4.56208600	-1.26027000	0.00175600
O	1.72925500	1.48821200	-0.52428200
O	4.02624600	1.51475200	-1.53886100
O	5.81270400	-0.61404700	-0.37801600
O	4.56370900	-2.22824500	1.09252500
C	4.07980000	-2.26405000	-1.48323800
F	3.92668900	-1.47679800	-2.54183300
F	5.03694400	-3.14983500	-1.73732200
F	2.94283100	-2.91042200	-1.25816000
C	3.73677400	2.22793200	0.98602800

F	3.64145300	3.50888500	0.63966200
F	4.99806800	1.95043600	1.28157000
F	2.98361800	2.02042800	2.06138400

$[LAu\text{-}yne]^{+}X^{-}$ E_{total} = -3296.24300

Au	1.86916200	1.16939100	-0.35265300
P	2.08418100	3.45324100	-0.49429600
S	2.94200000	-1.93528500	-0.18613600
O	3.97916200	-1.22413000	-0.91392900
O	2.64551300	-3.32718700	-0.46304100
C	3.50471600	-1.88979700	1.59139700
F	4.59510900	-2.63532000	1.69002000
F	2.56668000	-2.37130600	2.38831100
F	3.78939200	-0.64647400	1.94877700
N	1.60512900	-0.96625500	-0.17282600
S	0.06831300	-1.52884100	0.04357700
O	0.00073800	-2.82937600	0.68151600
O	-0.72473200	-0.41175700	0.52990800
C	-0.53521600	-1.80531300	-1.69812400
F	0.19273400	-2.73311100	-2.29436700
F	-1.79519000	-2.20509000	-1.62905800
F	-0.46786700	-0.67862300	-2.39202500
H	3.04240300	4.04321800	0.33885800
H	2.42106700	3.97254100	-1.75055000
H	0.91660700	4.16641500	-0.17228500
H	-3.06447500	-1.87286700	1.14157100
C	-4.09522100	-2.01587700	0.81520000
C	-4.74864300	-0.96770100	0.14530800
C	-4.75520100	-3.21656600	1.04950200
C	-4.05687400	0.25928800	-0.09231600
C	-6.07392200	-1.14623000	-0.28076800
C	-6.07068900	-3.38691100	0.61728200
H	-4.24124100	-4.02434600	1.56810100
C	-3.39074900	1.26049700	-0.26278100
C	-6.72733000	-2.35161000	-0.04752700
H	-6.58029800	-0.33210400	-0.79768500
H	-6.58465400	-4.32973700	0.79847100
N	-2.64378800	2.36596400	-0.46736700
H	-7.75402200	-2.48401400	-0.38510700
S	-2.30916900	3.30923000	0.91075500
C	-1.65902500	2.34238400	-1.55996800
C	-1.39511000	2.24332700	2.00091700
O	-1.43517700	4.38522100	0.44912900
O	-3.58817100	3.62138300	1.53293400
H	-1.33982500	3.36328900	-1.78134800
H	-0.79550300	1.71154500	-1.30511200

H	-2.15977800	1.92527400	-2.43840000
H	-0.49419700	1.89468800	1.48436700
H	-1.14046500	2.83723200	2.88453100
H	-2.02659500	1.39194900	2.27300900

Figure S1 (Ph)

1' E_{total} = -990.53094

C	0.77887100	0.33939600	-0.29425500
C	-0.41682600	0.48262100	-0.45693000
N	-1.74276100	0.64390900	-0.64512700
S	-2.75091400	-0.51812500	0.09720600
C	-2.68282900	-0.12202500	1.82962900
H	-3.10359500	0.87755800	1.97546000
H	-3.28100800	-0.86905800	2.36030300
H	-1.63819700	-0.16593000	2.15396400
C	-2.25601900	2.01793300	-0.77806300
H	-3.31716600	1.97605500	-1.03439900
H	-2.10912000	2.59220500	0.14676500
H	-1.71138000	2.49940400	-1.59494200
O	-4.09557100	-0.25956600	-0.40133300
O	-2.11787900	-1.81065300	-0.11858900
C	2.18323900	0.14205900	-0.13442100
C	2.78490300	-1.04345600	-0.58827100
C	2.98005200	1.12200100	0.47987300
C	4.15035400	-1.24443200	-0.42149400
H	2.16852600	-1.80222200	-1.06825600
C	4.34749000	0.91966700	0.63006600
H	2.51528900	2.04049900	0.83592600
C	4.93582000	-0.26387900	0.18462600
H	4.60517700	-2.16941300	-0.77255200
H	4.95677600	1.68771600	1.10385100
H	6.00590500	-0.42172100	0.30929600

1'-AuL$^+$ E_{total} = -1469.29032

C	-0.20104600	0.84480900	0.28263400
C	1.00590200	0.56778600	0.58600800
N	2.21143400	0.19338300	0.82832200
S	3.30059900	-0.08943200	-0.56024800
C	3.97625200	1.53427300	-0.78534900
H	4.46766600	1.83662200	0.14401500
H	4.70355900	1.46933500	-1.60090600
H	3.15720100	2.21018800	-1.04837500
C	2.75033700	-0.07161200	2.17993000
H	3.69196400	0.46939100	2.31240700
H	2.01554000	0.28741500	2.90302300
H	2.91720800	-1.14461400	2.30396400

O	4.30823200	-1.00310900	-0.05409000
O	2.43426500	-0.43875700	-1.66934300
C	-0.94845300	2.08423900	0.10956400
C	-0.30733300	3.32073100	0.29333000
C	-2.30562400	2.06321000	-0.23613300
C	-1.01196900	4.50577300	0.13602100
H	0.74774600	3.34412600	0.56644000
C	-3.00881000	3.25271600	-0.39511900
H	-2.80559300	1.10554400	-0.38127200
C	-2.36446800	4.47389000	-0.20867900
H	-0.50798500	5.45931500	0.28281900
H	-4.06283000	3.22575300	-0.66460500
H	-2.91524400	5.40480900	-0.33117400
Au	-1.03460200	-1.11335300	0.05965400
P	-2.03327800	-3.21512100	-0.21061500
H	-1.31831200	-4.16362000	-0.95387200
H	-2.32278800	-3.93517300	0.95583800
H	-3.27376400	-3.23009600	-0.86179400

2' E_{total} = -324.46730

C	-1.08204500	-0.16534200	-0.00132100
C	0.00940200	-0.97694800	-0.00153900
C	1.11342100	-0.08041000	-0.00015800
H	0.02969900	-2.05986400	-0.00232300
O	-0.68197800	1.12036000	-0.00059400
N	0.71192000	1.17190700	0.00005800
C	2.56423500	-0.40416900	0.00100300
H	3.16301100	0.51292800	0.00105400
H	2.83129700	-0.99569900	0.88539300
H	2.83267000	-0.99660400	-0.88234900
C	-2.54065400	-0.40876700	0.00120900
H	-3.00725300	0.01243800	0.90063600
H	-3.01874200	0.05791700	-0.86905300
H	-2.74445200	-1.48353600	-0.02417700

3' E_{total} = -1315.13297

C	0.77932100	-0.12854600	0.17366200
C	-0.30788300	-0.91456300	0.47107000
N	-1.63266200	-0.58141600	0.78584000
S	-2.76007000	-0.47936600	-0.47024800
C	-3.96285100	-1.70993900	-0.02631000
H	-4.35133200	-1.49039200	0.97262700
H	-4.76905200	-1.65628700	-0.76459200
H	-3.48005700	-2.69075300	-0.05135900
C	-1.89390300	0.25430700	1.96514900
H	-2.93735700	0.14658600	2.28174500

H	-1.24932900	-0.10544600	2.77283700
H	-1.68776100	1.31587100	1.77412800
O	-3.41844200	0.82612800	-0.42199600
O	-2.08930600	-0.90750500	-1.69513300
C	0.71553500	1.34285800	0.09520700
C	0.01056000	1.96397900	-0.94049600
C	1.30951400	2.13658000	1.08386400
C	-0.09657500	3.35265700	-0.98916600
H	-0.45033900	1.34732300	-1.71274900
C	1.20618700	3.52339500	1.03438200
H	1.84782800	1.65340600	1.90129200
C	0.50172600	4.13485000	-0.00356000
H	-0.64829900	3.82432100	-1.80114300
H	1.67146200	4.12971900	1.81035100
H	0.41871200	5.21996200	-0.04206700
N	0.10753300	-2.23375800	0.50886800
C	1.43095100	-2.32409400	0.24241500
C	1.90070500	-1.02076000	0.02897300
C	3.27693300	-0.73725000	-0.37080300
O	4.13557800	-1.62178500	-0.34467500
C	3.64373300	0.64395500	-0.84483600
H	2.89555000	1.05936500	-1.53165700
H	3.71255000	1.33596300	0.00544000
H	4.62019500	0.60112700	-1.33760500
C	2.11938300	-3.63795000	0.18900300
H	2.89504600	-3.71276600	0.96036100
H	1.40343600	-4.45664200	0.33172100
H	2.62638900	-3.77797500	-0.77328000
H	-0.50327100	-3.01885200	0.70808300

A' E_{total} = -1793.792758

C	0.00214800	0.64767700	-0.19486300
C	-0.81043500	-0.42707100	-0.26087500
N	-0.43699600	-1.75257700	-0.56424200
S	-0.14373800	-2.81481000	0.70720400
C	-1.36883700	-4.08150700	0.47268800
H	-1.23628000	-4.53495200	-0.51411600
H	-1.21771400	-4.83257300	1.25455600
H	-2.35838700	-3.62183400	0.56280100
C	0.17317400	-2.07372600	-1.85716500
H	-0.06461300	-3.10597800	-2.13988600
H	-0.26232000	-1.40197300	-2.60287800
H	1.26390300	-1.95000600	-1.84142200
O	1.17725500	-3.41532300	0.52840000
O	-0.45650300	-2.09306100	1.93901300
C	-0.54098700	2.01096000	-0.07869400

C	0.00193500	2.90061900	0.86091700
C	-1.59505800	2.46137600	-0.89138800
C	-0.52816900	4.17611100	1.02565500
H	0.83863700	2.57222100	1.47954000
C	-2.11093800	3.74472500	-0.74030900
H	-2.00031000	1.80409500	-1.66215600
C	-1.58888500	4.60277500	0.22712200
H	-0.10427100	4.84466500	1.77334700
H	-2.92282000	4.07801900	-1.38510500
H	-1.99709700	5.60485200	0.34740600
Au	2.06684500	0.36576400	-0.19468400
P	4.41318500	0.04352800	-0.16654800
H	4.87814900	-1.21513000	0.24336800
H	5.10064100	0.18981300	-1.38111500
H	5.17561600	0.88164700	0.66093400
N	-2.22441800	-0.31371200	-0.02364500
C	-2.95738600	0.12544500	0.99772200
C	-4.30822300	0.03418000	0.61980300
C	-4.31461200	-0.48468000	-0.64485800
H	-5.16659000	0.30349400	1.22129800
O	-3.05219900	-0.70079800	-1.05771200
C	-5.38339300	-0.84513500	-1.59118200
H	-6.35932600	-0.62696000	-1.15021500
H	-5.27968500	-0.28030500	-2.52517700
H	-5.33524100	-1.91235100	-1.83851800
C	-2.36681100	0.62792800	2.25285000
H	-3.01857200	0.37720100	3.09557800
H	-1.36803200	0.21071300	2.41633700
H	-2.28404500	1.72342300	2.20152700

B' **E_{total} = -1793.798358**

C	-0.22169400	-0.51077500	-0.38973300
C	0.65790400	0.63927100	-0.10483600
N	0.52180800	1.74756200	-0.92425100
S	1.31963400	3.21804300	-0.52483000
C	3.03779200	2.84566100	-0.74948400
H	3.30250100	1.98851000	-0.12019200
H	3.19361100	2.62221600	-1.81015700
H	3.59719800	3.74204300	-0.46175000
C	0.05601200	1.56802400	-2.30538800
H	-0.29210100	2.52661300	-2.69212200
H	0.85130100	1.17239100	-2.95109300
H	-0.79398200	0.87538400	-2.30762400
O	0.89675400	4.15521300	-1.55901900
O	1.03682800	3.51916700	0.86919600
C	0.34463300	-1.69888500	-0.87687500

C	-0.45485600	-2.87405700	-0.99314700
C	1.71203100	-1.75608800	-1.28349700
C	0.08728700	-4.04256400	-1.48272500
H	-1.49881600	-2.82870000	-0.68339600
C	2.24365000	-2.92958300	-1.77576200
H	2.34266500	-0.87089200	-1.19077200
C	1.43382300	-4.06764900	-1.87187300
H	-0.52011000	-4.94014400	-1.56546100
H	3.28577100	-2.97245800	-2.08376400
H	1.85808300	-4.99346200	-2.25695100
Au	-2.19526800	-0.20729900	0.15076200
P	-4.45092700	0.20516500	0.76059500
H	-4.73937200	0.22337500	2.13176000
H	-4.99267400	1.42957700	0.34678200
H	-5.41186500	-0.69811500	0.28676200
N	1.44442500	0.62480300	0.90128300
C	1.62067100	-0.31171000	1.88865400
C	2.78941500	-0.99210300	2.05184400
C	3.97678700	-0.87968900	1.21221800
H	2.85918600	-1.65558200	2.91383300
O	4.04862900	-0.15654300	0.21490500
C	5.14730400	-1.72356700	1.63838300
H	5.41864700	-1.51493300	2.68192600
H	4.87574700	-2.78800100	1.59160700
H	6.00880600	-1.53983200	0.99013900
C	0.49293400	-0.46706100	2.86112000
H	0.13662700	0.51594200	3.19475100
H	-0.35976500	-0.96992300	2.37790700
H	0.79097500	-1.05812400	3.73266500

C' **E_{total} = -1793.858122**

C	-0.74272200	-0.30870400	0.21366800
C	0.49356500	-1.04455100	0.12227900
N	1.20211300	-1.35513600	-0.98272500
S	2.89830900	-1.84583300	-0.80863600
C	2.79174600	-3.44814400	-0.05765300
H	2.16638800	-4.07513900	-0.69917000
H	3.81847400	-3.82855600	-0.03135700
H	2.37703200	-3.34519800	0.94634600
C	0.80540700	-0.84705300	-2.29843700
H	1.61166000	-1.03404600	-3.00856500
H	-0.09779200	-1.35563100	-2.64390700
H	0.61813500	0.23577500	-2.23571900
O	3.35630200	-2.01021100	-2.18006400
O	3.55911700	-0.88251600	0.05569200
C	-1.85744800	-0.39971300	-0.78785600

C	-2.62366500	0.72277100	-1.12199200
C	-2.23477700	-1.64398900	-1.31178300
C	-3.73043000	0.61050900	-1.95926000
H	-2.35294700	1.69787400	-0.71203000
C	-3.33829700	-1.75843500	-2.15236100
H	-1.66499500	-2.53660700	-1.04846500
C	-4.09155700	-0.63167100	-2.47669900
H	-4.31166800	1.49707600	-2.20705600
H	-3.61260800	-2.73391800	-2.55038800
H	-4.95690500	-0.72116800	-3.13088900
Au	0.26886200	1.67537300	0.13595000
P	1.27681500	3.79311500	0.09387100
H	1.47083300	4.42159400	1.33107800
H	2.56111500	3.86298100	-0.46202000
H	0.60543000	4.79138800	-0.62440300
N	0.99867600	-1.44137100	1.35603600
C	0.11932000	-1.13138400	2.25638600
C	-1.12811400	-0.52675900	1.69921200
C	-2.29058300	-1.54129200	1.81111800
H	-1.41455700	0.39520700	2.22729200
O	-2.06738200	-2.73189500	1.72402700
C	-3.65258100	-0.96402100	2.00488600
H	-3.71058700	-0.48994000	2.99524300
H	-3.84194200	-0.17311700	1.26522100
H	-4.41439600	-1.74470700	1.92603900
C	0.32877700	-1.37430600	3.69567000
H	-0.47023900	-2.01593700	4.09277700
H	1.29835700	-1.84588300	3.87706800
H	0.27431500	-0.42689500	4.24946100

D' E_{total} = -1315.10628

C	0.74511300	-0.09735600	-0.20010500
C	-0.52536000	-0.49985200	0.05626000
N	-1.57729100	0.27343700	0.56530300
S	-3.06387300	0.23584000	-0.19669700
C	-3.89519400	-1.18916700	0.46412700
H	-3.98745900	-1.06236000	1.54747500
H	-4.88541800	-1.24103000	0.00038700
H	-3.29106100	-2.06877500	0.22107500
C	-1.45508400	0.99231200	1.83763300
H	-2.29397700	0.74827900	2.50073500
H	-0.52701100	0.66269500	2.31412900
H	-1.42173300	2.07781100	1.68879000
O	-3.78005000	1.43246000	0.24255400
O	-2.81797800	0.01888500	-1.61962600
C	1.31520700	1.24554000	-0.21208300

C	0.50410200	2.37368900	-0.43188100
C	2.69624600	1.44780900	-0.06110900
C	1.05273700	3.64905600	-0.47765200
H	-0.56553000	2.24179900	-0.59484200
C	3.24464600	2.72619000	-0.10430200
H	3.35950300	0.59811500	0.10221600
C	2.42531800	3.83415800	-0.30833000
H	0.40453600	4.50540000	-0.65765900
H	4.31860400	2.85463600	0.02233800
H	2.85356000	4.83449700	-0.34418000
N	-0.74271000	-1.87987200	-0.15742400
C	0.38747500	-2.38567100	-0.51417700
C	1.49833300	-1.35075500	-0.53035300
C	2.53135000	-1.78221300	0.52588100
H	1.98444000	-1.30674300	-1.51766000
O	2.30933900	-1.62369100	1.70982200
C	3.78031600	-2.42012300	0.00378800
H	3.54677700	-3.19224100	-0.74195600
H	4.37911500	-1.66089700	-0.52085300
H	4.36929200	-2.84720000	0.82002800
C	0.59133200	-3.82429300	-0.80842600
H	1.25813200	-4.28901300	-0.06677700
H	-0.36679500	-4.35272700	-0.78603200
H	1.05729800	-3.96783300	-1.79284000

E' E_{total} = -1315.10933

C	0.83234100	-0.28393000	0.06930800
C	-0.58773100	-0.65746200	0.25409900
N	-1.58402400	0.32064000	0.41074400
S	-3.04128900	0.14692500	-0.40403800
C	-3.95858100	-1.09670700	0.47308100
H	-4.09849200	-0.76101100	1.50561100
H	-4.92732700	-1.20004800	-0.02634000
H	-3.38615800	-2.02693600	0.43310200
C	-1.54076100	1.23253400	1.56173400
H	-2.33125300	0.99291700	2.28502700
H	-0.57706400	1.10142700	2.06225200
H	-1.63883900	2.27330900	1.23947500
O	-3.72839700	1.42553100	-0.24029700
O	-2.73573700	-0.34267600	-1.74333100
C	1.28676400	1.11505100	0.04691900
C	0.79301400	1.98053700	-0.93704900
C	2.14426600	1.61526100	1.03276000
C	1.17034800	3.32020200	-0.94521900
H	0.11035300	1.59709200	-1.69652200
C	2.51287700	2.95732500	1.02803500

H	2.50917300	0.94813500	1.81491300
C	2.02838300	3.81139400	0.03785900
H	0.78740700	3.98448800	-1.71813500
H	3.17767800	3.33815500	1.80169100
H	2.31653800	4.86131500	0.03507400
N	-0.81937800	-1.92048100	0.21494400
C	0.46350000	-2.56960700	-0.00424300
C	1.47759500	-1.46521900	-0.11165000
C	2.87312000	-1.75350200	-0.50489400
O	3.19119200	-2.91706600	-0.72961100
C	3.86946100	-0.64305400	-0.65476200
H	3.47418600	0.17945000	-1.26452600
H	4.12459100	-0.21962800	0.32520600
H	4.77900900	-1.04105200	-1.11450900
C	0.72844800	-3.55962500	1.12718800
H	0.77063500	-3.02871300	2.08793600
H	-0.07908000	-4.29886400	1.17506800
H	1.67950700	-4.07699000	0.96481100
H	0.41886400	-3.12045400	-0.95843100

$TS_{A'}$ Etotal = -1793.761279

C	0.13479600	0.61454000	-0.15713500
C	-0.43936500	-0.54878800	-0.24170200
N	-0.35681100	-1.82491000	-0.55449600
S	-0.28743900	-2.99339000	0.72103300
C	-1.93619600	-3.64513200	0.75092100
H	-2.18031900	-4.04005900	-0.23980200
H	-1.94729500	-4.44925600	1.49374900
H	-2.61686600	-2.83897300	1.03743300
C	-0.14867600	-2.27846700	-1.94132700
H	-0.65035900	-3.23671100	-2.10727200
H	-0.58455400	-1.52813100	-2.60587600
H	0.92361500	-2.38520500	-2.14164100
O	0.63509600	-4.02056600	0.25888500
O	-0.03110300	-2.24437600	1.94047800
C	-0.44771900	1.95126900	-0.03522300
C	0.06966000	2.86119500	0.89680900
C	-1.51113000	2.35145700	-0.85780200
C	-0.49436500	4.12498900	1.03686100
H	0.91073100	2.56220900	1.52320100
C	-2.05995300	3.62307400	-0.72880200
H	-1.89706700	1.66309300	-1.60929600
C	-1.56073200	4.50989600	0.22464400
H	-0.09317200	4.81616900	1.77626600
H	-2.88325300	3.92212100	-1.37590300
H	-1.99497500	5.50295400	0.32779300

Au	2.23706300	0.35205900	-0.19092800
P	4.57327400	0.10345100	-0.18730900
H	5.07100400	-1.19482000	-0.00605200
H	5.26619400	0.49739000	-1.34077700
H	5.27903700	0.80241300	0.80212300
N	-2.45550500	-0.34898200	0.14608900
C	-3.21737500	0.29178000	1.00869200
C	-4.52757500	0.42094100	0.48496300
C	-4.47725300	-0.17625300	-0.73942900
H	-5.38378900	0.88673900	0.95660200
O	-3.23121400	-0.64457900	-0.95859800
C	-5.46932100	-0.40797800	-1.80793600
H	-6.41901200	0.06757700	-1.54752500
H	-5.11789000	0.00480200	-2.76125300
H	-5.64378800	-1.48129200	-1.95311400
C	-2.66372300	0.76716100	2.30022400
H	-3.34414500	0.52664700	3.12452100
H	-1.68708900	0.30914500	2.49518100
H	-2.53755700	1.85834800	2.27822100

$TS_{B'}$ Etotal = -1793.770065

C	-0.05239300	0.68521500	-0.31006400
C	-0.77663500	-0.50893200	-0.33241000
N	-0.22946900	-1.71284900	-0.82252000
S	0.10685900	-2.95999400	0.25513500
C	-1.18184200	-4.14216200	-0.04769400
H	-1.13601900	-4.44977700	-1.09691600
H	-1.00581500	-4.99954900	0.60954800
H	-2.14065800	-3.66533200	0.17750400
C	0.39380200	-1.76239600	-2.14752100
H	0.24654600	-2.75332400	-2.59182700
H	-0.11327200	-1.02589700	-2.77815500
H	1.46869300	-1.54170600	-2.11536300
O	1.38237400	-3.55276200	-0.13989800
O	-0.05948000	-2.42279000	1.60371800
C	-0.71612600	1.96947000	-0.29322100
C	-0.09995500	3.06827800	0.34251100
C	-1.96540200	2.17349600	-0.92123300
C	-0.73132400	4.30120200	0.40407300
H	0.87669500	2.92739100	0.80735000
C	-2.57806700	3.41725600	-0.88633900
H	-2.43454100	1.35426700	-1.46458300
C	-1.97138700	4.47872600	-0.21218600
H	-0.25207000	5.13164300	0.91861600
H	-3.53249100	3.56469800	-1.38832500
H	-2.45931500	5.45124900	-0.18032100

Au	2.01118700	0.50656700	-0.08304800
P	4.34379400	0.27284400	0.26849400
H	4.79685900	-0.98222300	0.69951900
H	5.19152500	0.50113800	-0.82522700
H	4.92547800	1.11068900	1.23126600
N	-1.99815600	-0.60725500	0.20898800
C	-2.63991900	-0.11081000	1.26830200
C	-4.01670600	-0.05566800	1.12330000
C	-4.37256500	-0.54921700	-0.13550500
H	-4.70688600	0.24649700	1.90198200
O	-3.39042800	-0.87502800	-0.89312900
C	-5.75689100	-0.76851400	-0.63253000
H	-6.49315600	-0.36644000	0.06935900
H	-5.88679100	-0.29517700	-1.61234800
H	-5.93358400	-1.84366000	-0.76117200
C	-1.86935600	0.26970800	2.48431400
H	-2.52700200	0.27431700	3.35822500
H	-1.03278500	-0.42047300	2.64312000
H	-1.45960100	1.28156900	2.35541900

$TS_{C'}$ Etotal = -1793.793854

C	0.26852300	-0.55948700	-0.20196500
C	0.87826600	0.77924200	-0.01579100
N	1.10011800	1.53817100	-1.13710500
S	1.94839500	3.05399100	-1.02328300
C	3.48187200	2.61567700	-0.24825500
H	3.29582100	2.22450000	0.75367200
H	3.98542200	1.87941700	-0.88094500
H	4.05673700	3.54707900	-0.20890200
C	0.37313200	1.30212000	-2.39459700
H	0.14811700	2.26039800	-2.86786600
H	0.96155100	0.69452800	-3.09089700
H	-0.57363000	0.79932200	-2.16987400
O	2.20892400	3.39926200	-2.41564200
O	1.19603400	3.98555600	-0.19767300
C	0.82194000	-1.52250100	-1.09214400
C	0.11399300	-2.73407100	-1.32043000
C	2.08492100	-1.35941300	-1.72640400
C	0.63788400	-3.72660000	-2.12475400
H	-0.85610700	-2.86969600	-0.84149100
C	2.60351800	-2.35504100	-2.53253500
H	2.65318400	-0.44617500	-1.56939400
C	1.88490800	-3.53789200	-2.72965400
H	0.08483400	-4.64823900	-2.28936600
H	3.57041100	-2.21782900	-3.01106200
H	2.29775900	-4.31764600	-3.36705600

Au	-1.61201300	-0.77154300	0.68217900
P	-3.75033600	-0.95837900	1.67118300
H	-3.97464500	-0.21148600	2.83622800
H	-4.84545000	-0.57353400	0.88549600
H	-4.14992600	-2.23606900	2.08646000
N	1.10460700	1.28201700	1.16863000
C	0.98298200	0.41003300	2.20011700
C	1.44207500	-0.89393900	2.13624900
C	2.61300800	-1.36177800	1.37592700
H	1.09802300	-1.57723700	2.91661700
O	3.39659800	-0.60416600	0.81118300
C	2.82076400	-2.84909100	1.39042800
H	3.09359500	-3.17626900	2.40379800
H	1.89110700	-3.37615300	1.12974900
H	3.61894500	-3.13036300	0.69692300
C	0.29063400	0.94157000	3.40796500
H	0.90214800	1.74599100	3.84040000
H	-0.67388400	1.39163000	3.13695600
H	0.13980400	0.16955400	4.16836400

$TS_{E'}$ Etotal = -1315.08826

C	-0.27670900	-2.48854400	-0.52034500
C	-1.27720800	-1.80027700	0.17541700
N	-2.60261400	-2.28449200	0.34017300
S	-3.08079300	-2.65901100	1.89871300
C	-3.42895900	-1.12259500	2.72493700
H	-4.24889700	-0.62244700	2.19994200
H	-3.72732000	-1.36498600	3.74979800
H	-2.52111500	-0.51303600	2.70632300
C	-3.59911400	-1.68973400	-0.55628900
H	-3.67683900	-0.60115700	-0.41782600
H	-3.29140900	-1.89916400	-1.58688700
H	-4.57032400	-2.16157500	-0.38304300
O	-4.33389900	-3.40259800	1.78761900
O	-1.92450800	-3.28294300	2.53819100
C	-0.37808700	-3.78942300	-1.19515800
C	-0.92906300	-4.89909500	-0.54006200
C	0.06846600	-3.93706200	-2.51517800
C	-1.02057900	-6.12652900	-1.18929800
H	-1.27483500	-4.79411600	0.48785500
C	-0.01755800	-5.16731700	-3.16047400
H	0.46932400	-3.07394200	-3.04906000
C	-0.56264700	-6.26559900	-2.49848200
H	-1.44767400	-6.98117700	-0.66697000
H	0.33491000	-5.26502100	-4.18602400
H	-0.63394100	-7.22794500	-3.00278800

N	-0.89511700	-0.62335100	0.69010900
C	0.40774200	-0.46320700	0.34512800
C	0.85151100	-1.63210000	-0.42765500
C	2.17771800	-1.75701000	-1.12863800
O	2.55438000	-0.85606000	-1.85594400
C	2.98412400	-2.98532400	-0.85852700
H	3.45698500	-2.87395100	0.12904500
H	2.35596600	-3.88297200	-0.80822300
H	3.76749500	-3.10574200	-1.61253600
C	1.14377700	0.82064900	0.53550100
H	0.98042800	1.47563900	-0.32958400
H	0.77656800	1.33017300	1.43236500
H	2.22367500	0.66807900	0.63479700
H	1.06479800	-1.45178400	0.88800300

$TS_{3'}$ E_{total} = -1315.07942

C	1.22965100	-0.51281700	0.30009900
C	-0.05631600	-0.88929200	0.70822500
N	-1.17569400	-0.04755100	0.86999500
S	-2.25312800	0.04319300	-0.41119900
C	-3.64202000	-0.91162700	0.14743400
H	-4.02468900	-0.46407400	1.06982400
H	-4.40426900	-0.88478400	-0.63730800
H	-3.29703500	-1.93441700	0.32486300
C	-1.14194500	1.05831000	1.82999900
H	-2.15903700	1.30360800	2.15435300
H	-0.57504300	0.71756300	2.70200000
H	-0.66893200	1.95876700	1.41478100
O	-2.68212300	1.43321900	-0.55867900
O	-1.64560600	-0.65441800	-1.54507000
C	1.62258700	0.89509100	0.09595600
C	1.13565600	1.60472400	-1.00524800
C	2.42519400	1.54968700	1.03533800
C	1.45659200	2.95018000	-1.16890800
H	0.50140300	1.09604200	-1.73220900
C	2.74326200	2.89452900	0.87200500
H	2.79274500	0.99729800	1.90151300
C	2.25963100	3.59659400	-0.23115700
H	1.07442800	3.49570800	-2.03003100
H	3.36628300	3.39717500	1.60993100
H	2.50625300	4.64935600	-0.35833000
N	-0.19490000	-2.22661500	0.83431000
C	1.06118000	-2.77675900	0.41816600
C	1.94978000	-1.70601100	0.11101100
C	3.33465200	-1.91182500	-0.35294900
O	3.80329900	-3.04503800	-0.39825200

C	4.15933100	-0.73016400	-0.77476500
H	3.61636200	-0.08132900	-1.47360900
H	4.41717200	-0.11126600	0.09502200
H	5.08253700	-1.08900000	-1.23921300
C	1.34557600	-4.21346500	0.71446300
H	1.97860800	-4.29508900	1.60728800
H	0.40495300	-4.74116000	0.90908800
H	1.87664000	-4.69907100	-0.10836200
H	0.01232100	-2.75207500	-0.32375200

Figure S2 (Me)

1'-AuL$^+$ E_{total} = -1277.69597

C	0.08345600	1.62226700	0.01476500
C	1.09466400	0.98042200	0.42394100
N	2.07227200	0.23087400	0.81209200
S	2.97405400	-0.68050400	-0.41787400
C	4.25721500	0.46316800	-0.84713700
H	4.83621000	0.69873800	0.05062000
H	4.88825300	-0.03737200	-1.58872200
H	3.79405700	1.35644100	-1.27585600
C	2.42555800	0.00387800	2.22938200
H	3.48618800	0.22101500	2.38558200
H	1.81862900	0.68317100	2.83095400
H	2.21444400	-1.03447100	2.49741100
O	3.52225600	-1.81520500	0.30220600
O	2.05896600	-0.84893000	-1.53063200
Au	-1.43558000	0.11507900	0.00170200
P	-3.19785600	-1.42958700	-0.06056800
H	-2.88716200	-2.72756000	-0.48741100
H	-3.85464700	-1.68479400	1.15064000
H	-4.27532100	-1.10935900	-0.89684100
C	-0.24814900	3.01021600	-0.40627300
H	0.62915300	3.66497400	-0.39006300
H	-0.67121400	3.00183400	-1.41704500
H	-1.01695900	3.42526300	0.25545100

2' E_{total} = -324.46730

C	-1.08204500	-0.16534200	-0.00132100
C	0.00940200	-0.97694800	-0.00153900
C	1.11342100	-0.08041000	-0.00015800
H	0.02969900	-2.05986400	-0.00232300
O	-0.68197800	1.12036000	-0.00059400
N	0.71192000	1.17190700	0.00005800
C	2.56423500	-0.40416900	0.00100300
H	3.16301100	0.51292800	0.00105400
H	2.83129700	-0.99569900	0.88539300

H	2.83267000	-0.99660400	-0.88234900
C	-2.54065400	-0.40876700	0.00120900
H	-3.00725300	0.01243800	0.90063600
H	-3.01874200	0.05791700	-0.86905300
H	-2.74445200	-1.48353600	-0.02417700

A' E_{total} **= -1602.19578 small imaginary freq. corresponding to PH3 rotation (-14.6 cm^{-1})**

C	-0.07371200	-1.21521900	-0.05705700
C	0.92880400	-0.33345900	-0.22235000
N	0.81289400	1.01310900	-0.63128700
S	0.75406400	2.22093300	0.53023300
C	2.23570300	3.15517800	0.22101400
H	2.20513500	3.55418300	-0.79716500
H	2.26755100	3.97510200	0.94524500
H	3.09448000	2.48857000	0.35266900
C	0.32292100	1.33705600	-1.97270900
H	0.68093700	2.32628000	-2.27950000
H	0.73017400	0.59444700	-2.66601900
H	-0.77422400	1.32626400	-2.02335100
O	-0.39459200	3.08504700	0.26135000
O	0.88680600	1.56649600	1.83052700
Au	-2.04073600	-0.54598500	-0.09717400
P	-4.29417800	0.19756400	-0.10056100
H	-4.51666200	1.58256200	-0.06473300
H	-5.09450100	-0.15945900	-1.19689600
H	-5.11525700	-0.21910400	0.95847500
N	2.30433700	-0.70979300	-0.01754600
C	3.01298900	-1.07016300	1.05203400
C	4.35895900	-1.16833700	0.65842100
C	4.39106400	-0.82843900	-0.66544400
H	5.19794400	-1.44107700	1.28498800
O	3.14823300	-0.54717800	-1.09598900
C	5.46851200	-0.72208000	-1.66367600
H	6.43700200	-0.89384000	-1.18689500
H	5.32941500	-1.46427200	-2.45923900
H	5.46843300	0.27100800	-2.12763900
C	2.39883400	-1.31143200	2.37288200
H	3.07617500	-0.96902300	3.16151500
H	1.43825300	-0.79577000	2.46412700
H	2.23875300	-2.38834500	2.51664300
C	0.17631600	-2.66490700	0.20316000
H	1.22575400	-2.97663700	0.09290400
H	-0.15797700	-2.92640000	1.21814800
H	-0.43213600	-3.27739800	-0.47474100

B' E_{total} **= -1602.18653**

C	-0.16248600	-0.63667500	-0.76337900
C	0.87296100	0.19700800	-0.15349200
N	1.05272100	1.48254900	-0.56637800
S	2.01105200	2.57868000	0.37316300
C	3.64547800	1.90601400	0.24597200
H	3.93202700	1.90380200	-0.80910400
H	4.29243700	2.57060900	0.82799400
H	3.64947500	0.89384000	0.65895200
C	0.29683400	2.00332700	-1.71166100
H	0.87177200	2.80354800	-2.18143000
H	0.16843000	1.20112800	-2.44739500
H	-0.68199300	2.38829200	-1.40351100
O	1.94393000	3.83050900	-0.36703500
O	1.55162800	2.52915300	1.75146400
Au	-2.09589000	-0.28726300	-0.17773600
P	-4.33937800	0.11080700	0.50392900
H	-4.57724200	1.27135300	1.25164500
H	-5.29230200	0.23660800	-0.51553000
H	-4.92736900	-0.86877500	1.31516700
N	1.43883700	-0.31863300	0.87249000
C	1.69353400	-1.57463500	1.32473000
C	2.59960700	-2.41414500	0.73237400
C	3.42399200	-2.11226600	-0.44078400
H	2.80912500	-3.35132100	1.24854700
O	3.25934100	-1.11676900	-1.14888300
C	4.50468500	-3.11222000	-0.74060700
H	4.07178900	-4.11356800	-0.87163100
H	5.04990700	-2.82692300	-1.64472600
H	5.20508200	-3.18118500	0.10301500
C	0.98625600	-1.94347400	2.58844700
H	1.31660400	-2.91353700	2.97175100
H	1.15329300	-1.17594100	3.35420000
H	-0.09847600	-1.98249000	2.40792100
C	0.26898600	-1.78186500	-1.53951800
H	1.13073900	-1.56318100	-2.19095500
H	0.70115100	-2.49744300	-0.78991200
H	-0.54450600	-2.29904900	-2.05299600

C' E_{total} = -1602.18224

C	-0.10089800	1.04133300	-0.74078600
C	-1.09694400	0.13998500	-0.23995200
N	-1.65153100	-0.94527800	-0.82611000
S	-2.33651900	-2.21173900	0.19088700
C	-3.86474400	-1.52701700	0.77202300
H	-4.44535400	-1.21314400	-0.09970000
H	-4.37024300	-2.34461600	1.29707700

H	-3.65082400	-0.69356300	1.44211100
C	-1.26651800	-1.34505700	-2.18135100
H	-1.40559900	-0.49645300	-2.85373700
H	-0.22206900	-1.68759700	-2.20330900
H	-1.91448700	-2.15952100	-2.50616000
O	-2.63931800	-3.28587700	-0.74404900
O	-1.41510900	-2.45727700	1.28903900
Au	1.58868800	-0.22770900	-0.05167400
P	3.46283000	-1.46891100	0.61437700
H	3.52771000	-1.82810800	1.96713500
H	3.65314100	-2.70652200	-0.01445100
H	4.71303000	-0.86444100	0.42579900
N	-1.54476500	0.47250000	1.04032500
C	-1.03207900	1.62222500	1.34489000
C	-0.20623200	2.20591600	0.24203400
C	-1.04237000	3.37106400	-0.35798700
H	0.75586900	2.60289400	0.59554600
O	-2.08646100	3.12805500	-0.92412400
C	-0.47627500	4.74082200	-0.19159100
H	0.47311900	4.80354000	-0.74368800
H	-1.17156700	5.49694500	-0.56553300
H	-0.23344400	4.93505400	0.86257600
C	-1.28291500	2.30500600	2.62979800
H	-0.33372800	2.53723700	3.13179500
H	-1.78992800	3.26624300	2.46045800
H	-1.90310100	1.68862700	3.28618700
C	0.18878800	1.40305400	-2.18675700
H	-0.69567900	1.82789400	-2.68305800
H	0.97843600	2.16615500	-2.20636900
H	0.55255300	0.56614300	-2.78863700

F' E_{total} = -1602.22203

C	0.01856600	-0.75022600	-0.52030600
C	0.98037100	0.15796400	0.16200800
N	1.13928300	1.46205800	-0.28330200
S	2.57022700	2.33440700	0.14823400
C	3.91740800	1.24077500	-0.23015300
H	3.72390500	0.76019200	-1.19340800
H	4.78852100	1.90216800	-0.29144300
H	4.03810700	0.50500200	0.56597700
C	0.04064900	2.36076000	-0.66867000
H	0.06498800	3.26839000	-0.05565500
H	0.10748400	2.64136200	-1.72403000
H	-0.91719200	1.87128100	-0.46994400
O	2.58947300	3.43329700	-0.81264600
O	2.55195900	2.66651600	1.56803400

Au	-2.23744200	-0.22435200	-0.20599100
P	-4.35475000	0.25337400	0.69827600
H	-4.38974100	1.24491600	1.68494000
H	-5.30266200	0.71411000	-0.22255300
H	-5.02400700	-0.80970100	1.31480000
N	1.74214000	-0.28110400	1.09169100
C	1.94104300	-1.58680300	1.47466100
C	2.51807900	-2.55019200	0.70112200
C	2.95072100	-2.42823900	-0.68962800
H	2.75286200	-3.49354200	1.19532400
O	2.66288700	-1.47697700	-1.42077700
C	3.79551000	-3.56369900	-1.19971000
H	3.27740700	-4.52243100	-1.06145600
H	4.02764400	-3.42117000	-2.25907800
H	4.73084200	-3.62722200	-0.62667500
C	1.60182700	-1.83693600	2.90881200
H	1.83570500	-2.86301600	3.20900300
H	2.14823300	-1.13745300	3.55467400
H	0.53112500	-1.65118300	3.07613400
C	-0.53810600	-0.57925200	-1.75820700
H	-0.39943200	0.33011800	-2.34130800
H	-0.01571100	-1.74459000	-0.06788100
H	-0.98344300	-1.42880600	-2.27518600

$TS_{A'}$ E_{total} = -1602.16688

C	-0.29890700	-1.21246200	-0.08577900
C	0.55537200	-0.25190900	-0.26912200
N	0.79351100	1.00599600	-0.58876100
S	1.01771900	2.17002500	0.66695900
C	2.78172100	2.36514900	0.72356300
H	3.15259400	2.58621000	-0.28215500
H	2.98839300	3.20248200	1.39773700
H	3.21890000	1.44188900	1.11517900
C	0.77362100	1.47345600	-1.98592300
H	1.48619800	2.29278900	-2.12330600
H	1.06952500	0.63470000	-2.62106800
H	-0.23395800	1.81496000	-2.24928900
O	0.41318900	3.39912600	0.17280300
O	0.55782400	1.54305000	1.89542600
Au	-2.26536000	-0.45063900	-0.11234800
P	-4.48768700	0.32434600	-0.10974000
H	-4.68424900	1.71199200	-0.15792900
H	-5.31481100	-0.09765600	-1.16057800
H	-5.27380900	-0.01679400	1.00017500
N	2.45843900	-0.98375600	-0.07858000
C	3.23331500	-1.34420600	0.92623600

C	4.59390200	-1.23115400	0.54482800
C	4.56223600	-0.76981000	-0.73697500
H	5.46702500	-1.45805600	1.14360900
O	3.27930800	-0.60839500	-1.12204800
C	5.59866300	-0.43553400	-1.73321000
H	6.59277100	-0.55265200	-1.29271600
H	5.52432800	-1.09313900	-2.60804700
H	5.48673500	0.59811900	-2.08283500
C	2.65470500	-1.77626900	2.22274300
H	3.29038100	-1.44835400	3.05196200
H	1.65028800	-1.36016400	2.36037800
H	2.58658100	-2.87098100	2.26968700
C	-0.06969800	-2.66145100	0.14946800
H	0.97654500	-2.94928400	-0.01460600
H	-0.35350300	-2.92071600	1.17873100
H	-0.71048300	-3.26123600	-0.50775100

TS$_{B'}$ E$_{total}$ = -1602.16867

C	0.12574900	-1.10516100	-0.22643100
C	1.02411300	-0.02550600	-0.27776400
N	0.63638800	1.24085600	-0.77169900
S	0.33786900	2.47083300	0.33610100
C	1.65014600	3.62952900	0.04658000
H	1.60421800	3.96457200	-0.99405000
H	1.49843000	4.47463600	0.72536500
H	2.59931800	3.12631700	0.25394200
C	0.03523000	1.35434200	-2.10266400
H	0.22032700	2.35435200	-2.50956500
H	0.53092300	0.62744100	-2.75352600
H	-1.04713200	1.16821900	-2.09359700
O	-0.92903800	3.10235200	-0.02522100
O	0.50762100	1.89339200	1.66802700
Au	-1.89778100	-0.72709800	-0.09530900
P	-4.21577500	-0.26163300	0.15826900
H	-4.58612600	1.09073900	0.15308200
H	-5.09276400	-0.78509700	-0.80241500
H	-4.82237600	-0.70258300	1.34293900
N	2.26416700	-0.12196700	0.17524300
C	2.91789500	-0.75659400	1.15688400
C	4.18903400	-1.19994000	0.86012700
C	4.51556100	-0.92639300	-0.48380500
H	4.86175400	-1.63809600	1.58869900
O	3.60977800	-0.38384100	-1.18452200
C	5.84603200	-1.20424600	-1.09755900
H	6.54254400	-1.62364300	-0.36593000
H	5.72681300	-1.90979100	-1.92854500

H	6.26229400	-0.27817200	-1.51109100
C	2.26337200	-0.85270200	2.49731300
H	3.02301700	-0.80694100	3.28380700
H	1.52933800	-0.05242000	2.63705300
H	1.74996000	-1.81982200	2.58427900
C	0.63125800	-2.48796900	-0.17126100
H	1.72431700	-2.59381800	-0.19907600
H	0.21993300	-3.01600500	0.70183900
H	0.20950200	-3.02762300	-1.03503300

$TS_{C'}$ E_{total} = -1602.26620

C	0.07976400	0.01336300	-0.90351300
C	-1.07790900	-0.23055000	-0.01221500
N	-1.94284500	-1.22193700	-0.40300000
S	-3.44995900	-1.44464100	0.41470000
C	-4.27636000	0.10181100	0.15859700
H	-4.35999000	0.26867200	-0.91884600
H	-5.26474300	-0.00787900	0.61693600
H	-3.70422500	0.89764900	0.64080400
C	-1.55194700	-2.19242900	-1.43354800
H	-1.84060900	-1.85778300	-2.43613300
H	-0.46569600	-2.34686400	-1.38392500
H	-2.02876000	-3.15295900	-1.22860200
O	-4.11205300	-2.48510000	-0.36150300
O	-3.20804000	-1.65850800	1.83253200
Au	1.96116300	-0.37624600	-0.21886100
P	4.15445500	-0.85618800	0.58258300
H	4.51594700	-0.24036400	1.78824700
H	4.43109200	-2.20118600	0.85943100
H	5.22885500	-0.50905400	-0.24644400
N	-1.25173200	0.48800500	1.03130100
C	-0.42030000	1.53864800	1.38418900
C	-0.38773100	2.74280700	0.74899200
C	-1.25409700	3.17162900	-0.35204900
H	0.24999900	3.51075400	1.18835800
O	-2.05280200	2.42817900	-0.92359100
C	-1.12609300	4.61905600	-0.73862300
H	-0.08298300	4.85868500	-0.98717000
H	-1.77052800	4.84628500	-1.59253400
H	-1.40191000	5.26314800	0.10771500
C	0.33317900	1.29509500	2.65182400
H	0.96035300	2.15042700	2.92125300
H	-0.37709200	1.10045800	3.46672700
H	0.96242500	0.39780000	2.56103700
C	-0.18719300	0.52724900	-2.23931200
H	-1.24038000	0.67172700	-2.50071100

H	0.35745600	1.48448500	-2.33438000
H	0.33876900	-0.11259700	-2.96937100

$TS_{F'}$ E_{total} = -1602.16854 small imaginary freq. corresponding to PH3 bending (-8 cm^{-1})

C	0.12133000	0.38303200	-0.93946800
C	-0.98549600	-0.20642900	-0.12121400
N	-1.43489400	-1.44028100	-0.54111200
S	-2.62650200	-2.27356400	0.39777300
C	-4.03839700	-1.20190400	0.32766700
H	-4.31705700	-1.08196600	-0.72299800
H	-4.83112100	-1.71256500	0.88427500
H	-3.78365300	-0.24422000	0.78794400
C	-0.85204300	-2.11555700	-1.70686800
H	-1.22027700	-3.14210900	-1.73158400
H	-1.14755500	-1.62010000	-2.63854200
H	0.24057100	-2.14207100	-1.62237400
O	-2.91546700	-3.47991600	-0.36834100
O	-2.15350000	-2.40327000	1.76735400
Au	2.06047900	0.03083000	-0.20399900
P	4.21748100	-0.45920300	0.60593400
H	4.77238400	0.45297200	1.51333600
H	4.36300200	-1.66809300	1.29885400
H	5.23719500	-0.56109800	-0.34918400
N	-1.43967700	0.35836100	0.92921400
C	-1.11742100	1.62411300	1.35736700
C	-1.59960500	2.76654500	0.78484300
C	-2.53239600	2.84132500	-0.33769900
H	-1.37456000	3.70586000	1.29103800
O	-2.79460000	1.88747800	-1.07771400
C	-3.17986700	4.18164900	-0.54855900
H	-2.41566900	4.96206000	-0.66612900
H	-3.82338400	4.16115300	-1.43276200
H	-3.77681900	4.45802200	0.33133800
C	-0.29786900	1.66011900	2.60645700
H	-0.08905500	2.68503100	2.92854700
H	-0.82495800	1.13078100	3.41114000
H	0.65364400	1.12981200	2.45217400
C	-0.19239100	1.00726900	-2.13127600
H	-1.22159300	1.07436000	-2.49911700
H	0.02810900	1.63899100	-0.89873700
H	0.59022100	1.50082000	-2.70924900

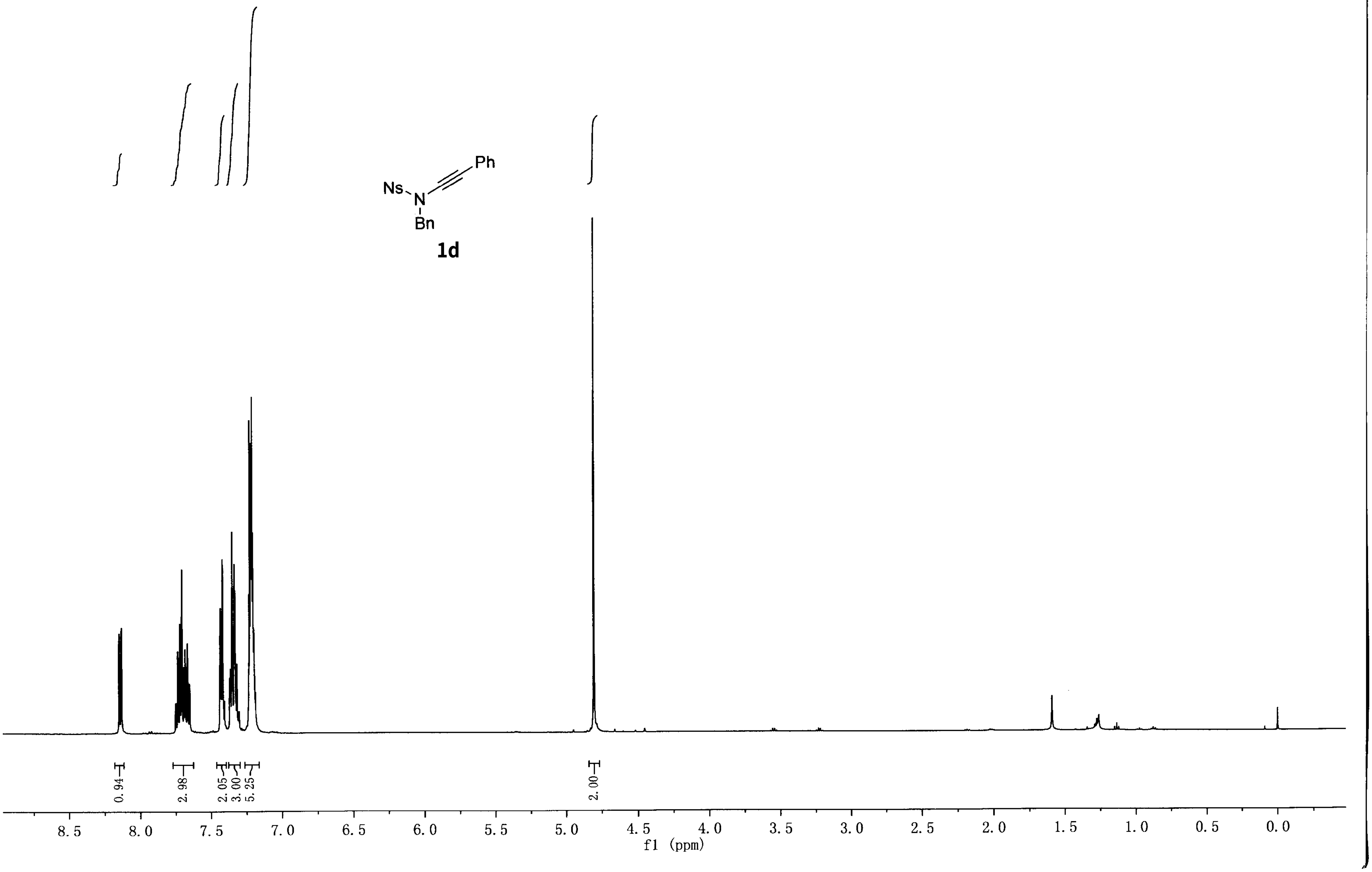
Ph
Ns
N
Bn
1d
0.94
2.98
2.05
3.00
5.25
2.00
8.5
8.0
7.5
7.0
6.5
6.0
5.5
5.0
4.5
4.0
3.5
3.0
2.5
2.0
1.5
1.0
0.5
0.0
f1 (ppm)

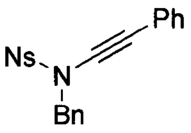
Ph
Ns
N
Bn

1d

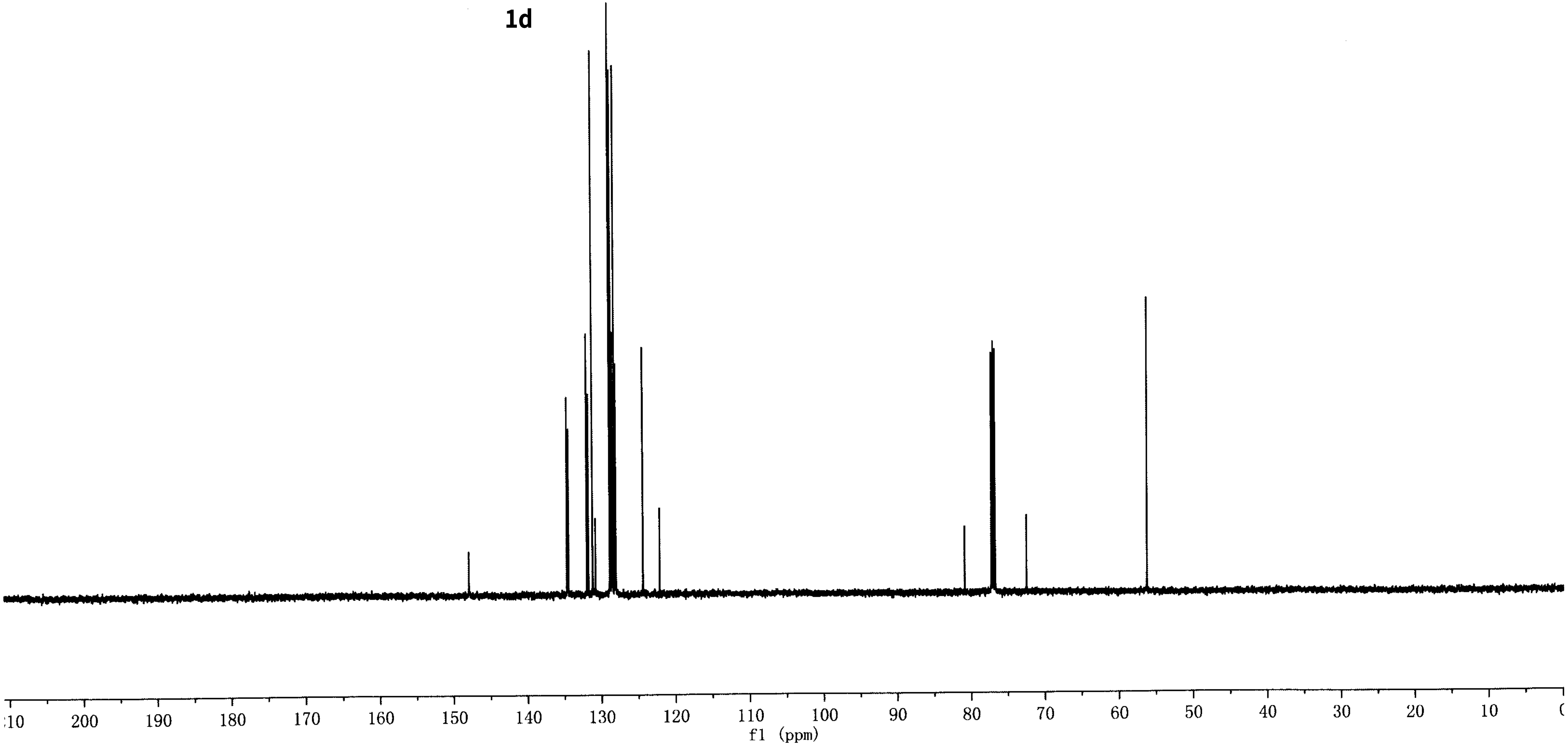
200
190
180
170
160
150
140
130
120
110
100
90
80
70
60
50
40
30
20
10
f1 (ppm)

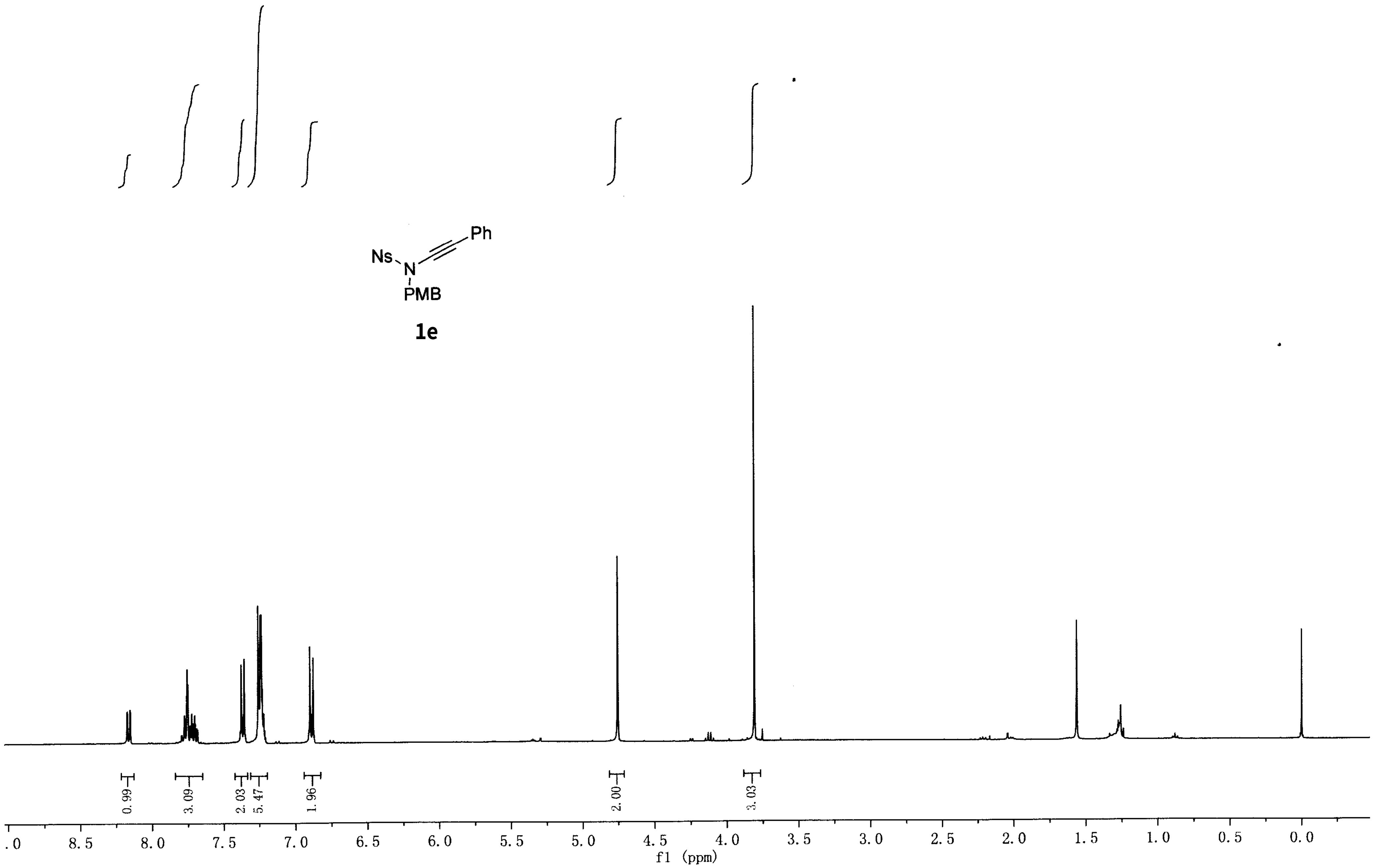
Ph
Ns
N
PMB
1e
0.99
3.09
2.03
5.47
1.96
2.00
3.03
.0
8.5
8.0
7.5
7.0
6.5
6.0
5.5
5.0
4.5
4.0
3.5
3.0
2.5
2.0
1.5
1.0
0.5
0.0
f1 (ppm)

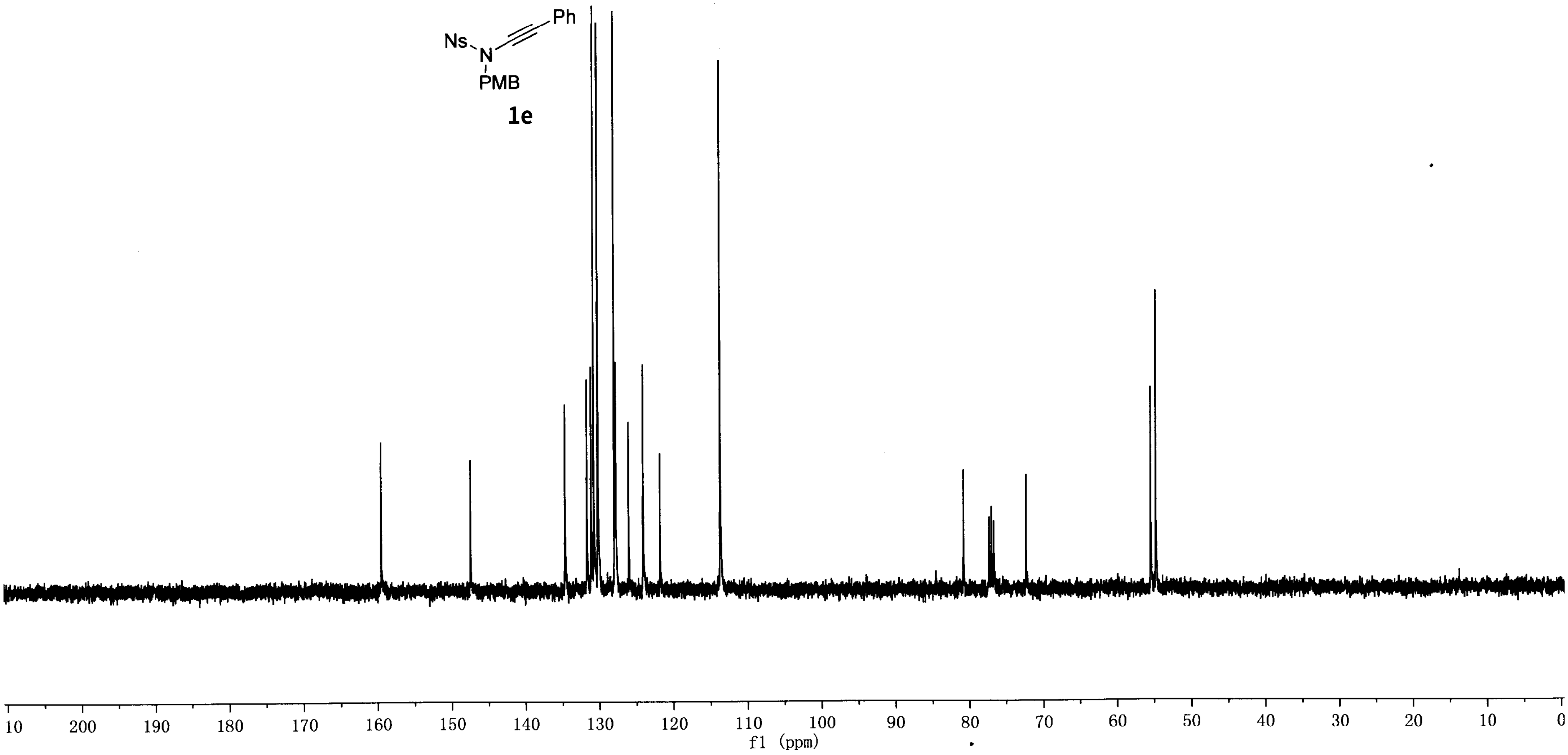

Ph
Ns
N
PMB
1e
10 200 190 180 170 160 150 140 130 120 110 100 90 80 70 60 50 40 30 20 10 0
f1 (ppm)

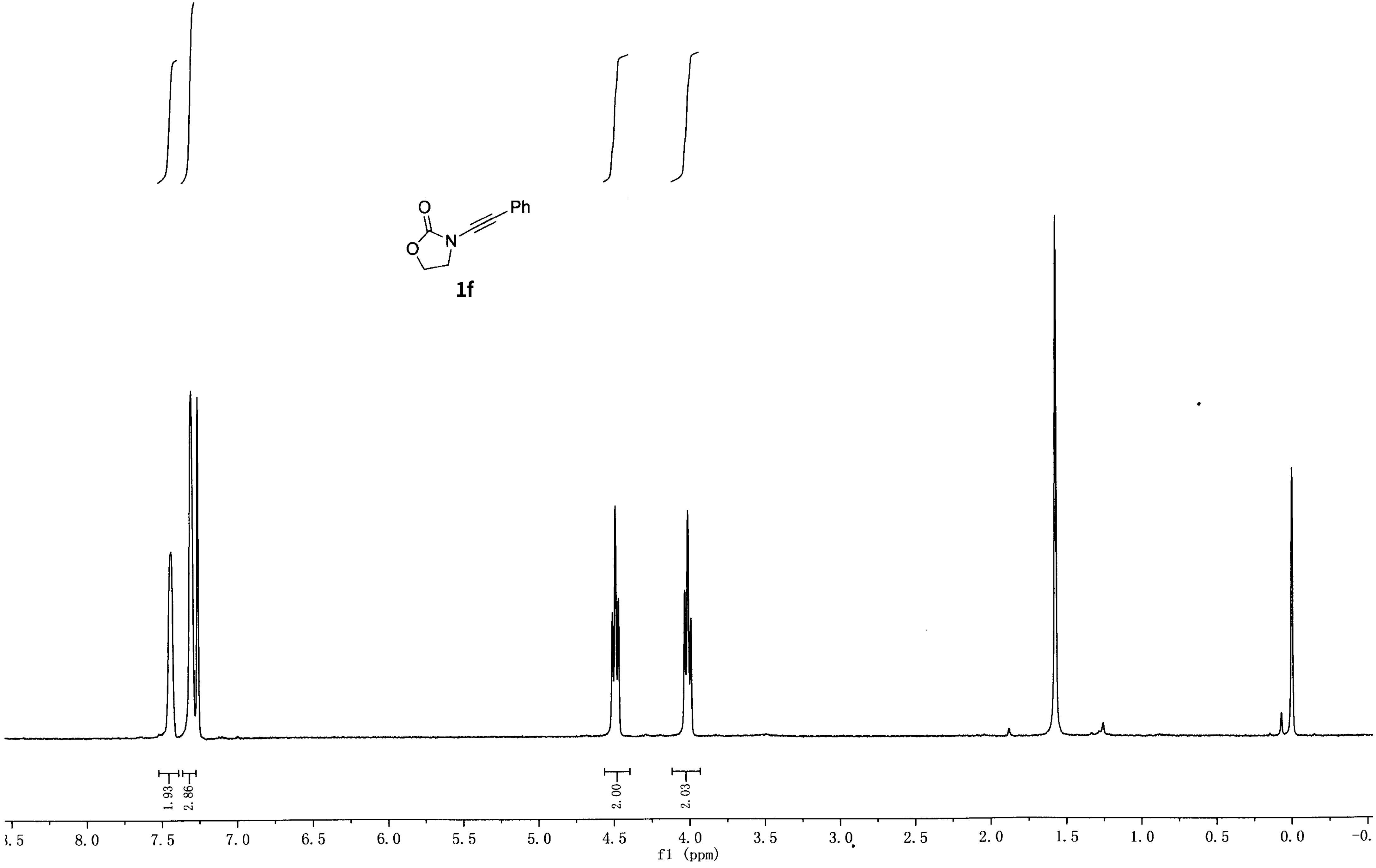
Ph
O
N
O
1f
1.93
2.86
2.00
2.03
8.0
7.5
7.0
6.5
6.0
5.5
5.0
4.5
4.0
3.5
3.0
2.5
2.0
1.5
1.0
0.5
0.0
f1 (ppm)

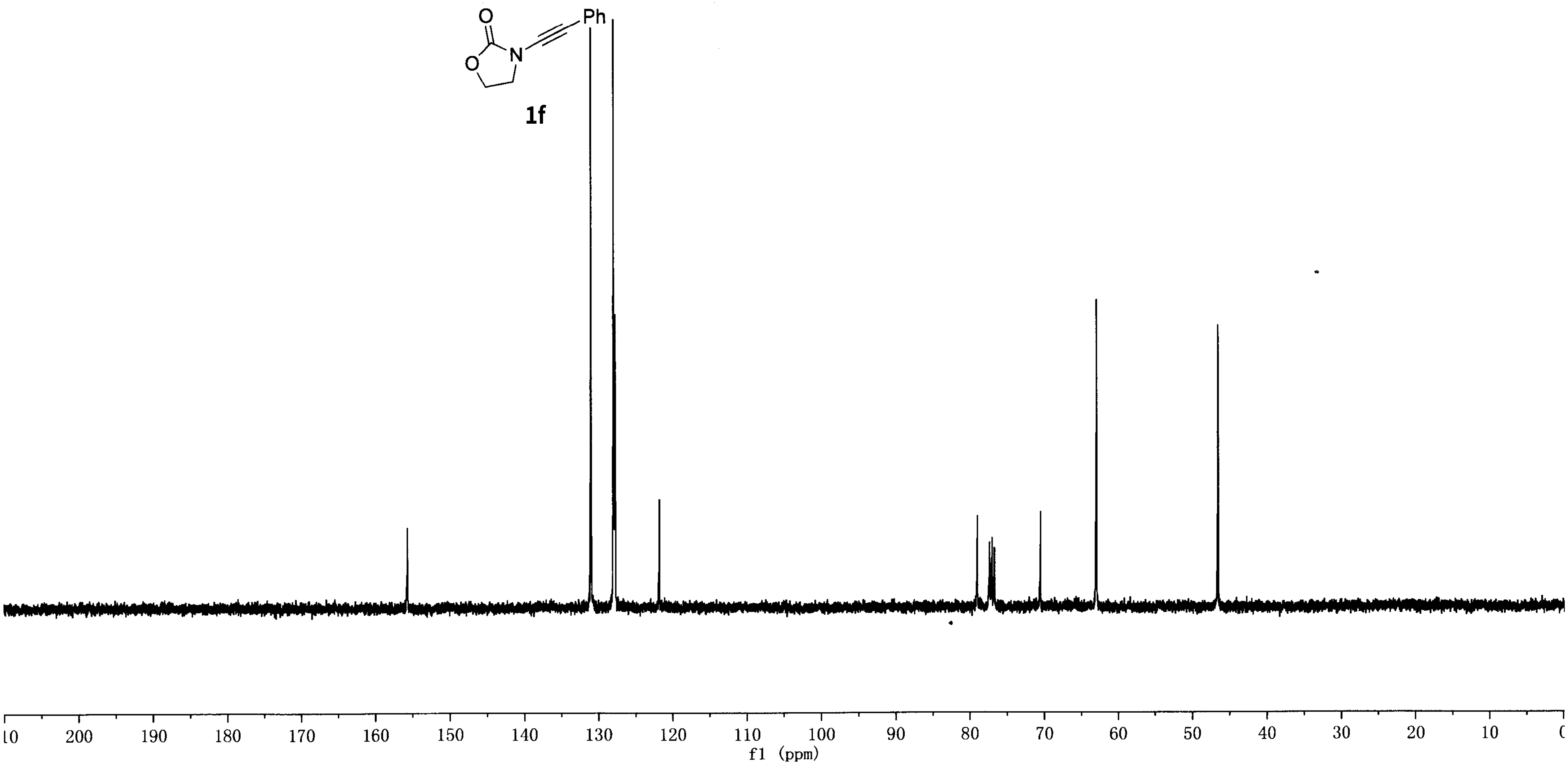
O
O
N
Ph
1f
10
200
190
180
170
160
150
140
130
120
110
100
90
80
70
60
50
40
30
20
10
f1 (ppm)

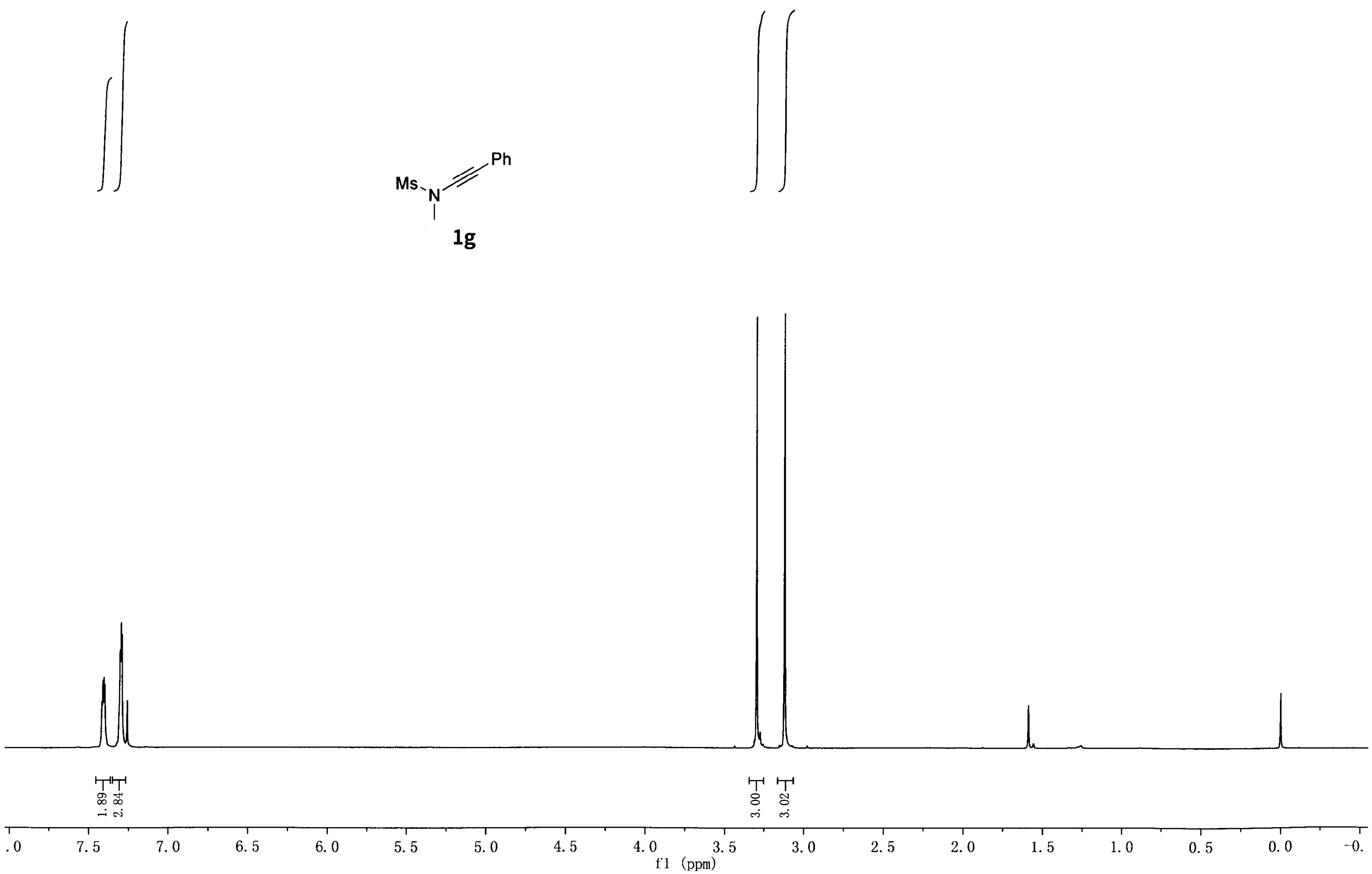

Ms
N
Ph
1g
1.89
2.84
3.00
3.02
.0
7.5
7.0
6.5
6.0
5.5
5.0
4.5
4.0
3.5
3.0
2.5
2.0
1.5
1.0
0.5
0.0
-0.
f1 (ppm)

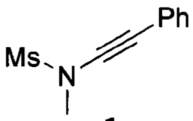
Ph
Ms
N

1g

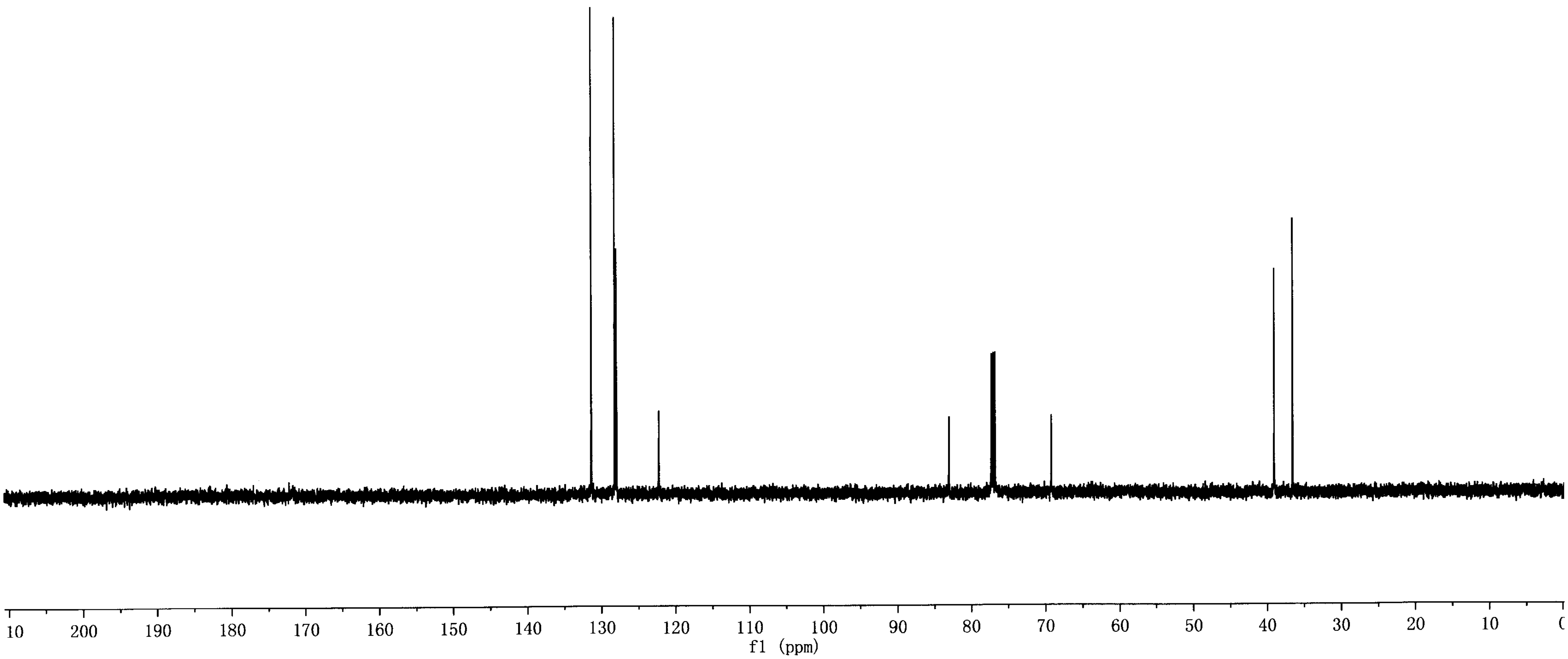
10
200
190
180
170
160
150
140
130
120
110
100
90
80
70
60
50
40
30
20
10
f1 (ppm)

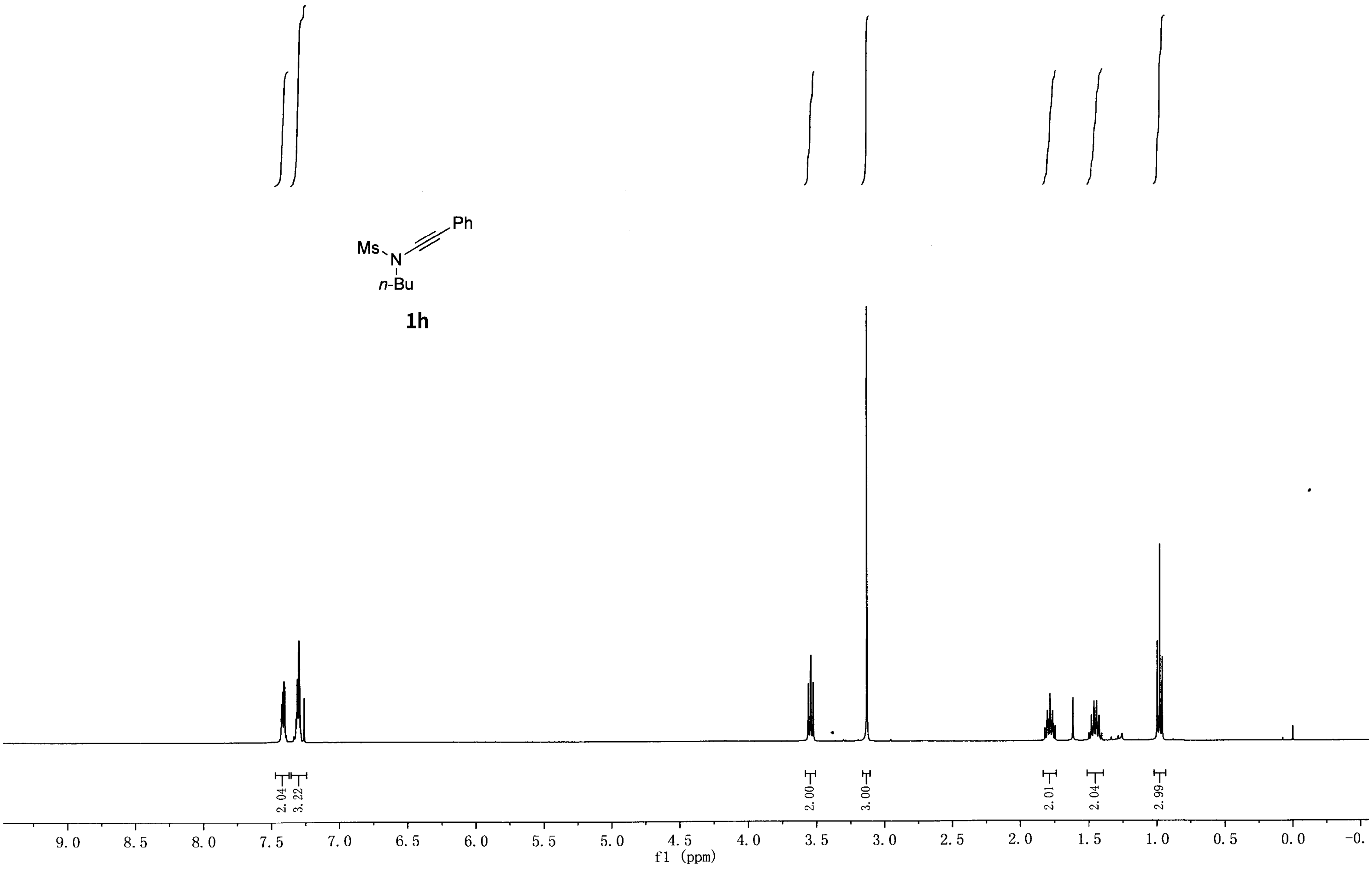

Ph
Ms
N
n-Bu
1h
2.04
3.22
2.00
3.00
2.01
2.04
2.99
9.0
8.5
8.0
7.5
7.0
6.5
6.0
5.5
5.0
4.5
4.0
3.5
3.0
2.5
2.0
1.5
1.0
0.5
0.0
-0.
f1 (ppm)

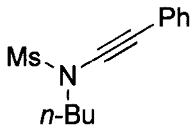

Ph
Ms
N
n-Bu

1h

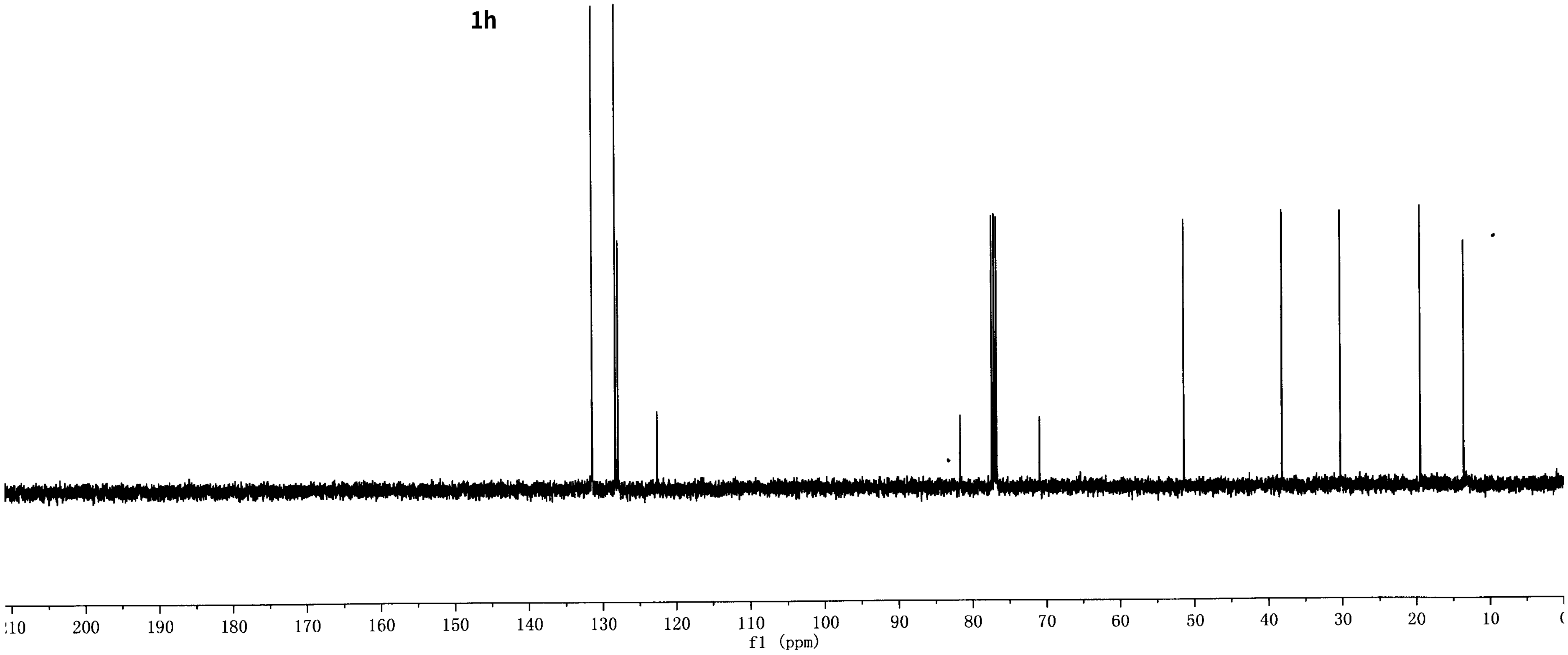

210 200 190 180 170 160 150 140 130 120 110 100 90 80 70 60 50 40 30 20 10 0
f1 (ppm)

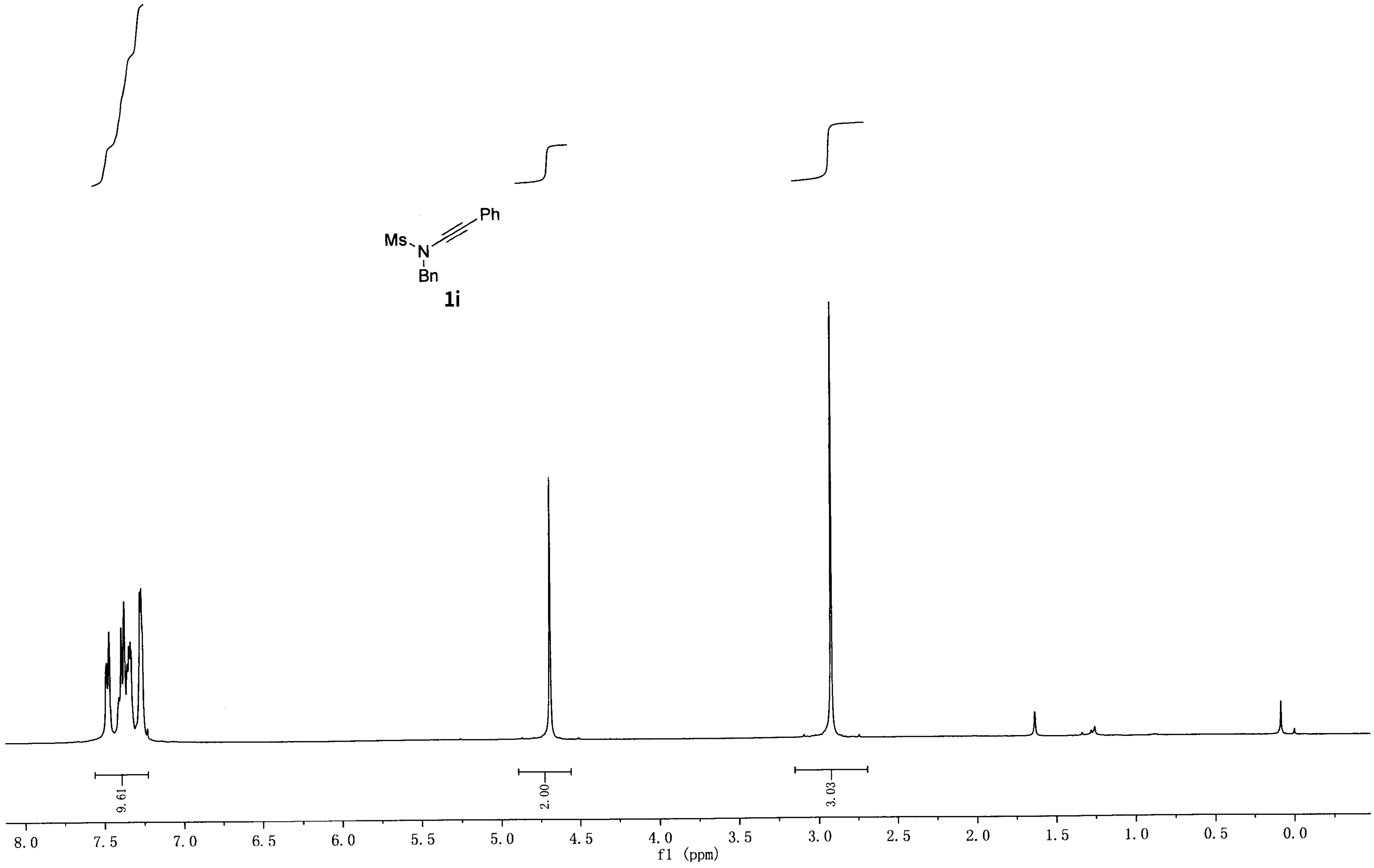
Ph
Ms
N
Bn
1i
9.61
2.00
3.03
8.0 7.5 7.0 6.5 6.0 5.5 5.0 4.5 4.0 3.5 3.0 2.5 2.0 1.5 1.0 0.5 0.0
f1 (ppm)

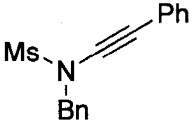

Ms
N
Bn
Ph

1i

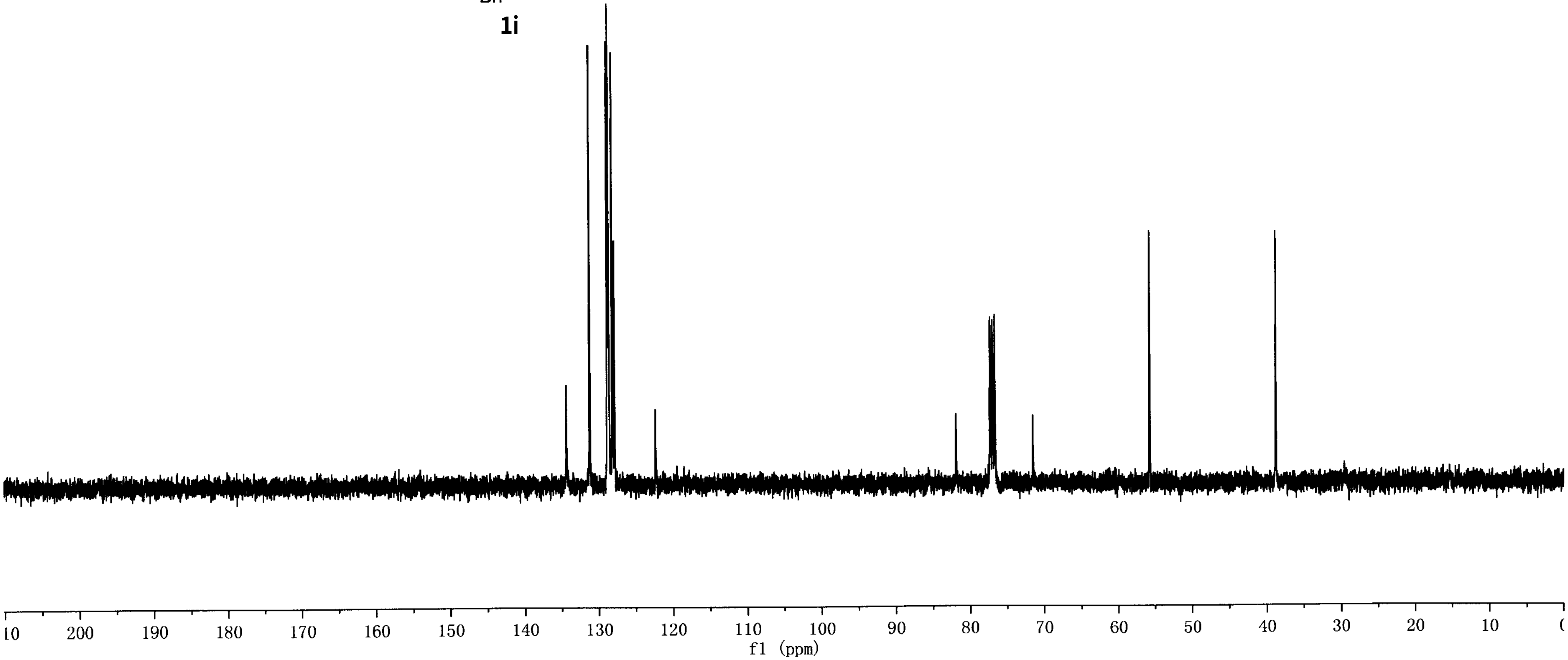

10
200
190
180
170
160
150
140
130
120
110
100
90
80
70
60
50
40
30
20
10
f1 (ppm)

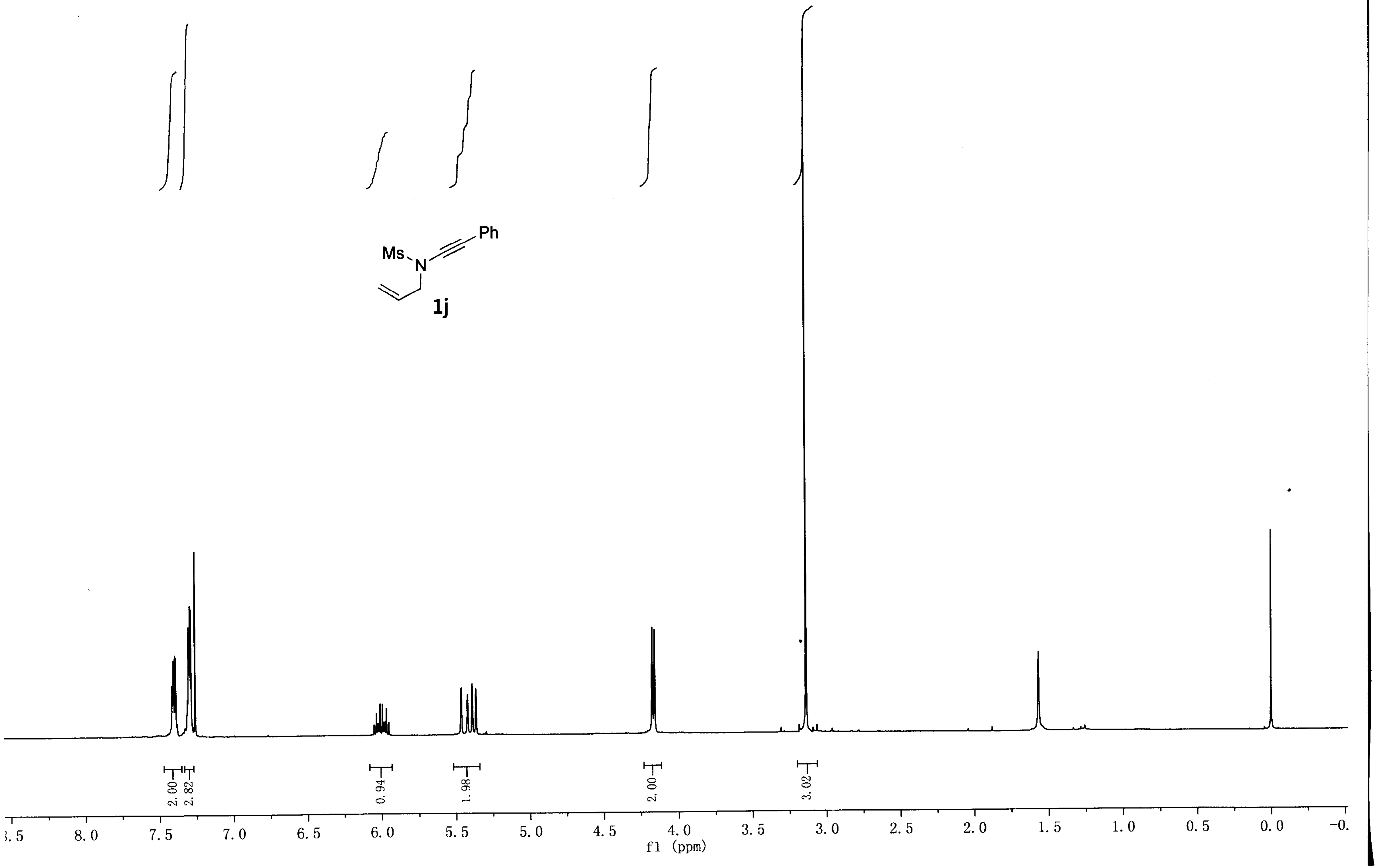
Ph
Ms
N
1j
2.00
2.82
0.94
1.98
2.00
3.02
8.0
7.5
7.0
6.5
6.0
5.5
5.0
4.5
4.0
3.5
3.0
2.5
2.0
1.5
1.0
0.5
0.0
f1 (ppm)

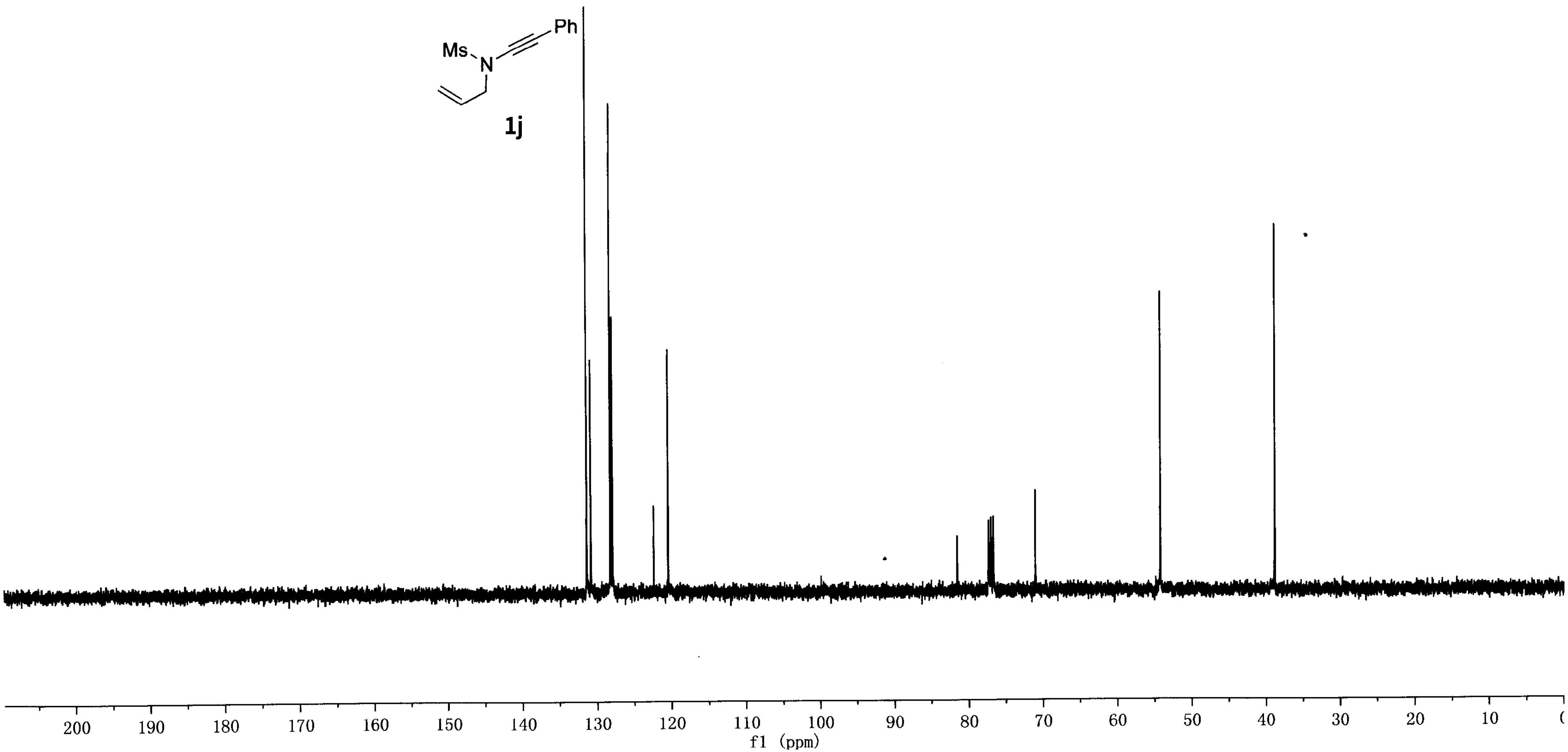
Ms
N
Ph
1j
200
190
180
170
160
150
140
130
120
110
100
90
80
70
60
50
40
30
20
10
f1 (ppm)

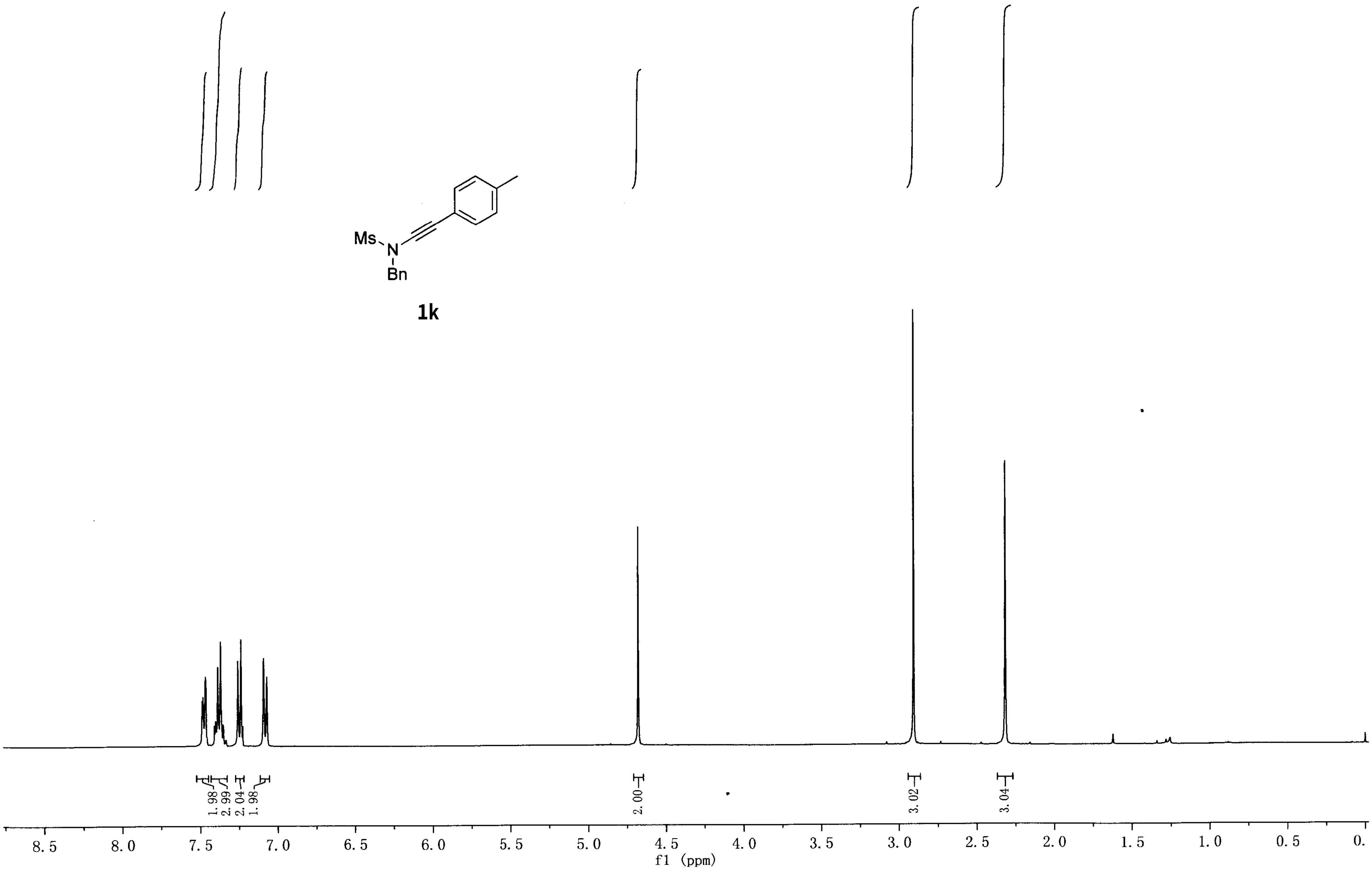
Ms
N
Bn
1k
1.98
2.99
2.04
1.98
2.00
3.02
3.04
8.5 8.0 7.5 7.0 6.5 6.0 5.5 5.0 4.5 4.0 3.5 3.0 2.5 2.0 1.5 1.0 0.5 0.
f1 (ppm)

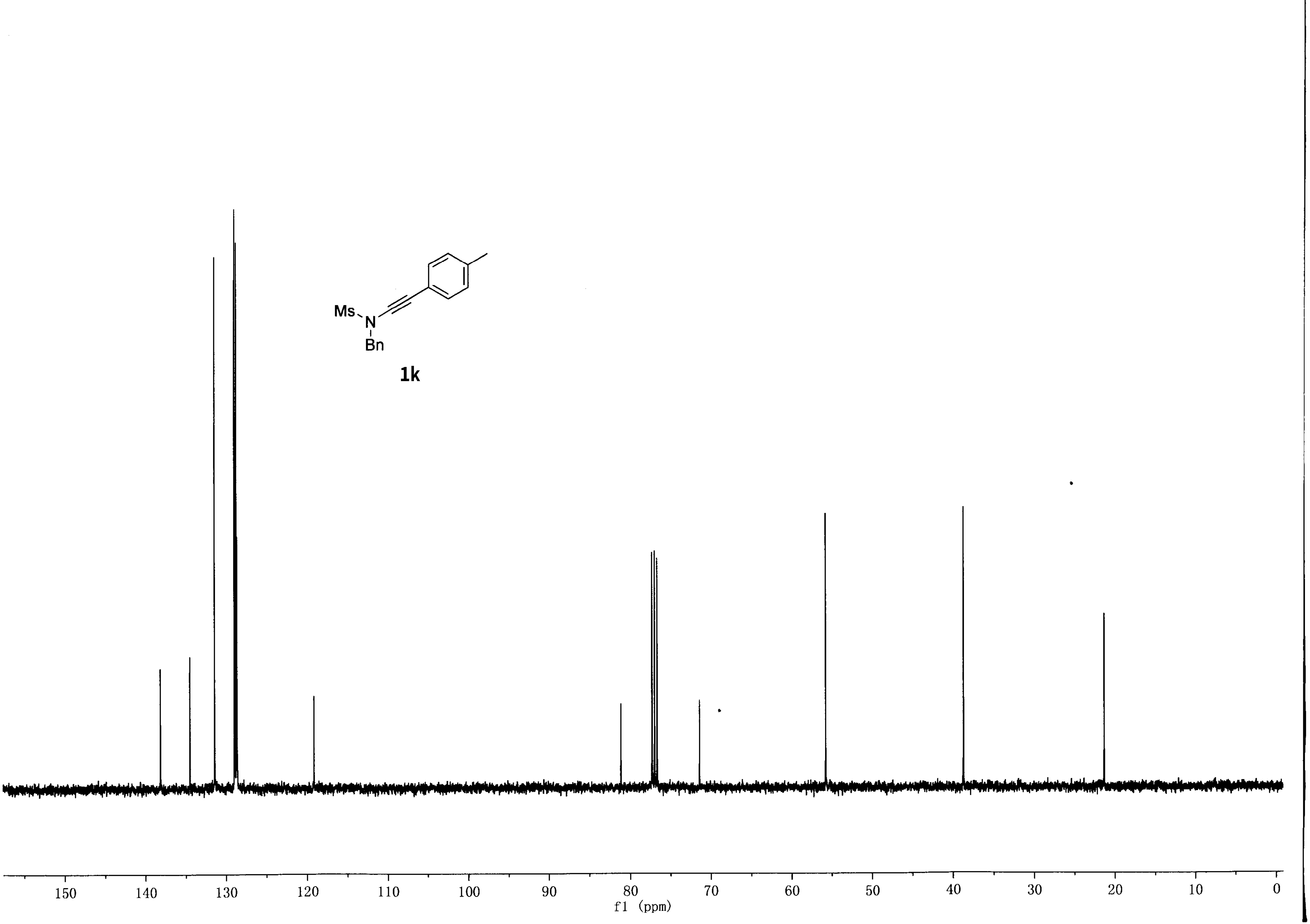
Ms
N
Bn
1k
150
140
130
120
110
100
90
80
70
60
50
40
30
20
10
0
f1 (ppm)

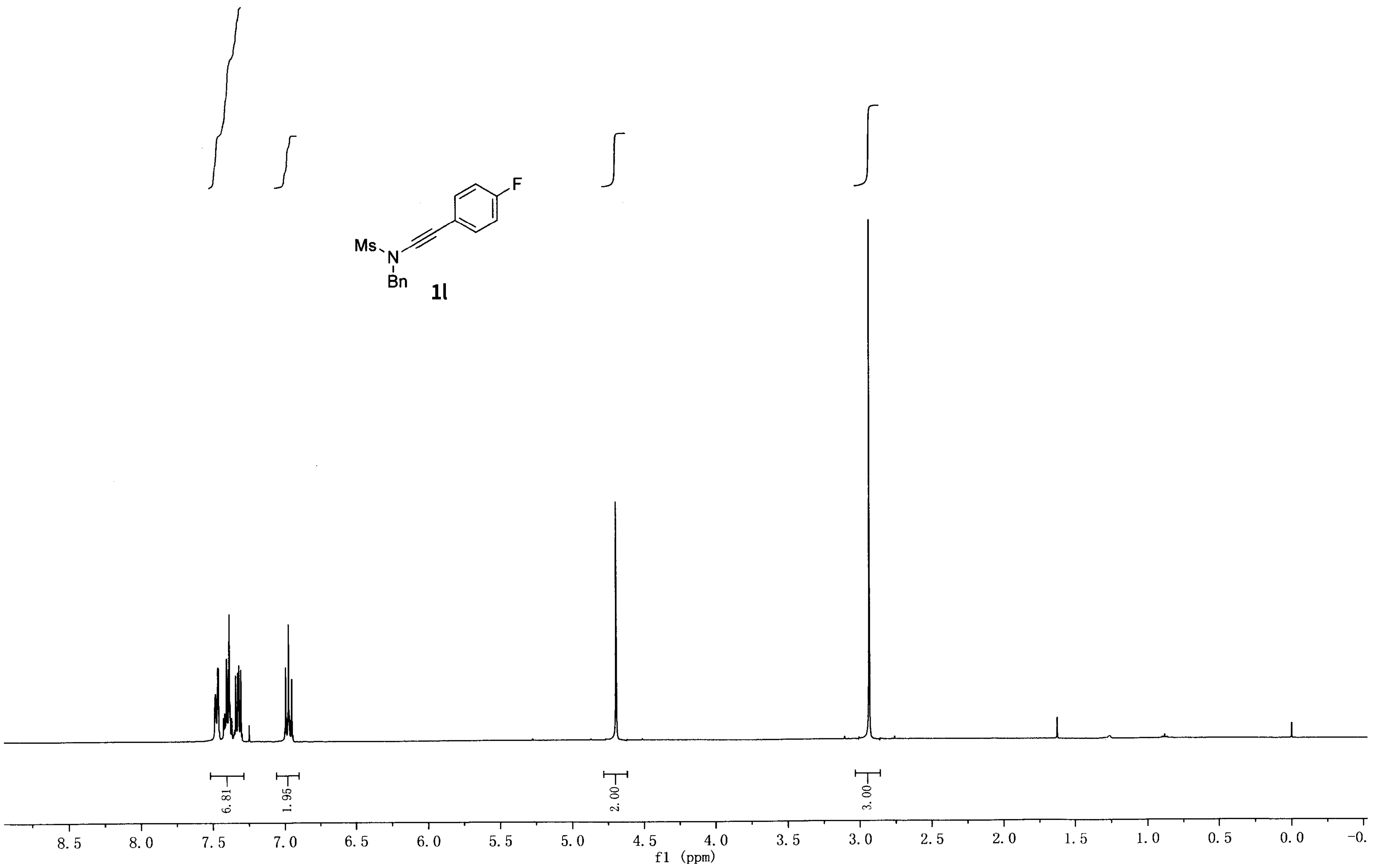
Ms
N
Bn
F
1l
6.81
1.95
2.00
3.00
8.5
8.0
7.5
7.0
6.5
6.0
5.5
5.0
4.5
4.0
3.5
3.0
2.5
2.0
1.5
1.0
0.5
0.0
-0.
f1 (ppm)

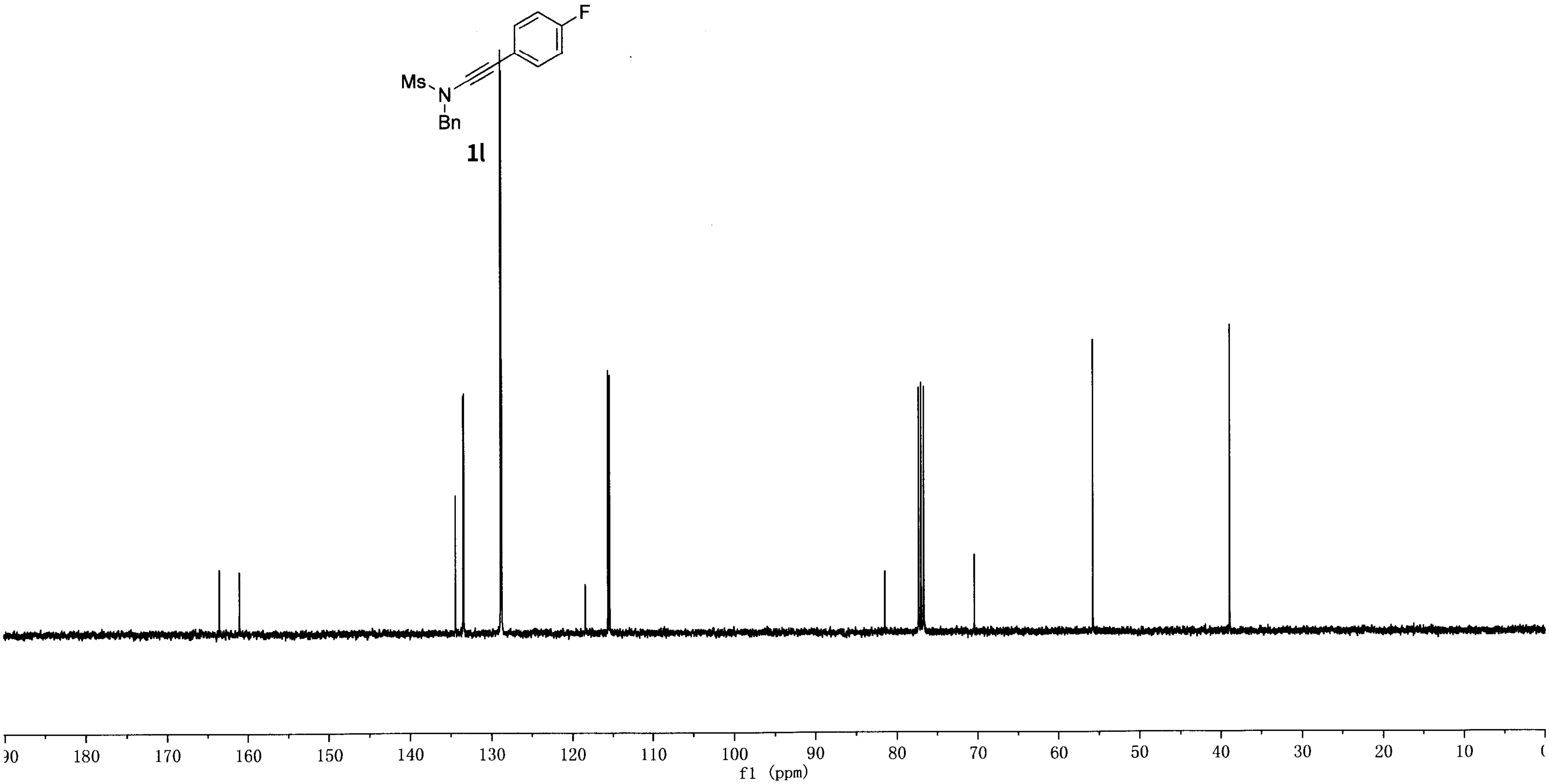
F
Ms
N
Bn
1l
180
170
160
150
140
130
120
110
100
90
80
70
60
50
40
30
20
10
f1 (ppm)

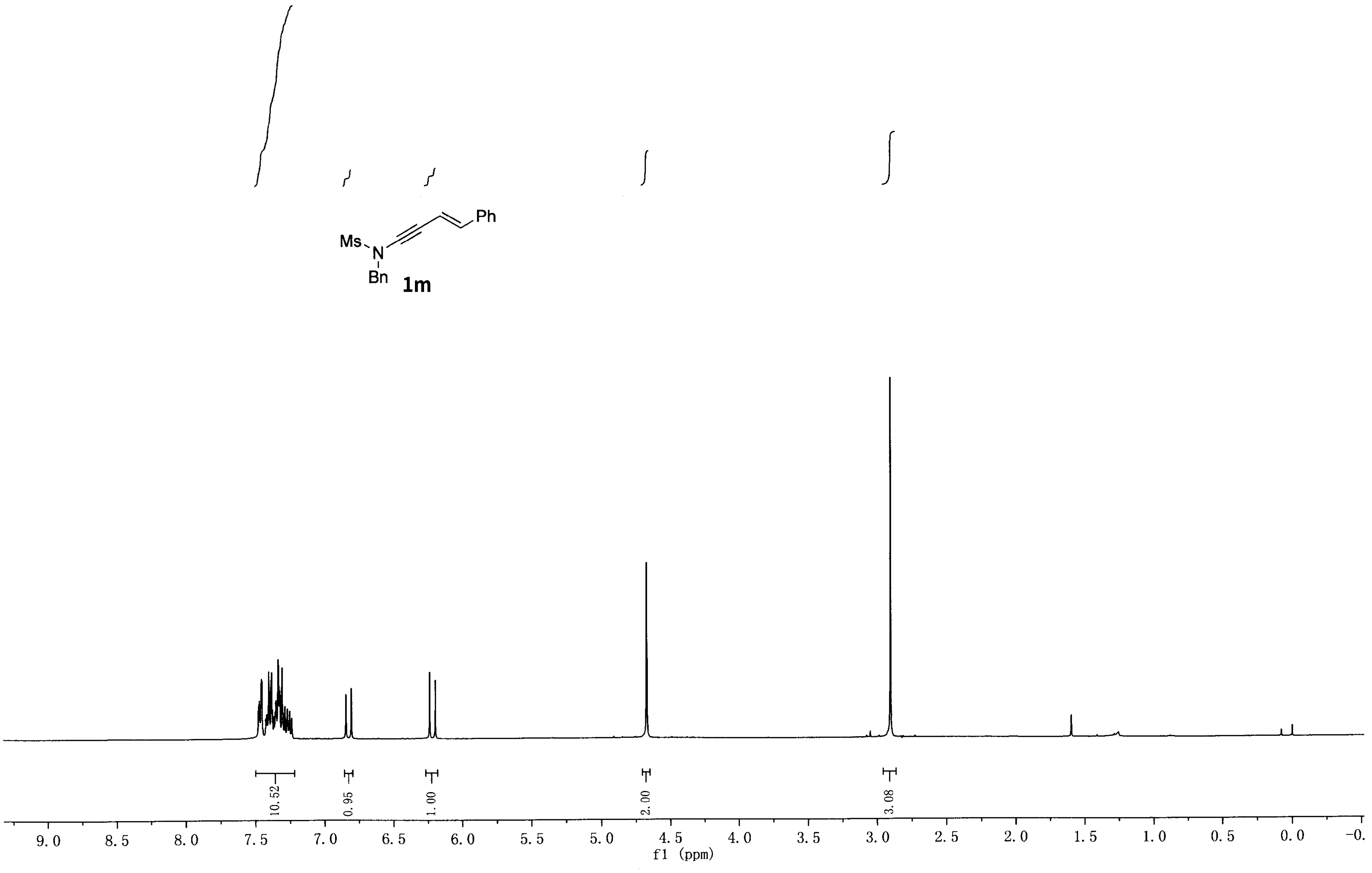

Ph
Ms
N
Bn
1m
10.52
0.95
1.00
2.00
3.08
9.0
8.5
8.0
7.5
7.0
6.5
6.0
5.5
5.0
4.5
4.0
3.5
3.0
2.5
2.0
1.5
1.0
0.5
0.0
-0.
f1 (ppm)

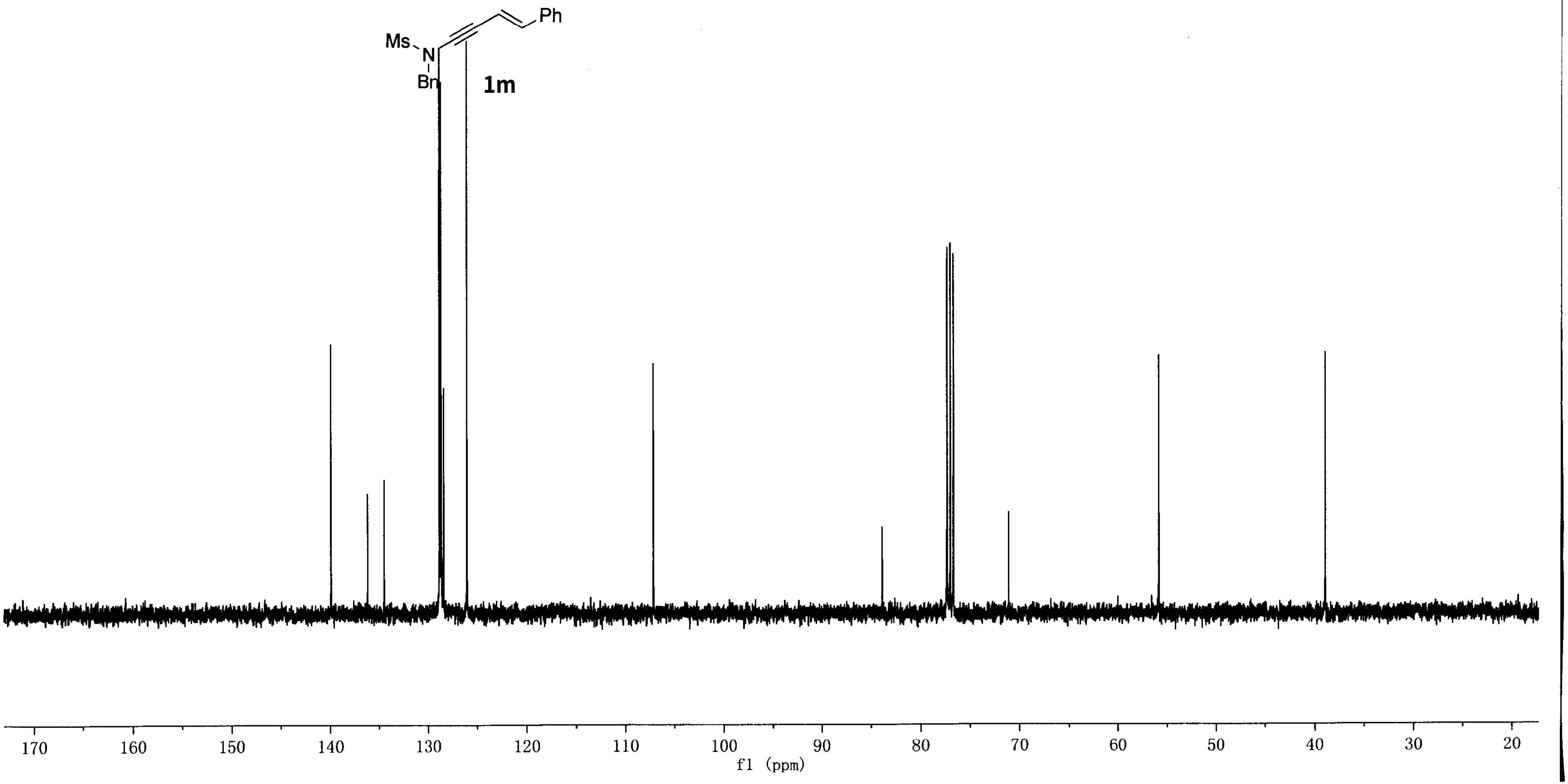
Ms
N
Bn
Ph
1m
170 160 150 140 130 120 110 100 90 80 70 60 50 40 30 20
f1 (ppm)

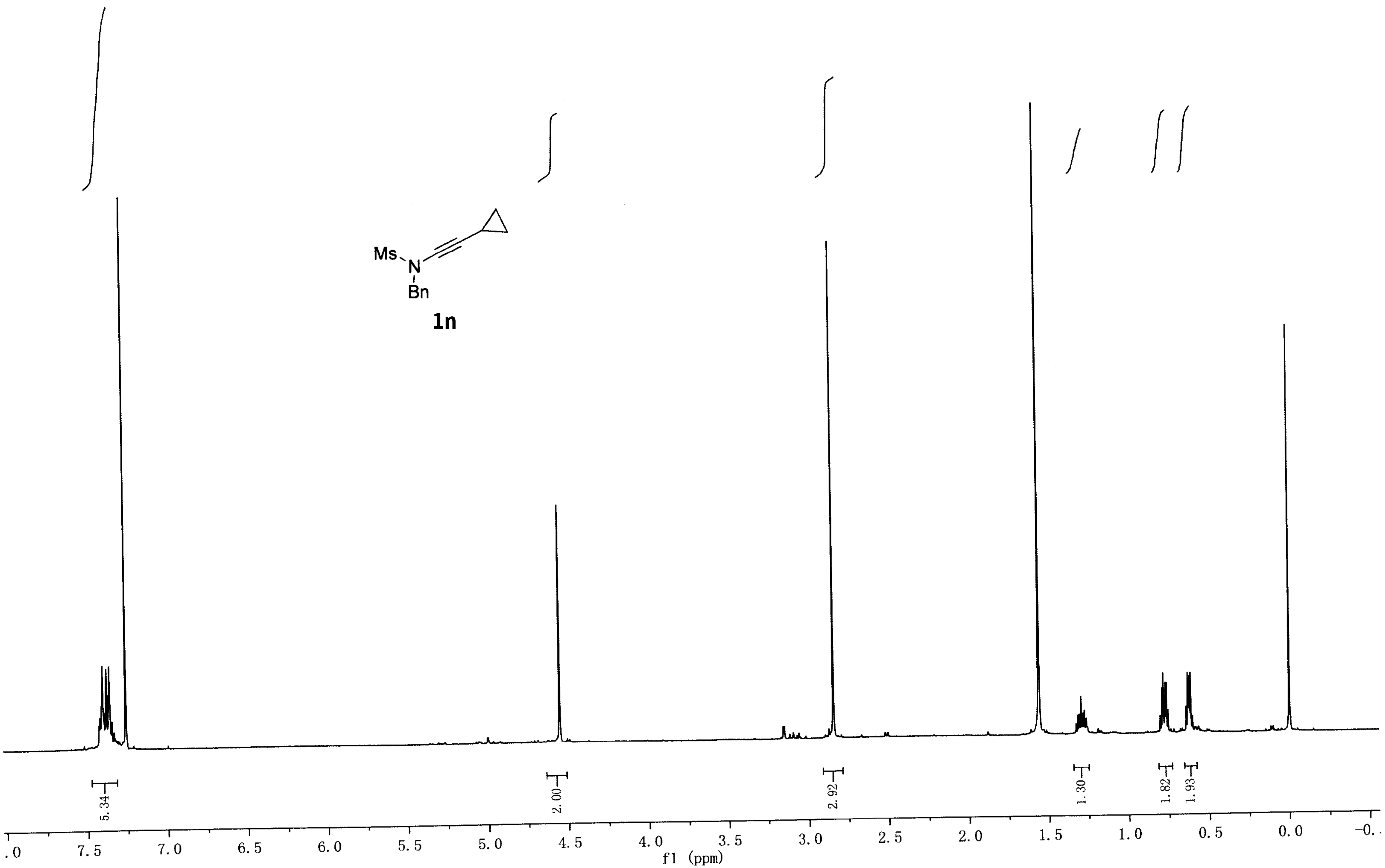
Ms
N
Bn
1n
5.34
2.00
2.92
1.30
1.82
1.93
.0
7.5
7.0
6.5
6.0
5.5
5.0
4.5
4.0
3.5
3.0
2.5
2.0
1.5
1.0
0.5
0.0
-0.
f1 (ppm)

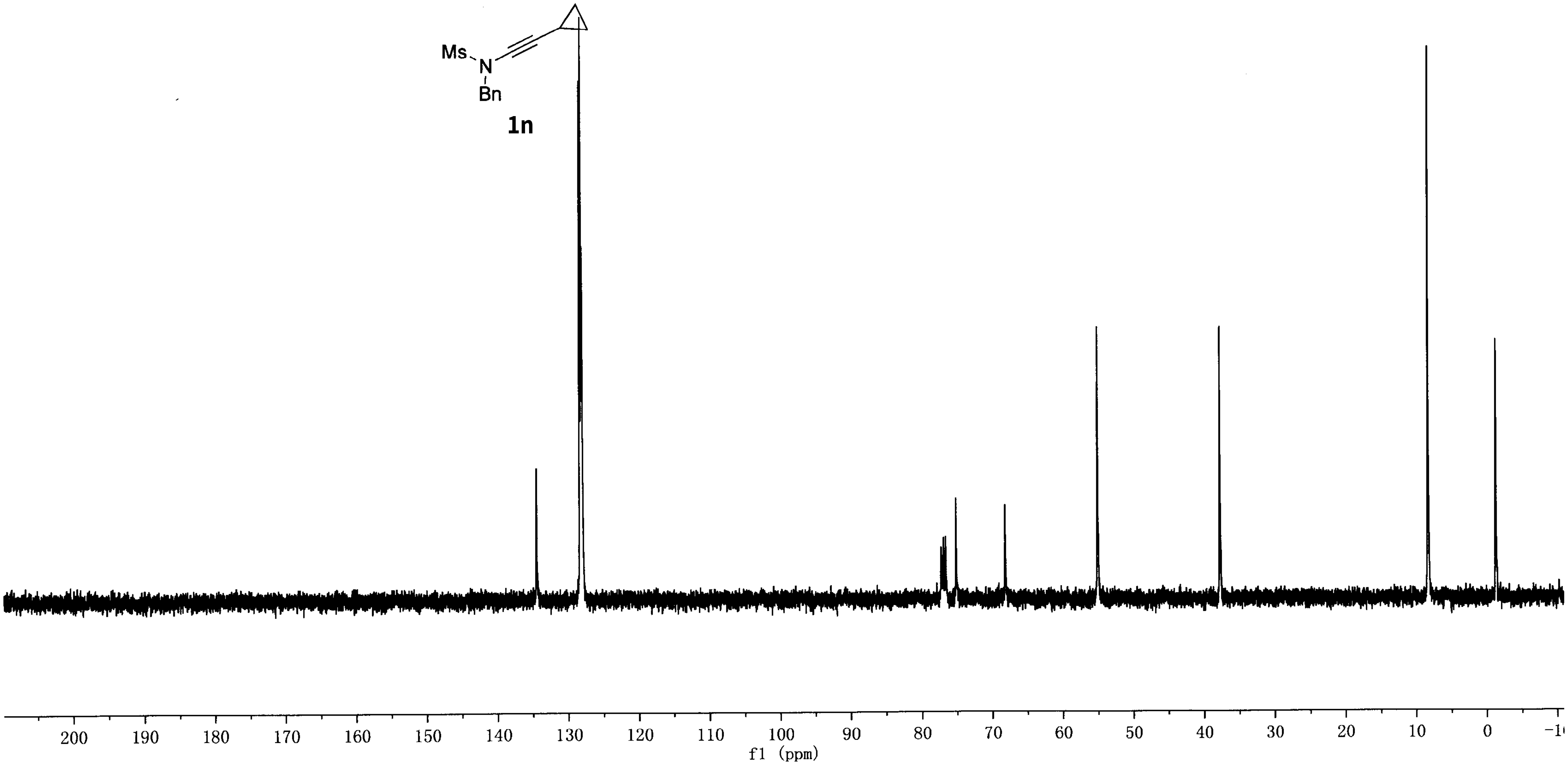

Ms
N
Bn
1n
200
190
180
170
160
150
140
130
120
110
100
90
80
70
60
50
40
30
20
10
0
f1 (ppm)

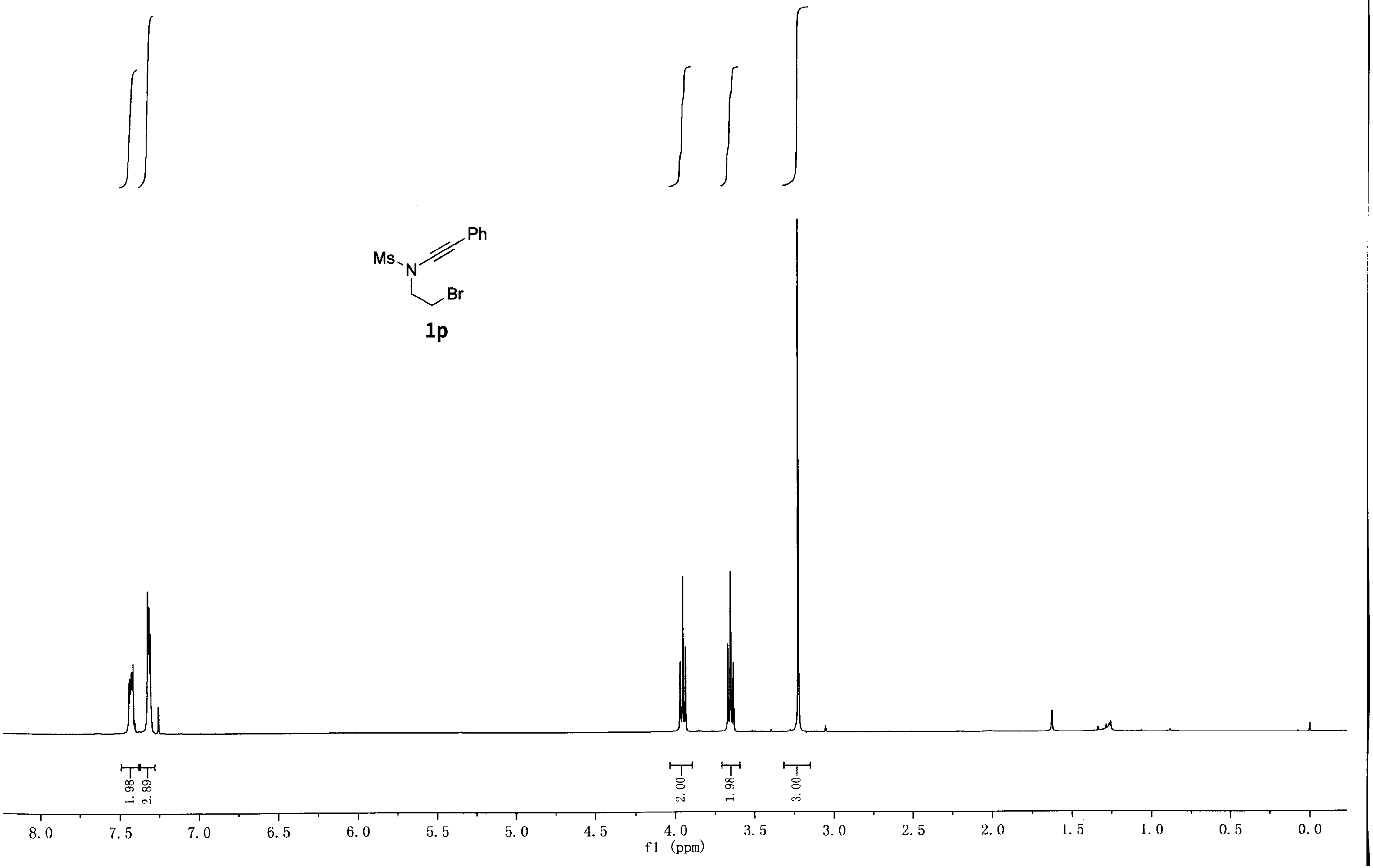
Ph
Ms
N
Br
1p
1.98
2.89
2.00
1.98
3.00
8.0
7.5
7.0
6.5
6.0
5.5
5.0
4.5
4.0
3.5
3.0
2.5
2.0
1.5
1.0
0.5
0.0
f1 (ppm)

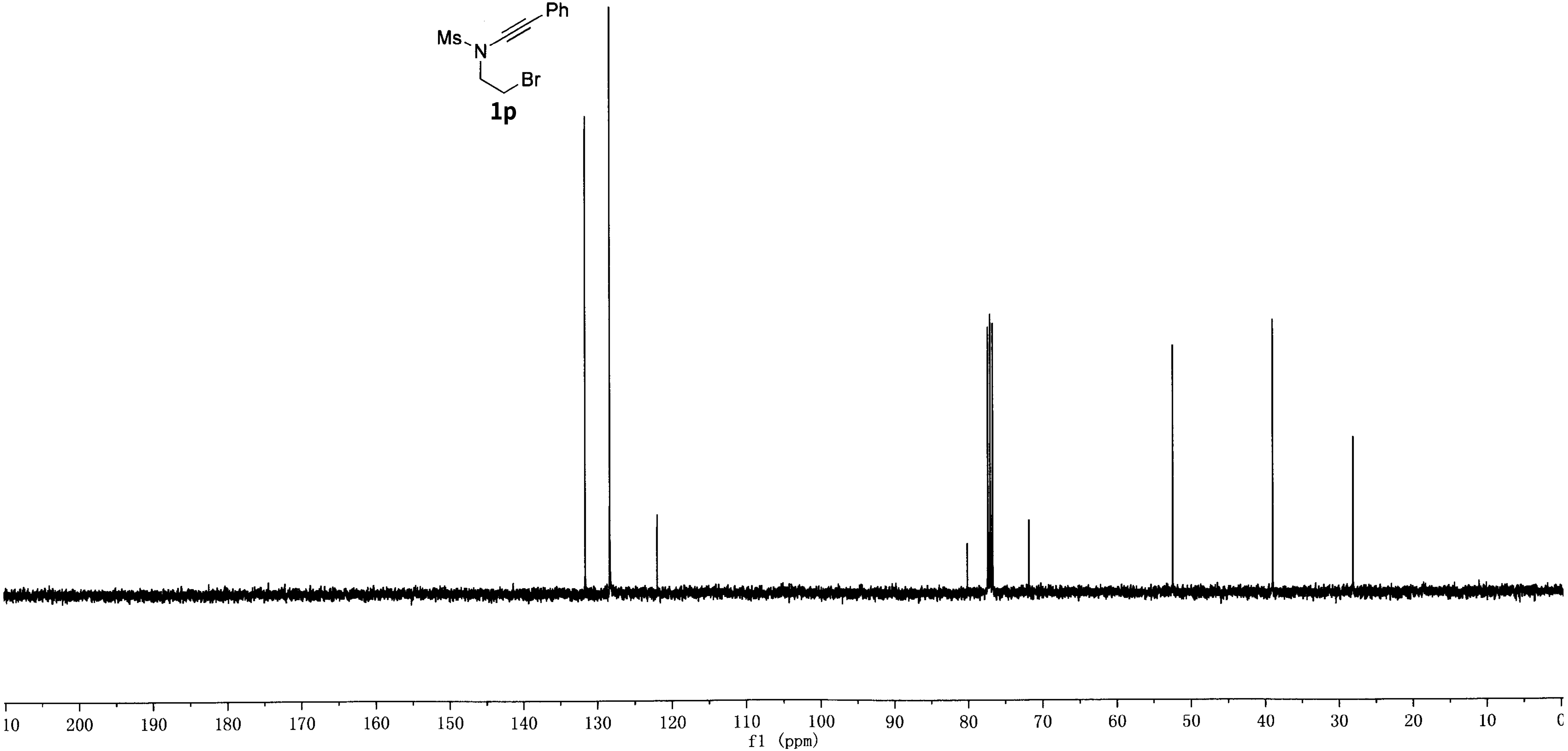

Ph
Ms
N
Br
1p
10
200
190
180
170
160
150
140
130
120
110
100
90
80
70
60
50
40
30
20
10
f1 (ppm)

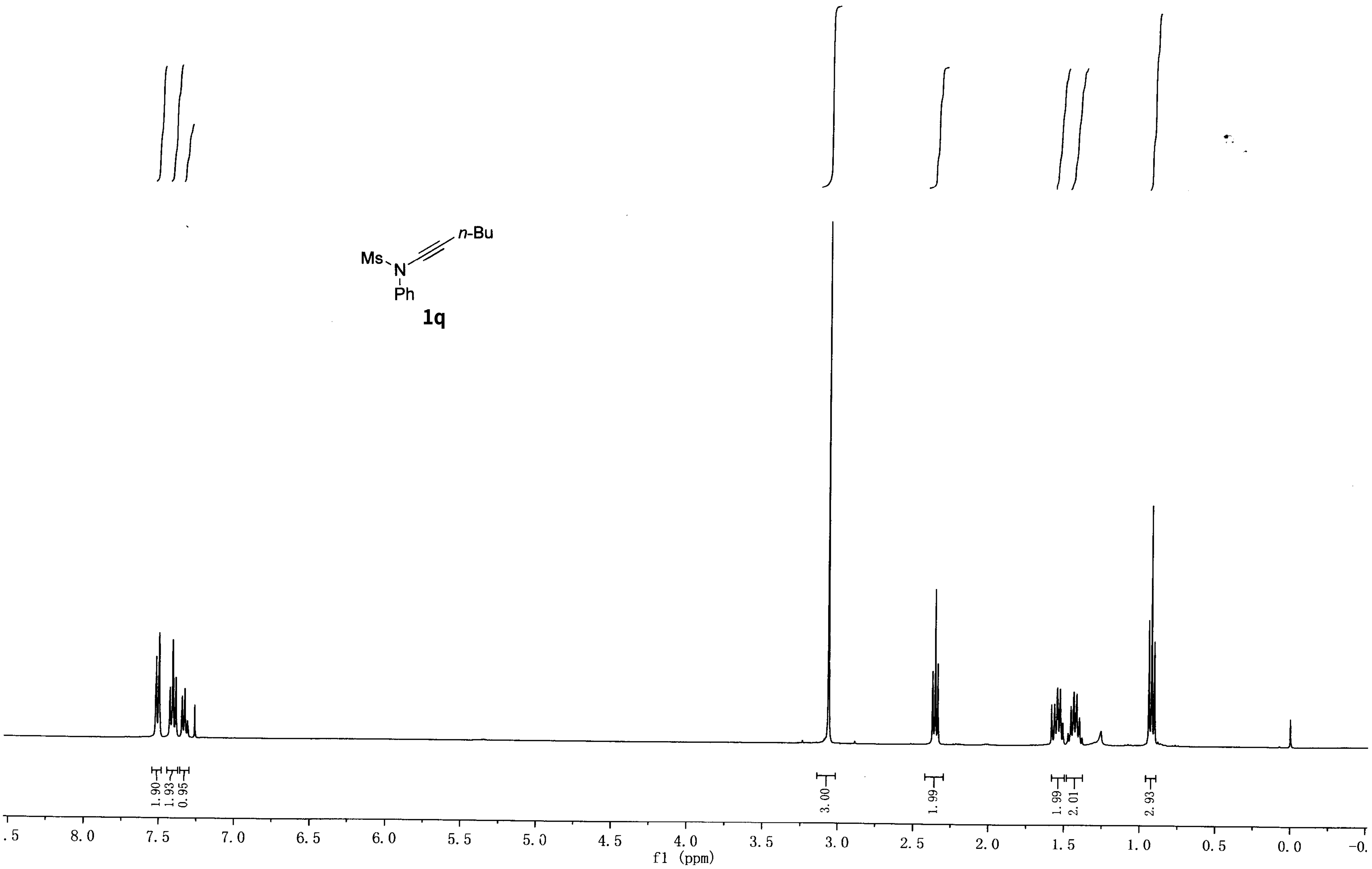

n-Bu
Ms
N
Ph
1q
1.90
1.93
0.95
3.00
1.99
1.99
2.01
2.93
.5
8.0
7.5
7.0
6.5
6.0
5.5
5.0
4.5
4.0
3.5
3.0
2.5
2.0
1.5
1.0
0.5
0.0
-0.
f1 (ppm)

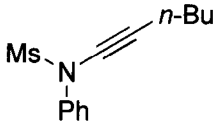
Ms
N
Ph
n-Bu

1q

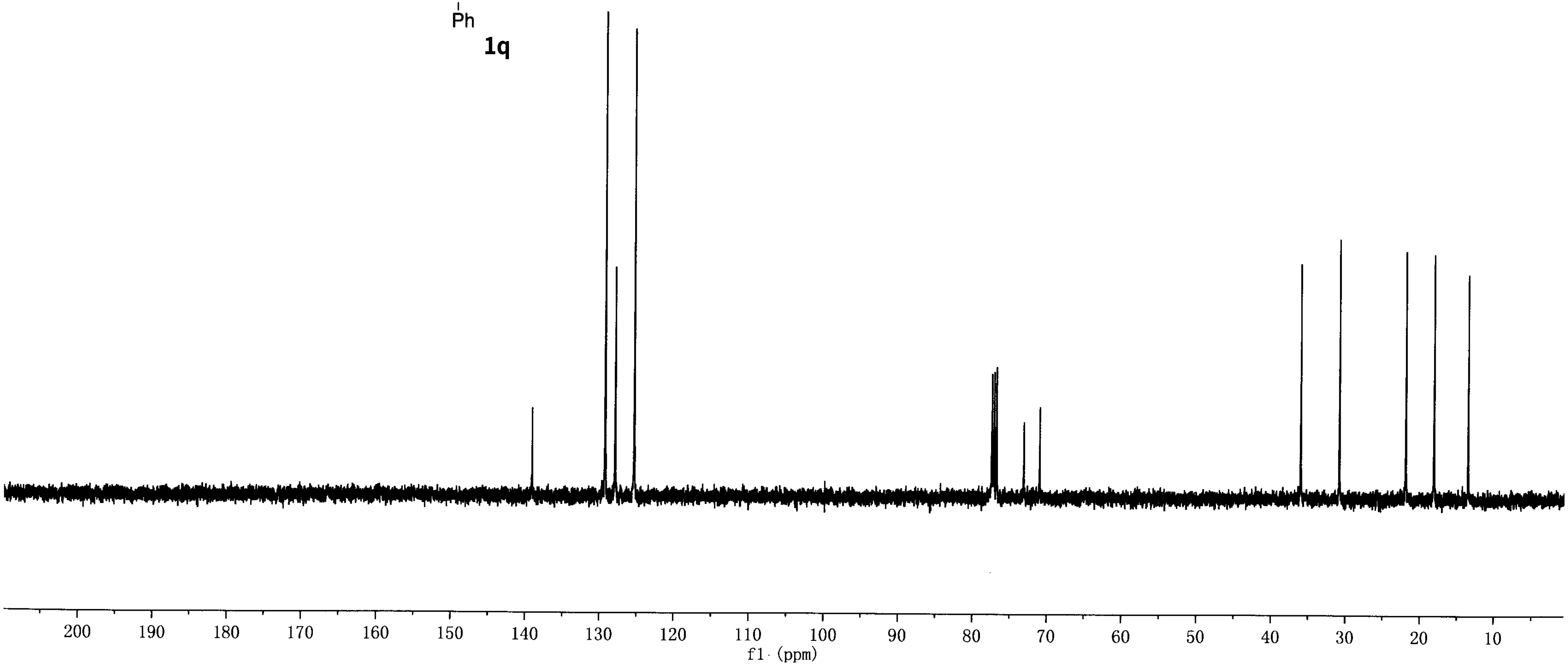
200 190 180 170 160 150 140 130 120 110 100 90 80 70 60 50 40 30 20 10
f1 (ppm)

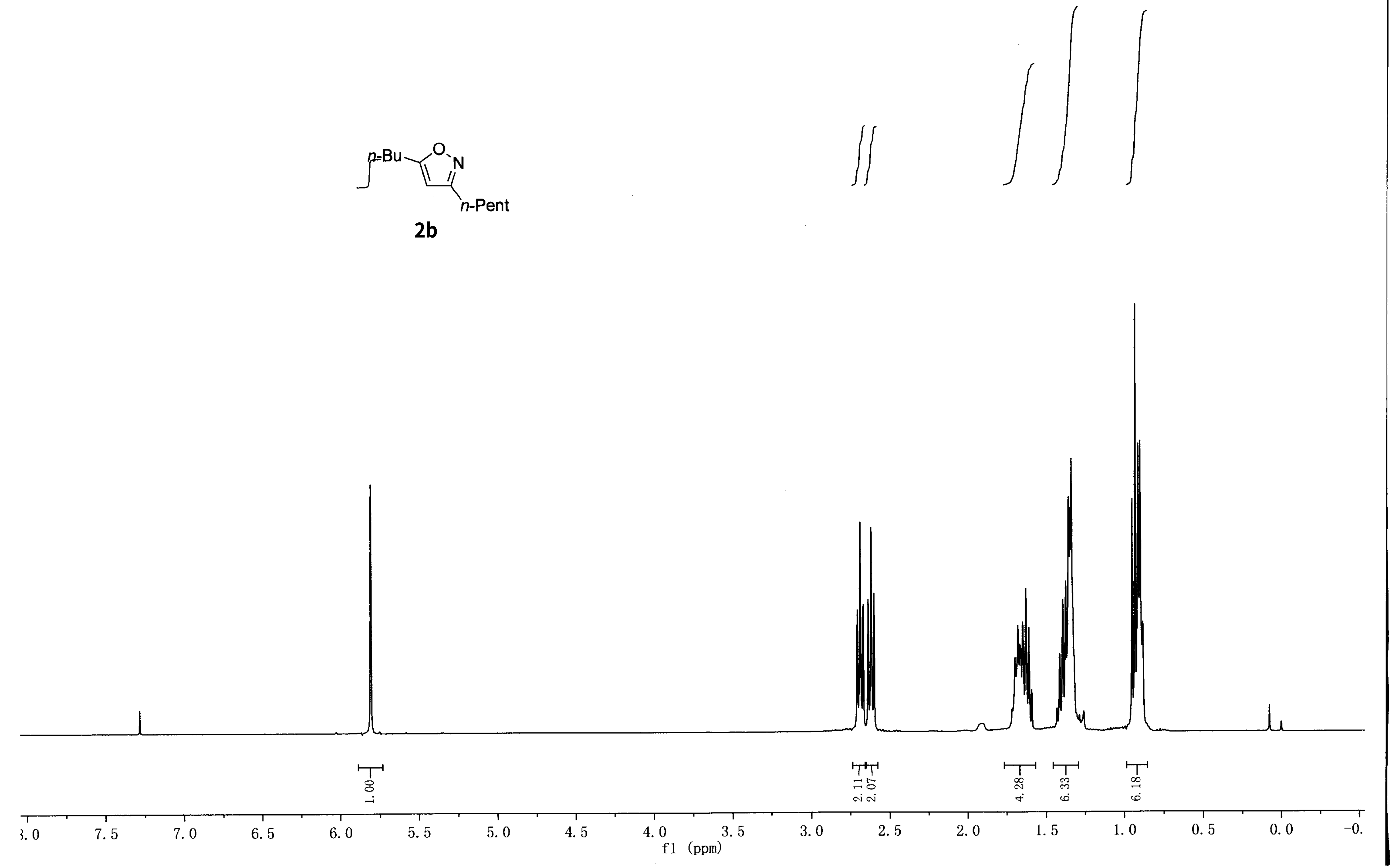
n-Bu
O
N
n-Pent
2b
1.00
2.11
2.07
4.28
6.33
6.18
8.0
7.5
7.0
6.5
6.0
5.5
5.0
4.5
4.0
3.5
3.0
2.5
2.0
1.5
1.0
0.5
0.0
-0.
f1 (ppm)

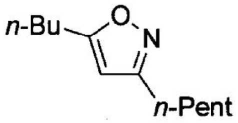

2b

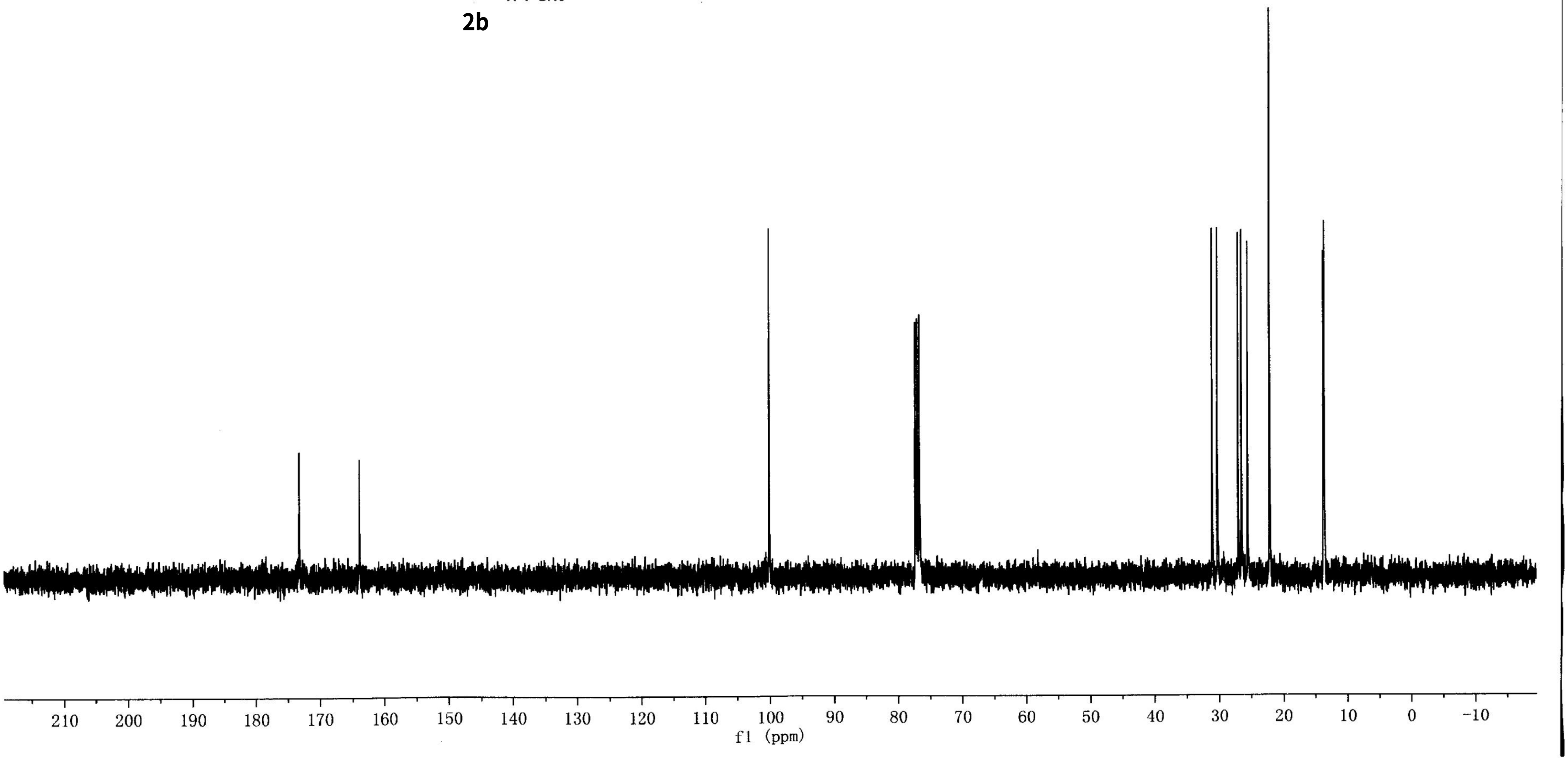
210
200
190
180
170
160
150
140
130
120
110
100
90
80
70
60
50
40
30
20
10
0
-10
f1 (ppm)

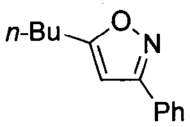

2c

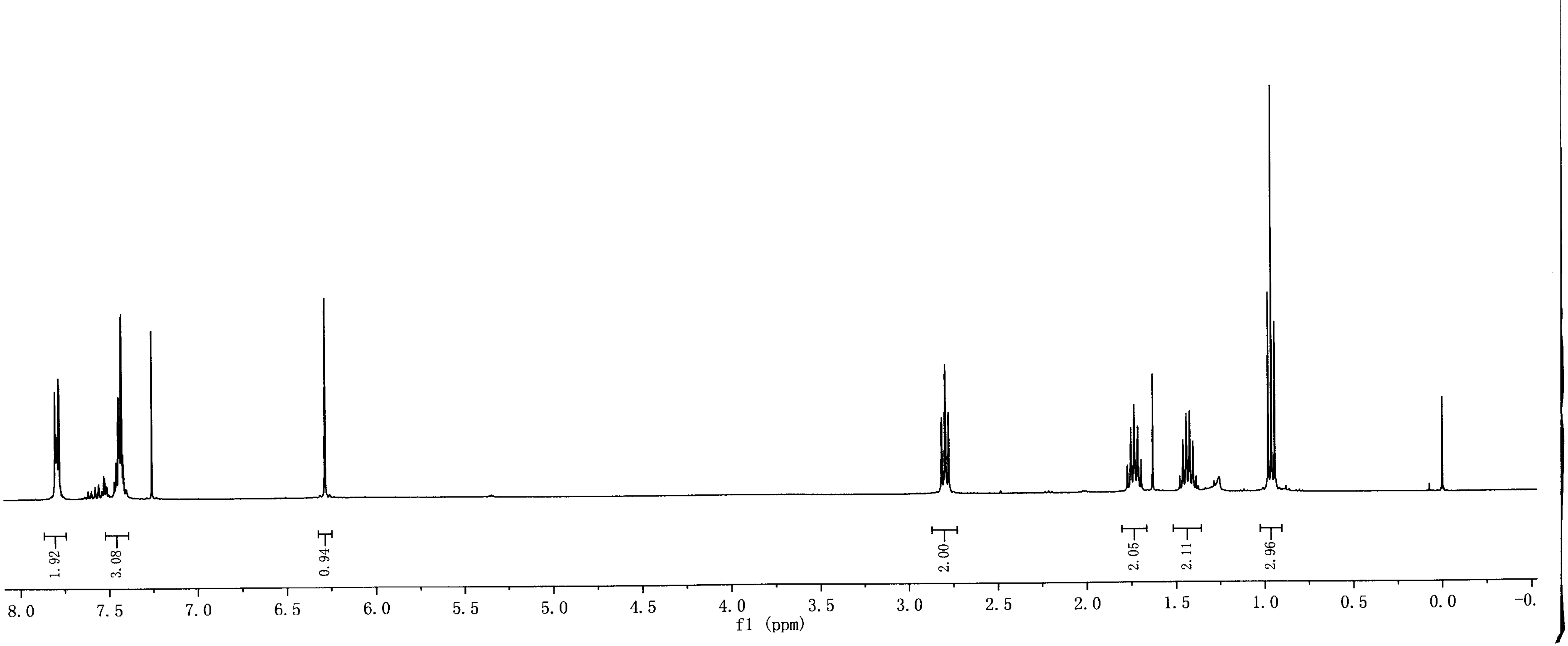

1.92
3.08
0.94
2.00
2.05
2.11
2.96
8.0
7.5
7.0
6.5
6.0
5.5
5.0
4.5
4.0
3.5
3.0
2.5
2.0
1.5
1.0
0.5
0.0
-0.
f1 (ppm)

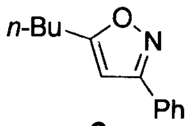
n-Bu
O
N
Ph

2c

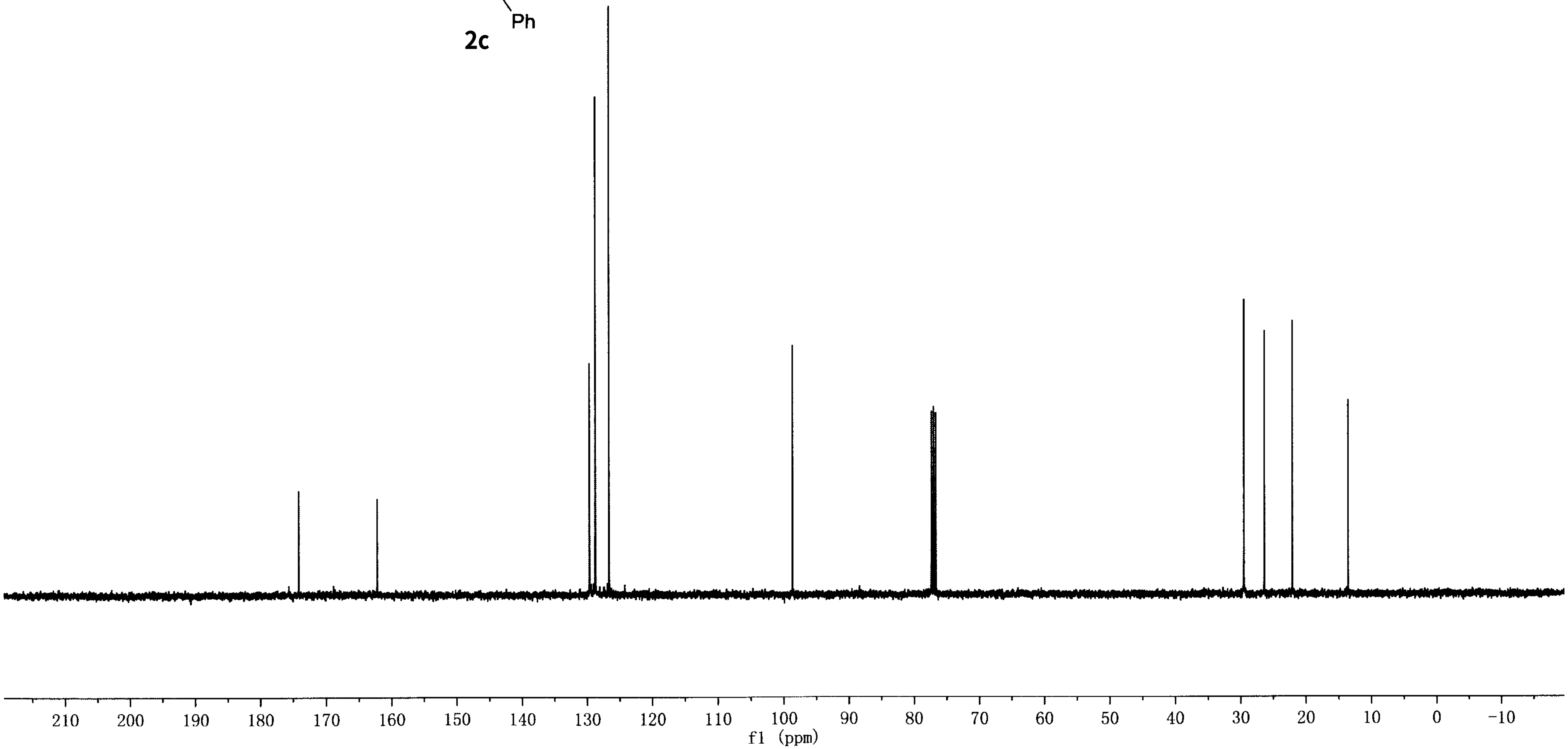
210 200 190 180 170 160 150 140 130 120 110 100 90 80 70 60 50 40 30 20 10 0 -10
f1 (ppm)

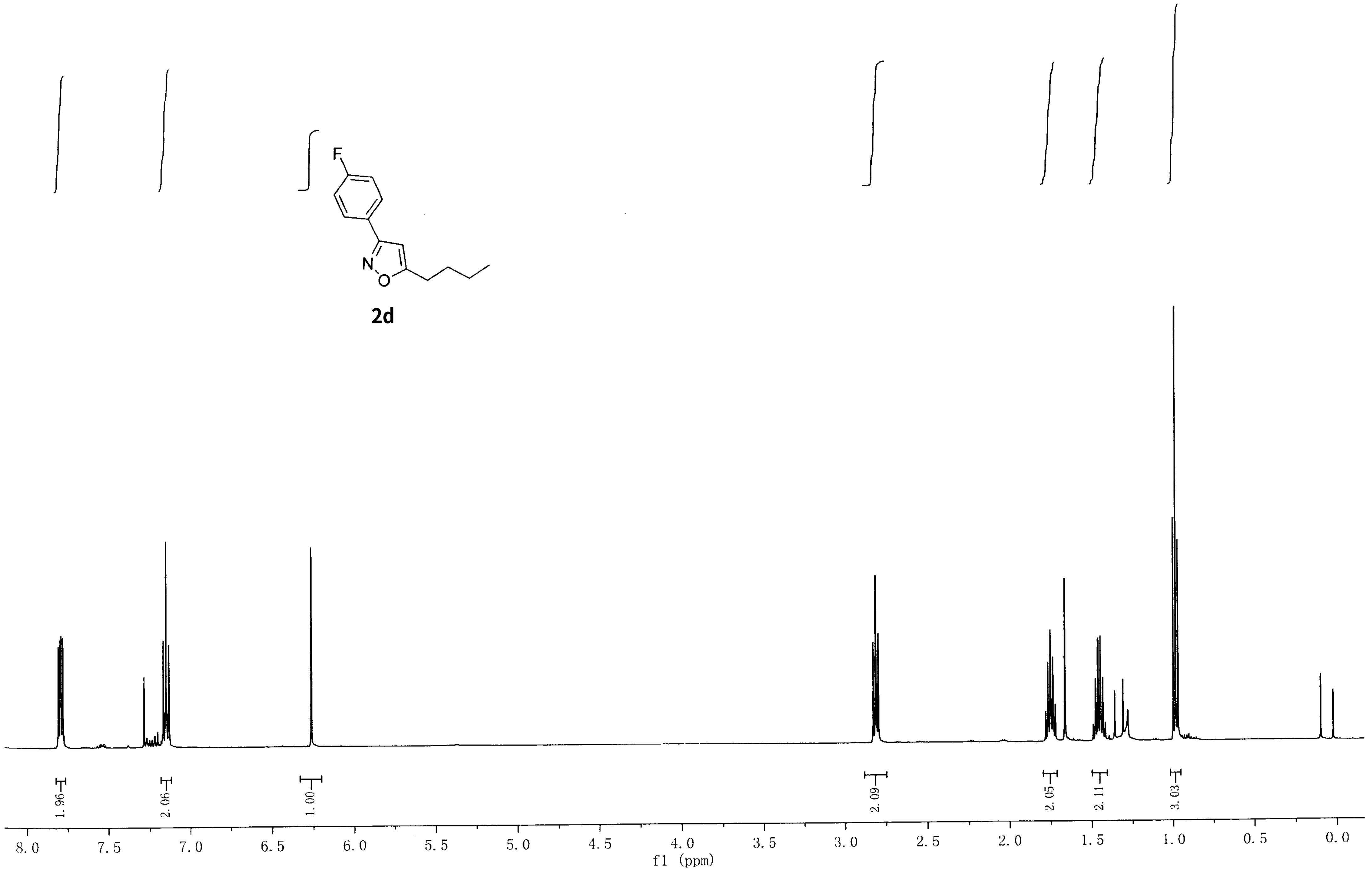

F
N
O
2d
1.96
2.06
1.00
2.09
2.05
2.11
3.03
8.0
7.5
7.0
6.5
6.0
5.5
5.0
4.5
4.0
3.5
3.0
2.5
2.0
1.5
1.0
0.5
0.0
f1 (ppm)

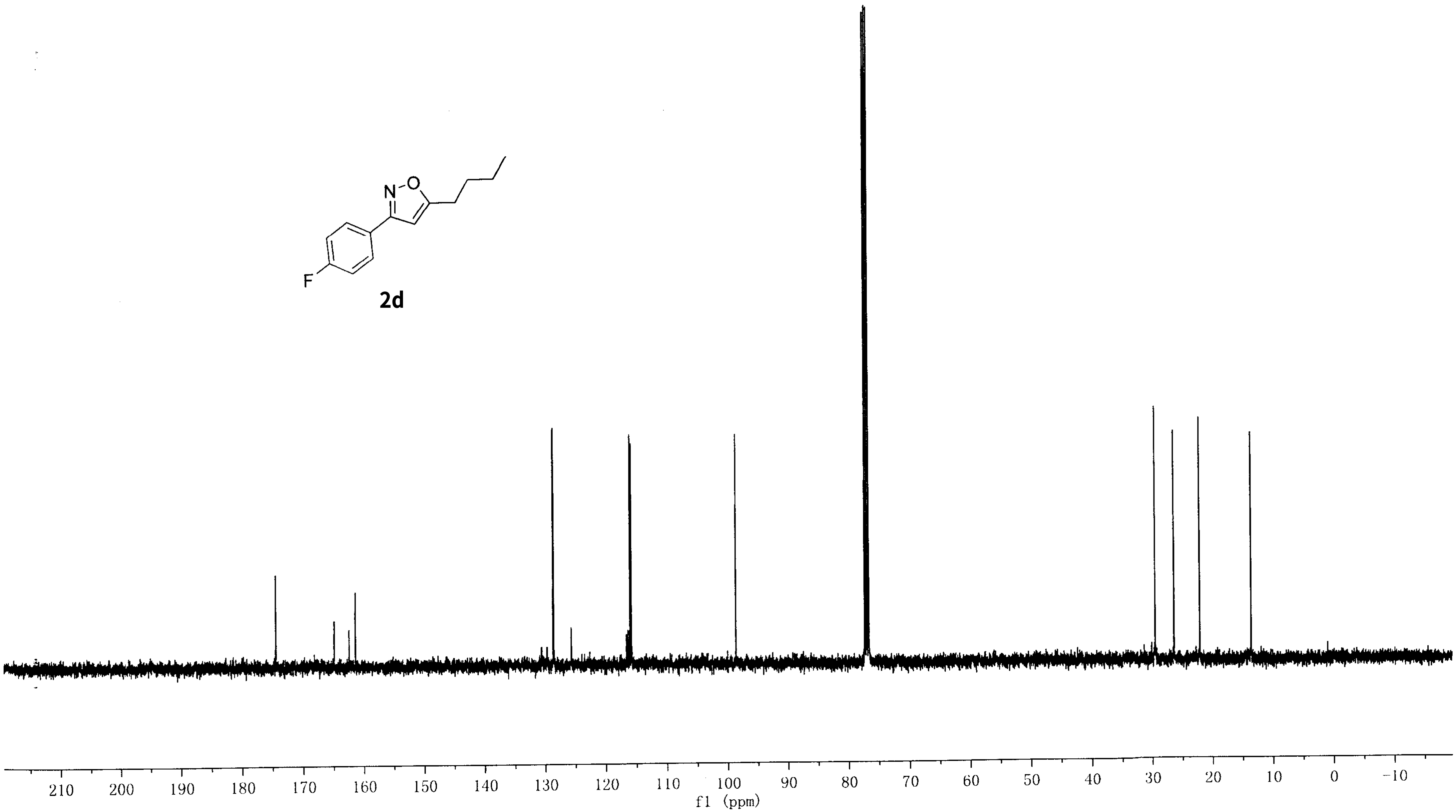
N
O
F
2d
210 200 190 180 170 160 150 140 130 120 110 100 90 80 70 60 50 40 30 20 10 0 −10
f1 (ppm)

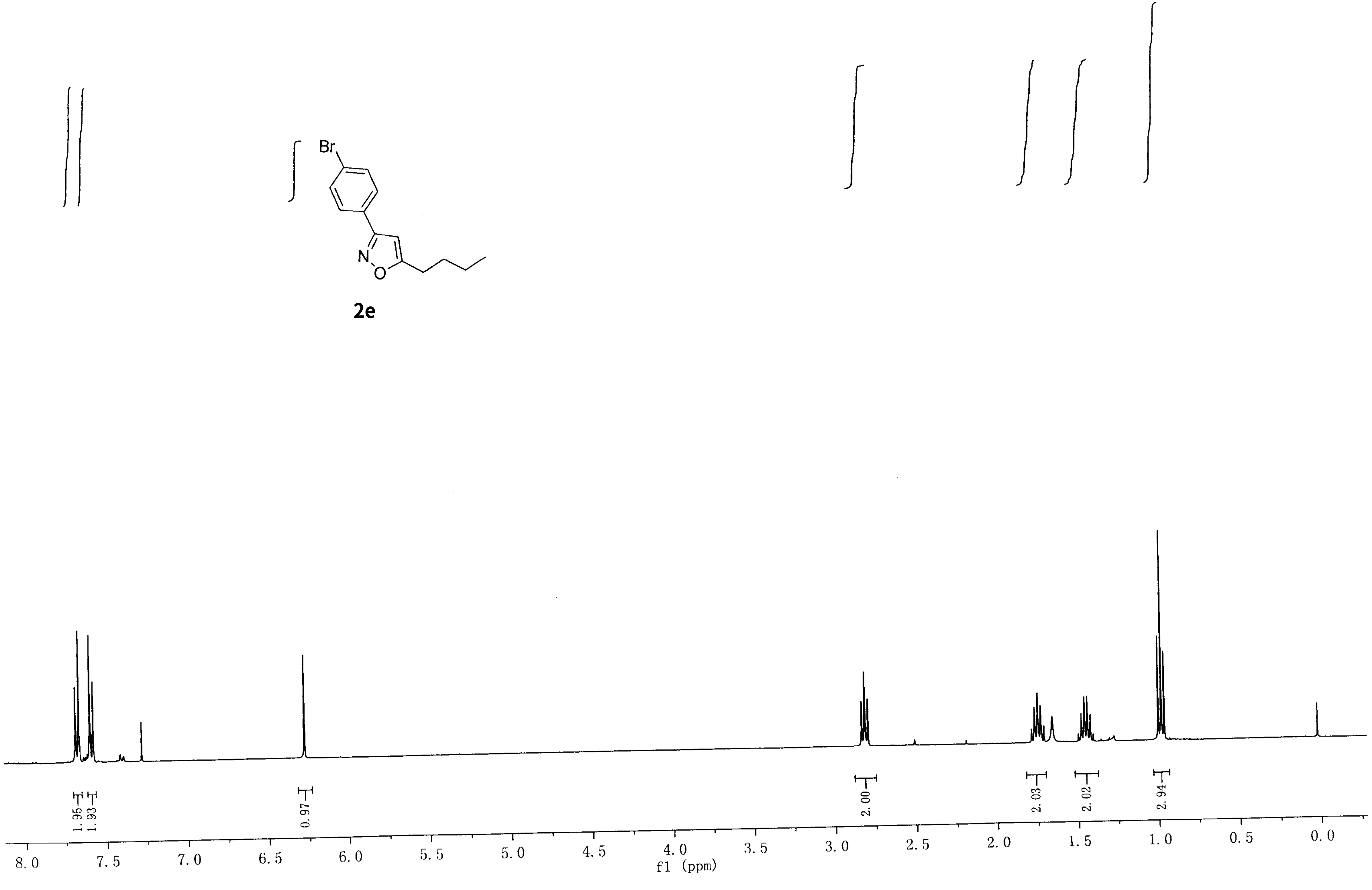

Br
N
O
1.95
1.93
0.97
2.00
2.03
2.02
2.94
8.0
7.5
7.0
6.5
6.0
5.5
5.0
4.5
4.0
3.5
3.0
2.5
2.0
1.5
1.0
0.5
0.0
f1 (ppm)

2e

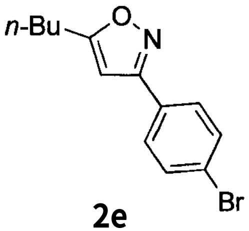
n-Bu
O
N
Br

2e

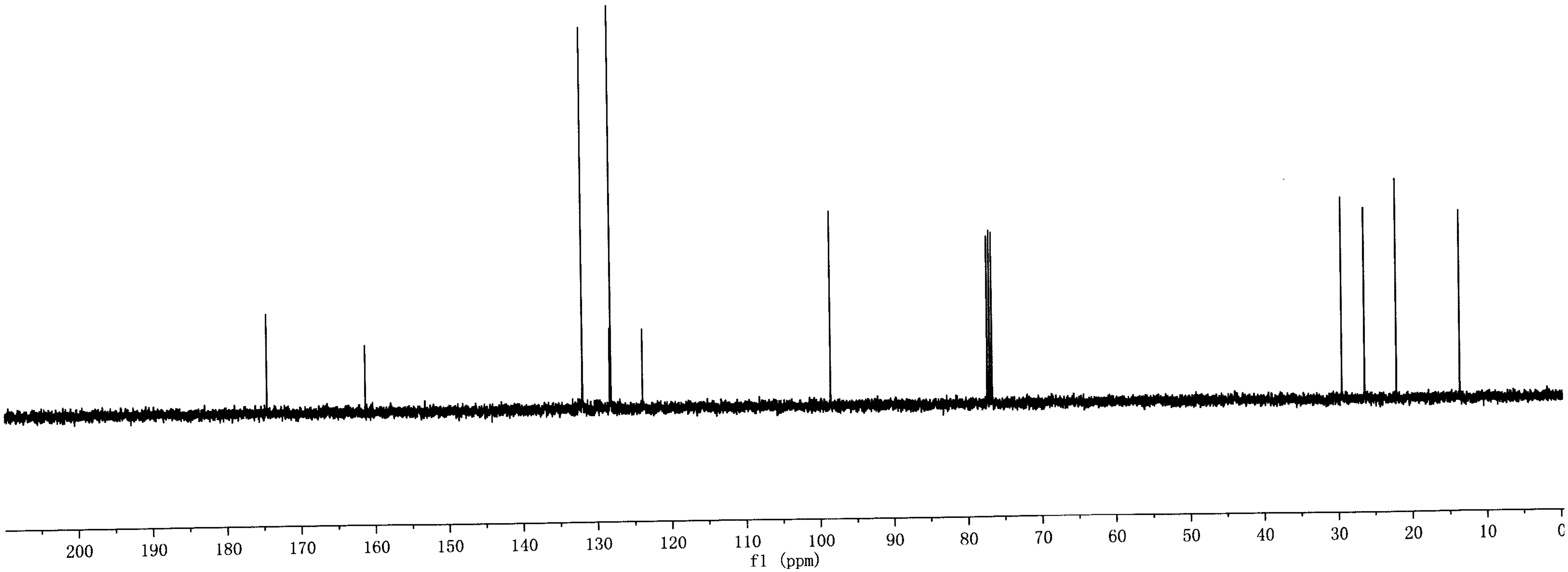
200 190 180 170 160 150 140 130 120 110 100 90 80 70 60 50 40 30 20 10 0
f1 (ppm)

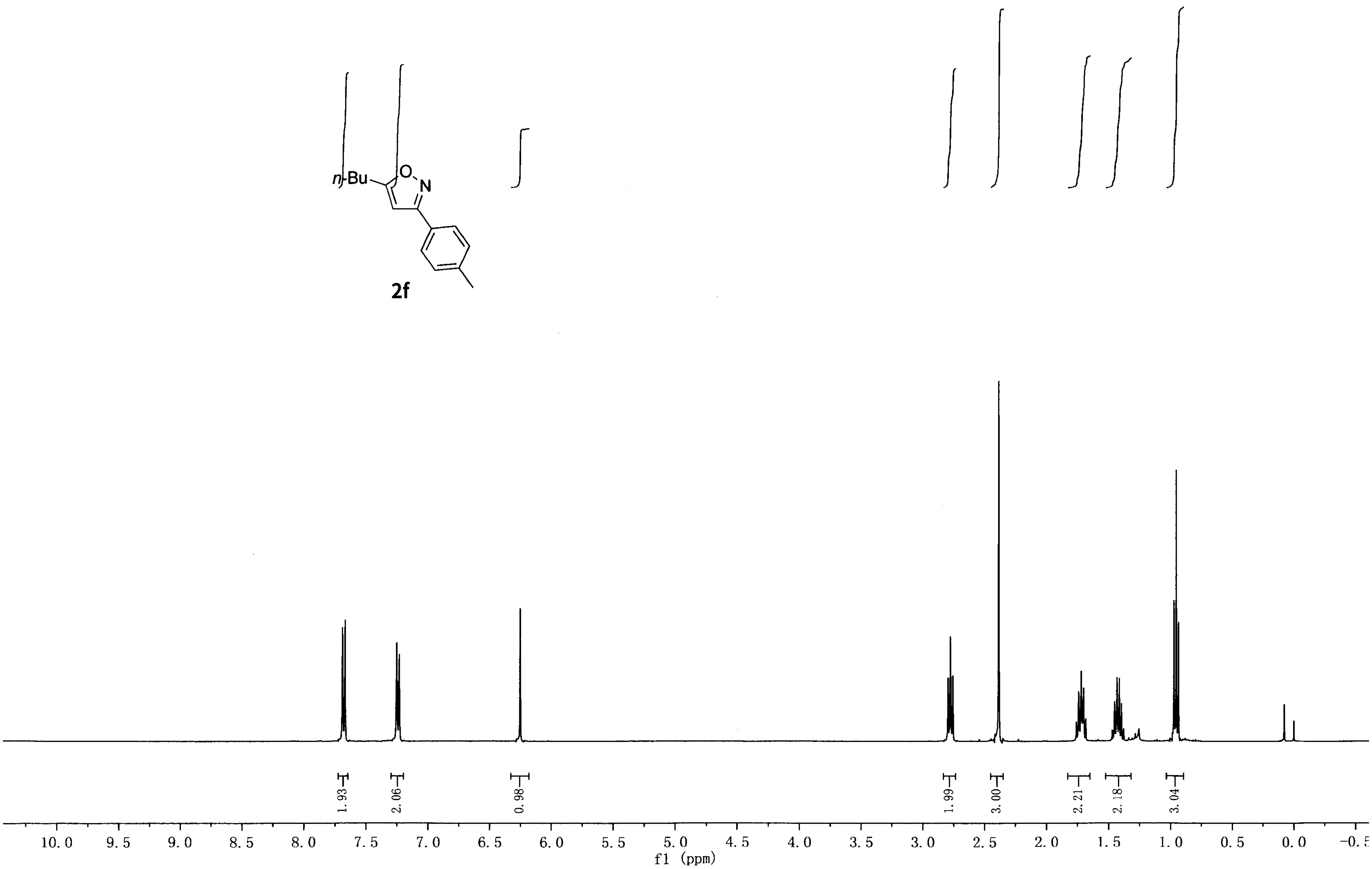

n-Bu
O
N
2f
1.93
2.06
0.98
1.99
3.00
2.21
2.18
3.04
10.0
9.5
9.0
8.5
8.0
7.5
7.0
6.5
6.0
5.5
5.0
4.5
4.0
3.5
3.0
2.5
2.0
1.5
1.0
0.5
0.0
f1 (ppm)

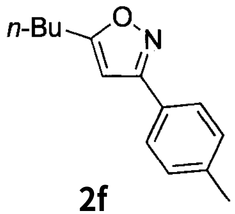
n-Bu
O
N

2f

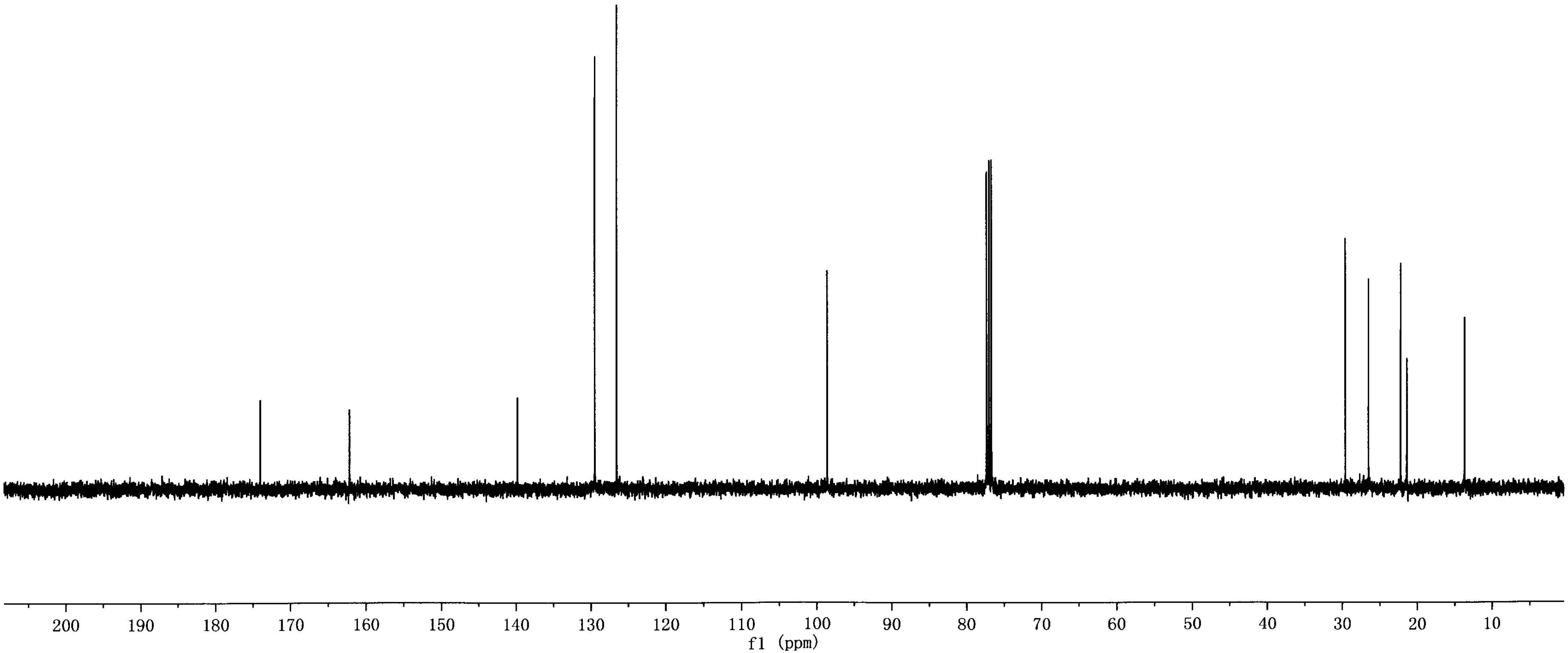
200
190
180
170
160
150
140
130
120
110
100
90
80
70
60
50
40
30
20
10
f1 (ppm)

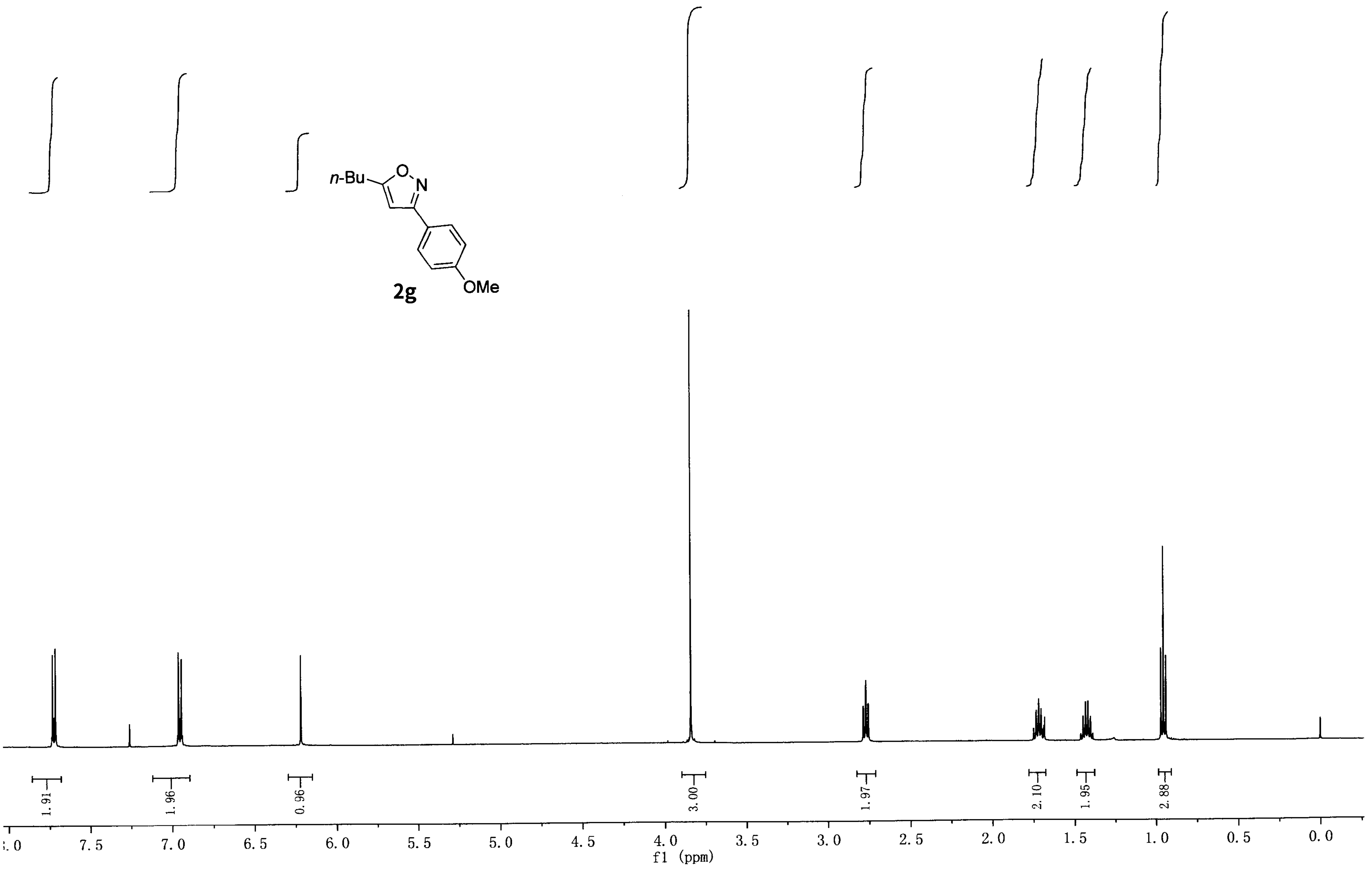

n-Bu
O
N
OMe
2g
1.91
1.96
0.96
3.00
1.97
2.10
1.95
2.88
7.5
7.0
6.5
6.0
5.5
5.0
4.5
4.0
3.5
3.0
2.5
2.0
1.5
1.0
0.5
0.0
f1 (ppm)

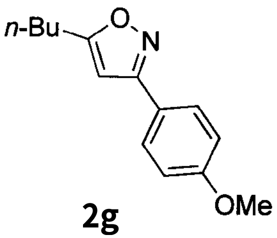
n-Bu
O
N
OMe

2g

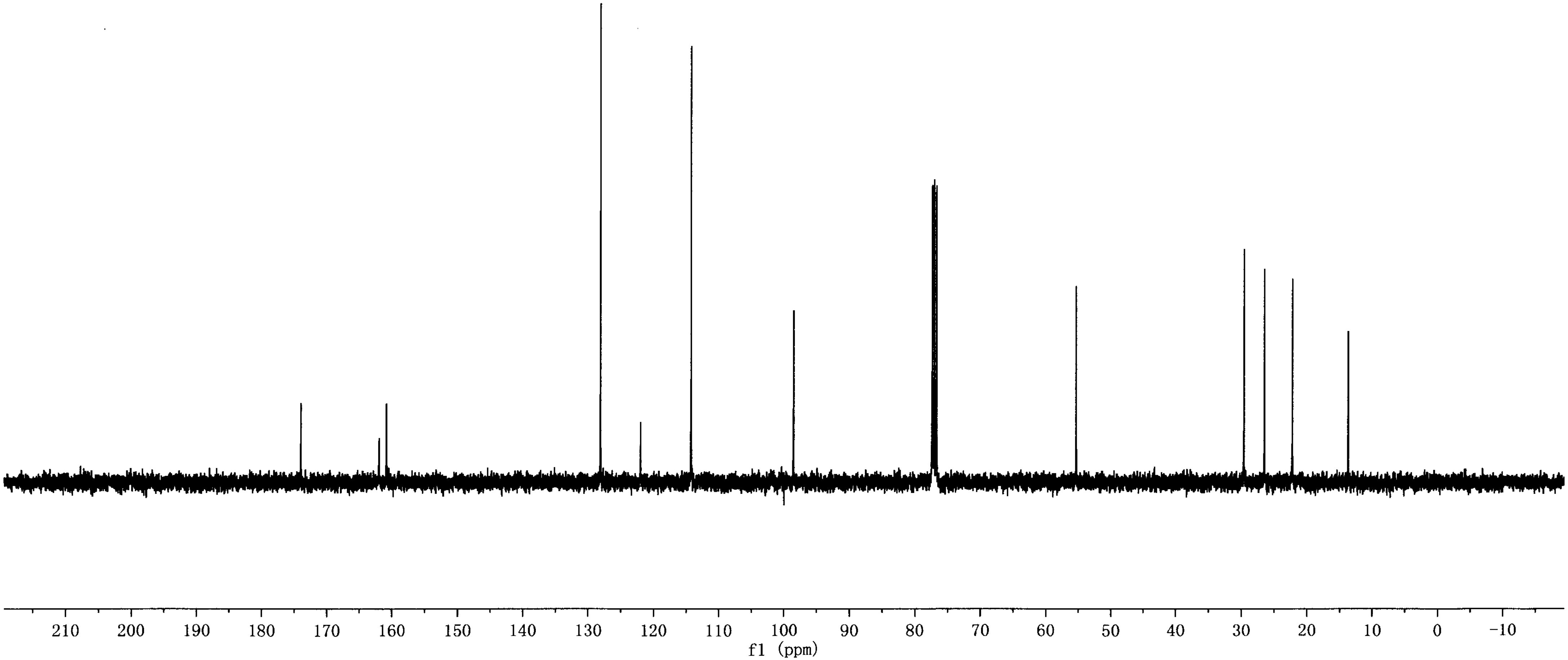
210 200 190 180 170 160 150 140 130 120 110 100 90 80 70 60 50 40 30 20 10 0 -10
f1 (ppm)

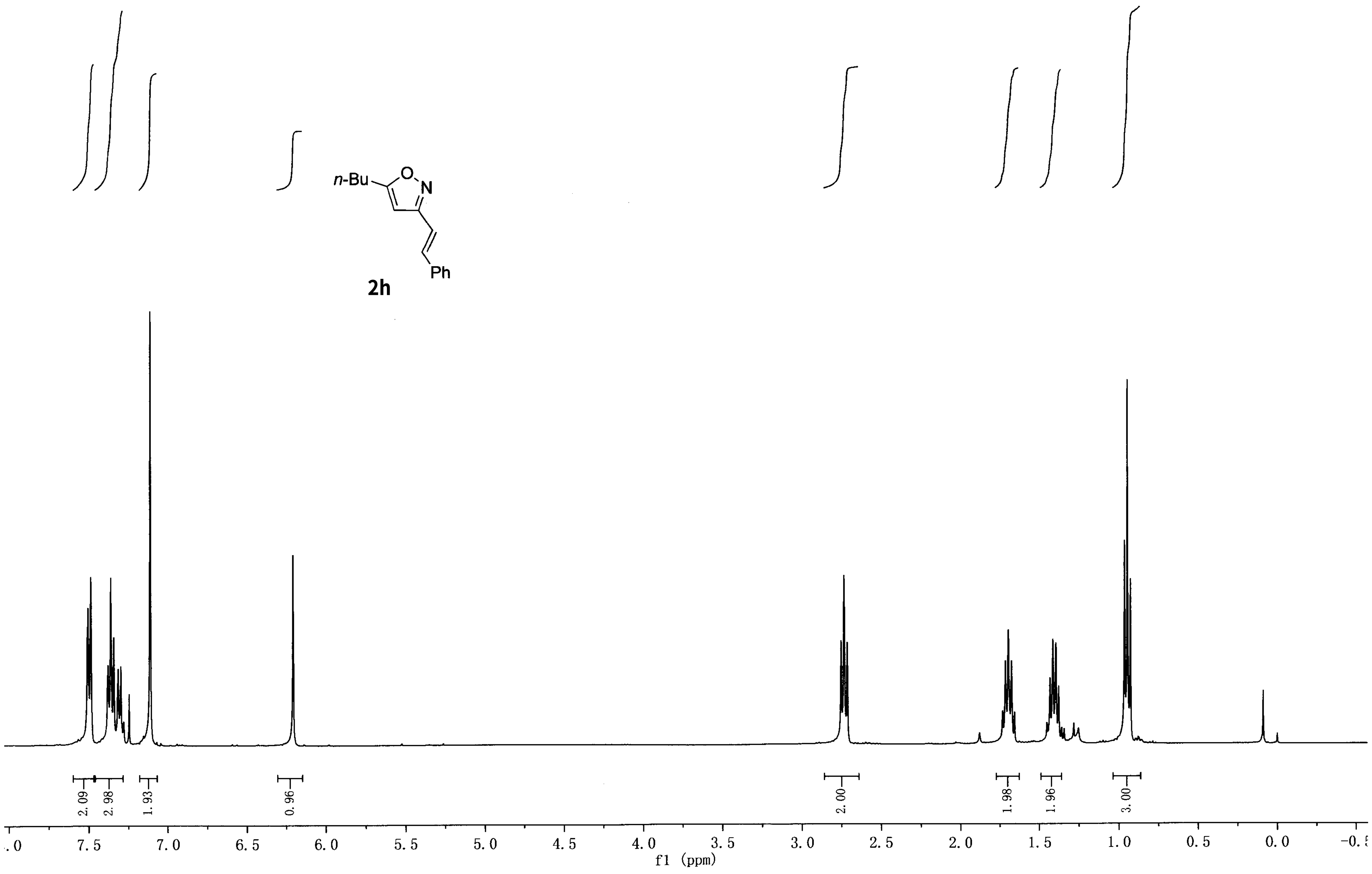
n-Bu
O
N
Ph
2h
2.09
2.98
1.93
0.96
2.00
1.98
1.96
3.00
7.5
7.0
6.5
6.0
5.5
5.0
4.5
4.0
3.5
3.0
2.5
2.0
1.5
1.0
0.5
0.0
f1 (ppm)

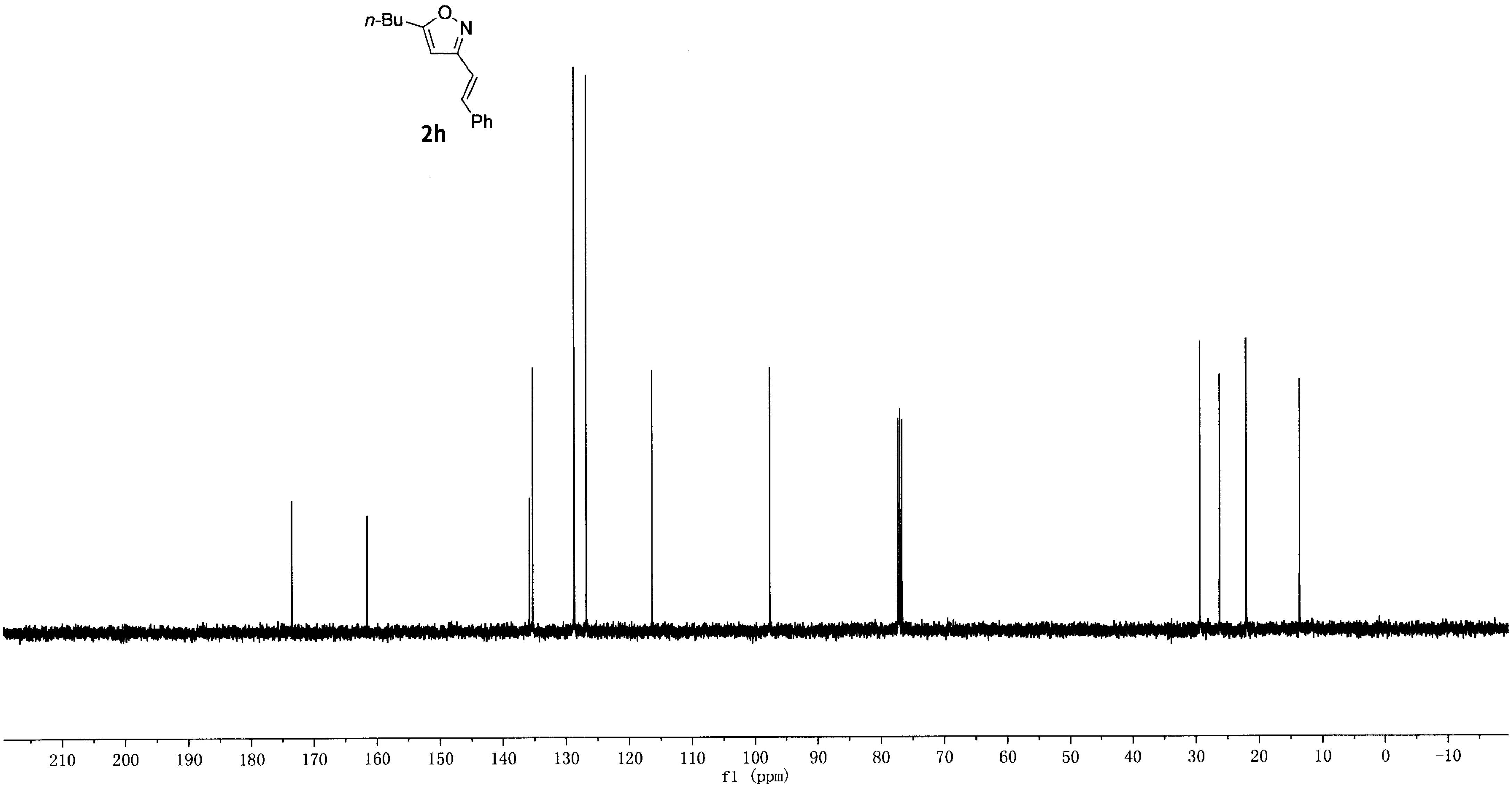
n-Bu
O
N
Ph
2h
210
200
190
180
170
160
150
140
130
120
110
100
90
80
70
60
50
40
30
20
10
0
-10
f1 (ppm)

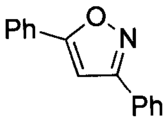

2i

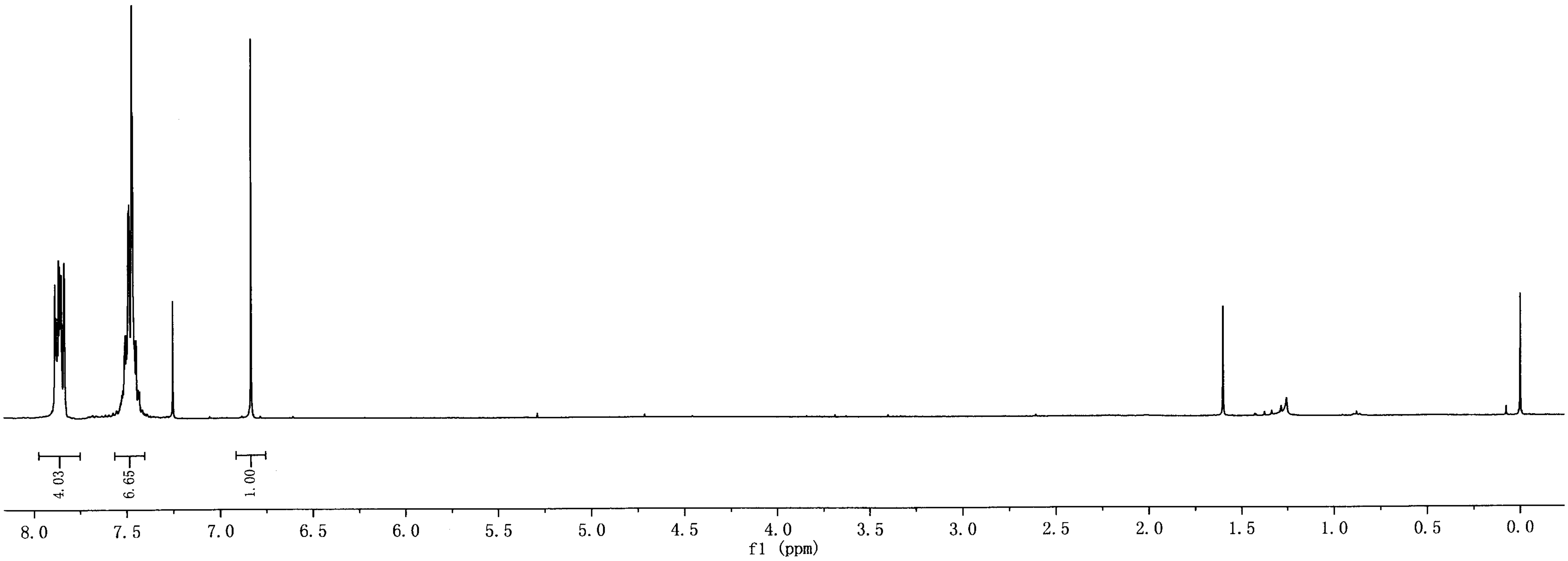

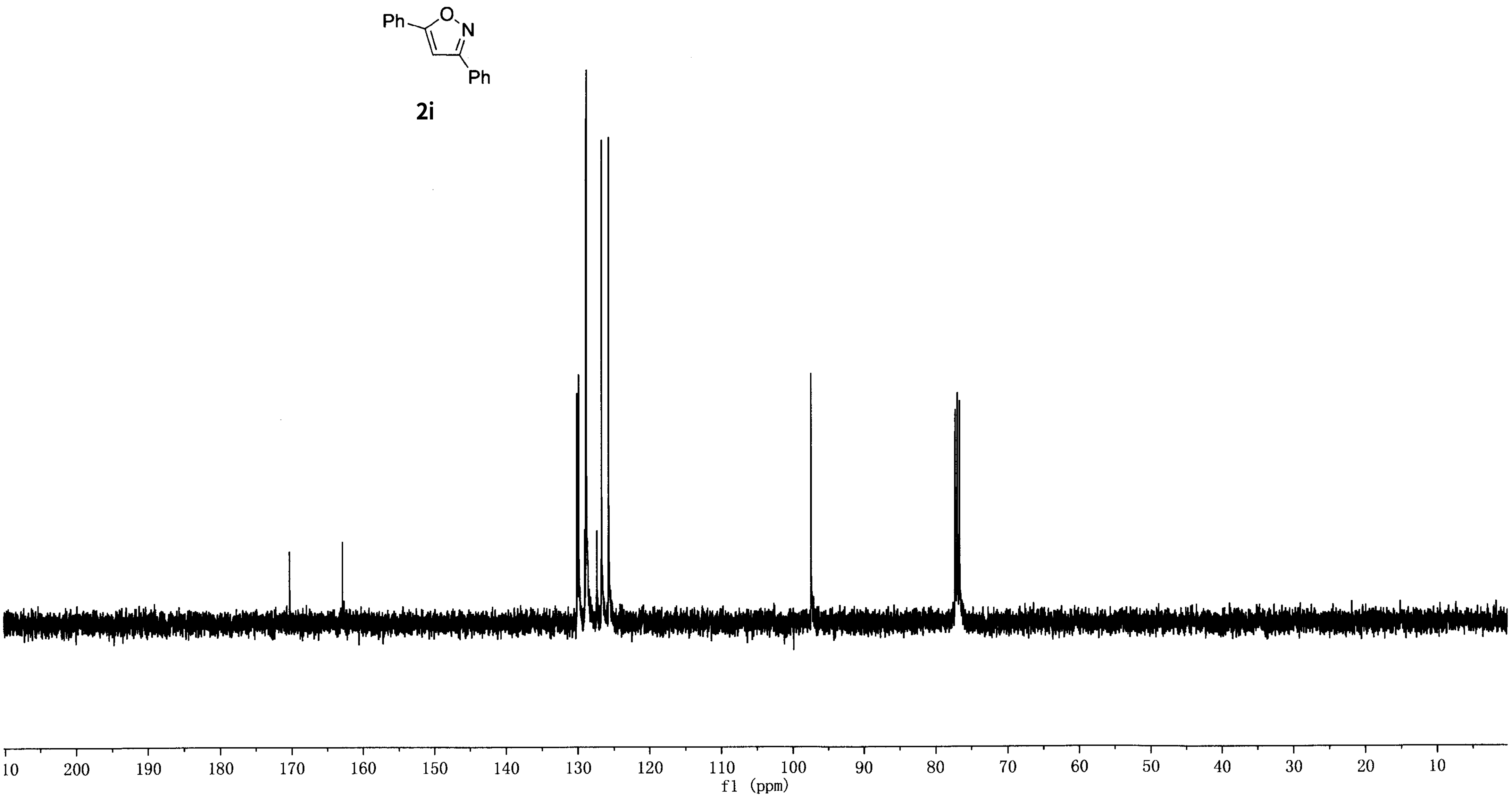

Ph
O
N
Ph
2i
10
200
190
180
170
160
150
140
130
120
110
100
90
80
70
60
50
40
30
20
10
f1 (ppm)

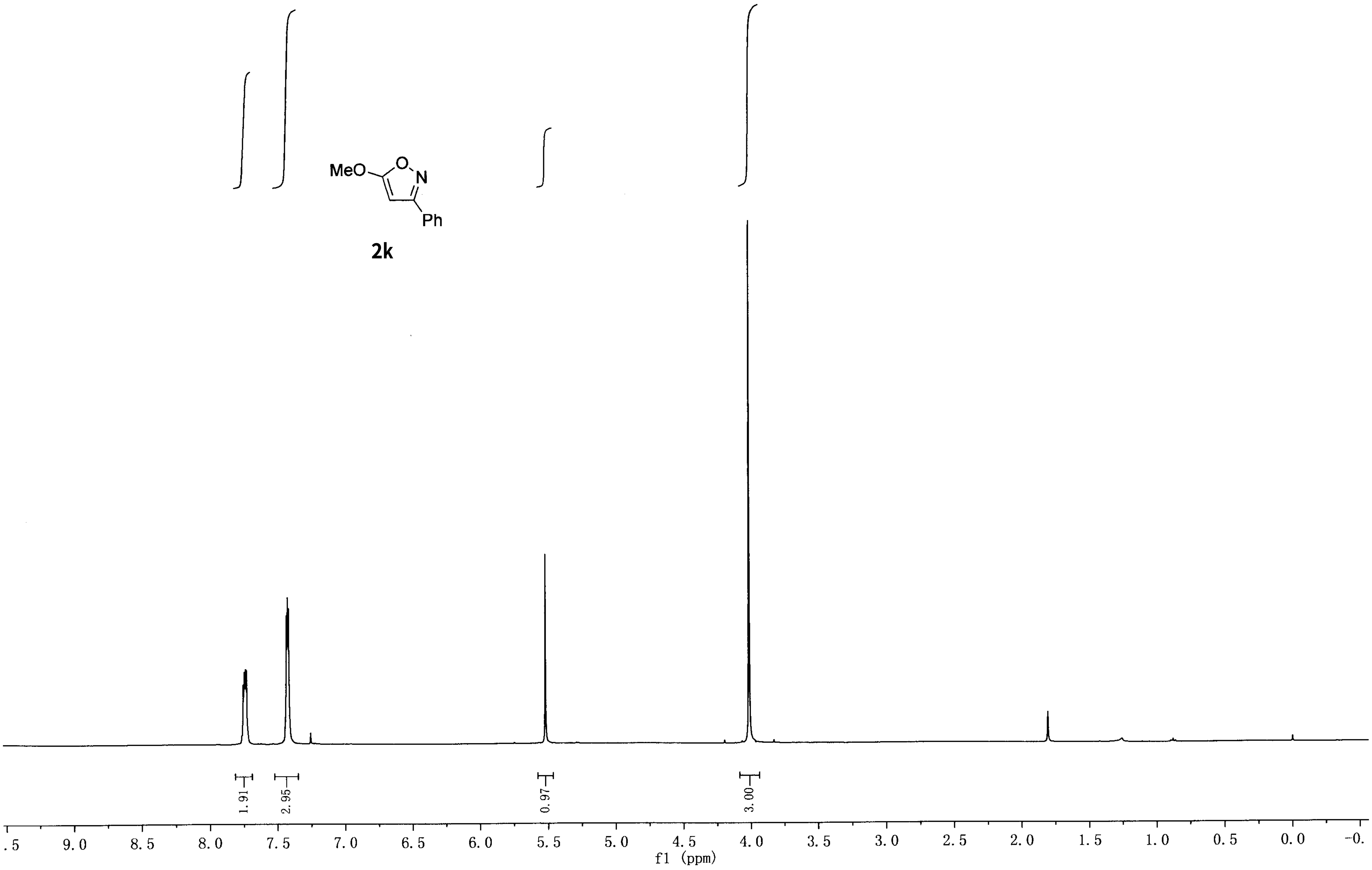
MeO
O
N
Ph
2k
1.91
2.95
0.97
3.00
.5
9.0
8.5
8.0
7.5
7.0
6.5
6.0
5.5
5.0
4.5
4.0
3.5
3.0
2.5
2.0
1.5
1.0
0.5
0.0
-0.
f1 (ppm)

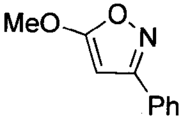
MeO
O
N
Ph

2k

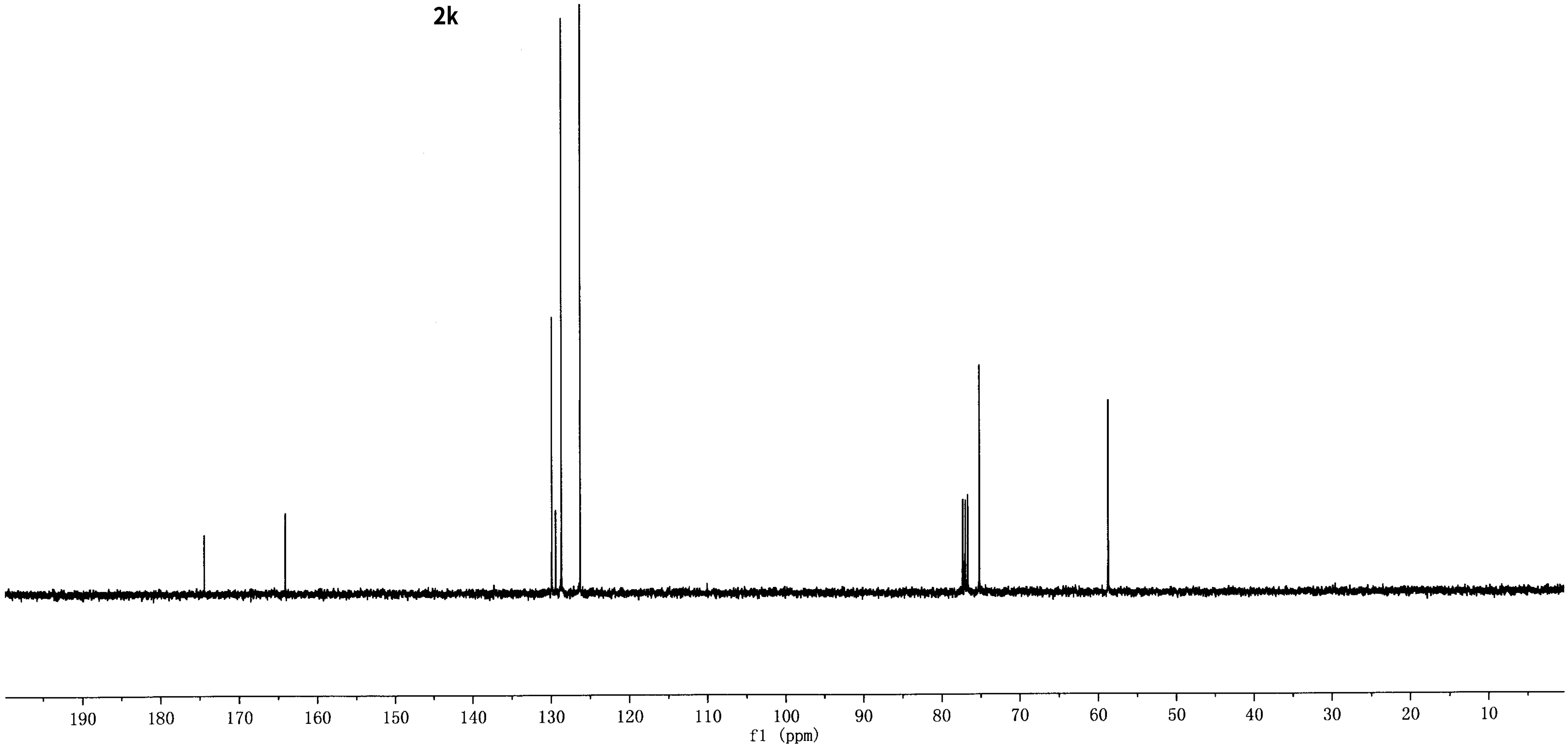
190
180
170
160
150
140
130
120
110
100
90
80
70
60
50
40
30
20
10
f1 (ppm)

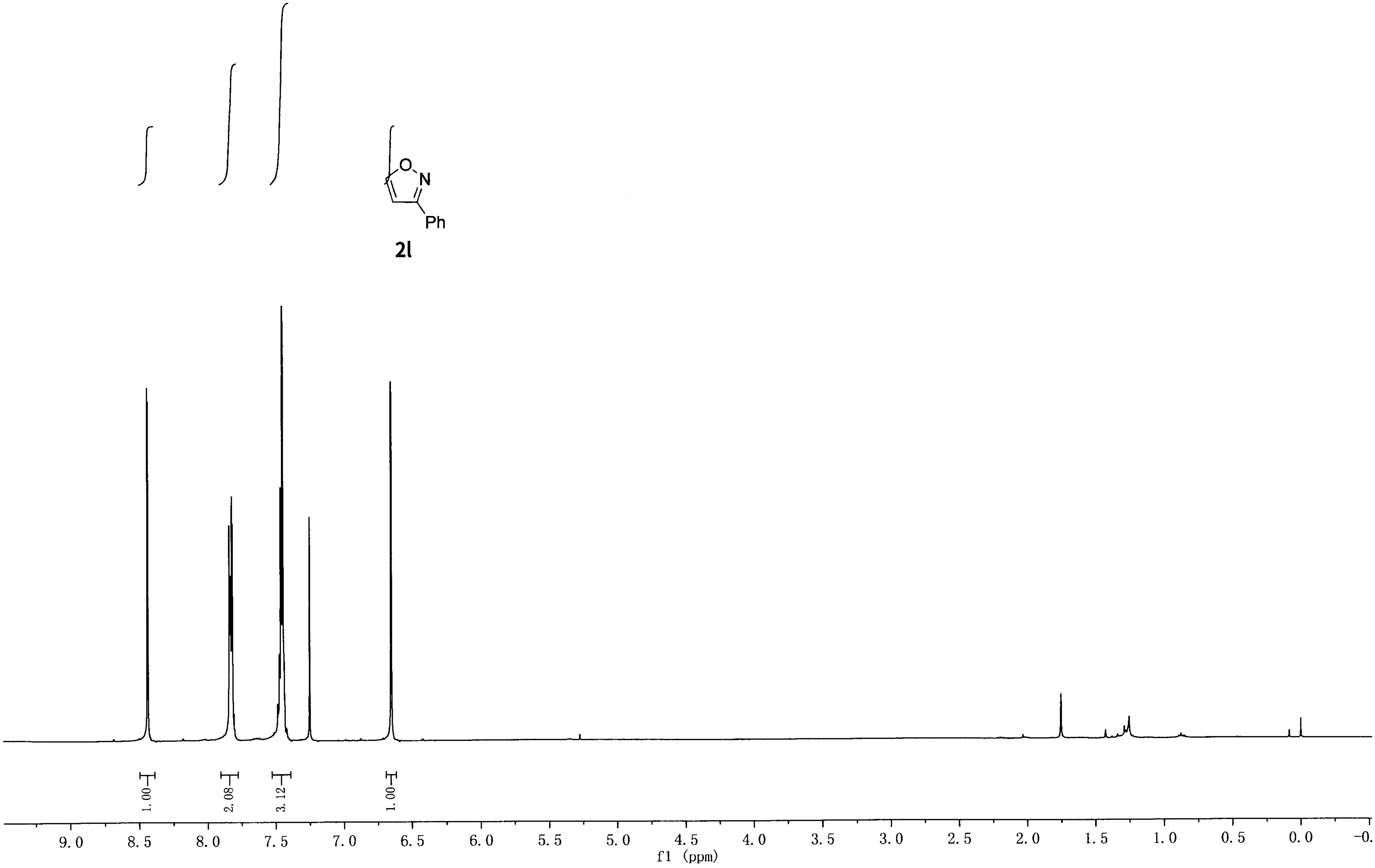
Ph
2l
1.00
2.08
3.12
1.00
9.0
8.5
8.0
7.5
7.0
6.5
6.0
5.5
5.0
4.5
4.0
3.5
3.0
2.5
2.0
1.5
1.0
0.5
0.0
-0.
f1 (ppm)

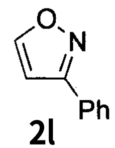
O
N
Ph

2l

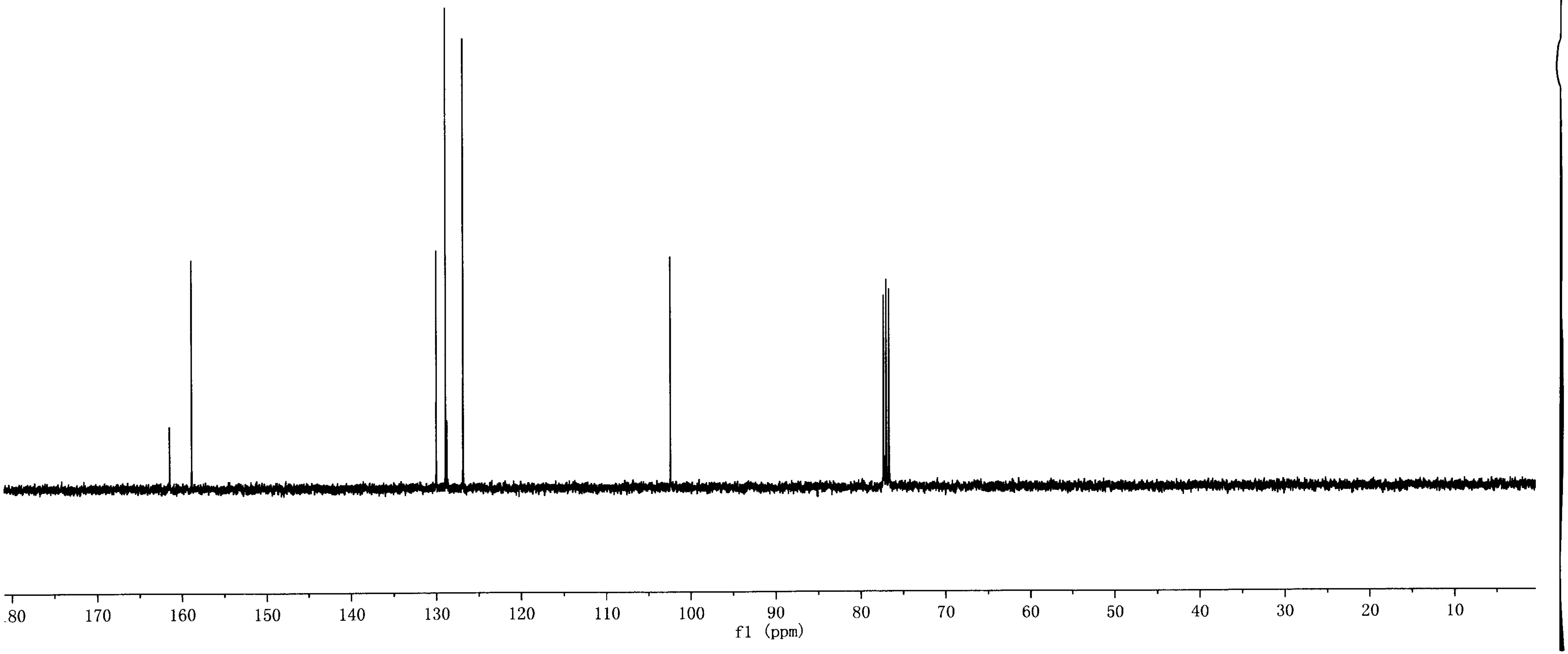
.80
170
160
150
140
130
120
110
100
90
80
70
60
50
40
30
20
10
f1 (ppm)

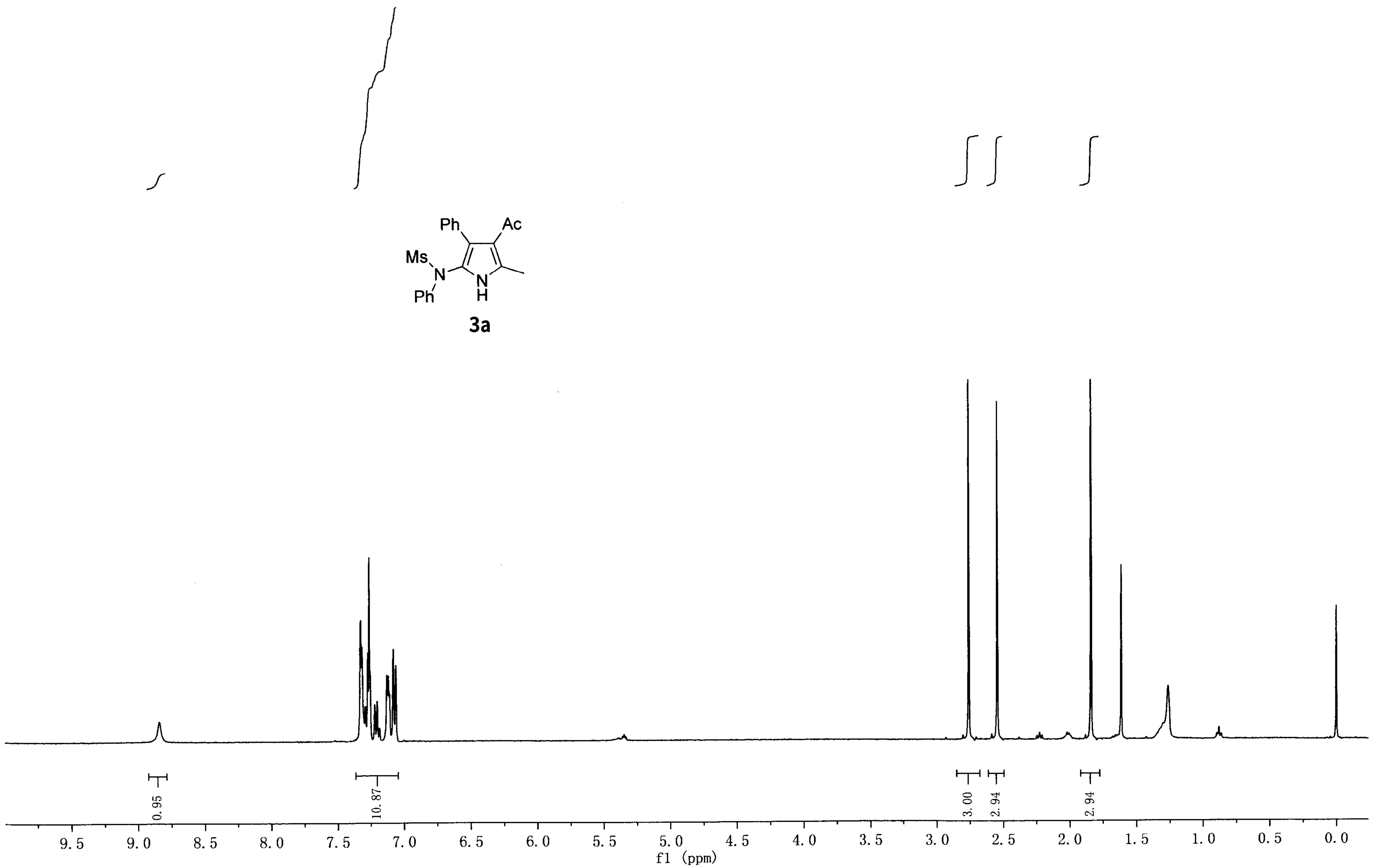
Ph
Ac
Ms
N
Ph
N
H
3a
0.95
10.87
3.00
2.94
2.94
9.5
9.0
8.5
8.0
7.5
7.0
6.5
6.0
5.5
5.0
4.5
4.0
3.5
3.0
2.5
2.0
1.5
1.0
0.5
0.0
f1 (ppm)

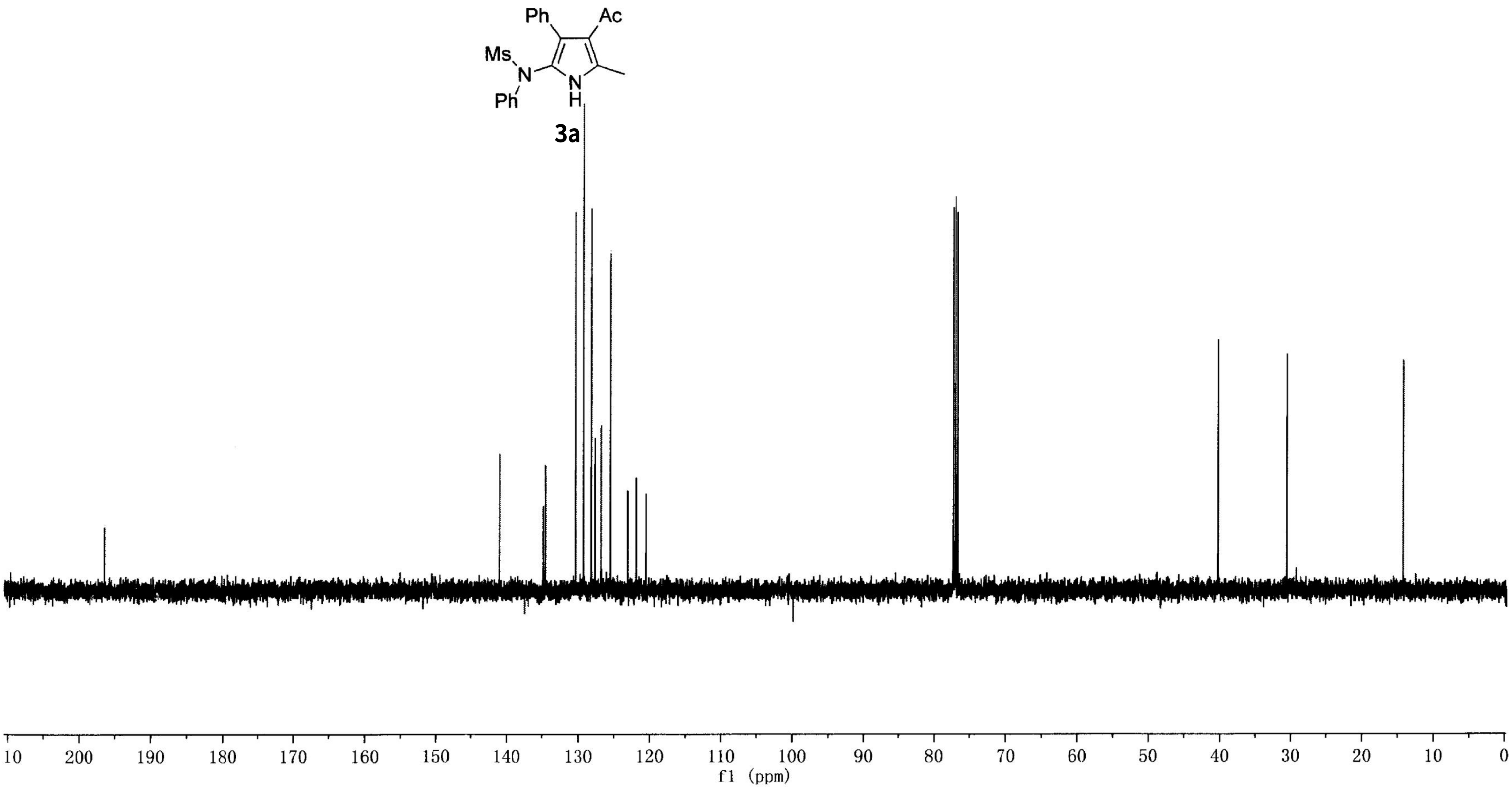
Ph
Ac
Ms
N
Ph
N
H
3a
10 200 190 180 170 160 150 140 130 120 110 100 90 80 70 60 50 40 30 20 10 0
f1 (ppm)

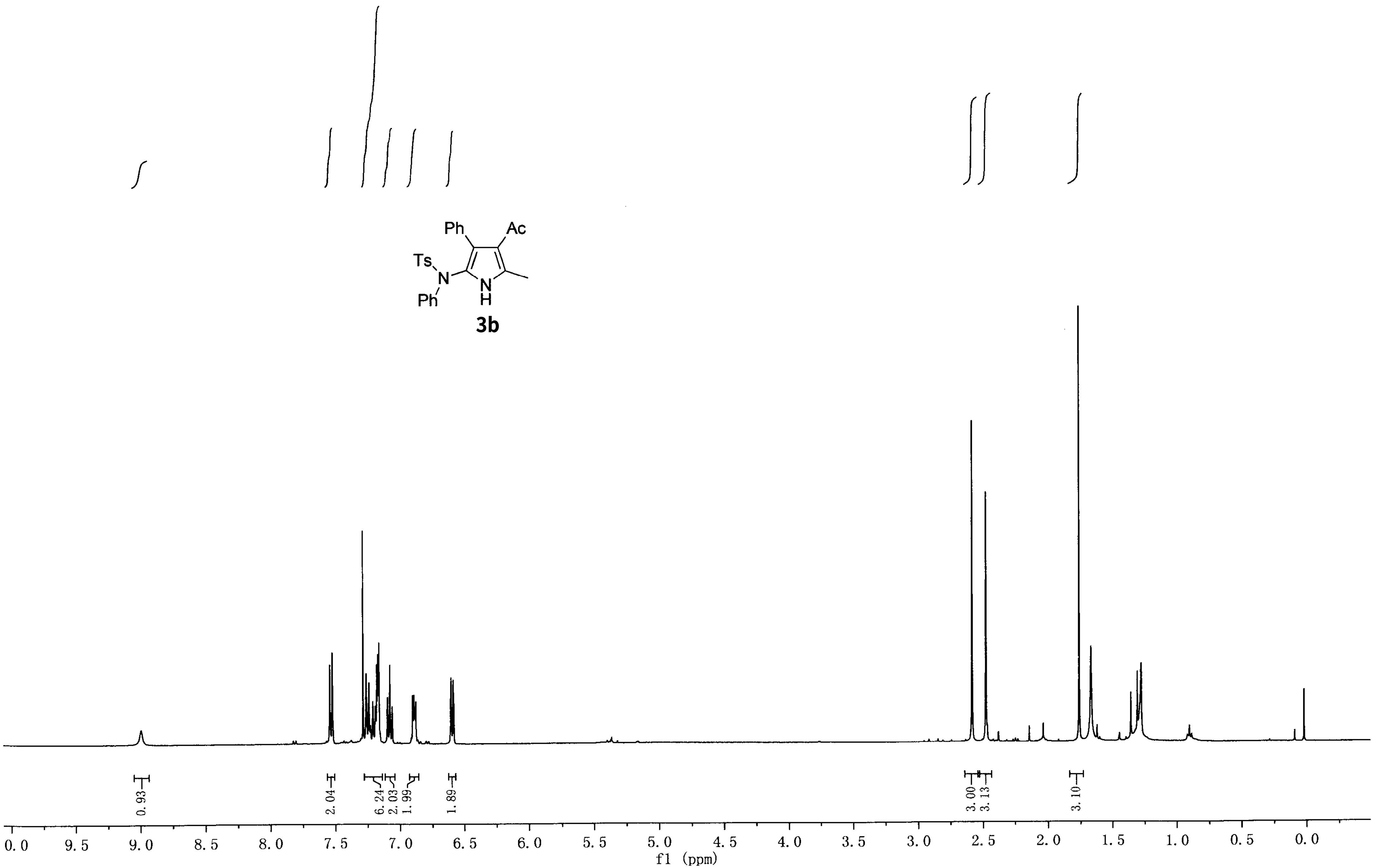

Ph
Ac
Ts
N
Ph
N
H
3b
0.93
2.04
6.24
2.03
1.99
1.89
3.00
3.13
3.10
0.0
9.5
9.0
8.5
8.0
7.5
7.0
6.5
6.0
5.5
5.0
4.5
4.0
3.5
3.0
2.5
2.0
1.5
1.0
0.5
0.0
f1 (ppm)

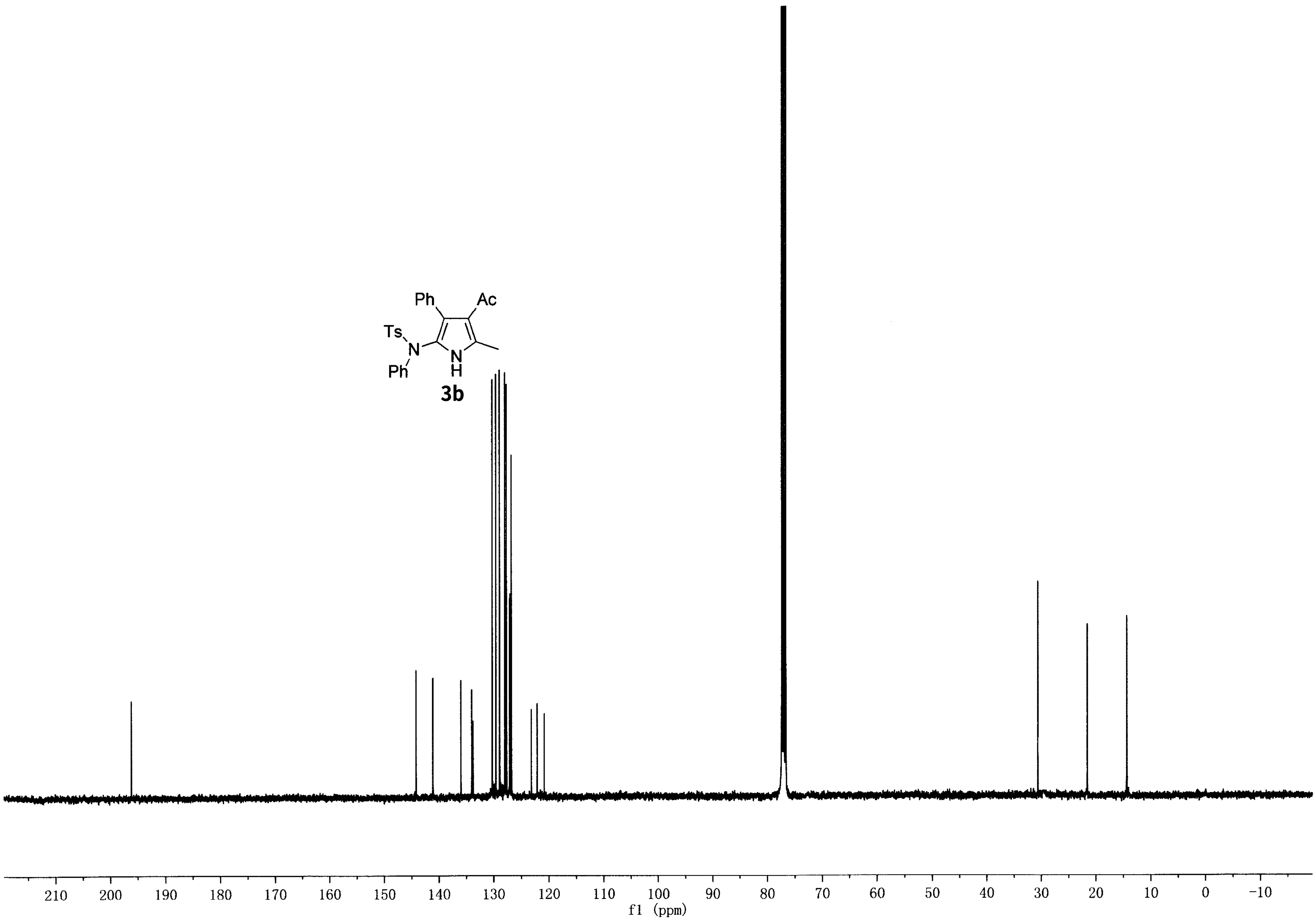
Ph
Ac
Ts
N
Ph
N
H
3b
210 200 190 180 170 160 150 140 130 120 110 100 90 80 70 60 50 40 30 20 10 0 −10
f1 (ppm)

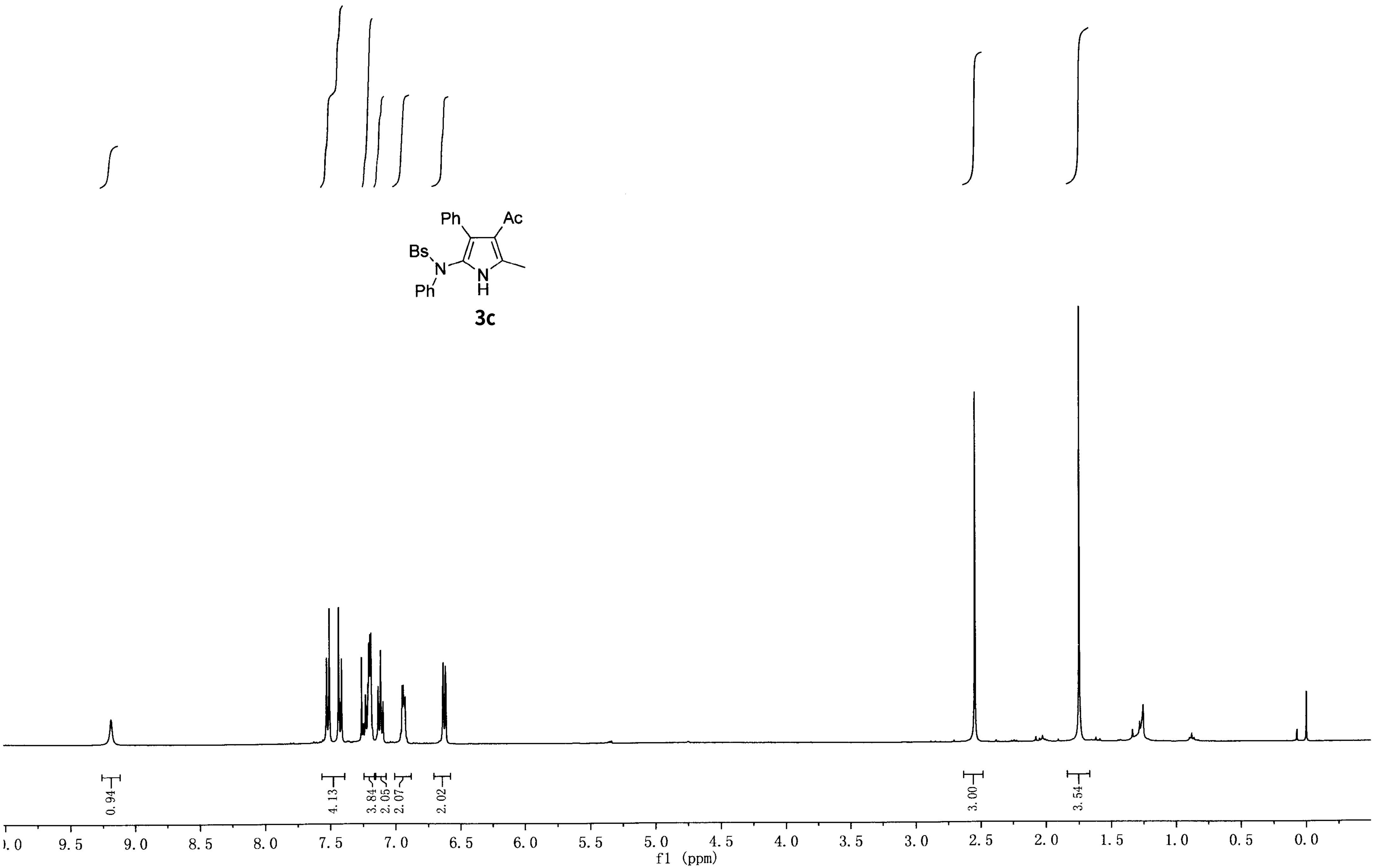
3c
0.94
4.13
3.84
2.05
2.07
2.02
3.00
3.54
). 0
9. 5
9. 0
8. 5
8. 0
7. 5
7. 0
6. 5
6. 0
5. 5
5. 0
4. 5
4. 0
3. 5
3. 0
2. 5
2. 0
1. 5
1. 0
0. 5
0. 0
f1 (ppm)

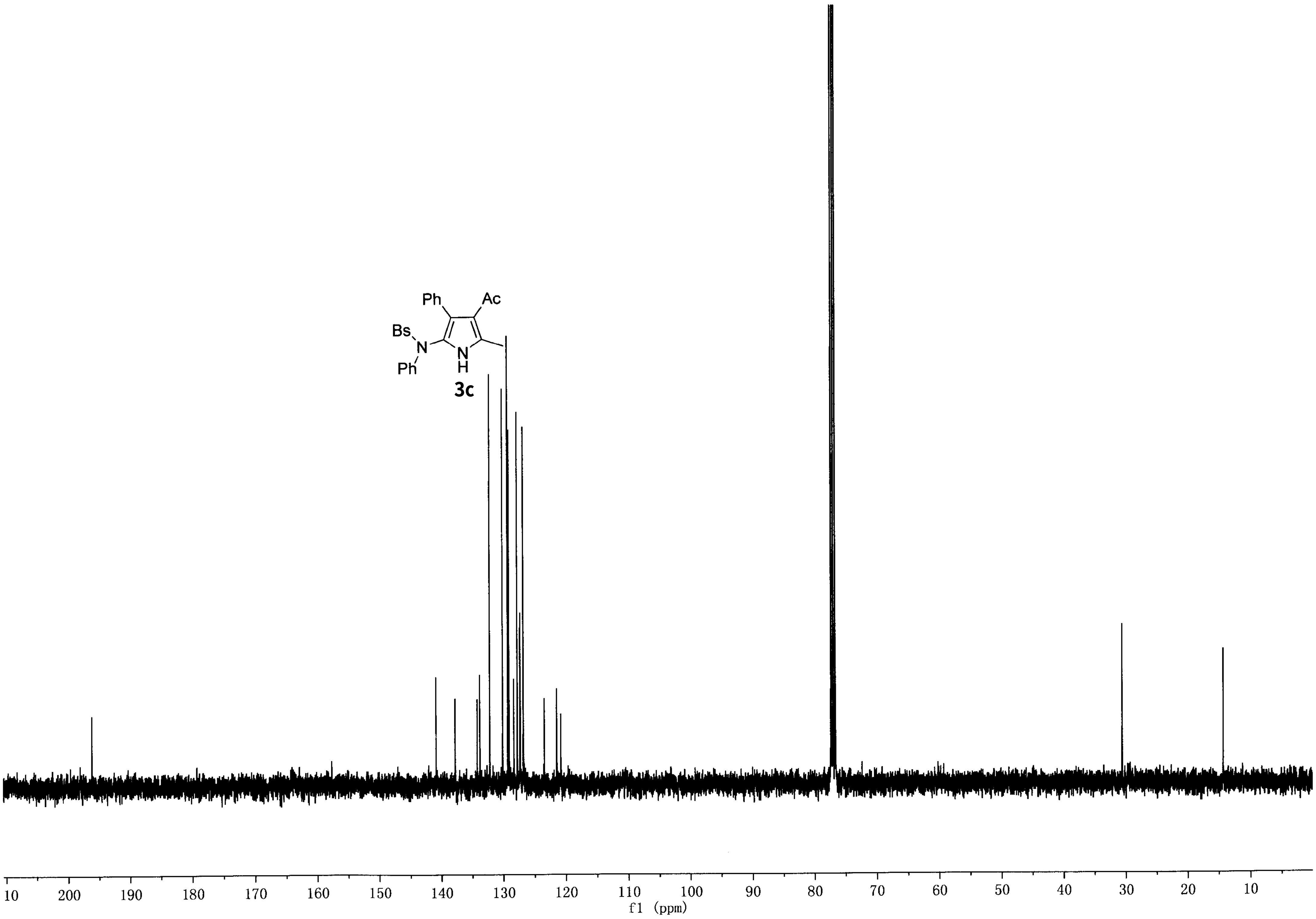

Ph
Ac
Bs
N
N
H
Ph
3c
10 200 190 180 170 160 150 140 130 120 110 100 90 80 70 60 50 40 30 20 10
f1 (ppm)

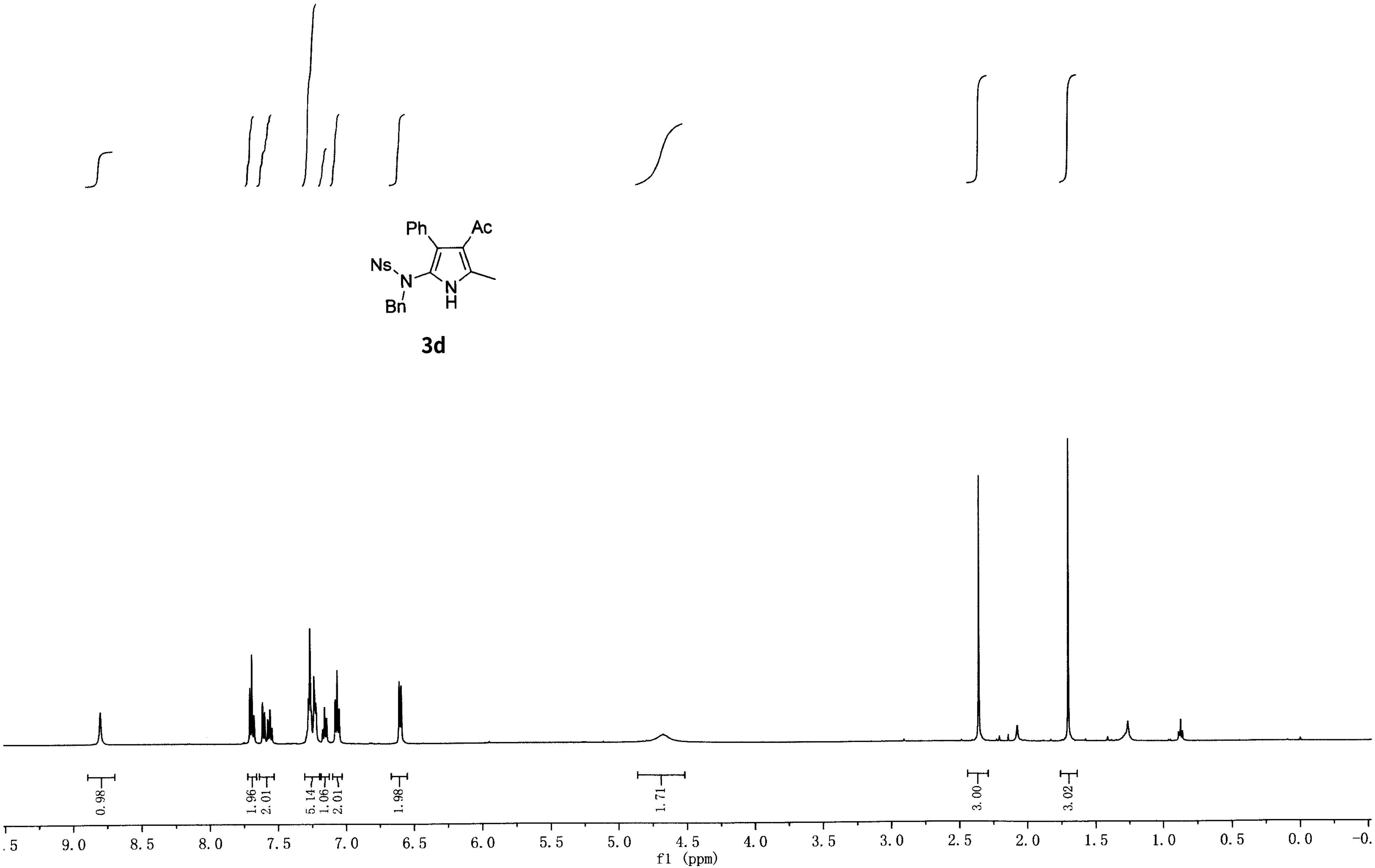
Ph
Ac
Ns
N
N
H
Bn
3d
0.98
1.96
2.01
5.14
1.06
2.01
1.98
1.71
3.00
3.02
9.0
8.5
8.0
7.5
7.0
6.5
6.0
5.5
5.0
4.5
4.0
3.5
3.0
2.5
2.0
1.5
1.0
0.5
0.0
f1 (ppm)

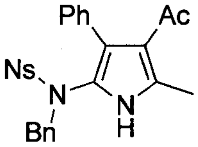
Ph
Ac
Ns
N
Bn
N
H

3d

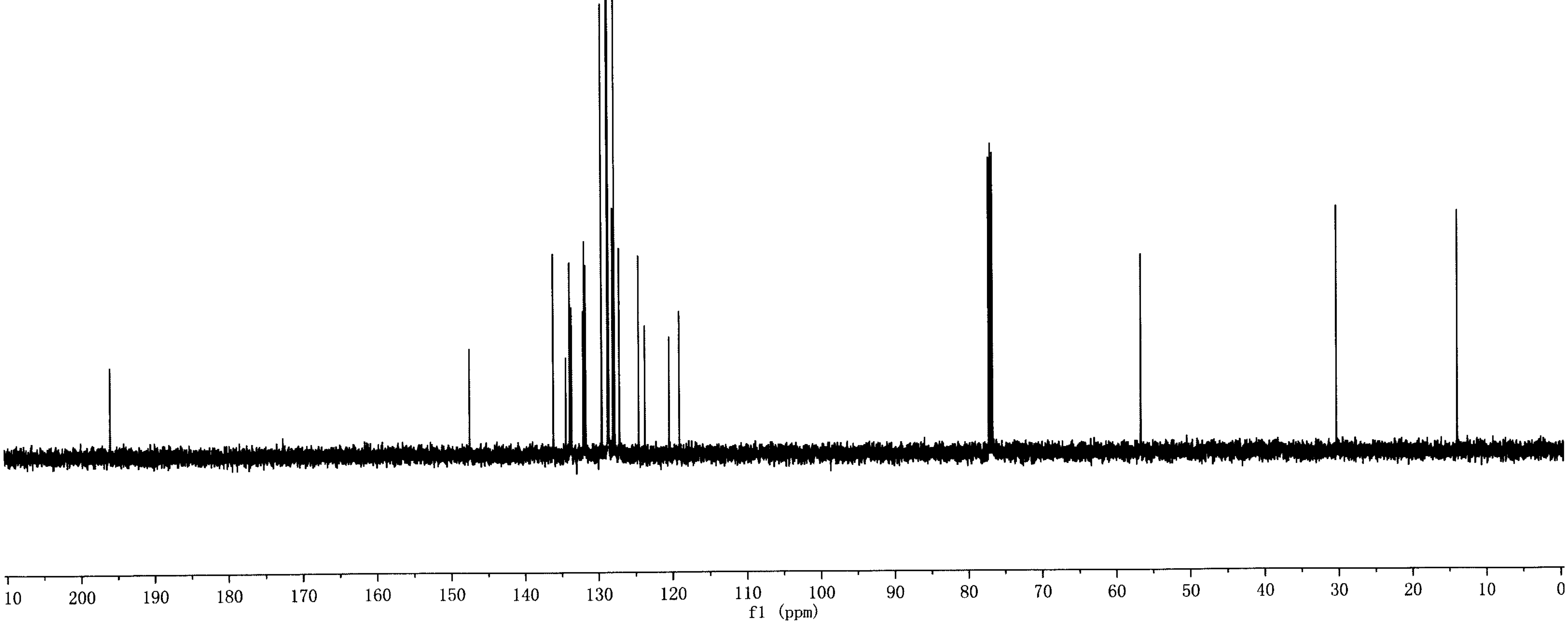
10
200
190
180
170
160
150
140
130
120
110
100
90
80
70
60
50
40
30
20
10
0
f1 (ppm)

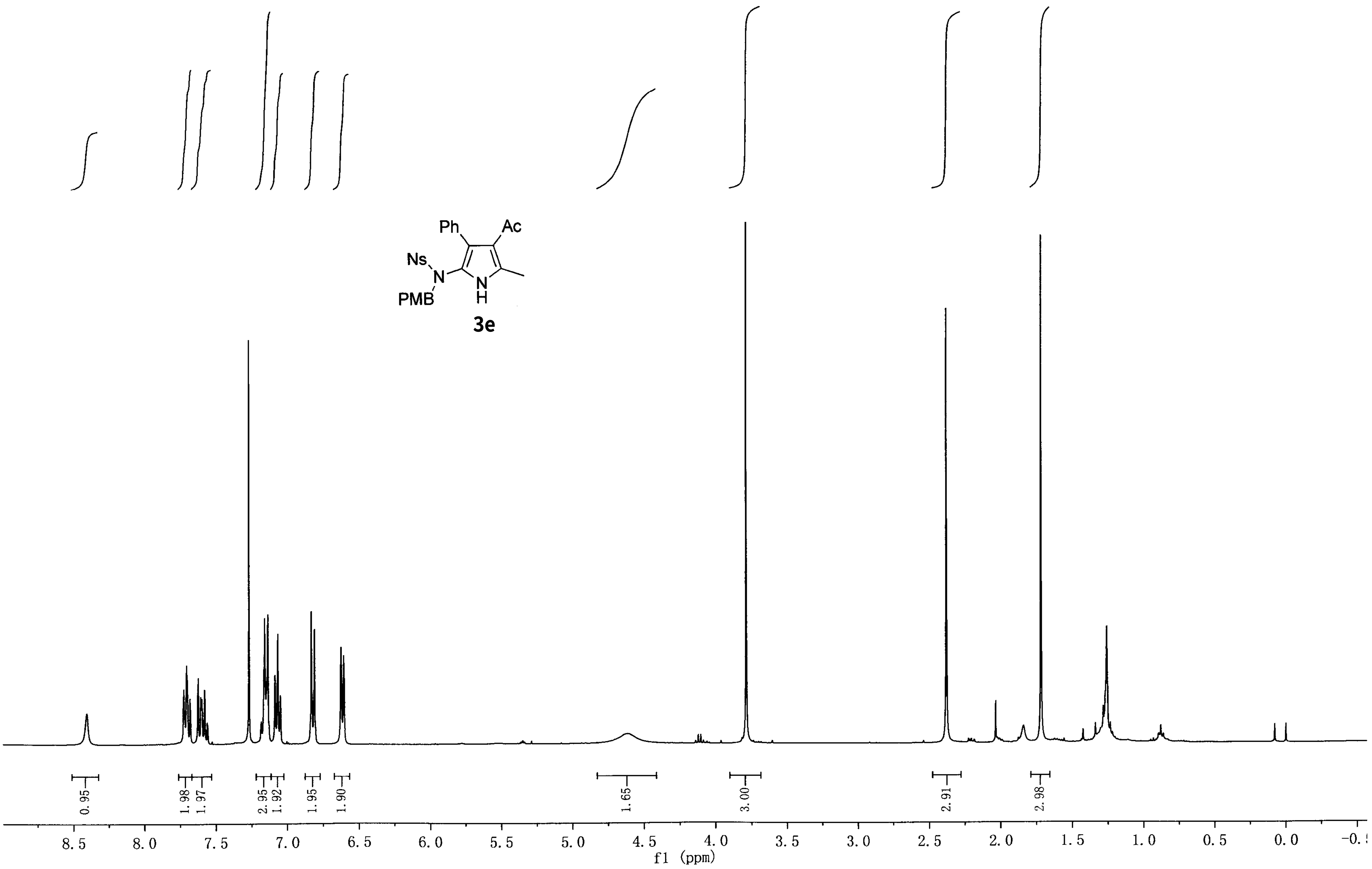

Ph
Ac
Ns
N
N
H
PMB
3e
0.95
1.98
1.97
2.95
1.92
1.95
1.90
1.65
3.00
2.91
2.98
8.5
8.0
7.5
7.0
6.5
6.0
5.5
5.0
4.5
4.0
3.5
3.0
2.5
2.0
1.5
1.0
0.5
0.0
f1 (ppm)

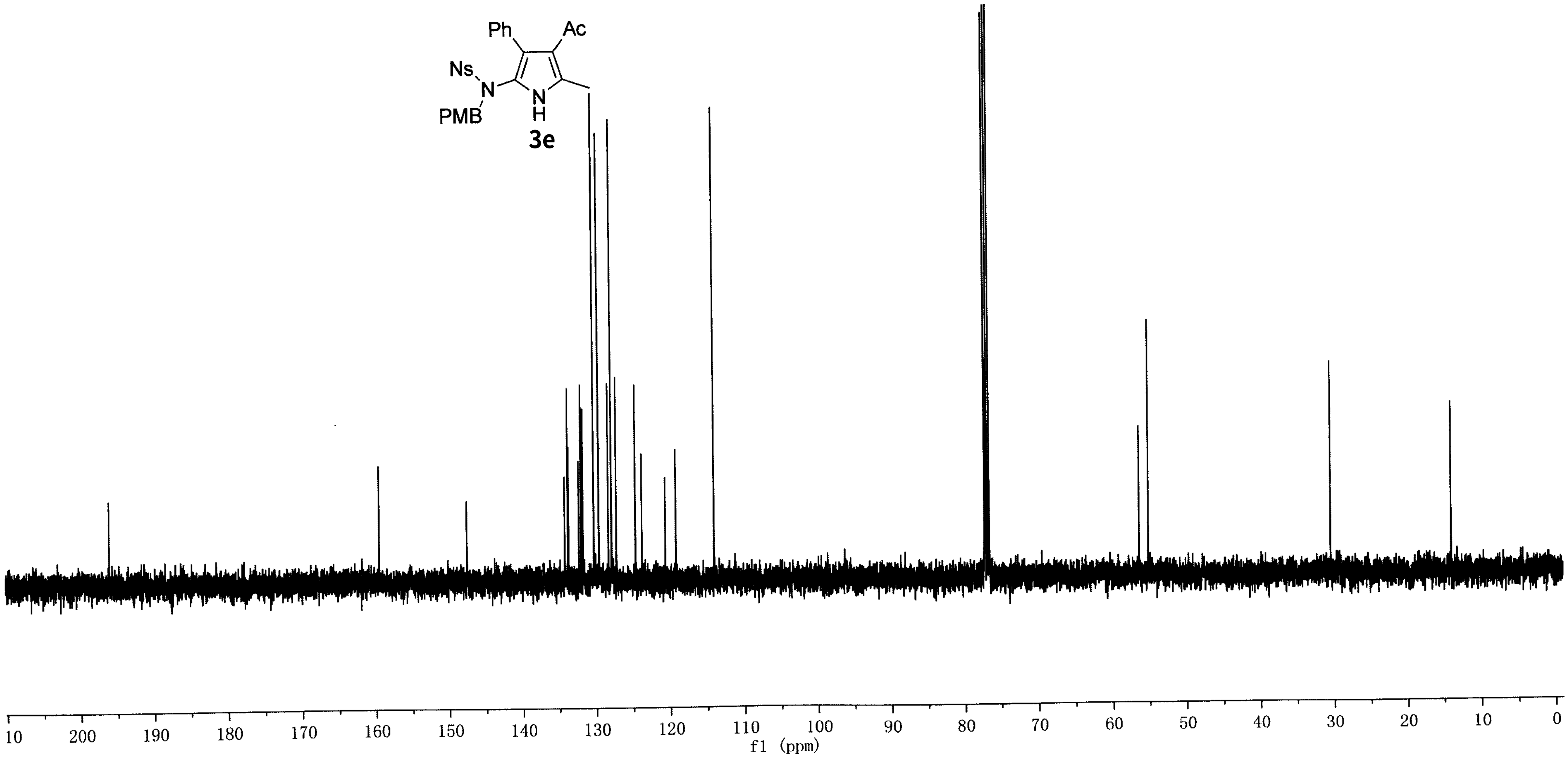
Ph
Ac
Ns
N
PMB
N
H
3e
10 200 190 180 170 160 150 140 130 120 110 100 90 80 70 60 50 40 30 20 10 0
f1 (ppm)

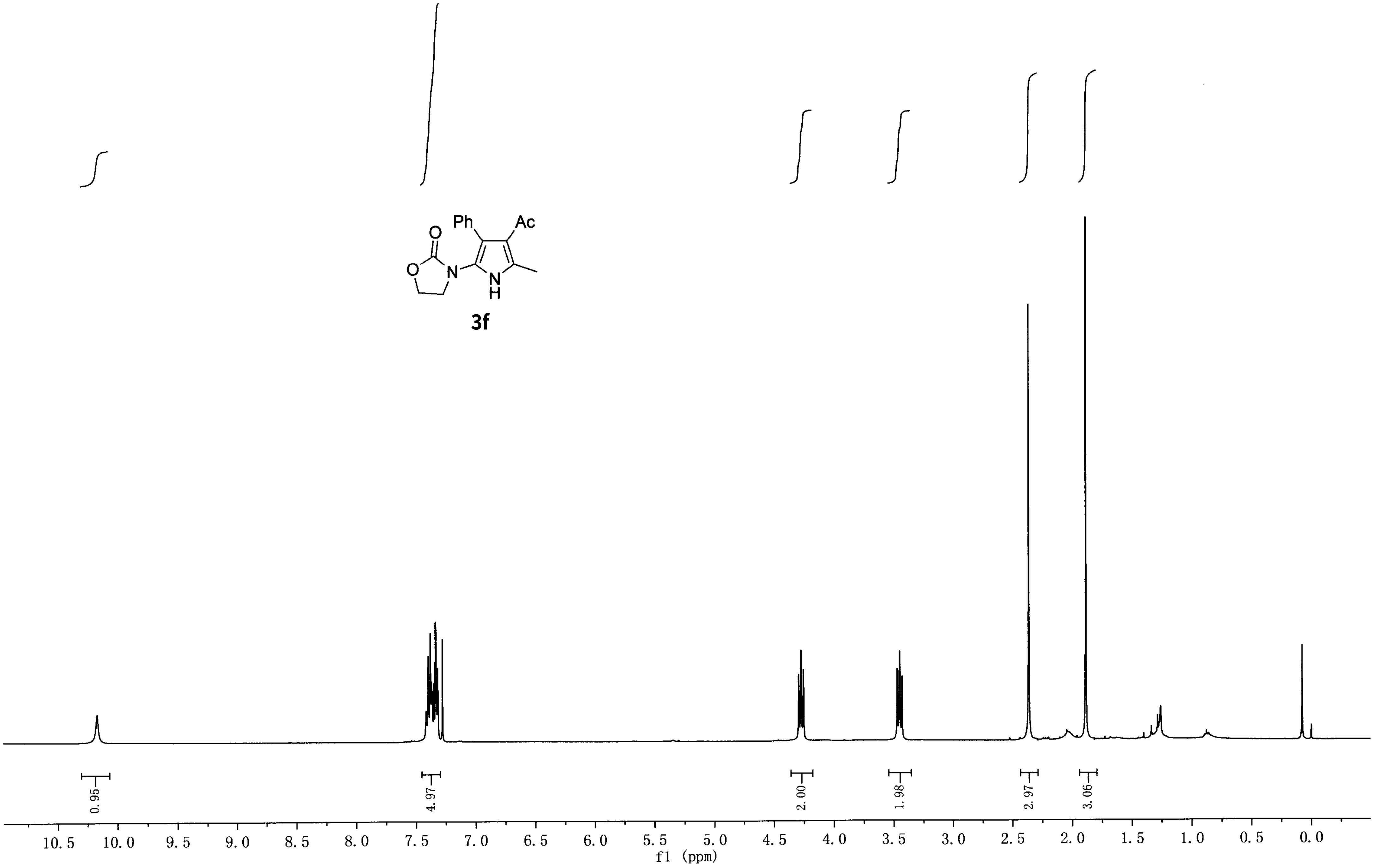
Ph
Ac
O
O
N
N
H
3f
0.95
4.97
2.00
1.98
2.97
3.06
10.5 10.0 9.5 9.0 8.5 8.0 7.5 7.0 6.5 6.0 5.5 5.0 4.5 4.0 3.5 3.0 2.5 2.0 1.5 1.0 0.5 0.0
f1 (ppm)

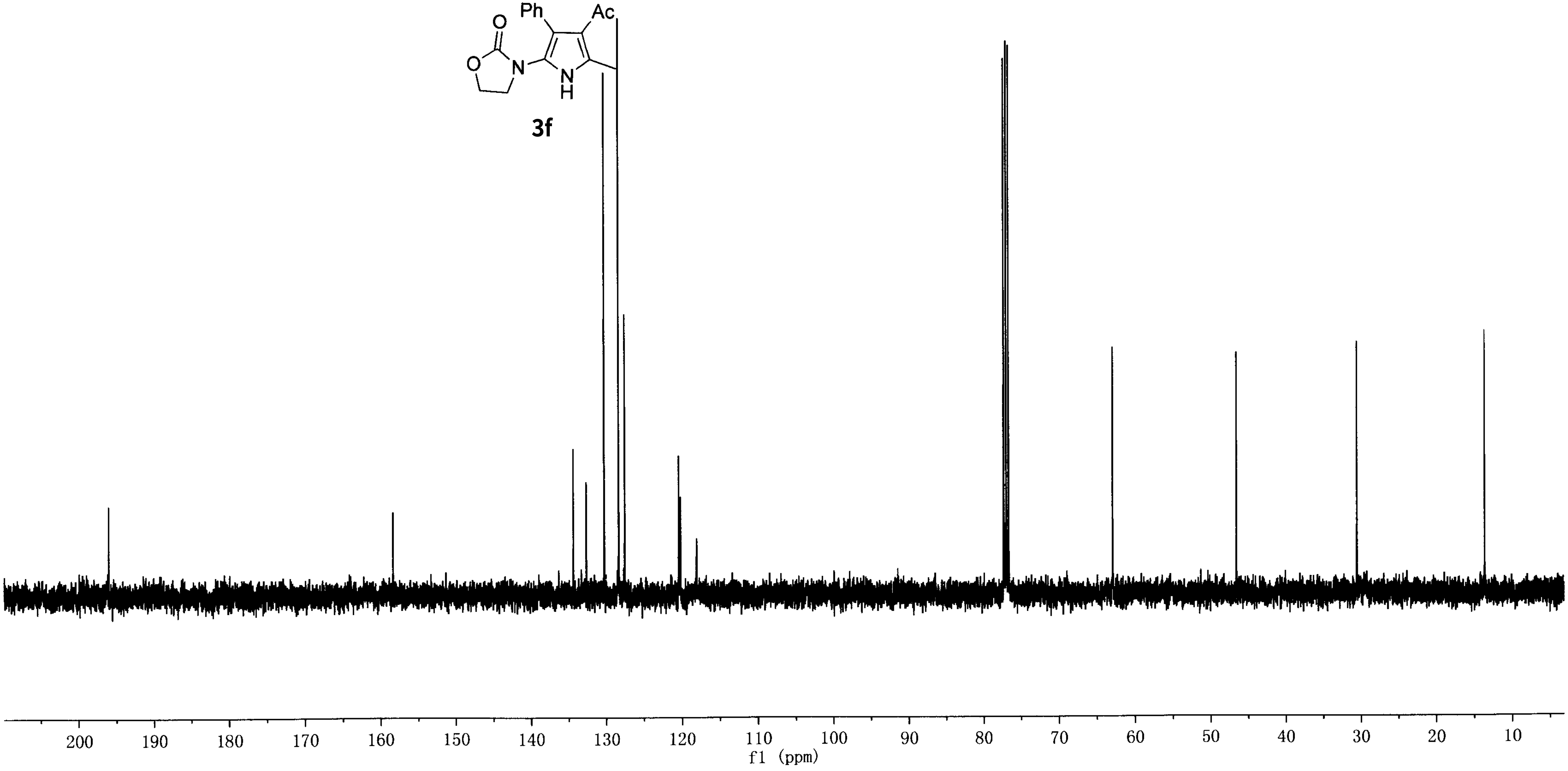

Ph
Ac
O
O
N
N
H
200
190
180
170
160
150
140
130
120
110
100
90
80
70
60
50
40
30
20
10
f1 (ppm)

3f

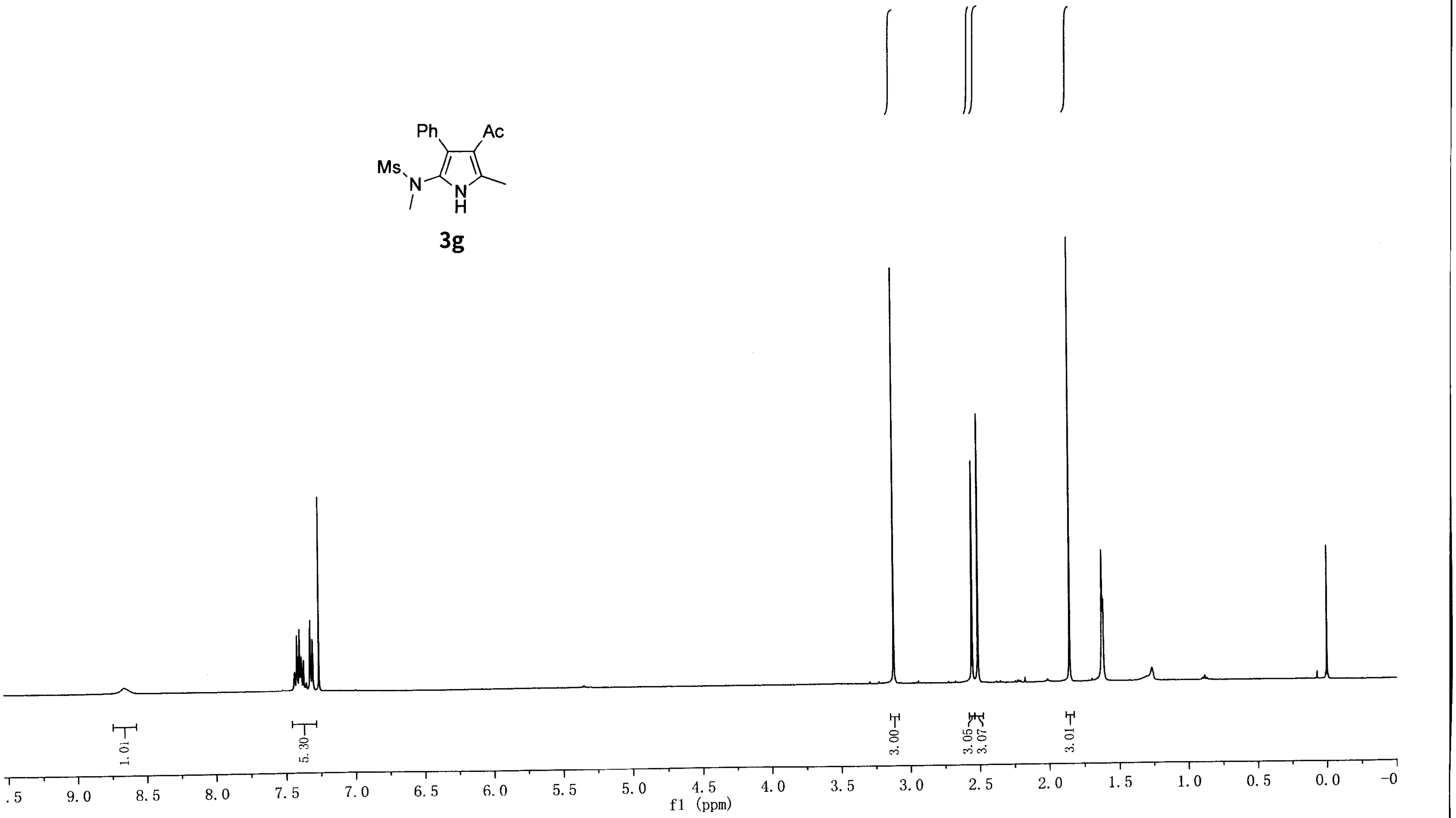

Ph
Ac
Ms
N
N
H
3g
1.01
5.30
3.00
3.05
3.07
3.01
.5
9.0
8.5
8.0
7.5
7.0
6.5
6.0
5.5
5.0
4.5
4.0
3.5
3.0
2.5
2.0
1.5
1.0
0.5
0.0
-0
f1 (ppm)

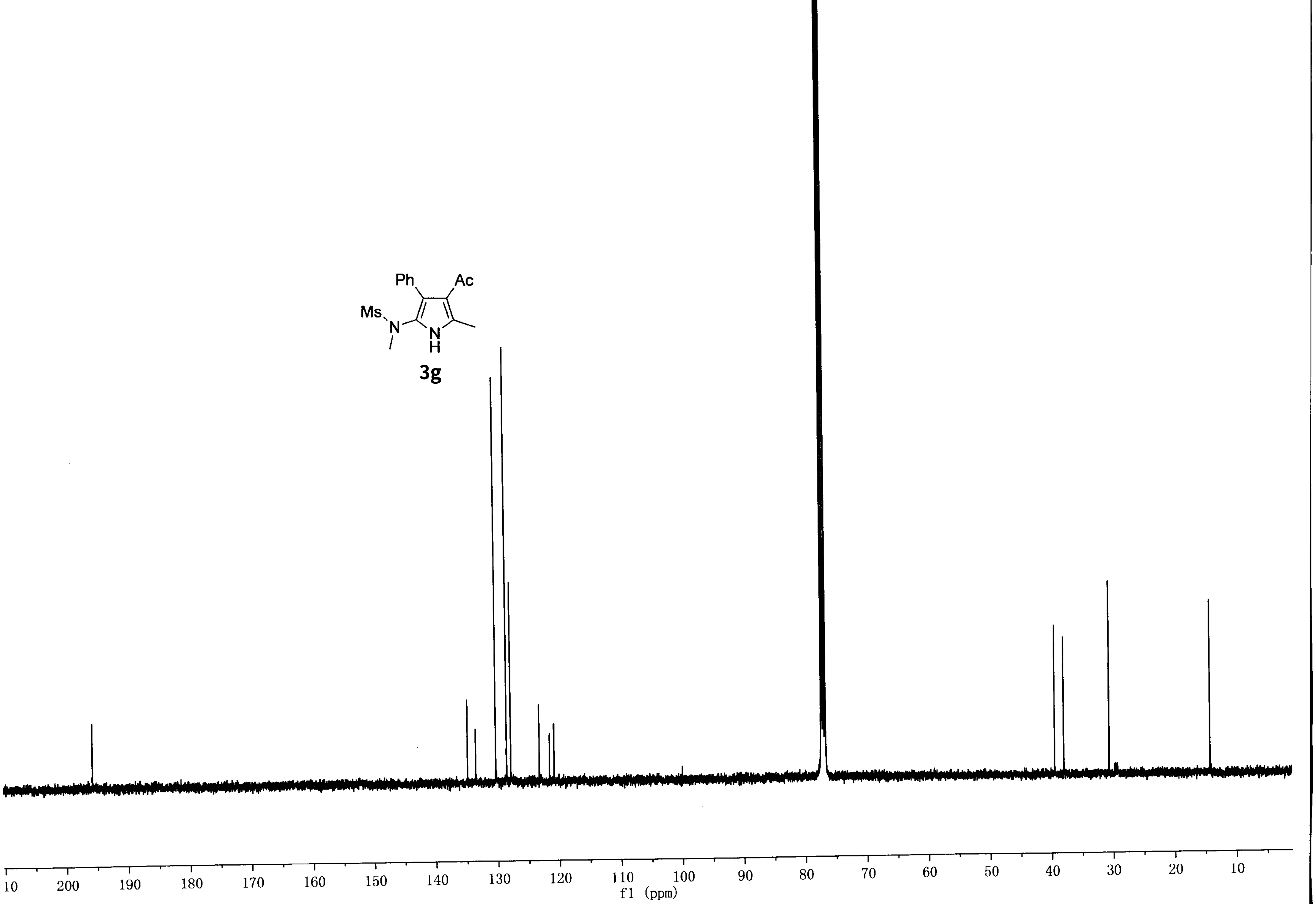
Ph
Ac
Ms
N
N
H
3g
10
200
190
180
170
160
150
140
130
120
110
100
90
80
70
60
50
40
30
20
10
f1 (ppm)

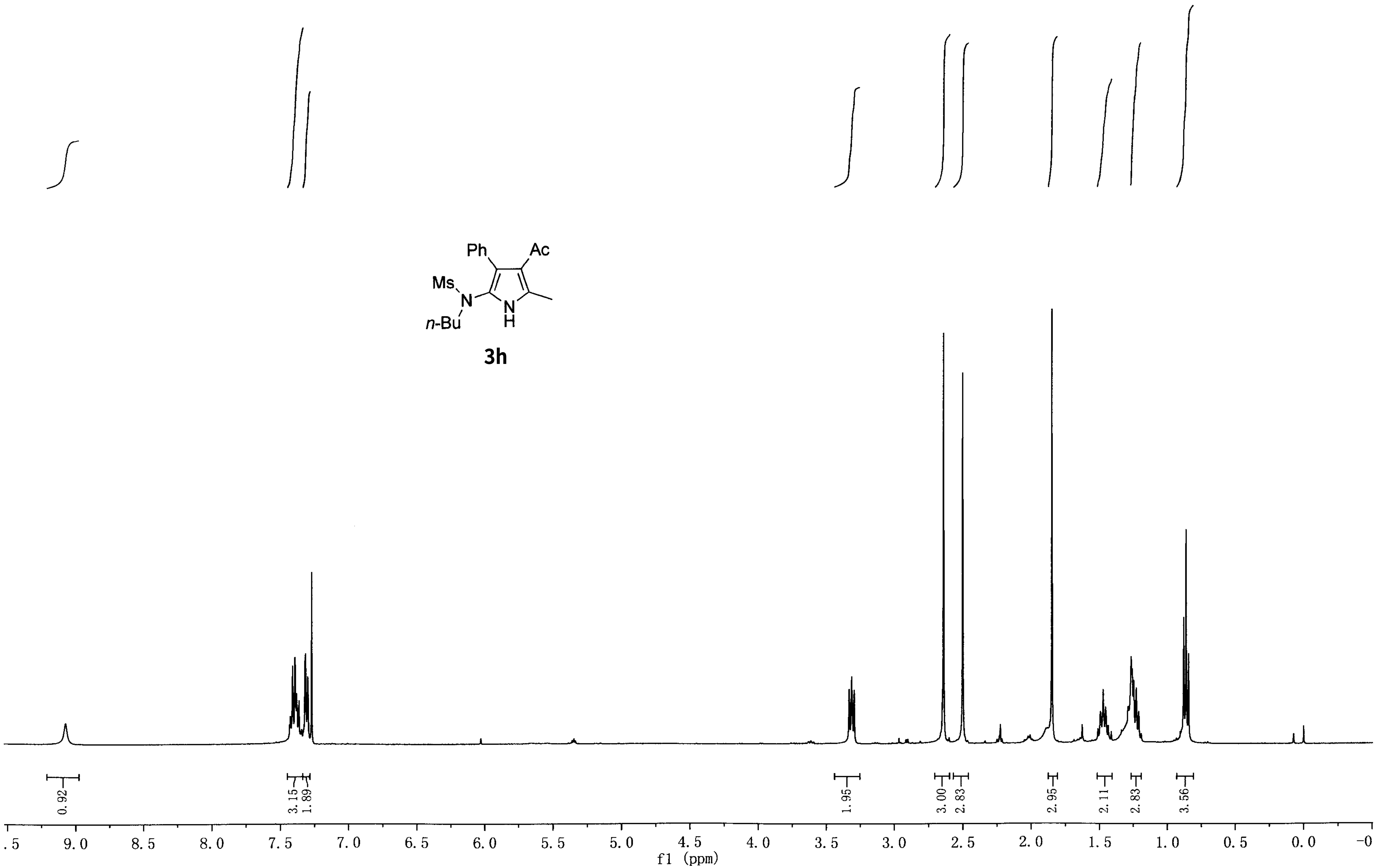

Ph
Ac
Ms
N
n-Bu
N
H
3h
0.92
3.15
1.89
1.95
3.00
2.83
2.95
2.11
2.83
3.56
.5
9.0
8.5
8.0
7.5
7.0
6.5
6.0
5.5
5.0
4.5
4.0
3.5
3.0
2.5
2.0
1.5
1.0
0.5
0.0
-0
f1 (ppm)

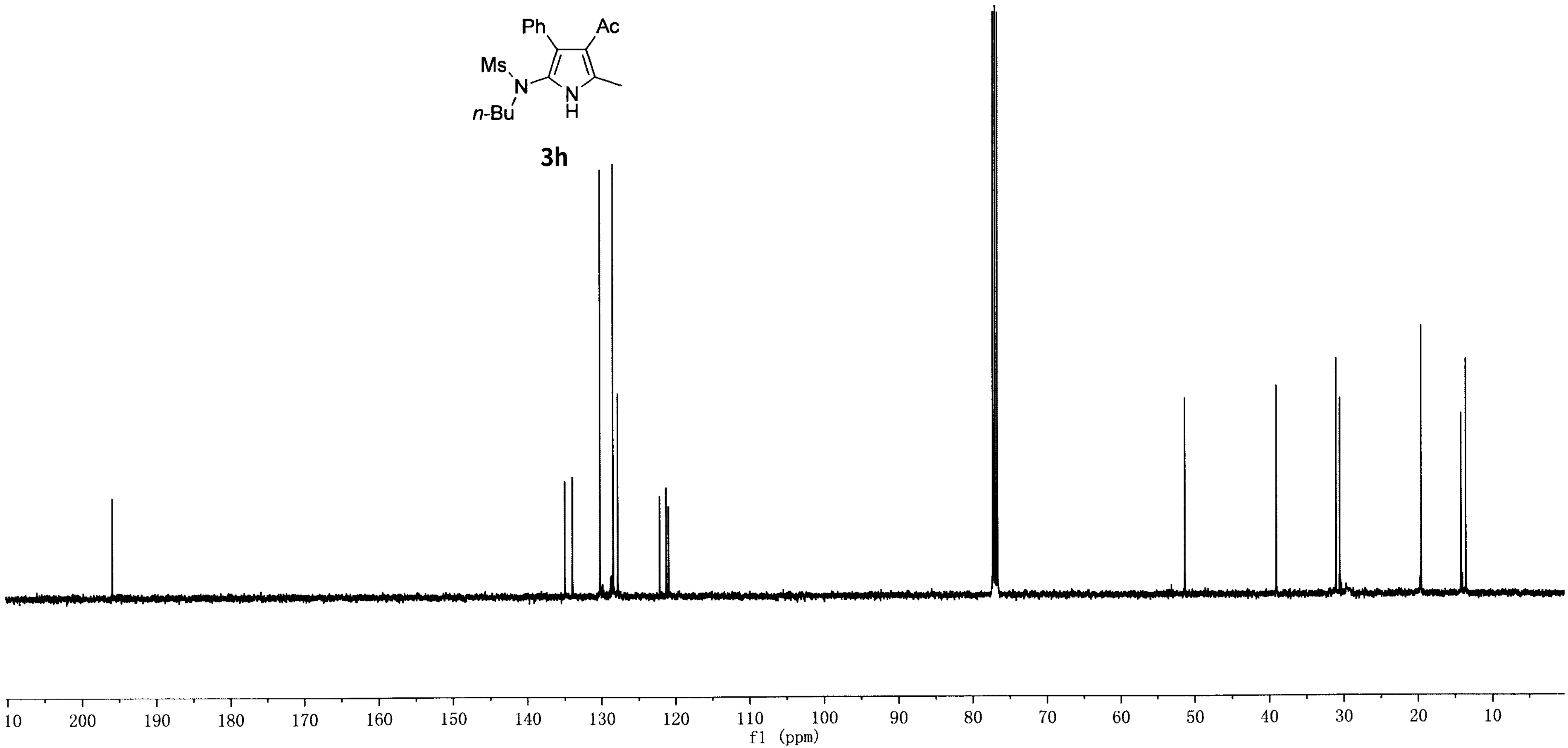
Ph
Ac
Ms
N
N
H
n-Bu
3h
10 200 190 180 170 160 150 140 130 120 110 100 90 80 70 60 50 40 30 20 10
f1 (ppm)

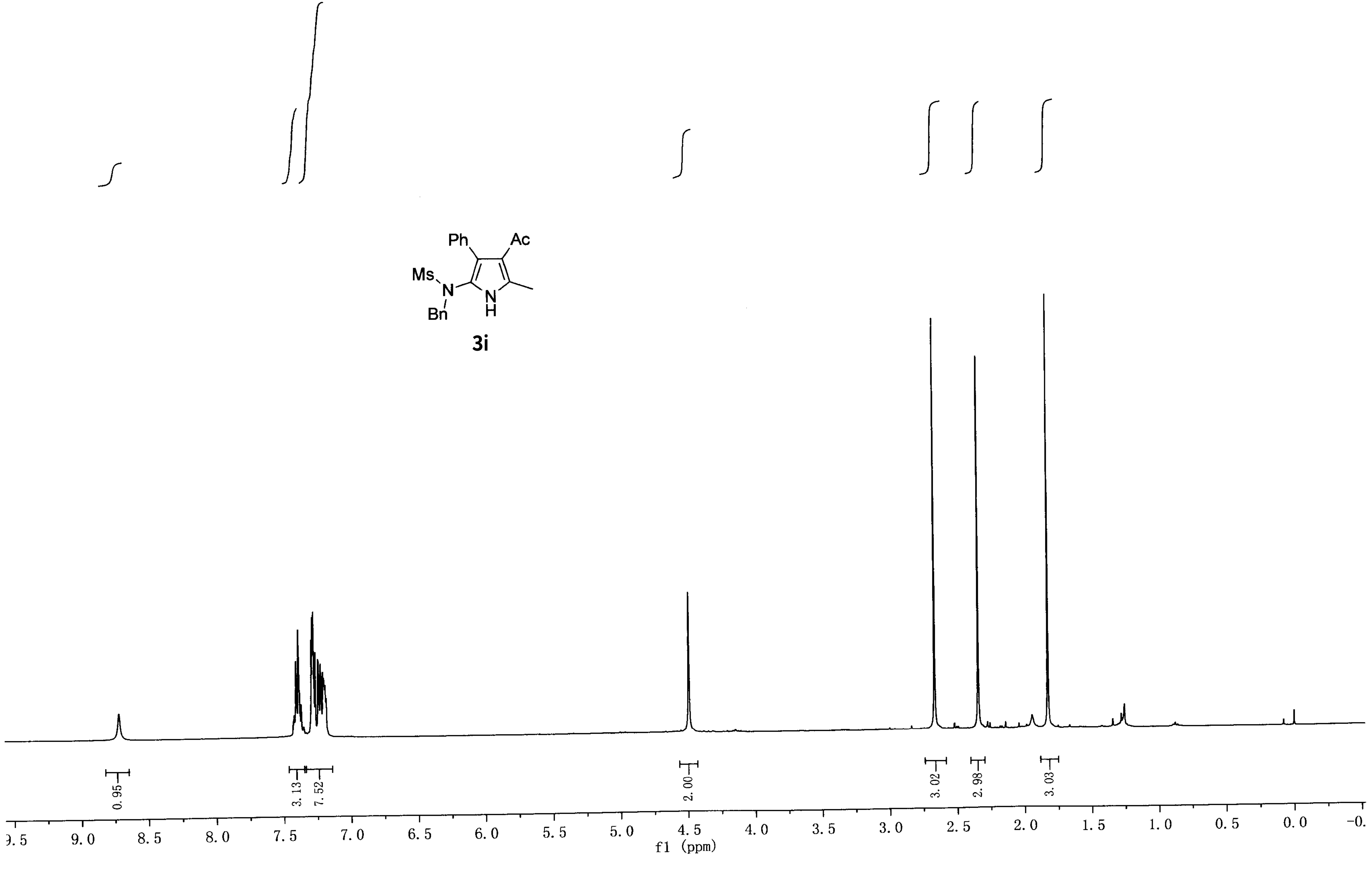

Ph
Ac
Ms
N
N
H
Bn
3i
0.95
3.13
7.52
2.00
3.02
2.98
3.03
9.0 8.5 8.0 7.5 7.0 6.5 6.0 5.5 5.0 4.5 4.0 3.5 3.0 2.5 2.0 1.5 1.0 0.5 0.0
f1 (ppm)

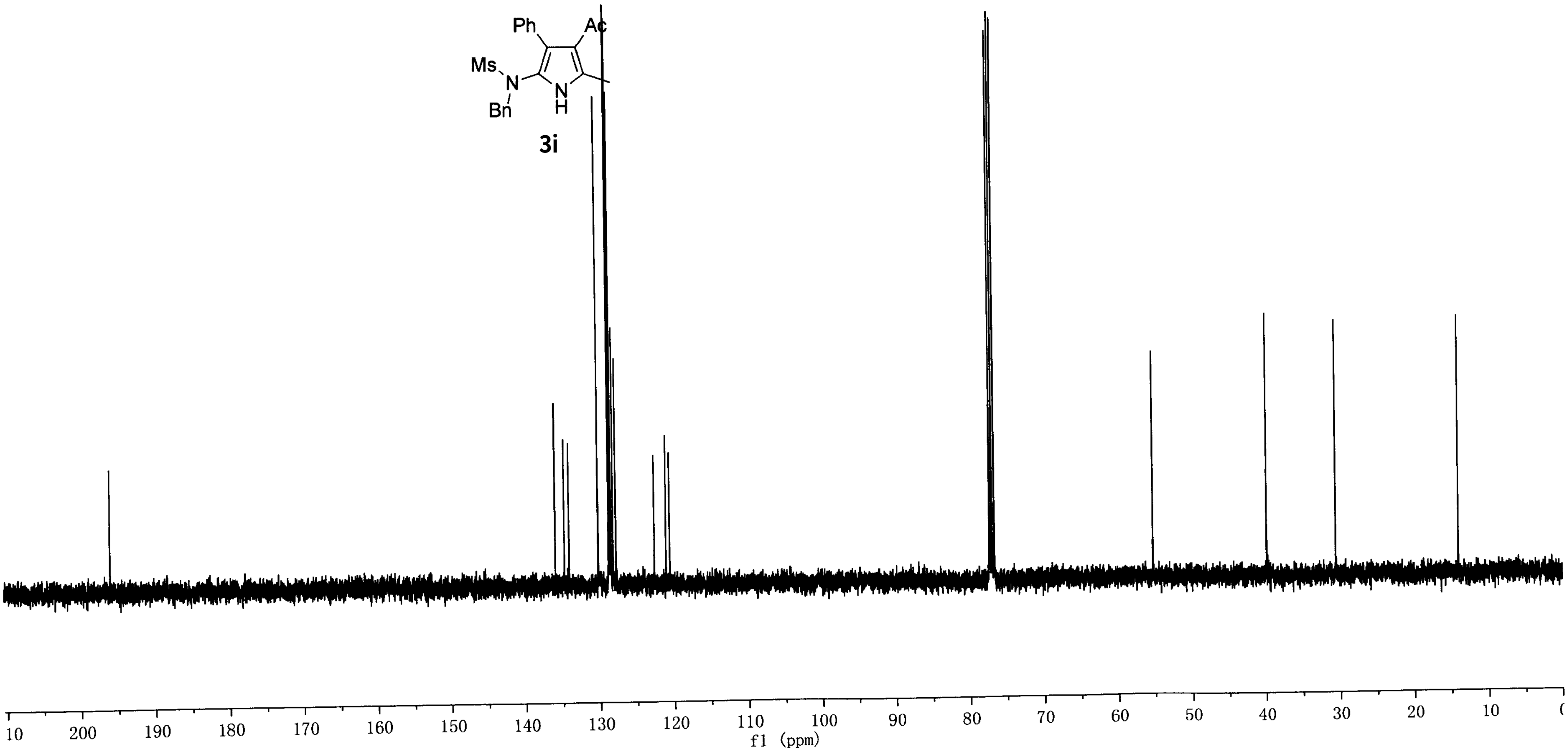

Ph
Ac
Ms
N
Bn
N
H
3i
10 200 190 180 170 160 150 140 130 120 110 100 90 80 70 60 50 40 30 20 10
f1 (ppm)

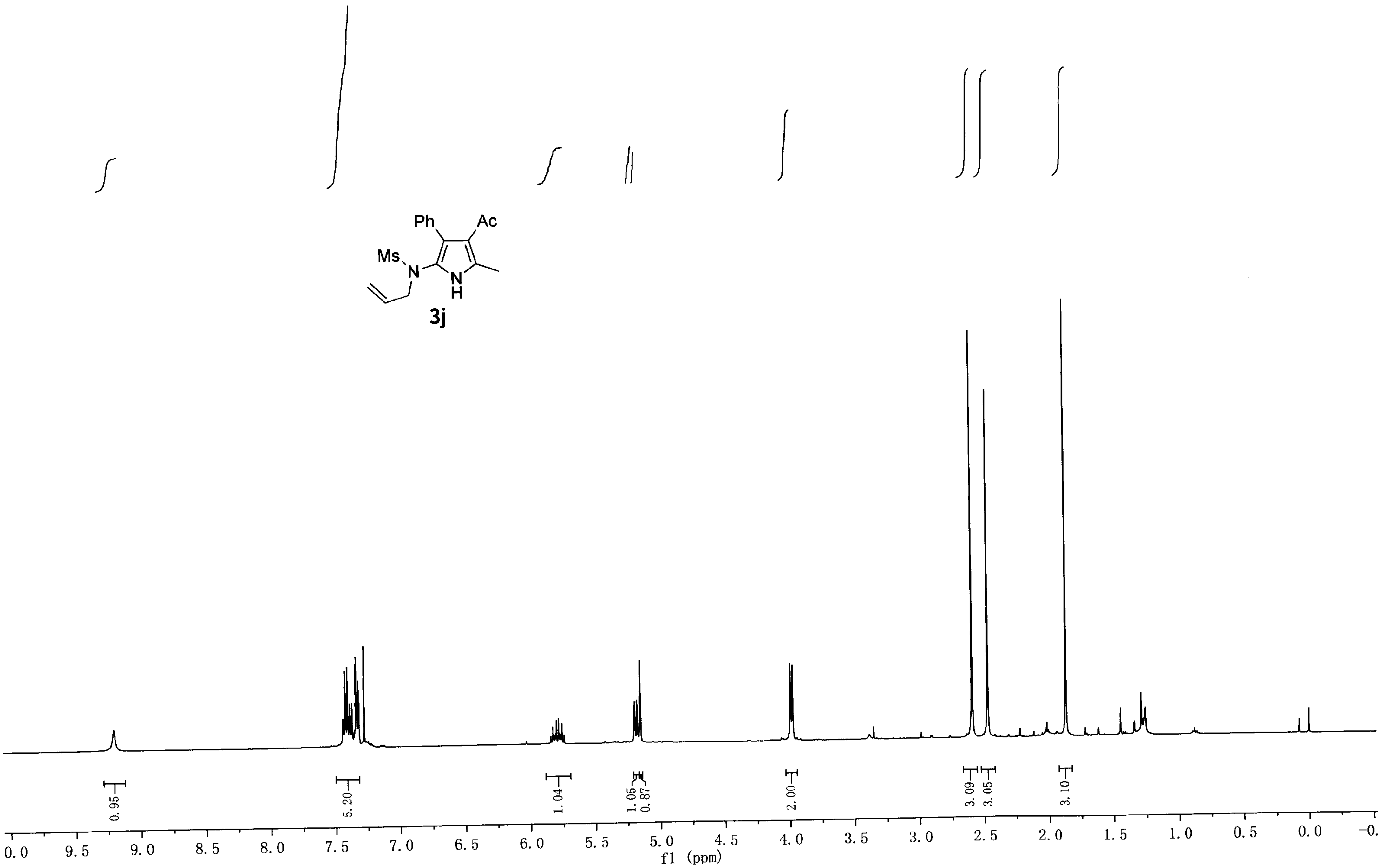

Ph
Ac
Ms
N
N
H
3j
0.95
5.20
1.04
1.05
0.87
2.00
3.09
3.05
3.10
0.0 9.5 9.0 8.5 8.0 7.5 7.0 6.5 6.0 5.5 5.0 4.5 4.0 3.5 3.0 2.5 2.0 1.5 1.0 0.5 0.0 -0.
f1 (ppm)

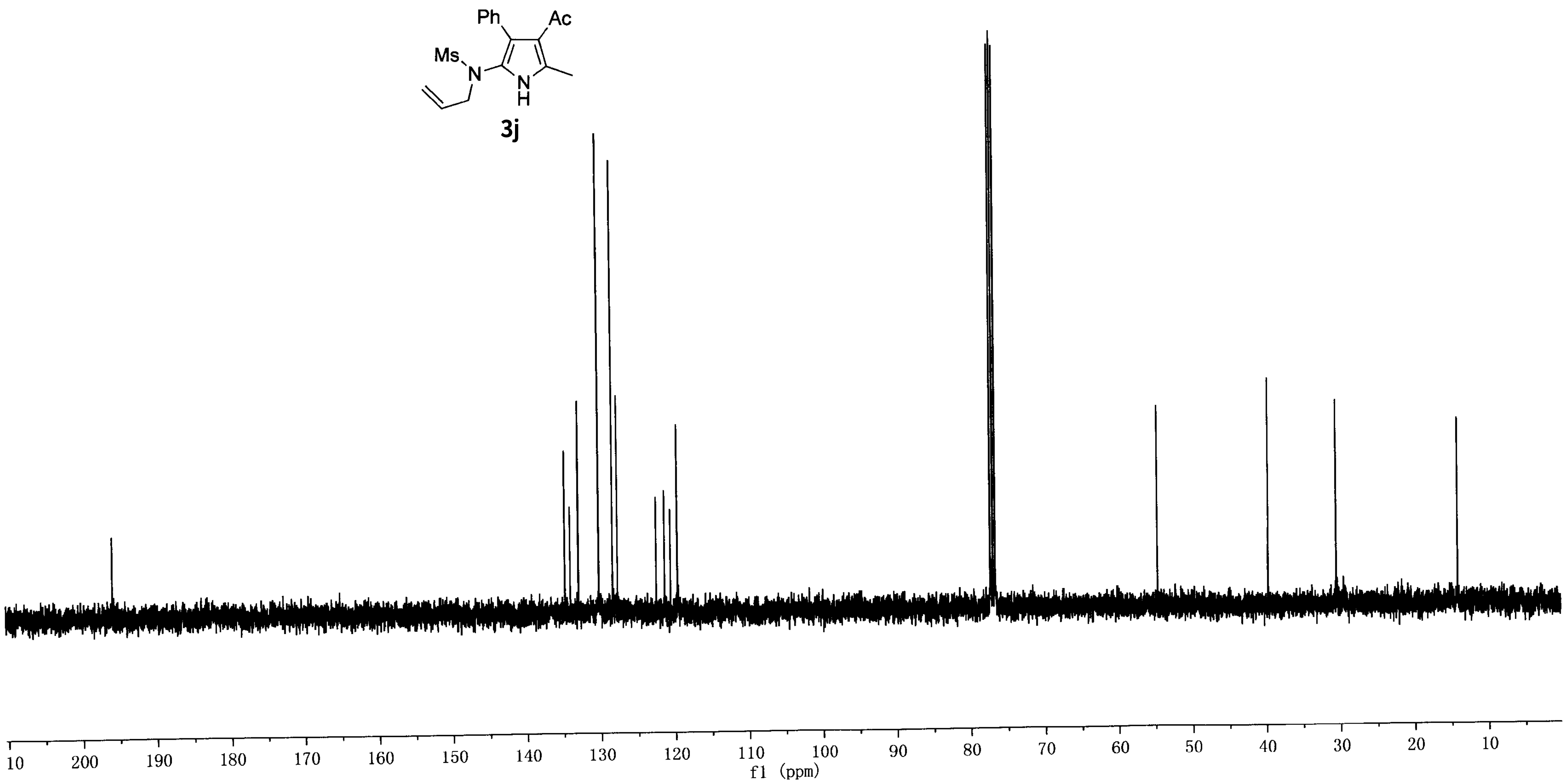
Ph
Ac
Ms
N
N
H
3j
10 200 190 180 170 160 150 140 130 120 110 100 90 80 70 60 50 40 30 20 10
f1 (ppm)

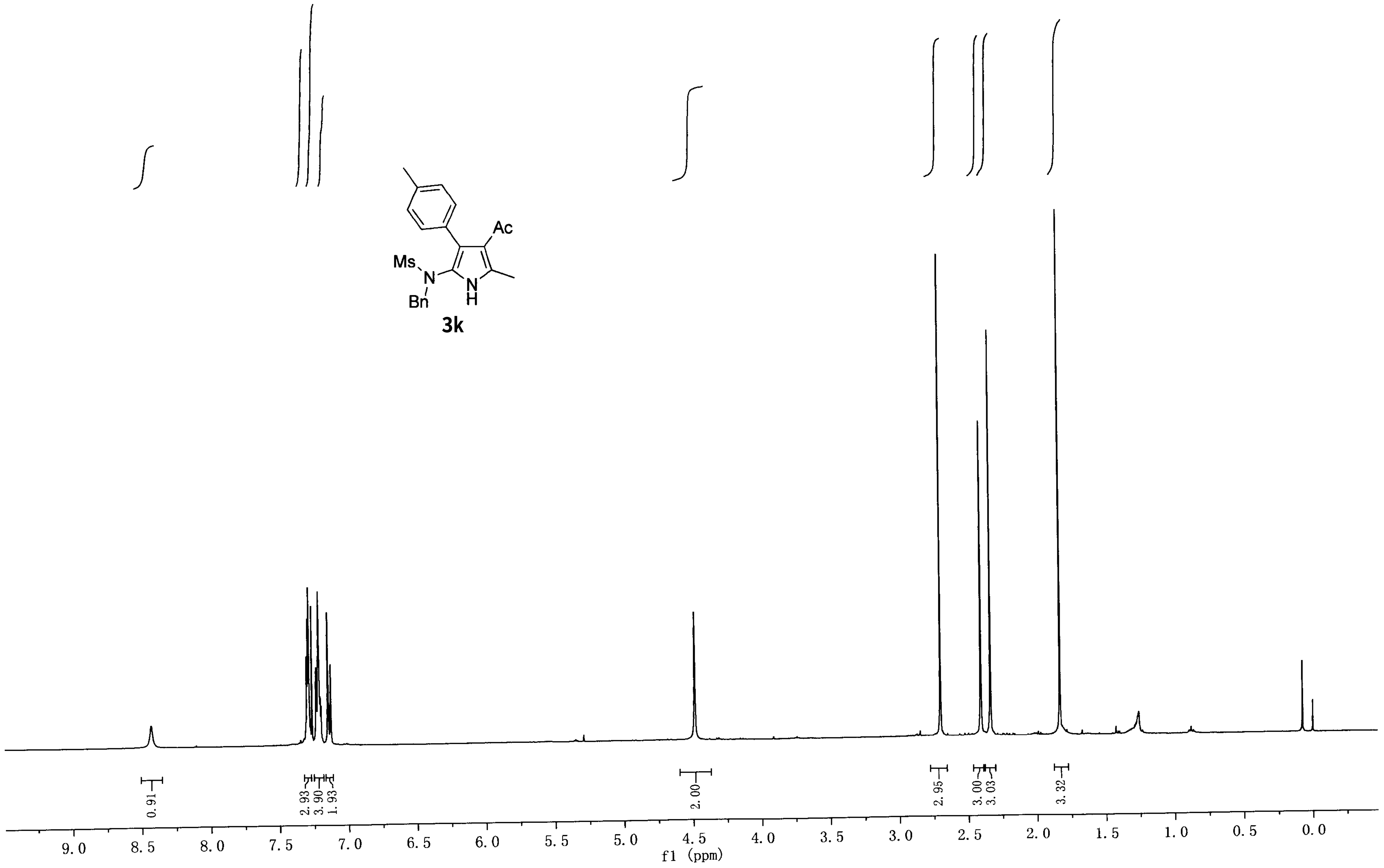

Ac
Ms
N
Bn
N
H
3k
0.91
2.93
3.90
1.93
2.00
2.95
3.00
3.03
3.32
9.0
8.5
8.0
7.5
7.0
6.5
6.0
5.5
5.0
4.5
4.0
3.5
3.0
2.5
2.0
1.5
1.0
0.5
0.0
f1 (ppm)

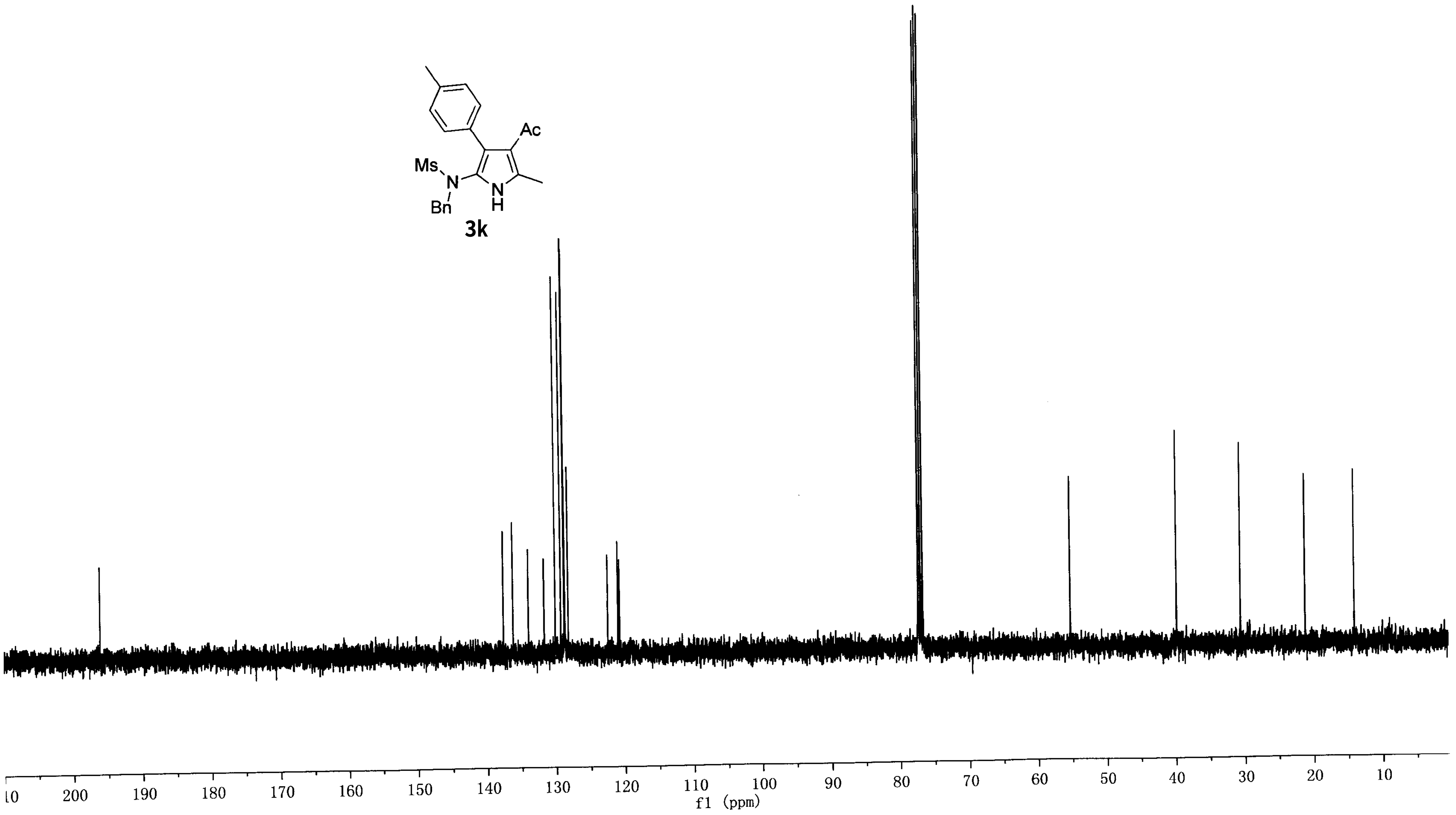
Ac
Ms
N
Bn
N
H
3k
10
200
190
180
170
160
150
140
130
120
110
100
90
80
70
60
50
40
30
20
10
f1 (ppm)

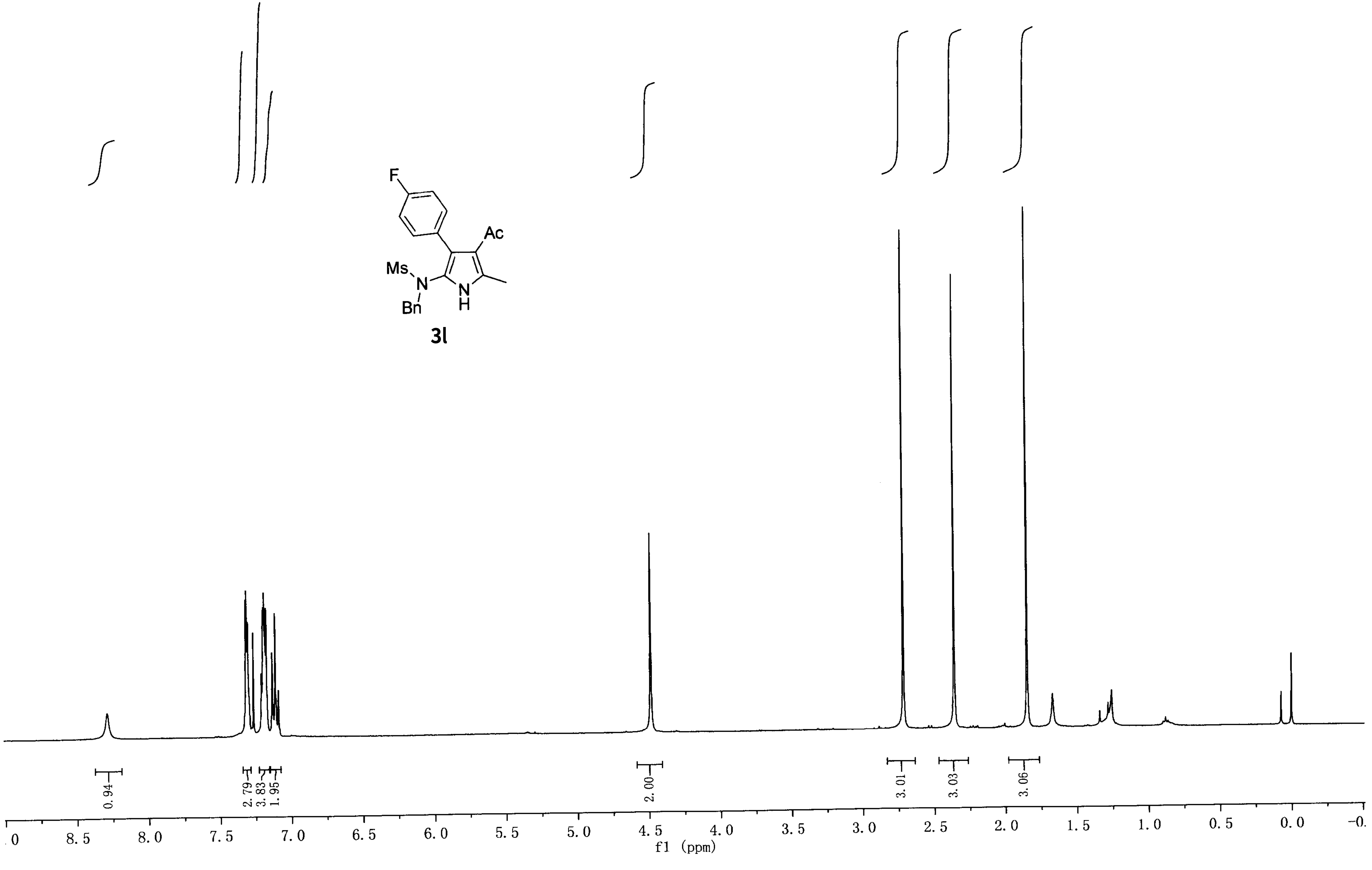
F
Ac
Ms
N
Bn
N
H
3l
0.94
2.79
3.83
1.95
2.00
3.01
3.03
3.06
8.5
8.0
7.5
7.0
6.5
6.0
5.5
5.0
4.5
4.0
3.5
3.0
2.5
2.0
1.5
1.0
0.5
0.0
f1 (ppm)

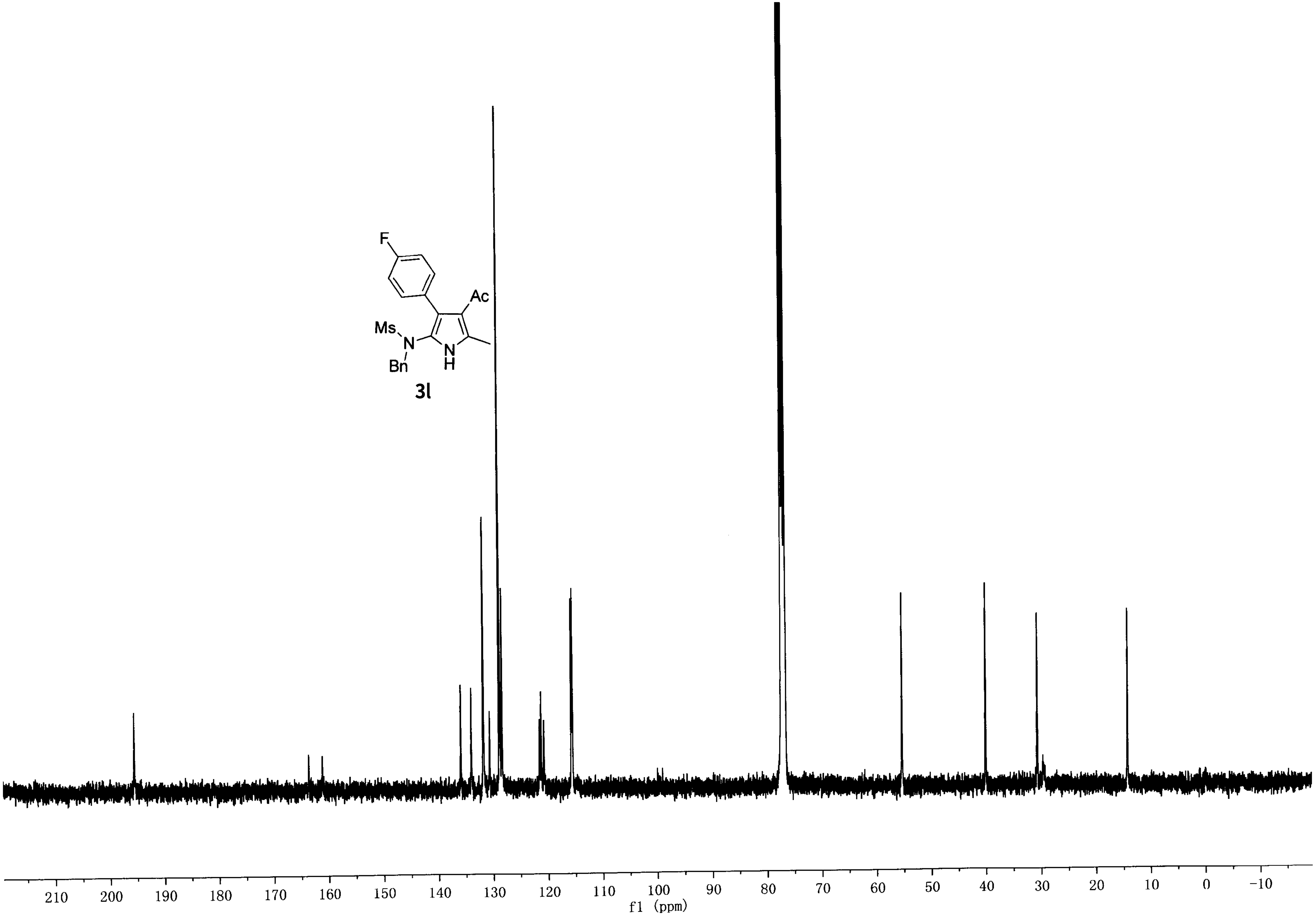
F
Ac
Ms
N
Bn
N
H
3l
210
200
190
180
170
160
150
140
130
120
110
100
90
80
70
60
50
40
30
20
10
0
-10
f1 (ppm)

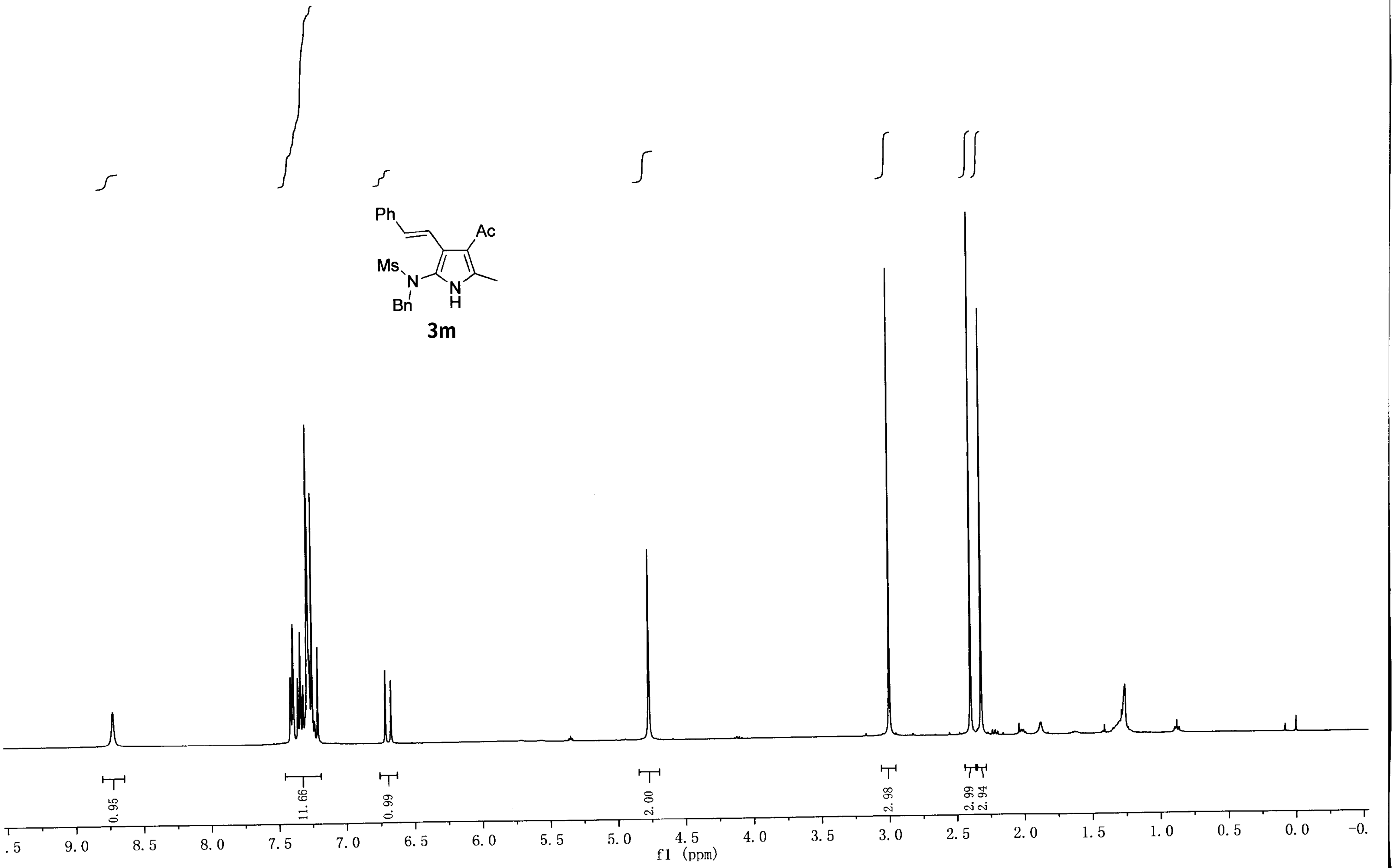
Ph
Ac
Ms
N
N
H
Bn
3m
0.95
11.66
0.99
2.00
2.98
2.99
2.94
.5
9.0
8.5
8.0
7.5
7.0
6.5
6.0
5.5
5.0
4.5
4.0
3.5
3.0
2.5
2.0
1.5
1.0
0.5
0.0
-0.
f1 (ppm)

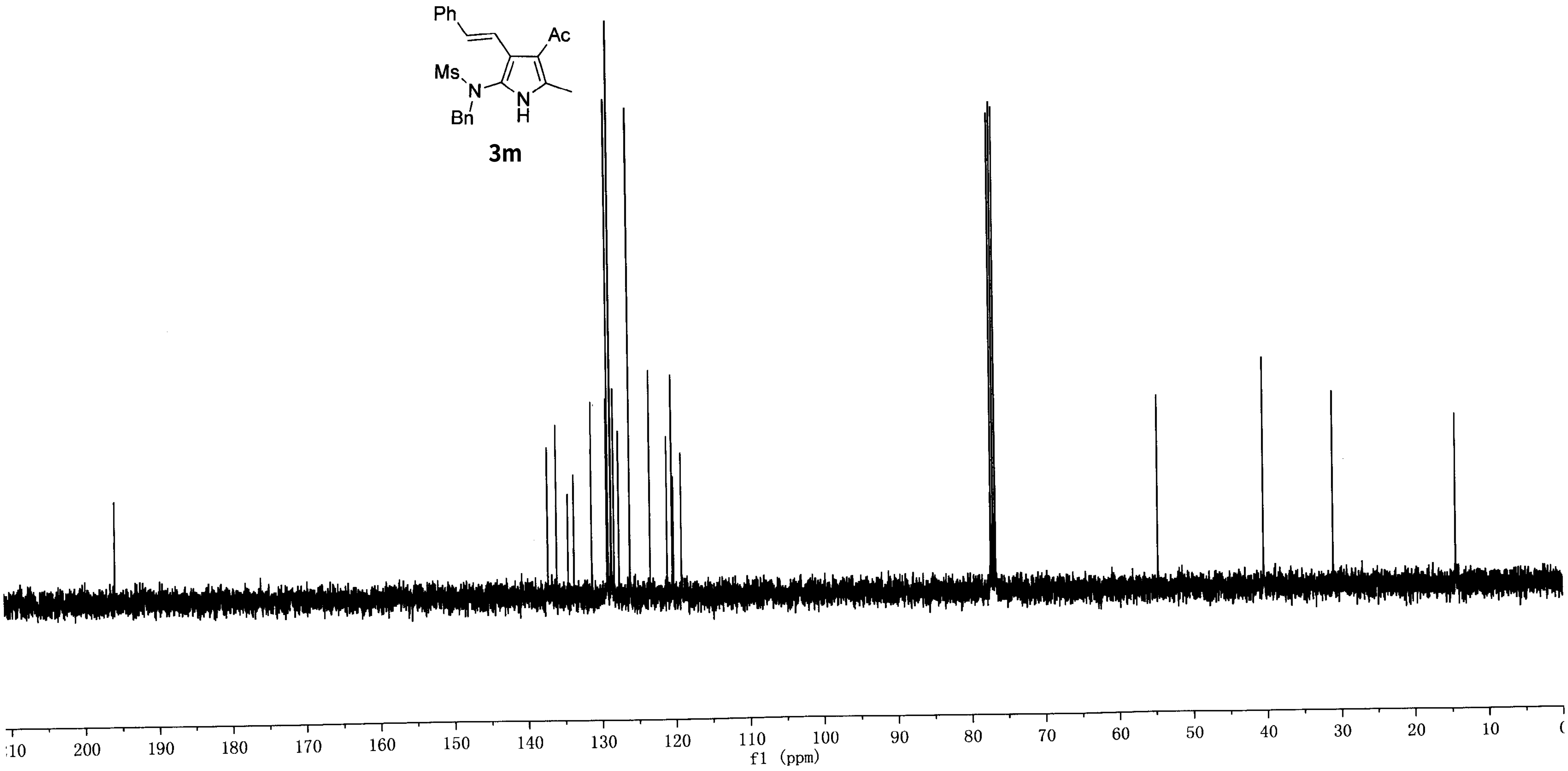
Ph
Ac
Ms
N
Bn
N
H
3m
200
190
180
170
160
150
140
130
120
110
100
90
80
70
60
50
40
30
20
10
f1 (ppm)

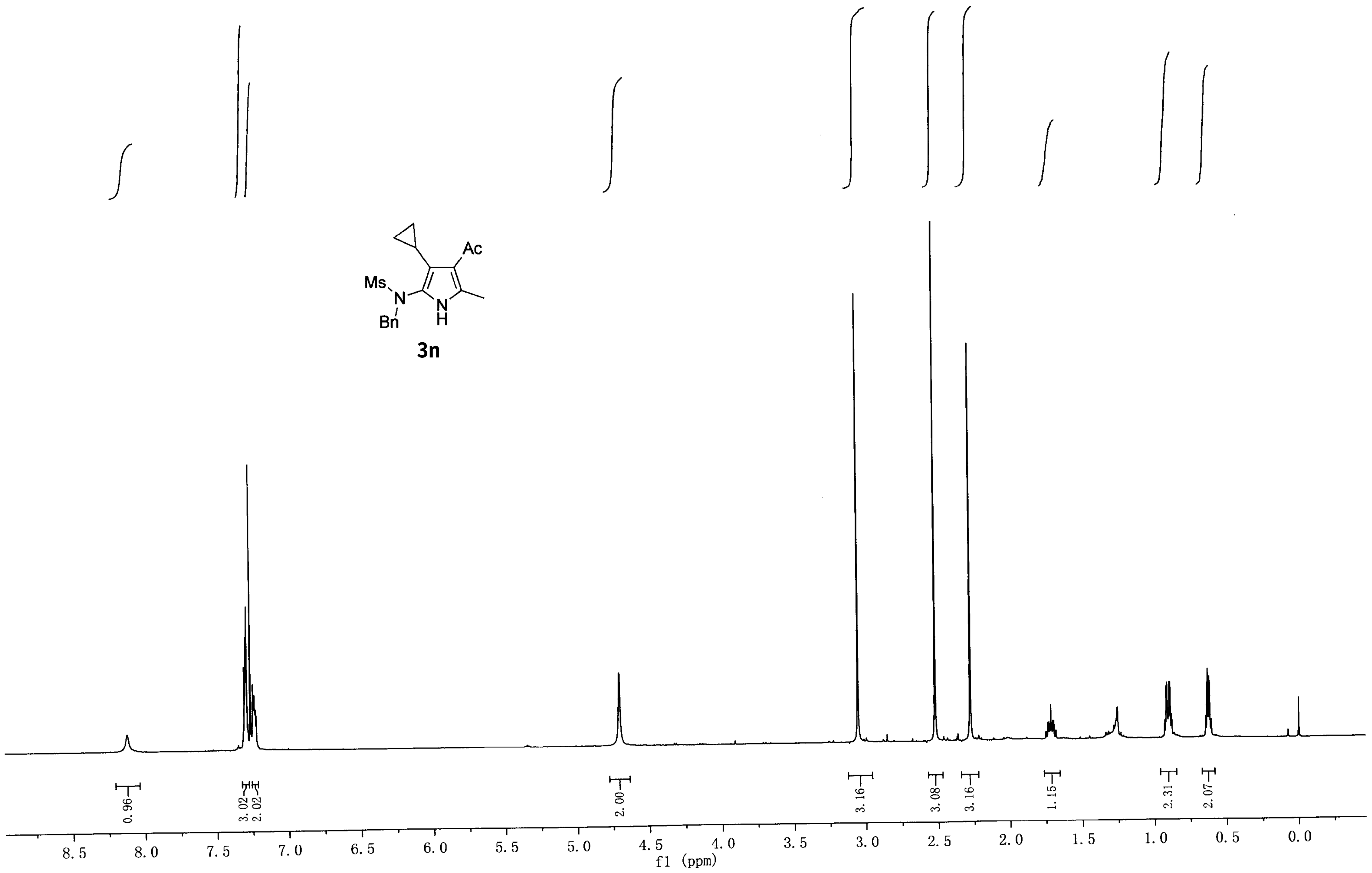
Ac
Ms
N
Bn
N
H
3n
0.96
3.02
2.02
2.00
3.16
3.08
3.16
1.15
2.31
2.07
8.5
8.0
7.5
7.0
6.5
6.0
5.5
5.0
4.5
4.0
3.5
3.0
2.5
2.0
1.5
1.0
0.5
0.0
f1 (ppm)

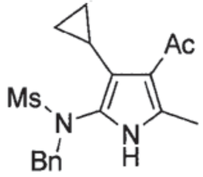
Ac
Ms
N
Bn
N
H

3n

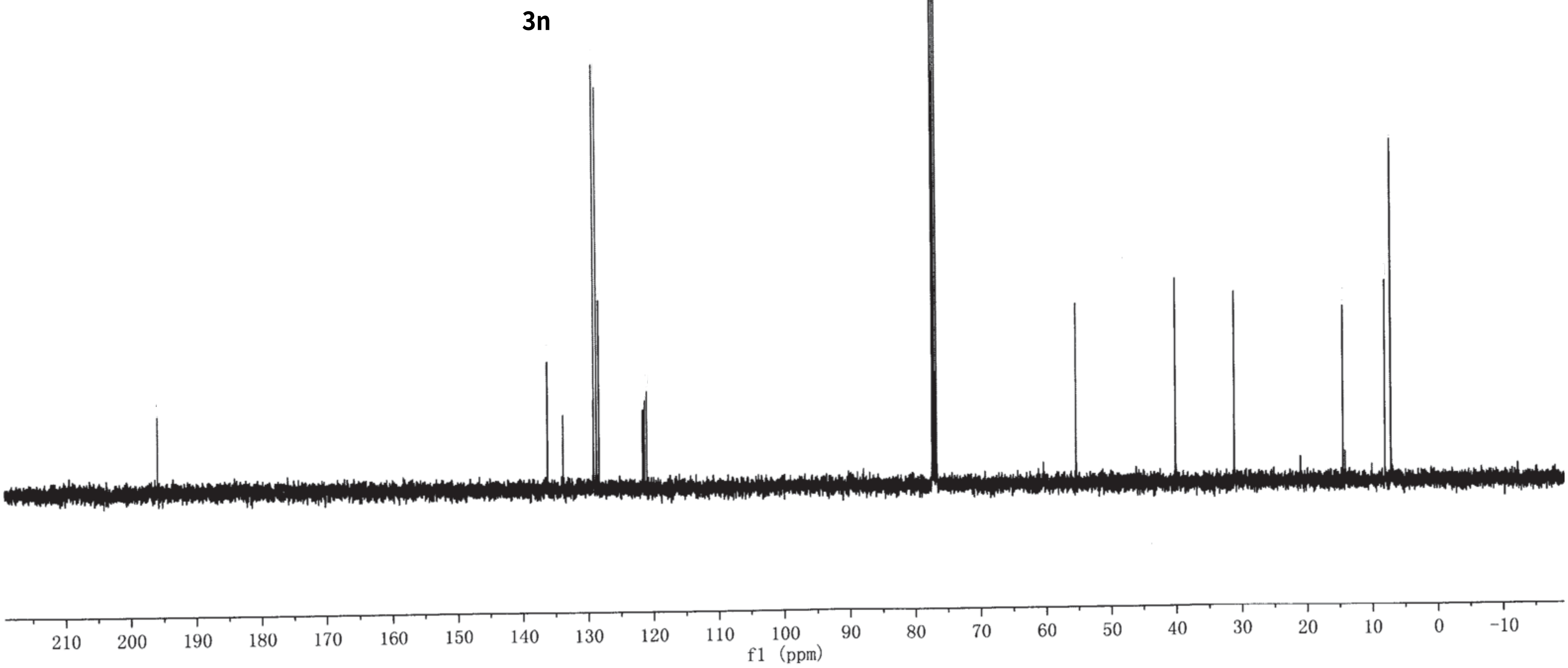
210 200 190 180 170 160 150 140 130 120 110 100 90 80 70 60 50 40 30 20 10 0 -10
f1 (ppm)

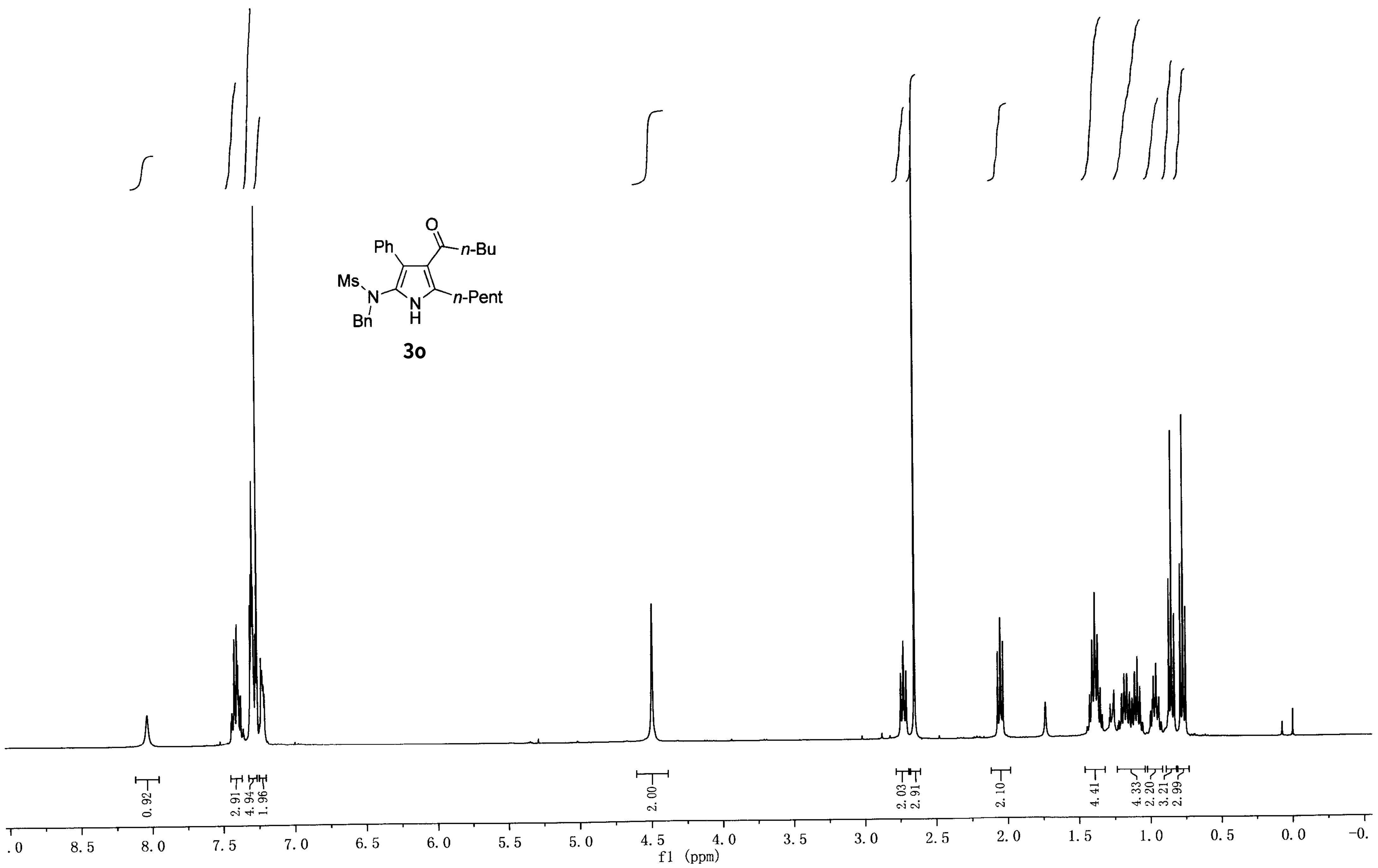

Ph
O
n-Bu
Ms
N
n-Pent
N
H
Bn
3o
0.92
2.91
4.94
1.96
2.00
2.03
2.91
2.10
4.41
4.33
2.20
3.21
2.99
8.5
8.0
7.5
7.0
6.5
6.0
5.5
5.0
4.5
4.0
3.5
3.0
2.5
2.0
1.5
1.0
0.5
0.0
f1 (ppm)

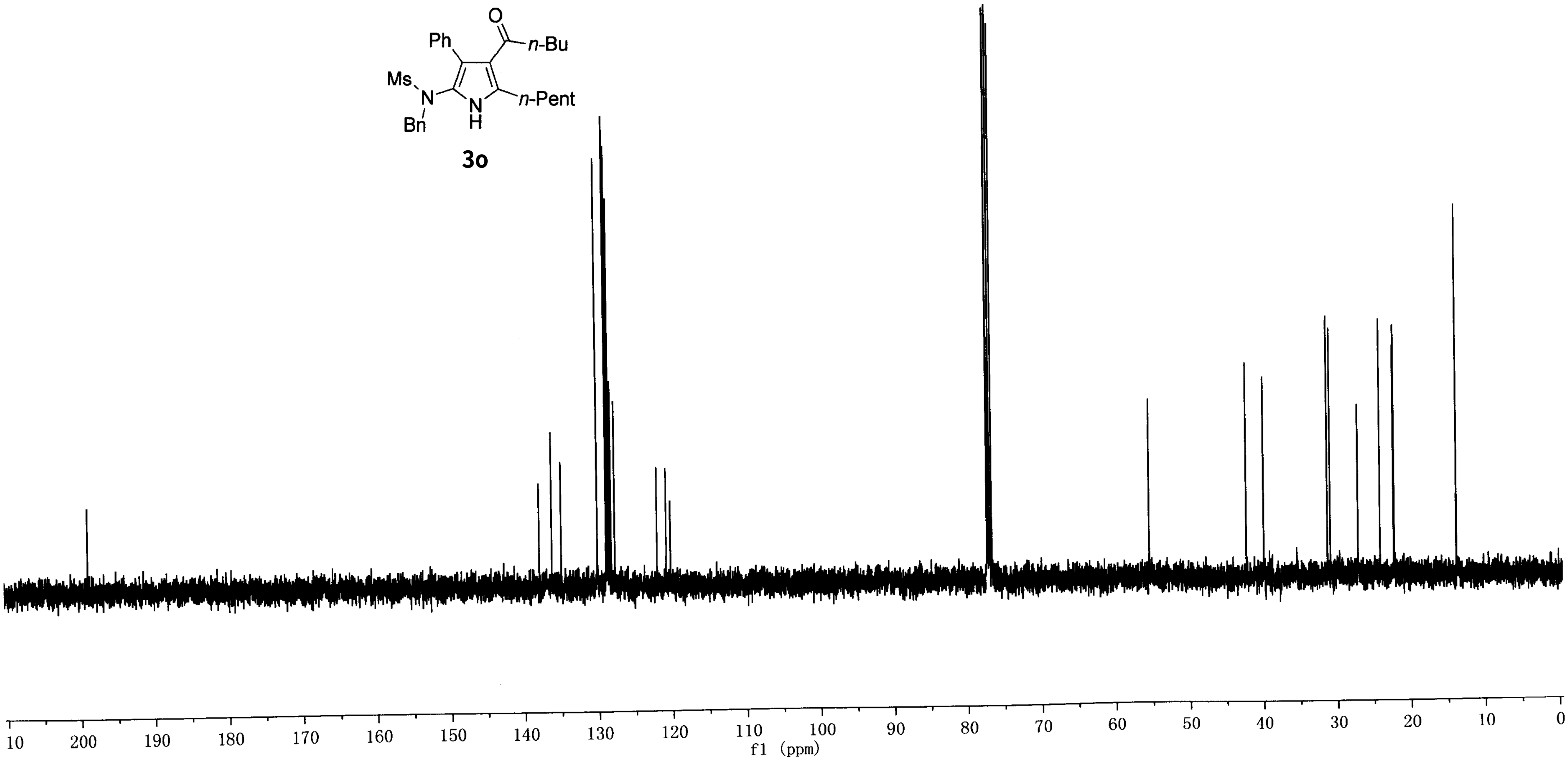
O
Ph
n-Bu
Ms
N
n-Pent
Bn
N
H
3o
10
200
190
180
170
160
150
140
130
120
110
100
90
80
70
60
50
40
30
20
10
0
f1 (ppm)

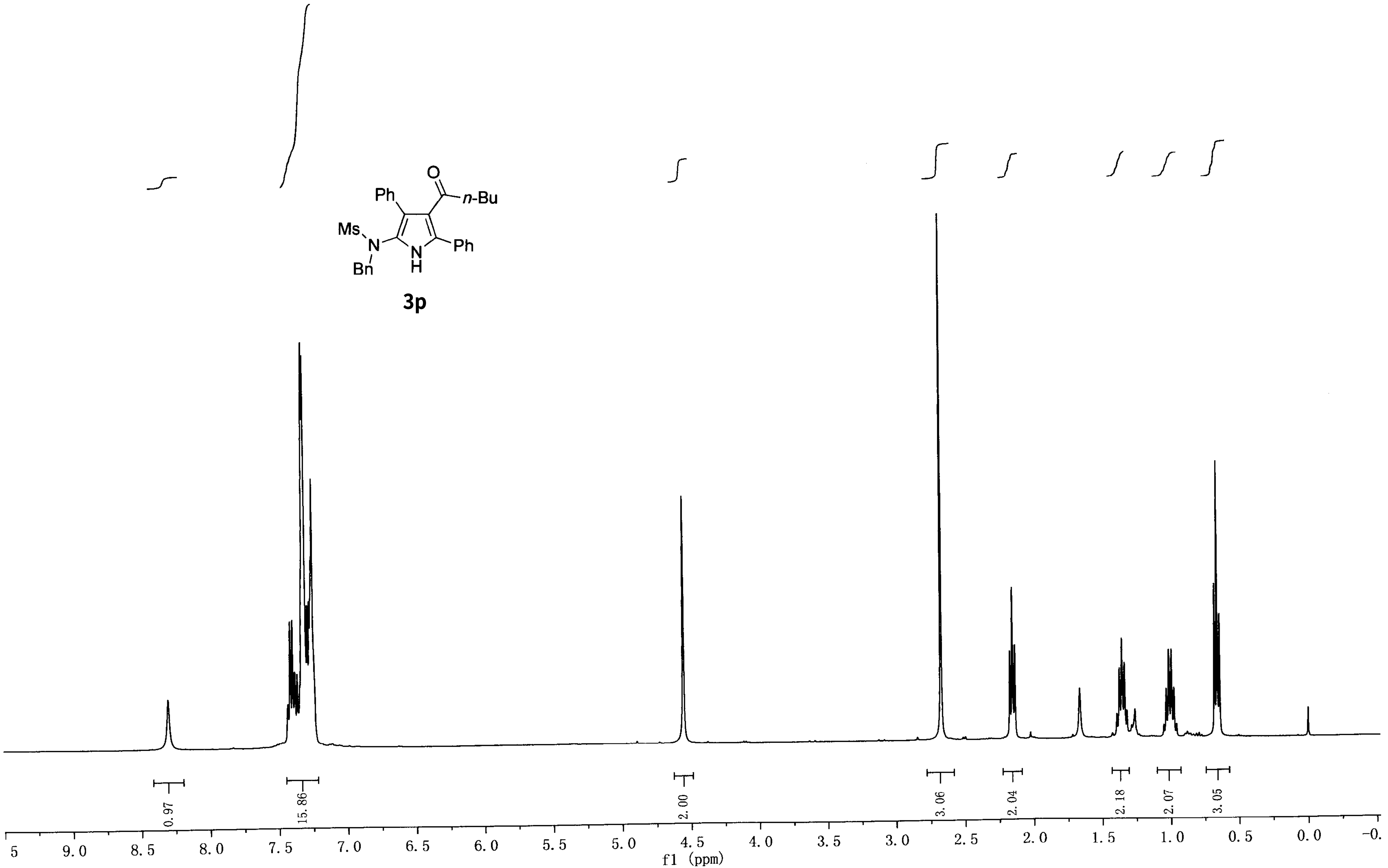

Ph
O
n-Bu
Ms
N
Ph
N
H
Bn
3p
0.97
15.86
2.00
3.06
2.04
2.18
2.07
3.05
9.0
8.5
8.0
7.5
7.0
6.5
6.0
5.5
5.0
4.5
4.0
3.5
3.0
2.5
2.0
1.5
1.0
0.5
0.0
f1 (ppm)

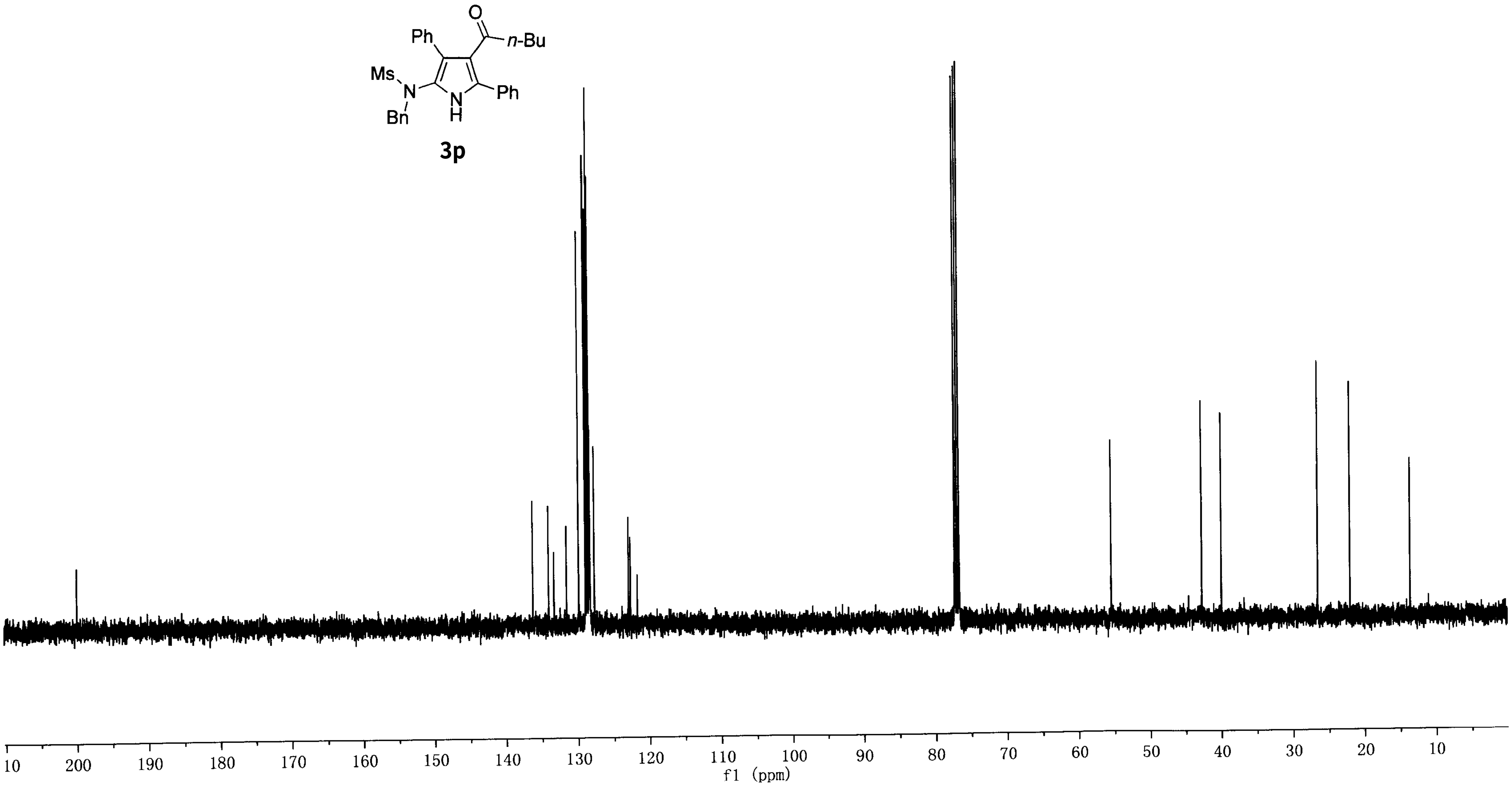
Ph
O
n-Bu
Ms
N
Bn
N
H
Ph
3p
10 200 190 180 170 160 150 140 130 120 110 100 90 80 70 60 50 40 30 20 10
f1 (ppm)

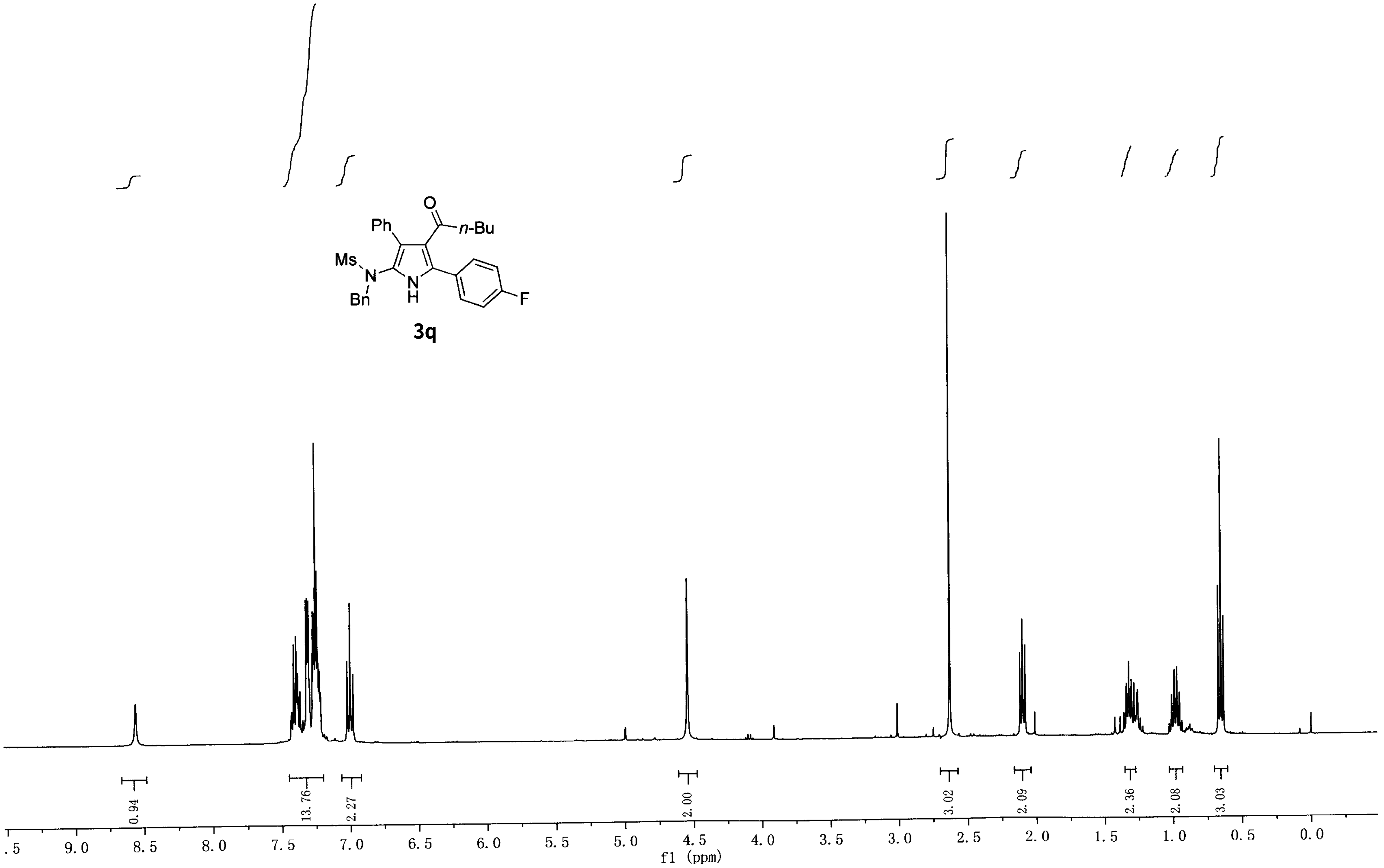
Ph
O
n-Bu
Ms
N
N
H
Bn
F
3q
0.94
13.76
2.27
2.00
3.02
2.09
2.36
2.08
3.03
.5
9.0
8.5
8.0
7.5
7.0
6.5
6.0
5.5
5.0
4.5
4.0
3.5
3.0
2.5
2.0
1.5
1.0
0.5
0.0
f1 (ppm)

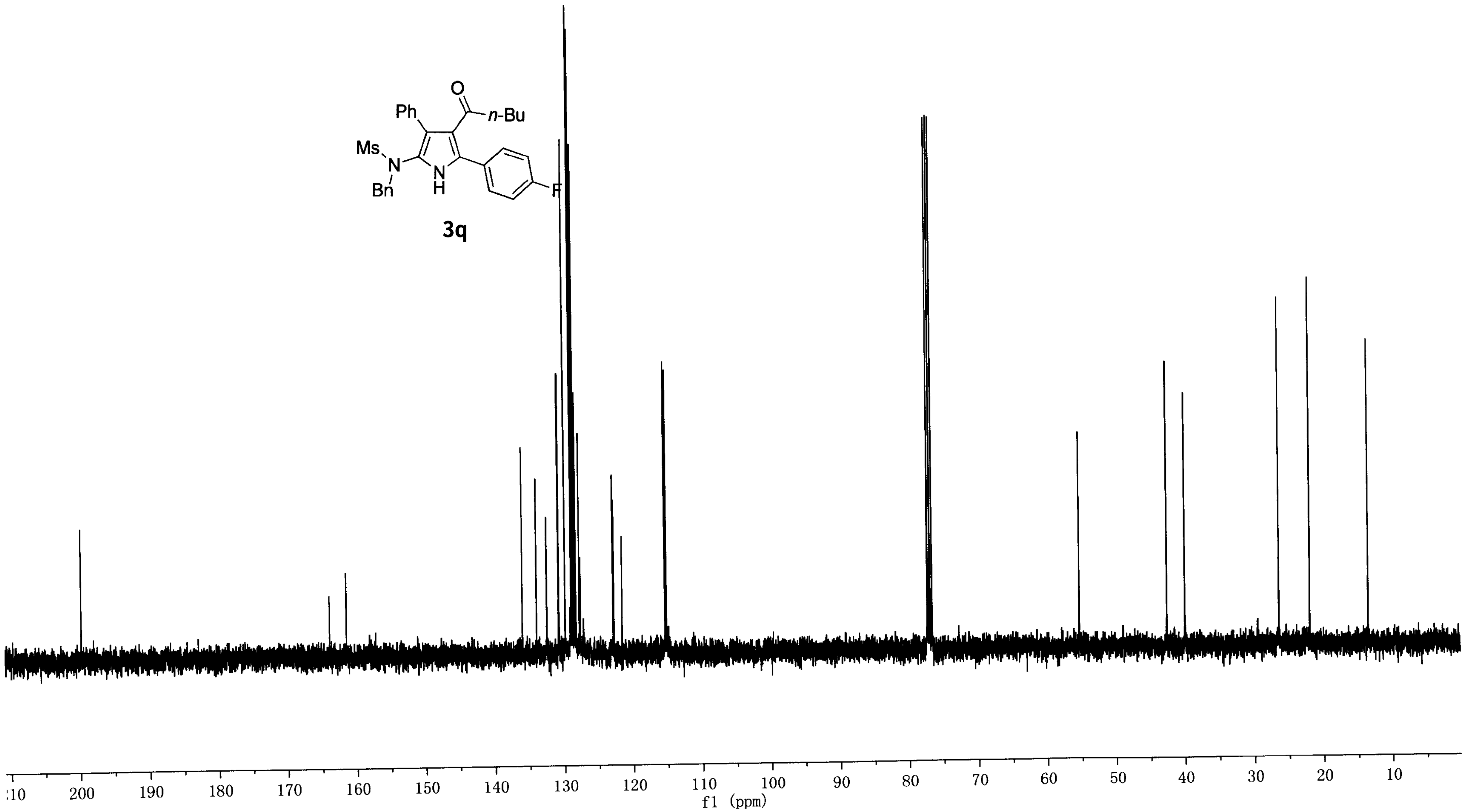
Ph
O
n-Bu
Ms
N
Bn
N
H
F
3q
210
200
190
180
170
160
150
140
130
120
110
100
90
80
70
60
50
40
30
20
10
f1 (ppm)

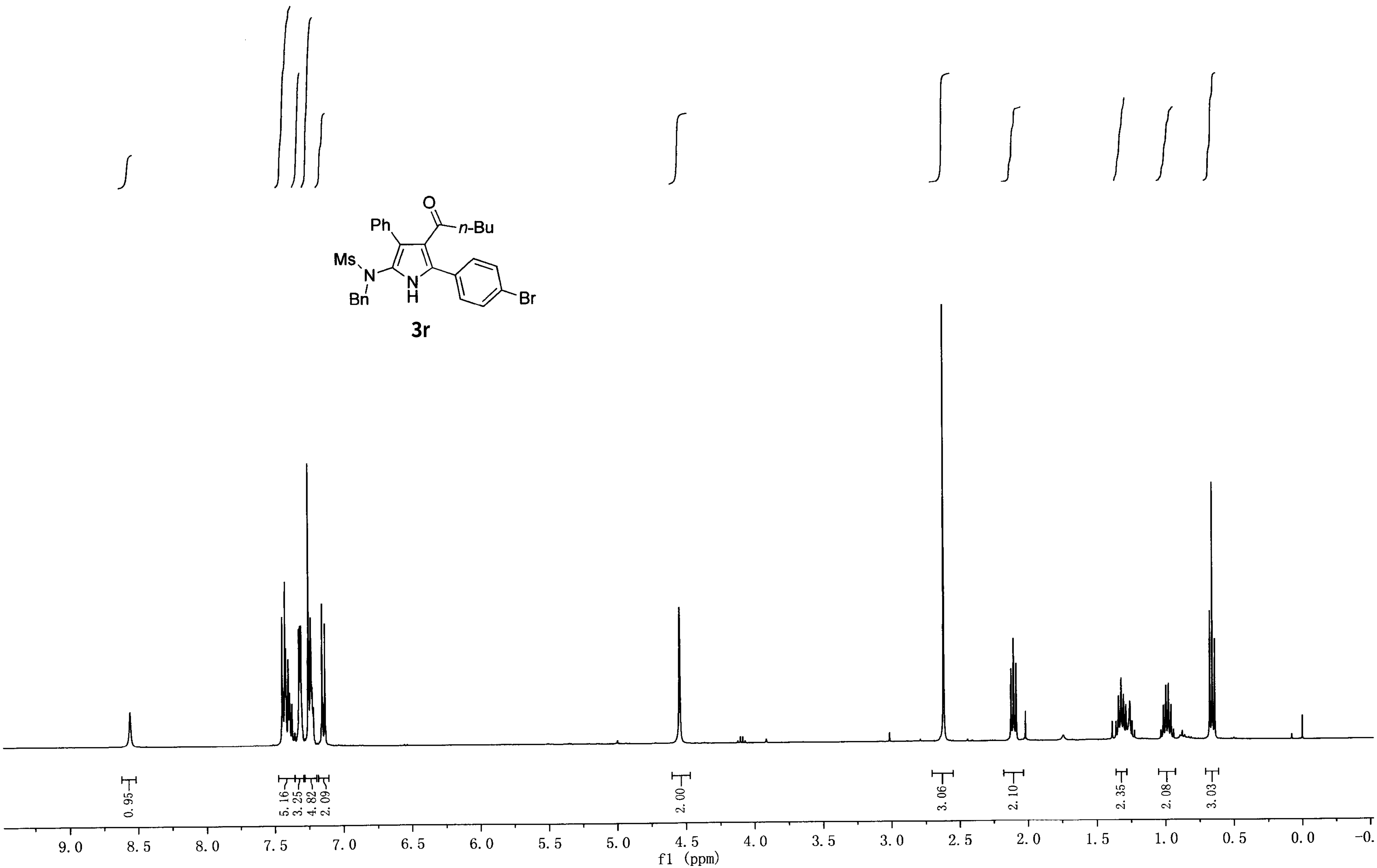

Ph
O
n-Bu
Ms
N
Bn
N
H
Br
3r
0.95
5.16
3.25
4.82
2.09
2.00
3.06
2.10
2.35
2.08
3.03
9.0
8.5
8.0
7.5
7.0
6.5
6.0
5.5
5.0
4.5
4.0
3.5
3.0
2.5
2.0
1.5
1.0
0.5
0.0
-0.
f1 (ppm)

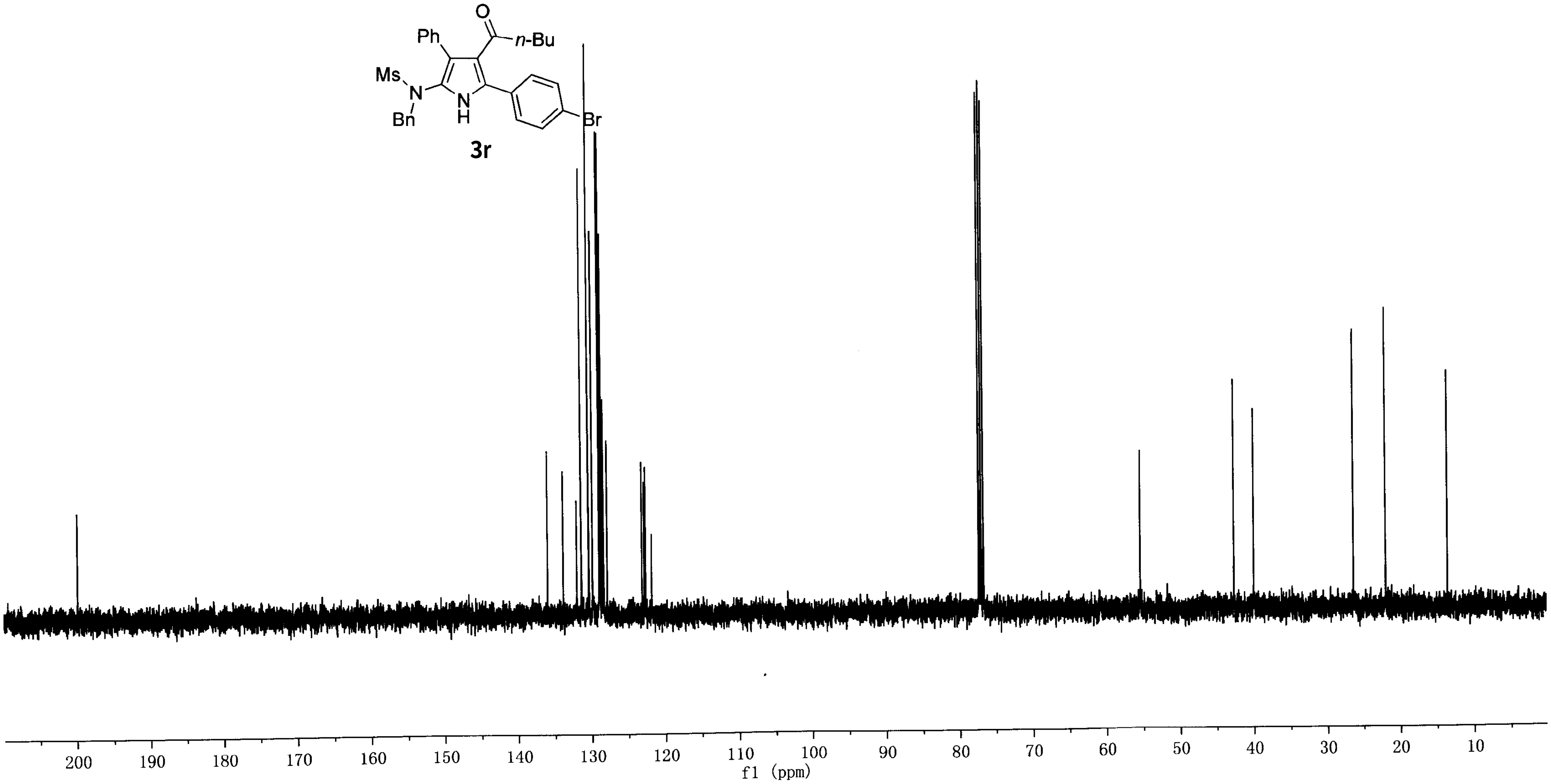
Ph
O
n-Bu
Ms
N
Bn
N
H
Br
200
190
180
170
160
150
140
130
120
110
100
90
80
70
60
50
40
30
20
10
f1 (ppm)

3r

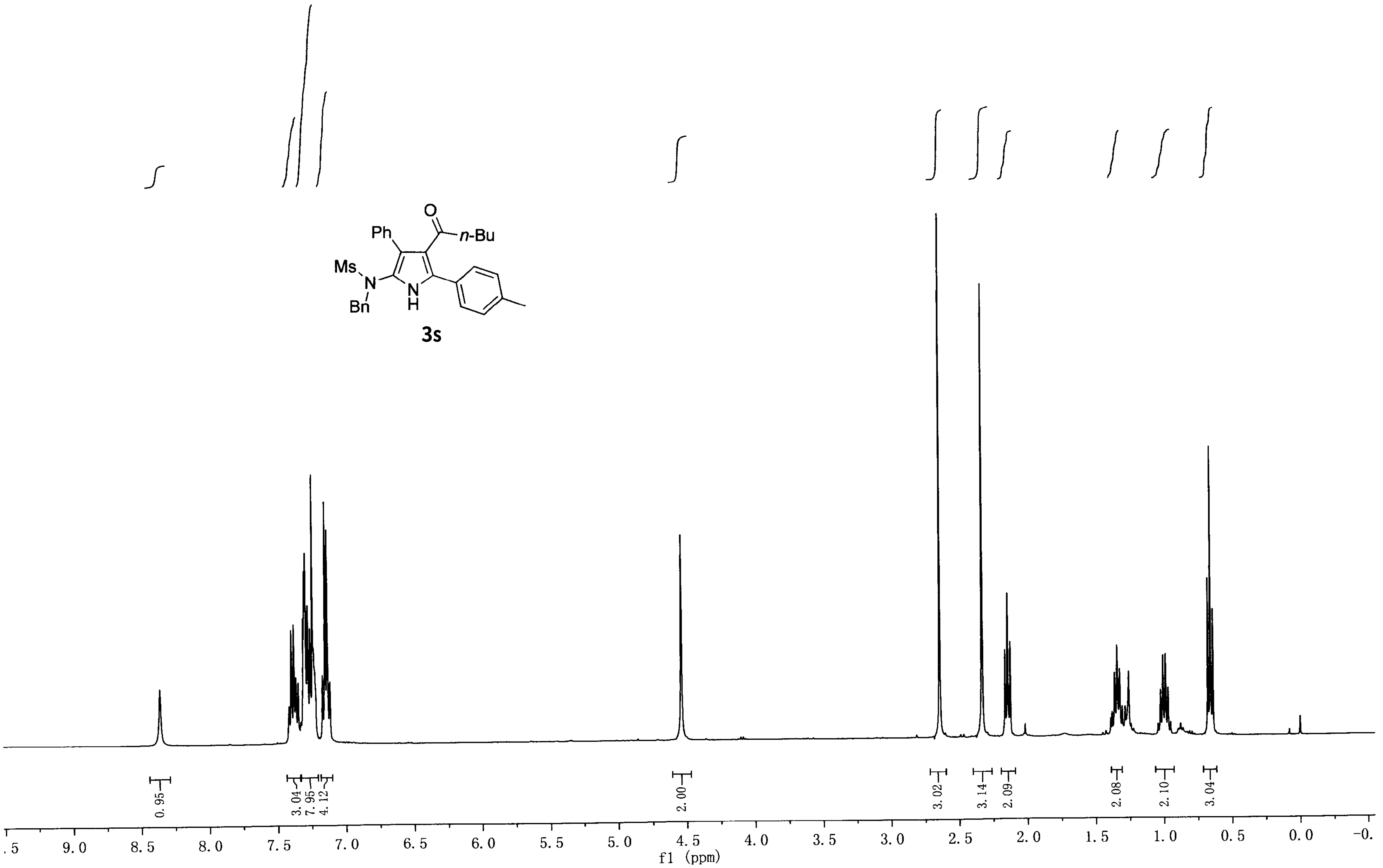

Ph
O
n-Bu
Ms
N
Bn
N
H
3s
0.95
3.04
7.95
4.12
2.00
3.02
3.14
2.09
2.08
2.10
3.04
.5
9.0
8.5
8.0
7.5
7.0
6.5
6.0
5.5
5.0
4.5
4.0
3.5
3.0
2.5
2.0
1.5
1.0
0.5
0.0
-0.
f1 (ppm)

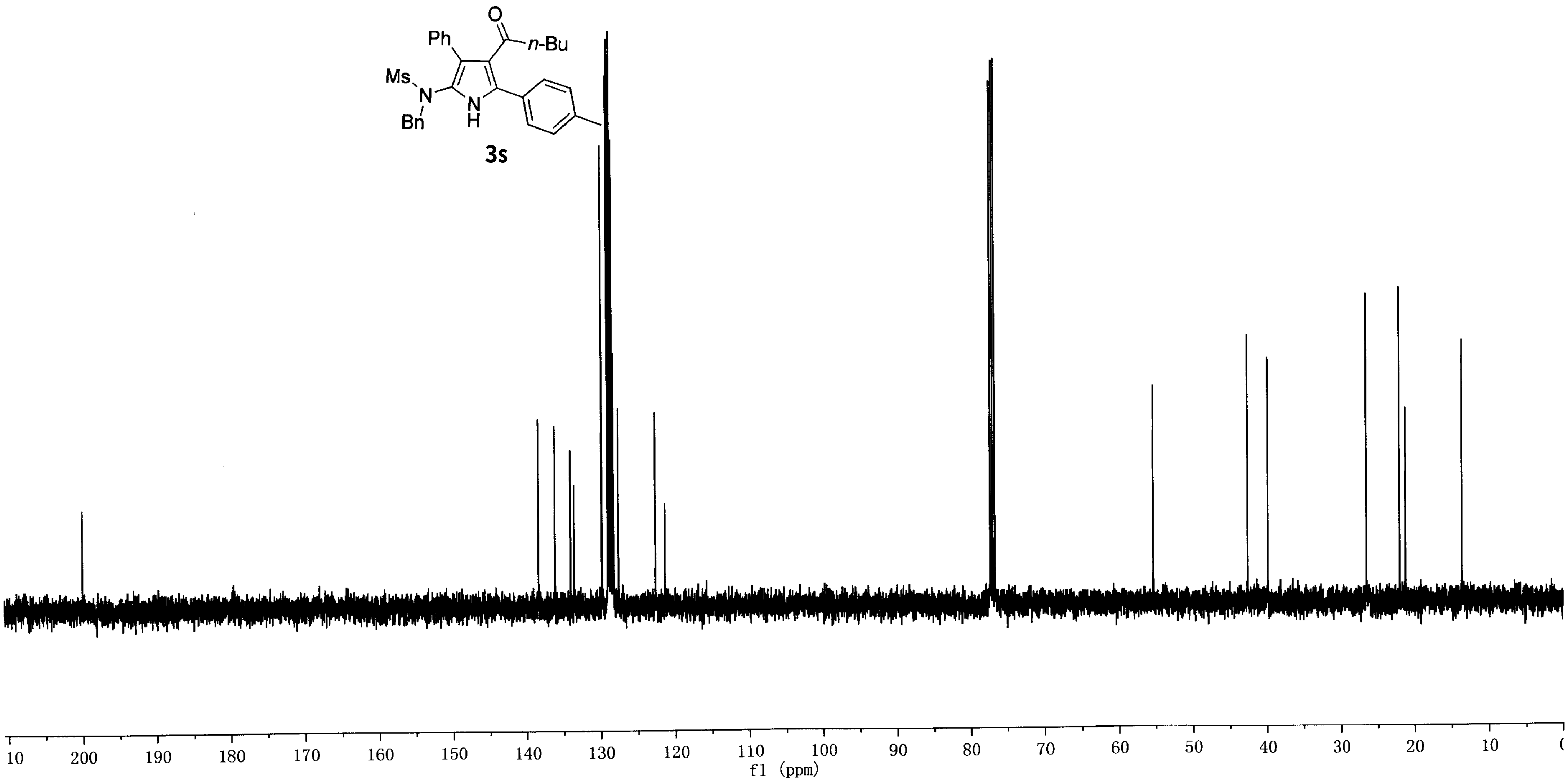

O
Ph
n-Bu
Ms
N
Bn
N
H
3s
10
200
190
180
170
160
150
140
130
120
110
100
90
80
70
60
50
40
30
20
10
f1 (ppm)

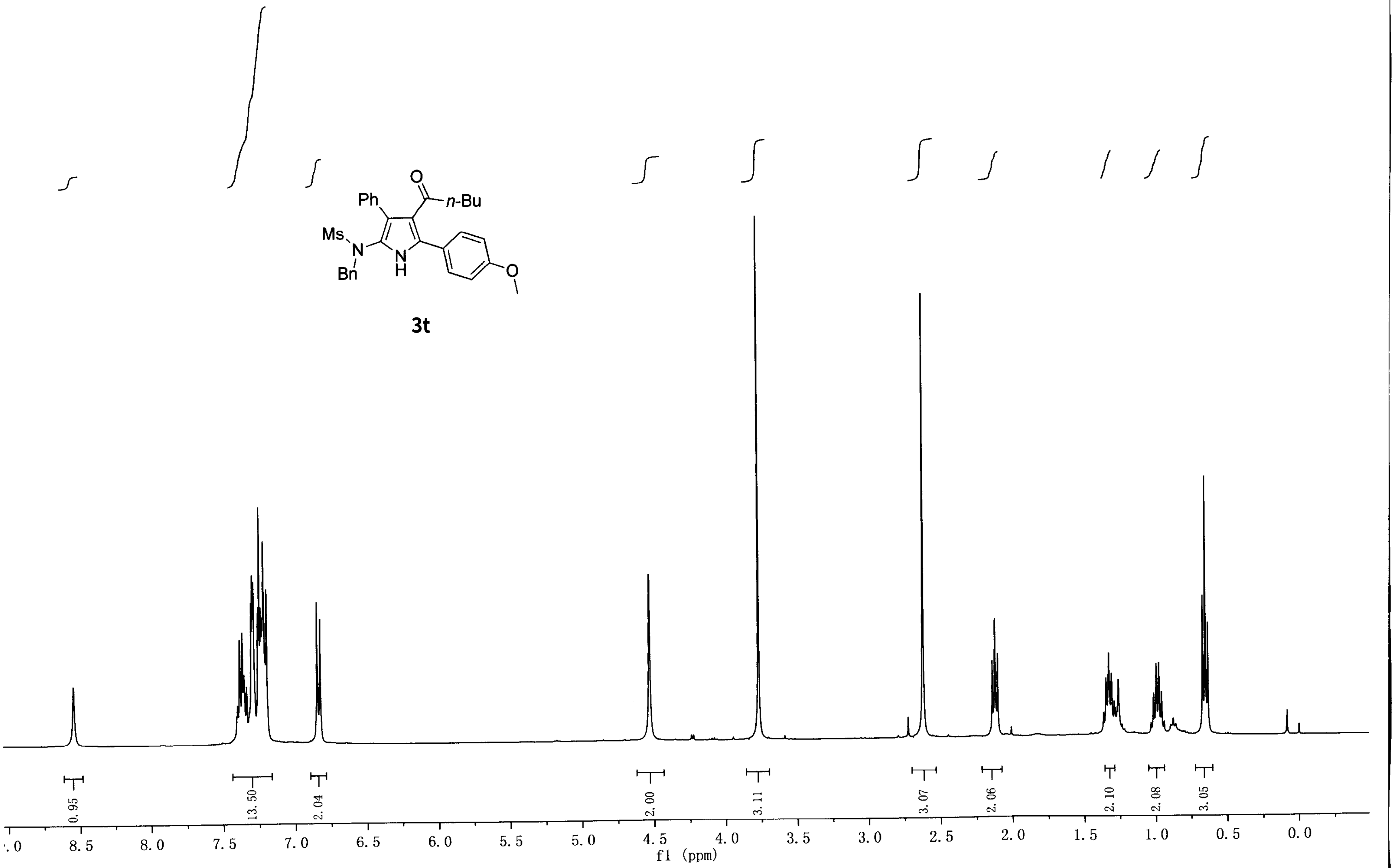
Ph
O
n-Bu
Ms
N
Bn
N
H
O
3t
0.95
13.50
2.04
2.00
3.11
3.07
2.06
2.10
2.08
3.05
8.5
8.0
7.5
7.0
6.5
6.0
5.5
5.0
4.5
4.0
3.5
3.0
2.5
2.0
1.5
1.0
0.5
0.0
f1 (ppm)

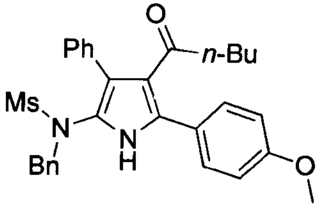
Ph
O
n-Bu
Ms
N
N
H
Bn
O

3t

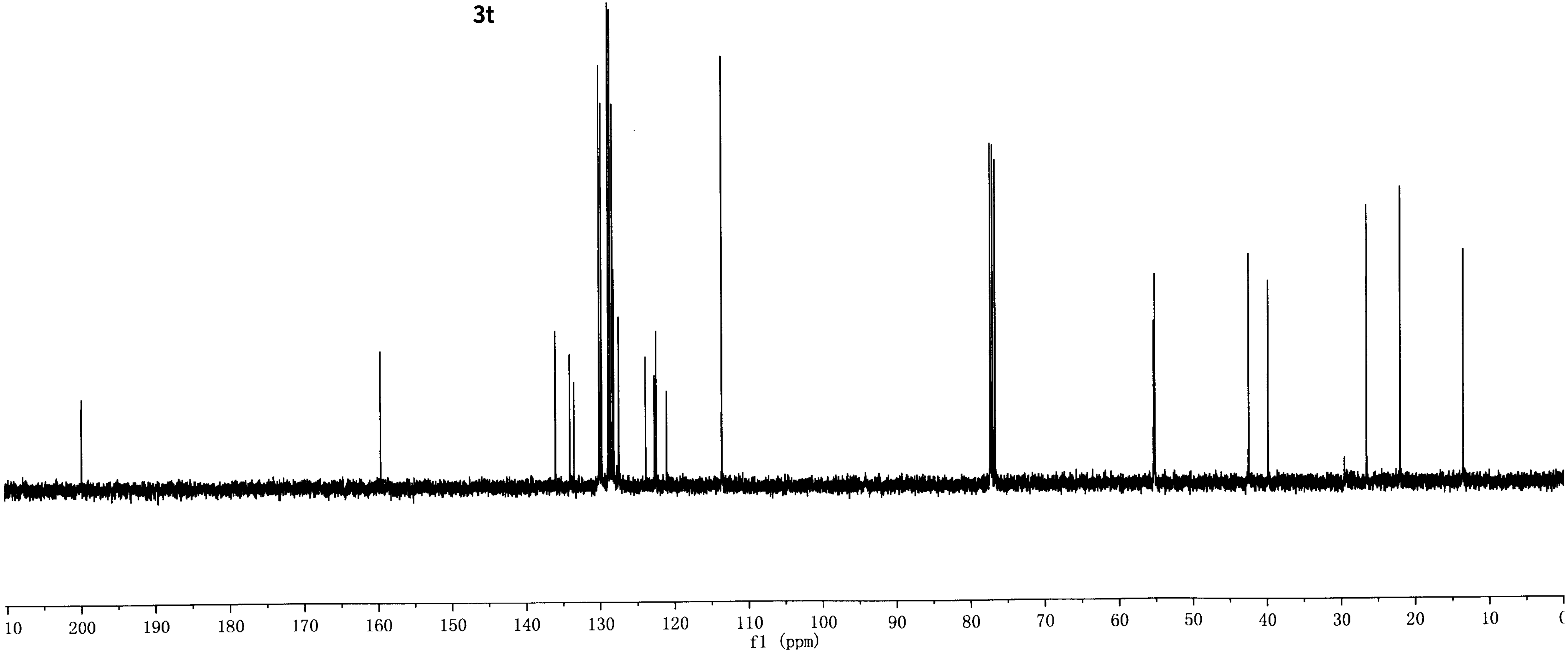
10 200 190 180 170 160 150 140 130 120 110 100 90 80 70 60 50 40 30 20 10
f1 (ppm)

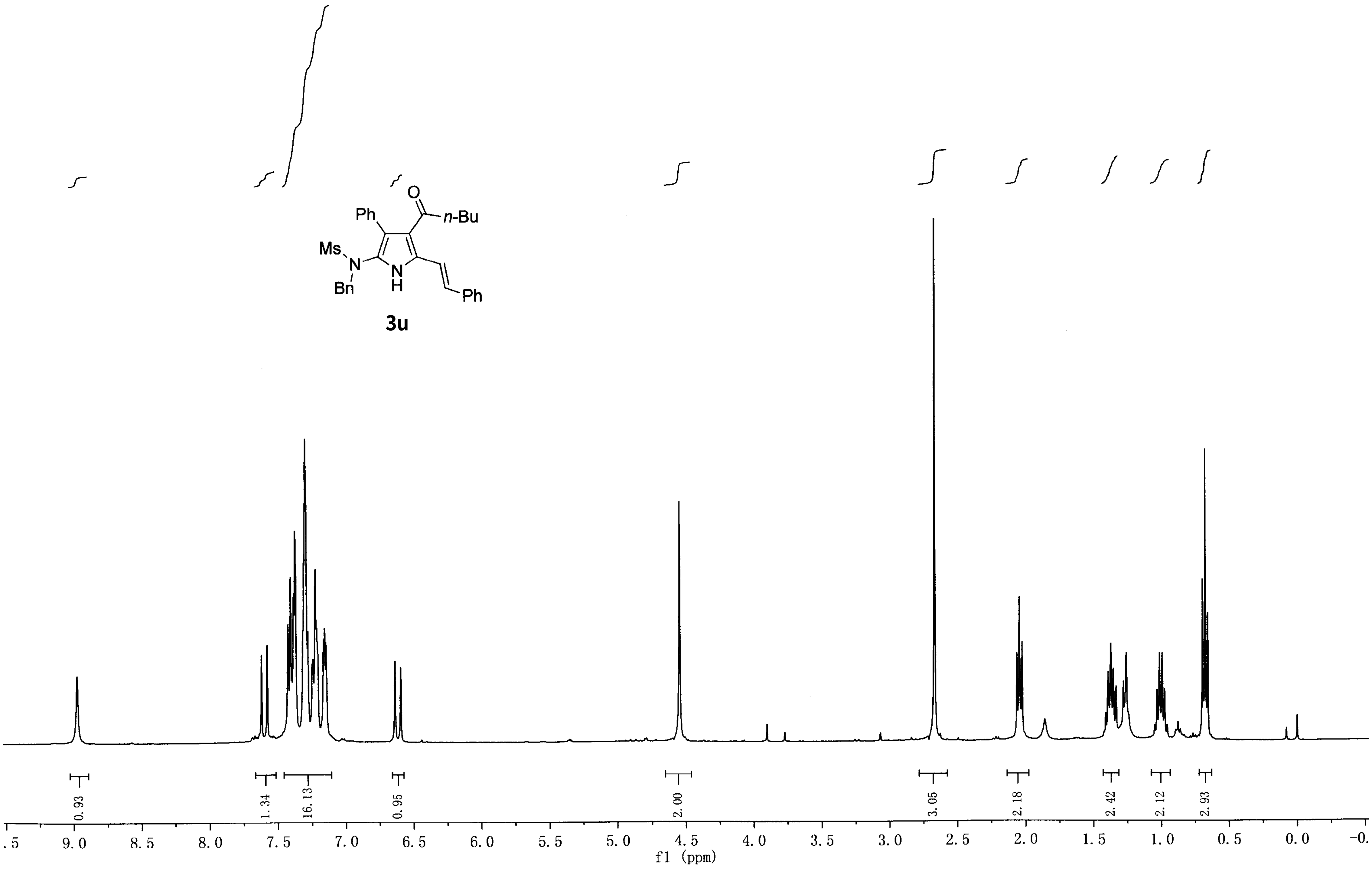

Ph
O
n-Bu
Ms
N
Bn
N
H
Ph
0.93
1.34
16.13
0.95
2.00
3.05
2.18
2.42
2.12
2.93
9.0
8.5
8.0
7.5
7.0
6.5
6.0
5.5
5.0
4.5
4.0
3.5
3.0
2.5
2.0
1.5
1.0
0.5
0.0
f1 (ppm)

3u

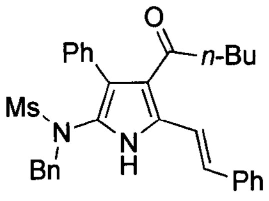
O
Ph
n-Bu
Ms
N
N
H
Bn
Ph

3u

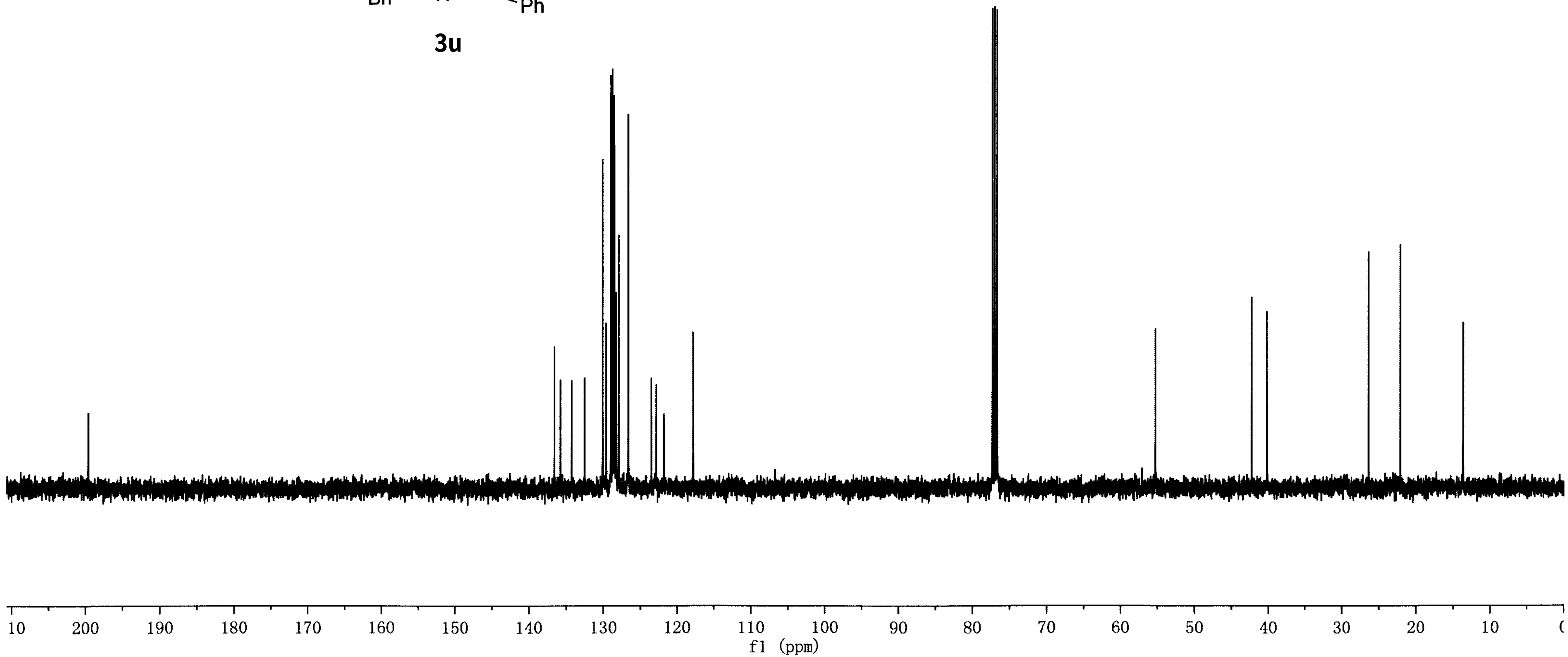
10
200
190
180
170
160
150
140
130
120
110
100
90
80
70
60
50
40
30
20
10
f1 (ppm)

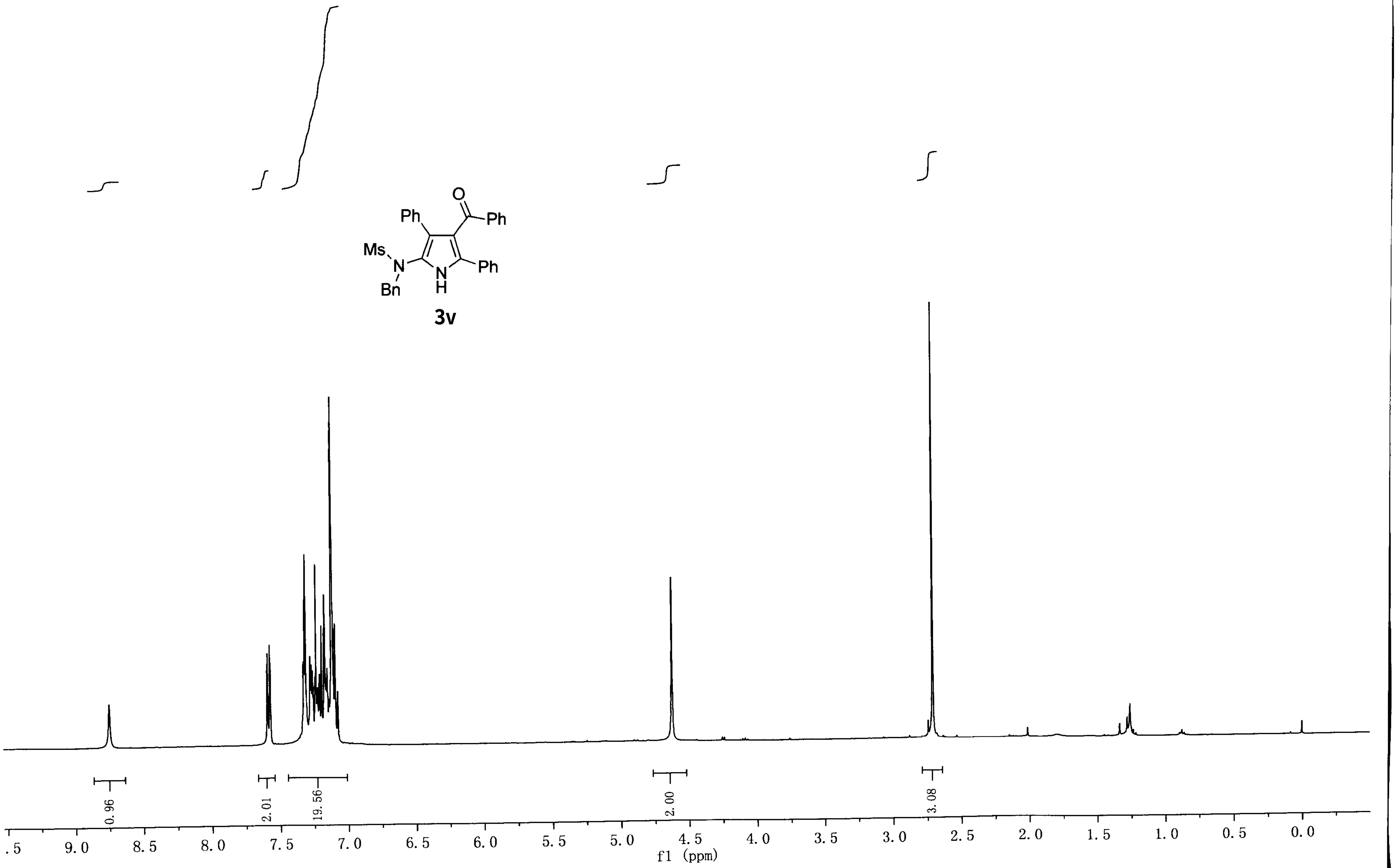

Ph
O
Ph
Ms
N
N
H
Ph
Bn
3v
0.96
2.01
19.56
2.00
3.08
.5
9.0
8.5
8.0
7.5
7.0
6.5
6.0
5.5
5.0
4.5
4.0
3.5
3.0
2.5
2.0
1.5
1.0
0.5
0.0
f1 (ppm)

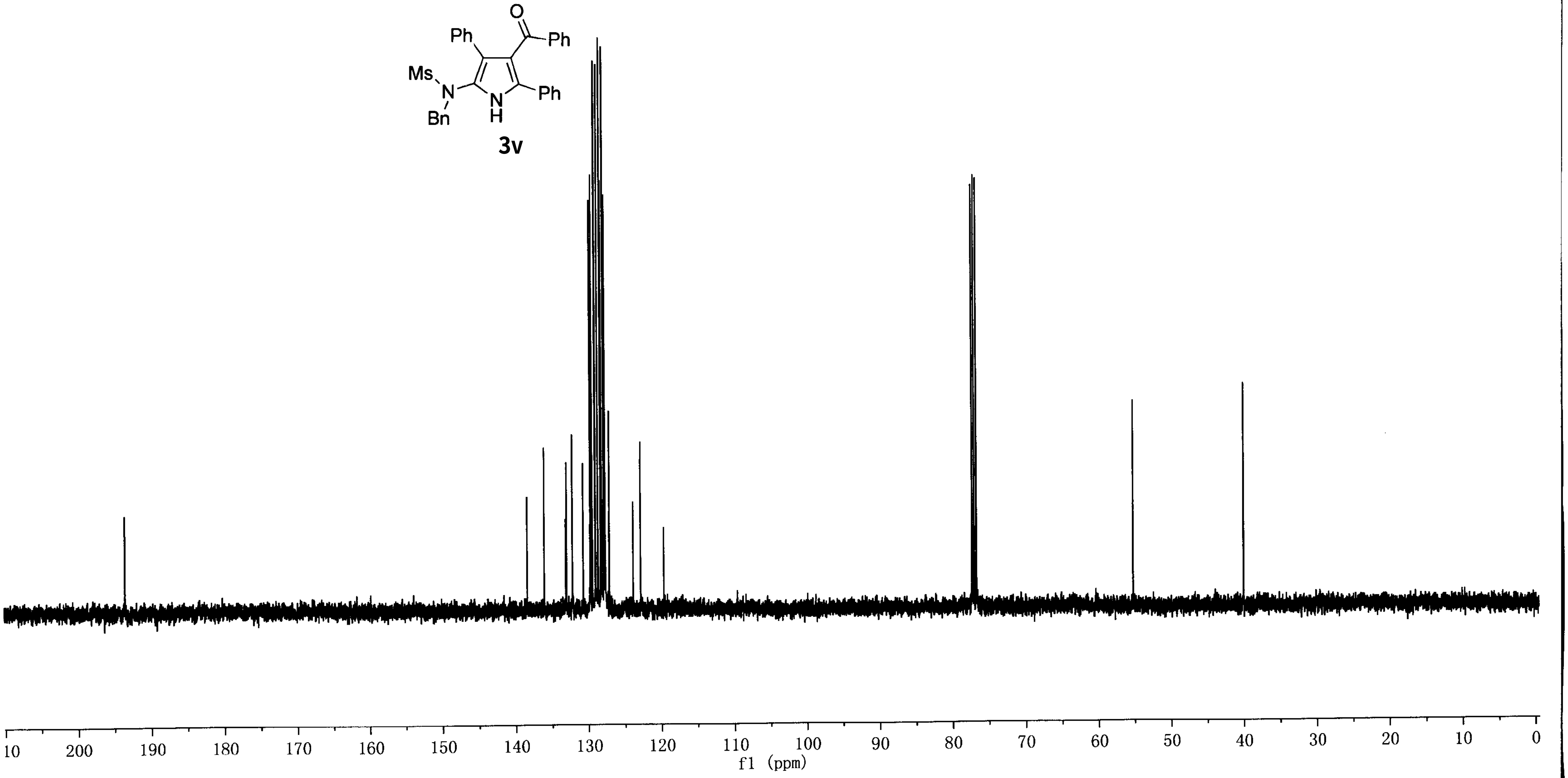
O
Ph
Ph
Ms
N
Bn
N
H
Ph
3v
10
200
190
180
170
160
150
140
130
120
110
100
90
80
70
60
50
40
30
20
10
0
f1 (ppm)

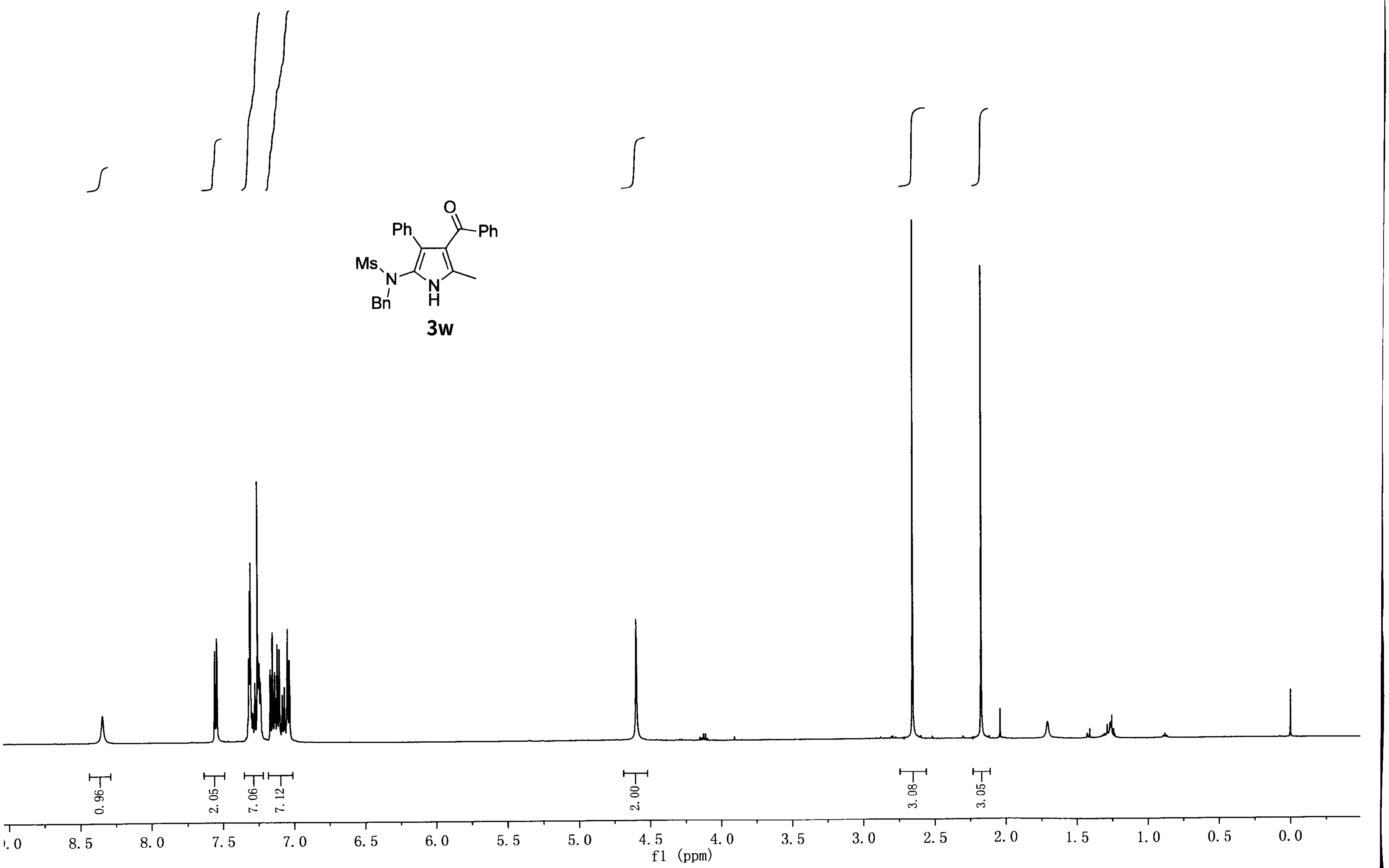

Ph
O
Ph
Ms
N
Bn
N
H
3w
0.96
2.05
7.06
7.12
2.00
3.08
3.05
8.5
8.0
7.5
7.0
6.5
6.0
5.5
5.0
4.5
4.0
3.5
3.0
2.5
2.0
1.5
1.0
0.5
0.0
f1 (ppm)

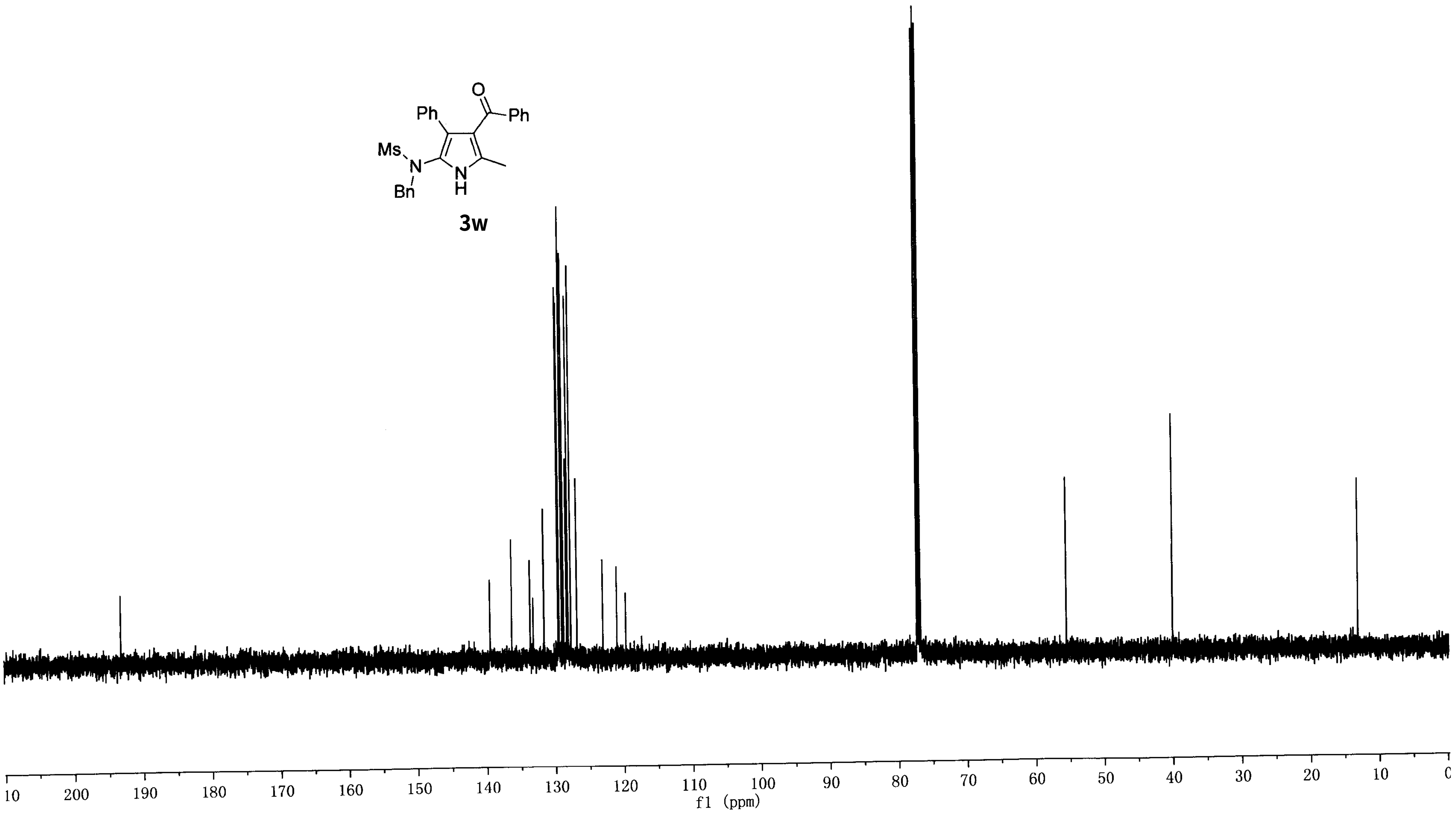

O
Ph
Ph
Ms
N
Bn
N
H
3w
10
200
190
180
170
160
150
140
130
120
110
100
90
80
70
60
50
40
30
20
10
C
f1 (ppm)

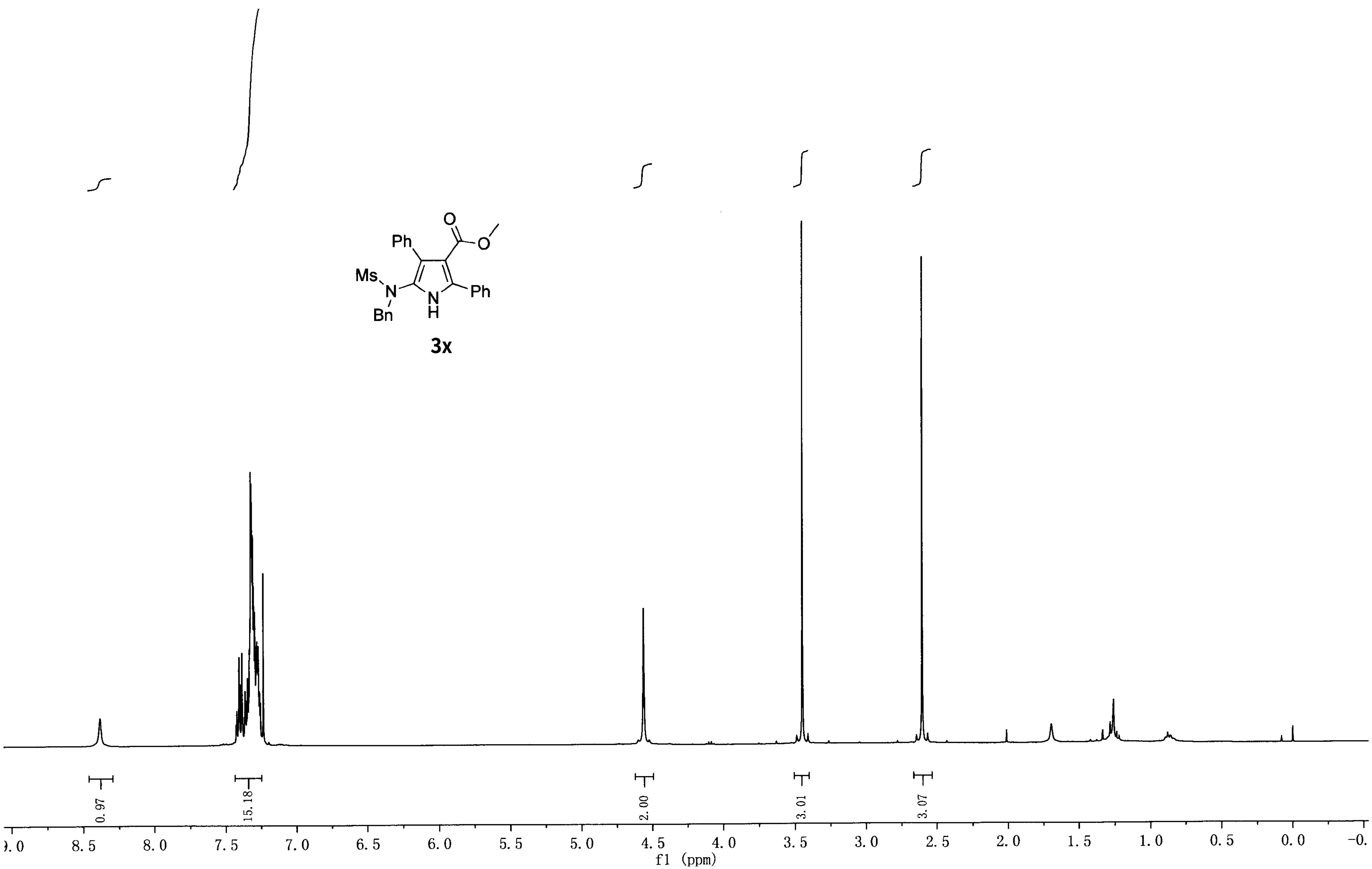

Ph
O
O
Ms
N
Ph
N
H
Bn
3x
0.97
15.18
2.00
3.01
3.07
8.5
8.0
7.5
7.0
6.5
6.0
5.5
5.0
4.5
4.0
3.5
3.0
2.5
2.0
1.5
1.0
0.5
0.0
f1 (ppm)

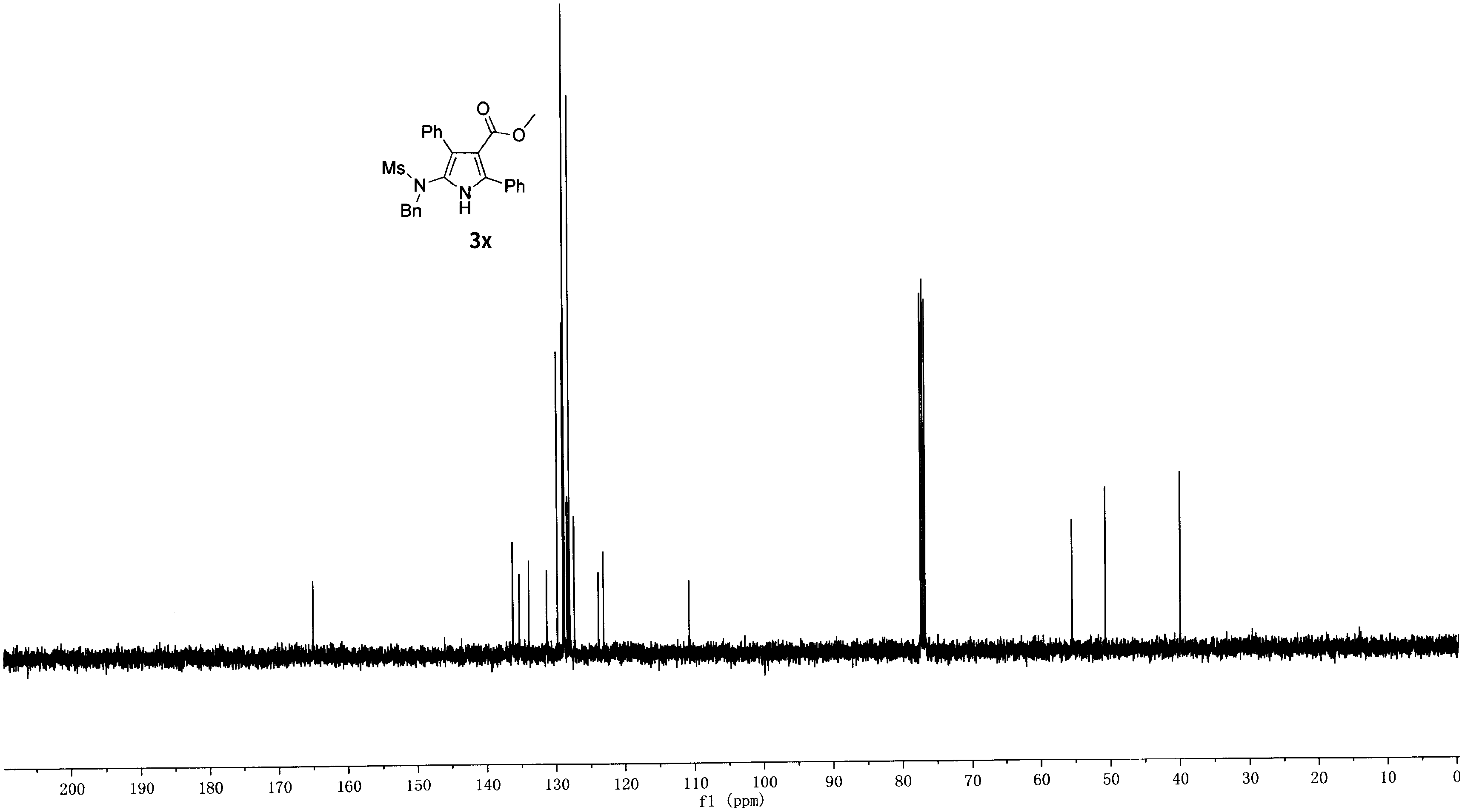

Ph
O
O
Ms
N
Ph
N
H
Bn
3x
200
190
180
170
160
150
140
130
120
110
100
90
80
70
60
50
40
30
20
10
0
f1 (ppm)

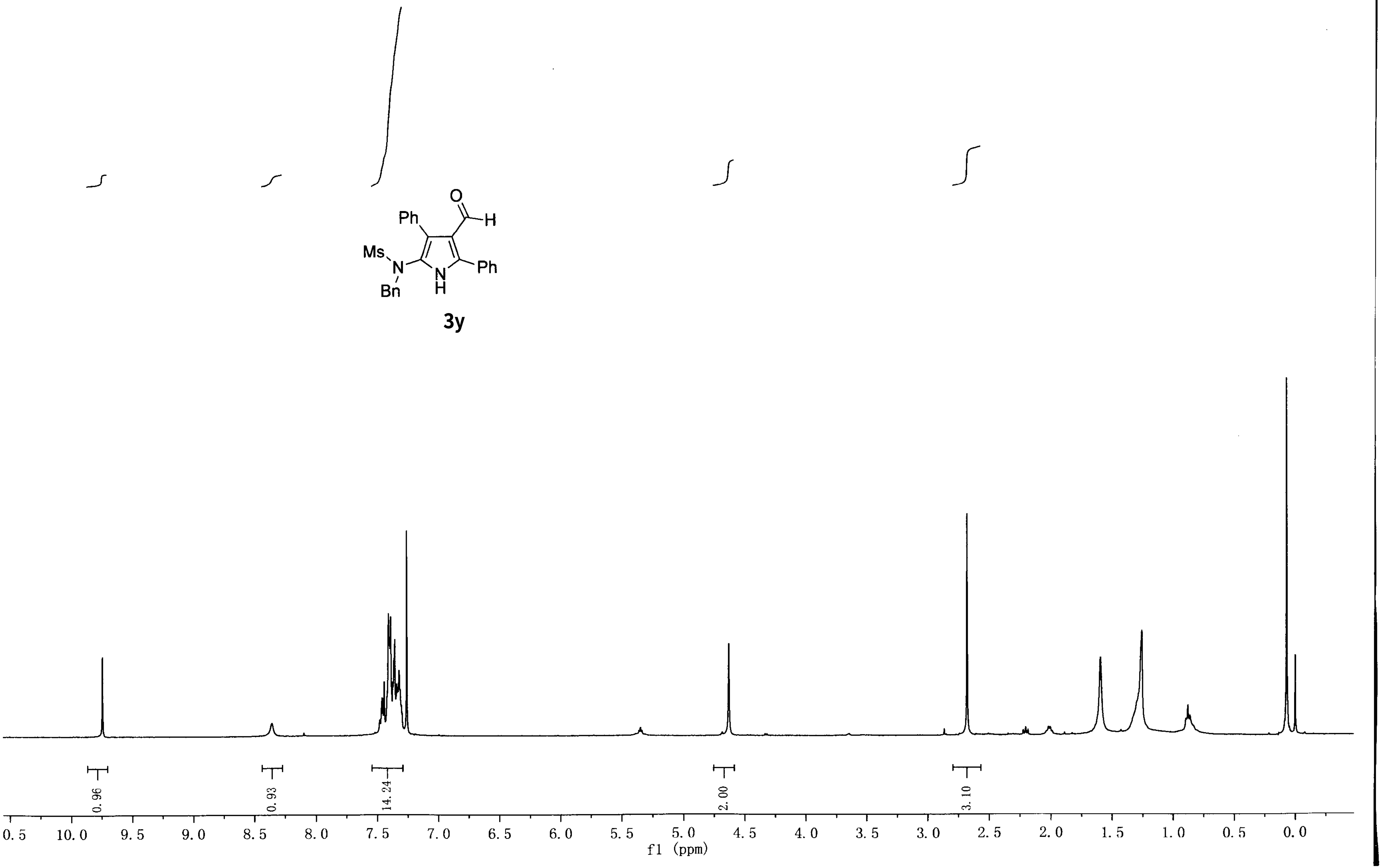
Ph
O
H
Ms
N
Ph
Bn
N
H
3y
0.96
0.93
14.24
2.00
3.10
0.5
10.0
9.5
9.0
8.5
8.0
7.5
7.0
6.5
6.0
5.5
5.0
4.5
4.0
3.5
3.0
2.5
2.0
1.5
1.0
0.5
0.0
f1 (ppm)

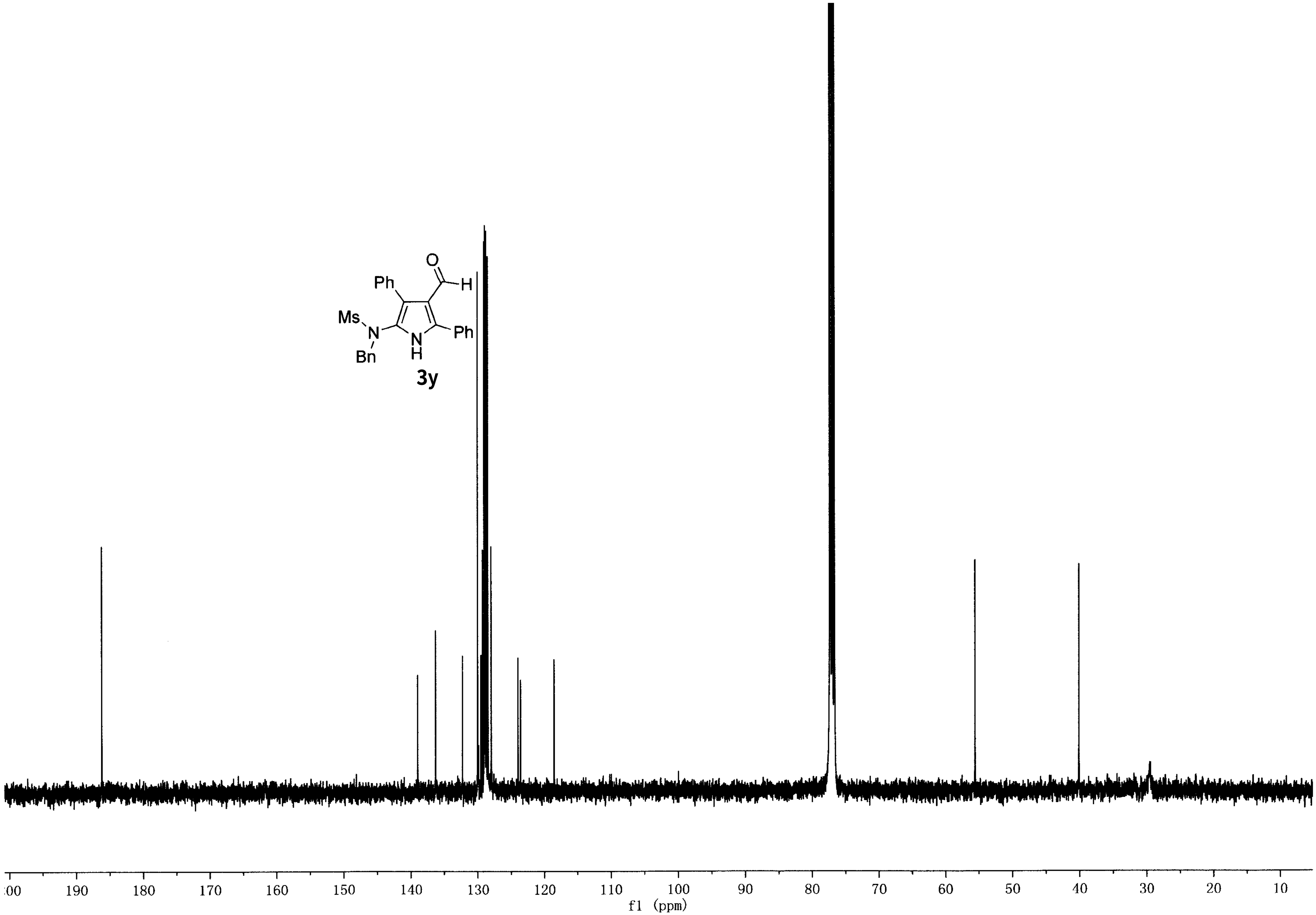

Ph
O
H
Ms
N
Bn
N
H
Ph
3y
00
190
180
170
160
150
140
130
120
110
100
90
80
70
60
50
40
30
20
10
f1 (ppm)

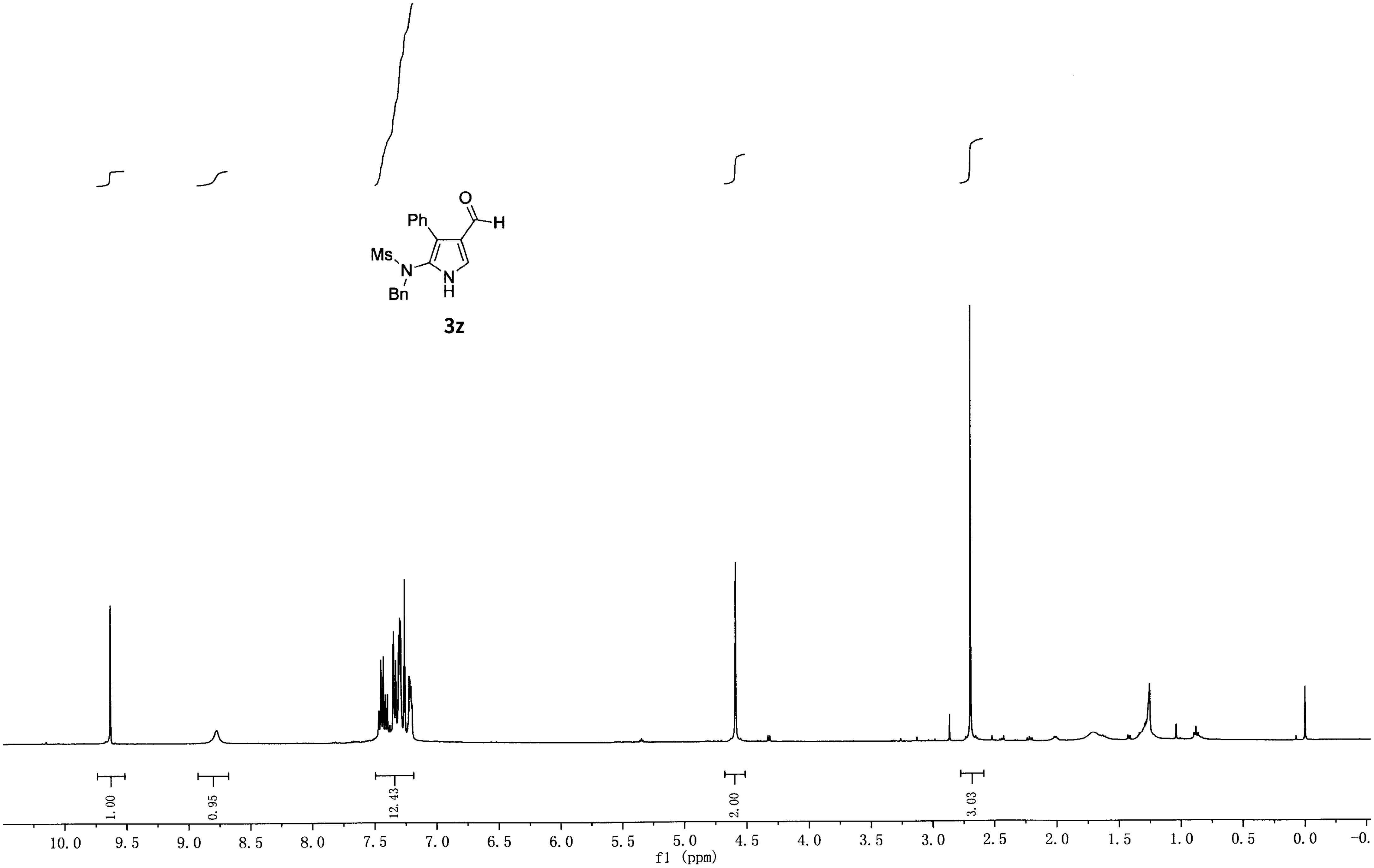

Ph
O
H
Ms
N
Bn
N
H
3z
1.00
0.95
12.43
2.00
3.03
10.0
9.5
9.0
8.5
8.0
7.5
7.0
6.5
6.0
5.5
5.0
4.5
4.0
3.5
3.0
2.5
2.0
1.5
1.0
0.5
0.0
-0.
f1 (ppm)

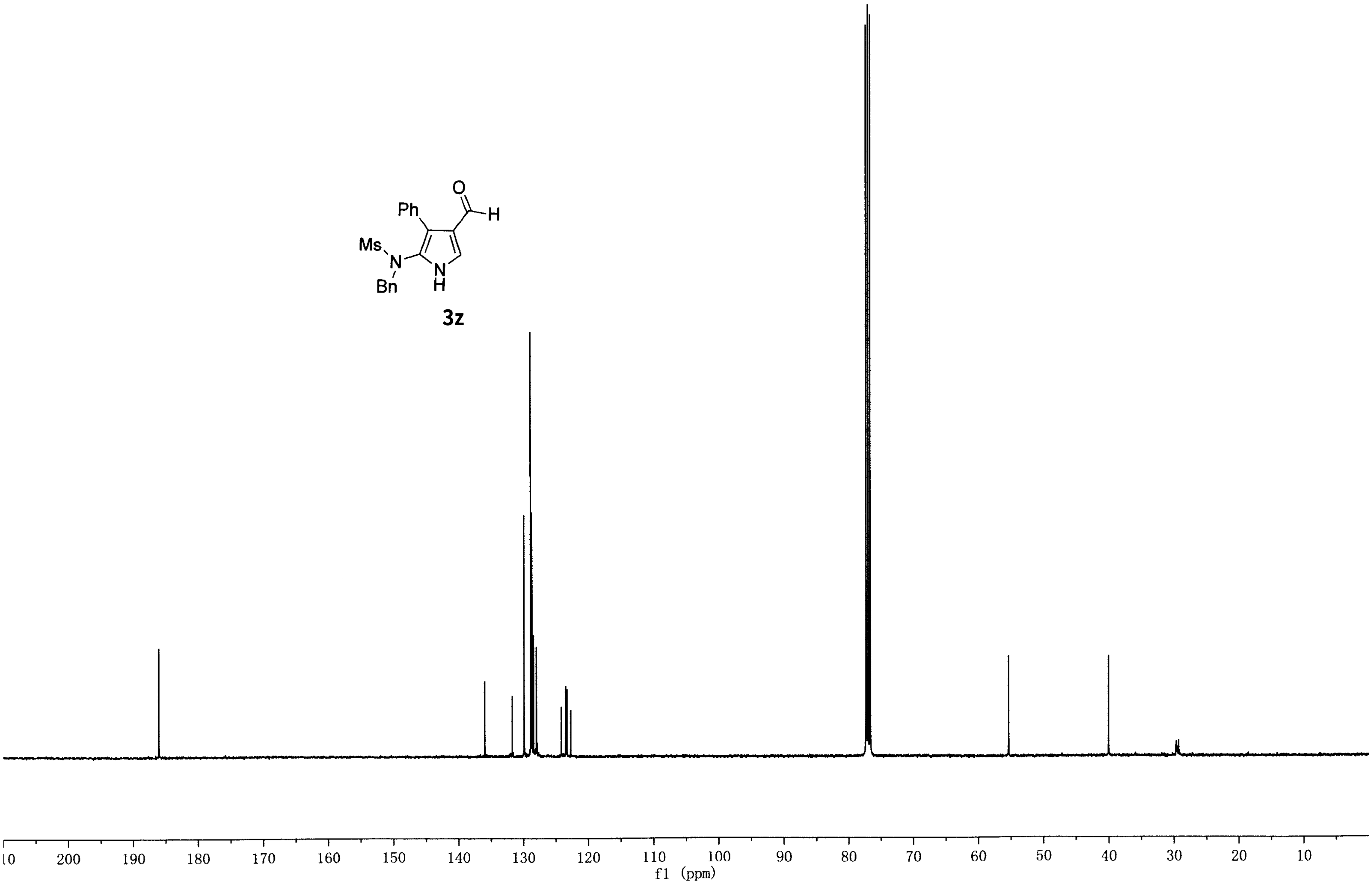
O
Ph
H
Ms
N
Bn
N
H
3z
10
200
190
180
170
160
150
140
130
120
110
100
90
80
70
60
50
40
30
20
10
f1 (ppm)

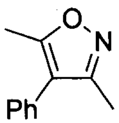

4a

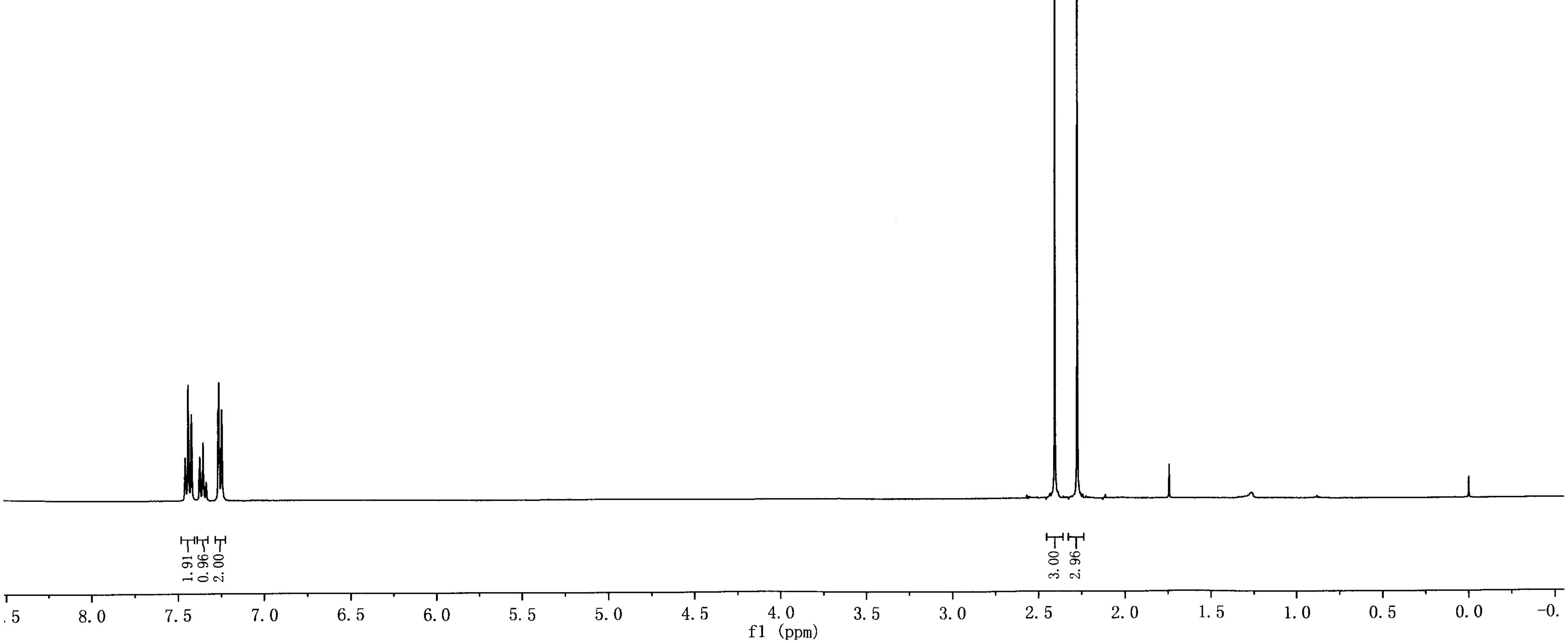

1.91
0.96
2.00
3.00
2.96
.5 8.0 7.5 7.0 6.5 6.0 5.5 5.0 4.5 4.0 3.5 3.0 2.5 2.0 1.5 1.0 0.5 0.0 -0.
f1 (ppm)

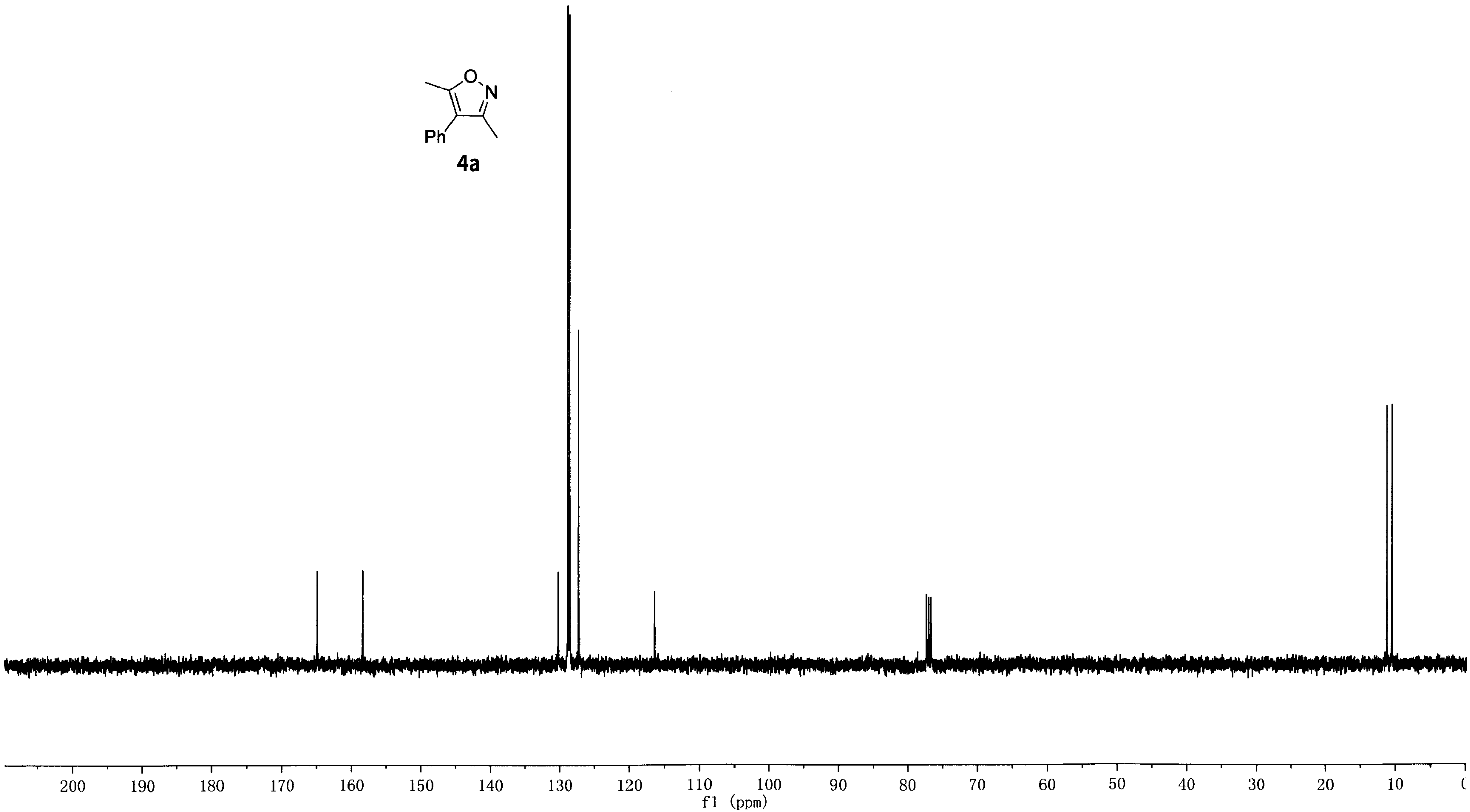
O
N
Ph
4a
200 190 180 170 160 150 140 130 120 110 100 90 80 70 60 50 40 30 20 10
f1 (ppm)

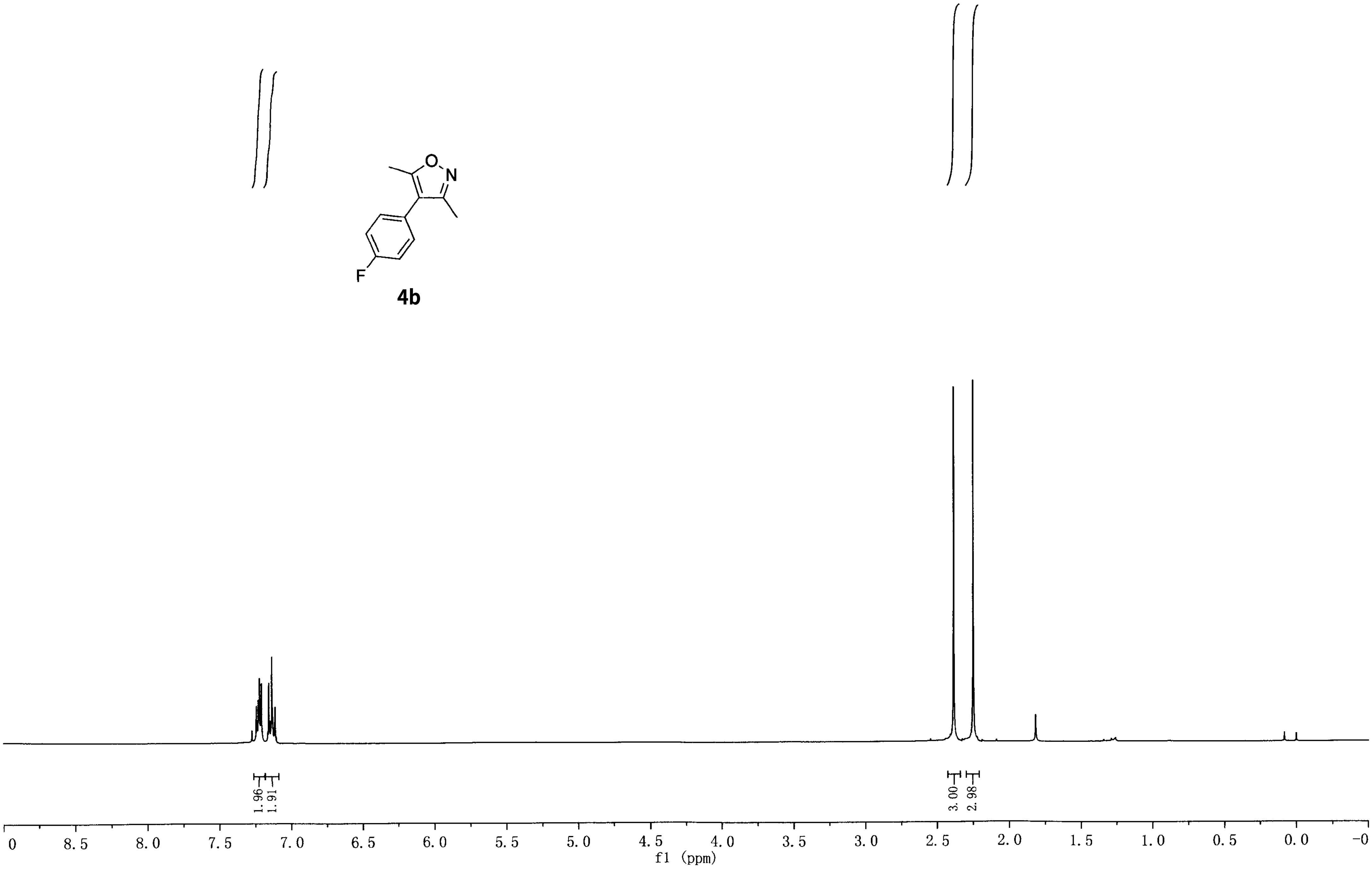

O
N
F
4b
1.96
1.91
3.00
2.98
0
8.5
8.0
7.5
7.0
6.5
6.0
5.5
5.0
4.5
4.0
3.5
3.0
2.5
2.0
1.5
1.0
0.5
0.0
-0
f1 (ppm)

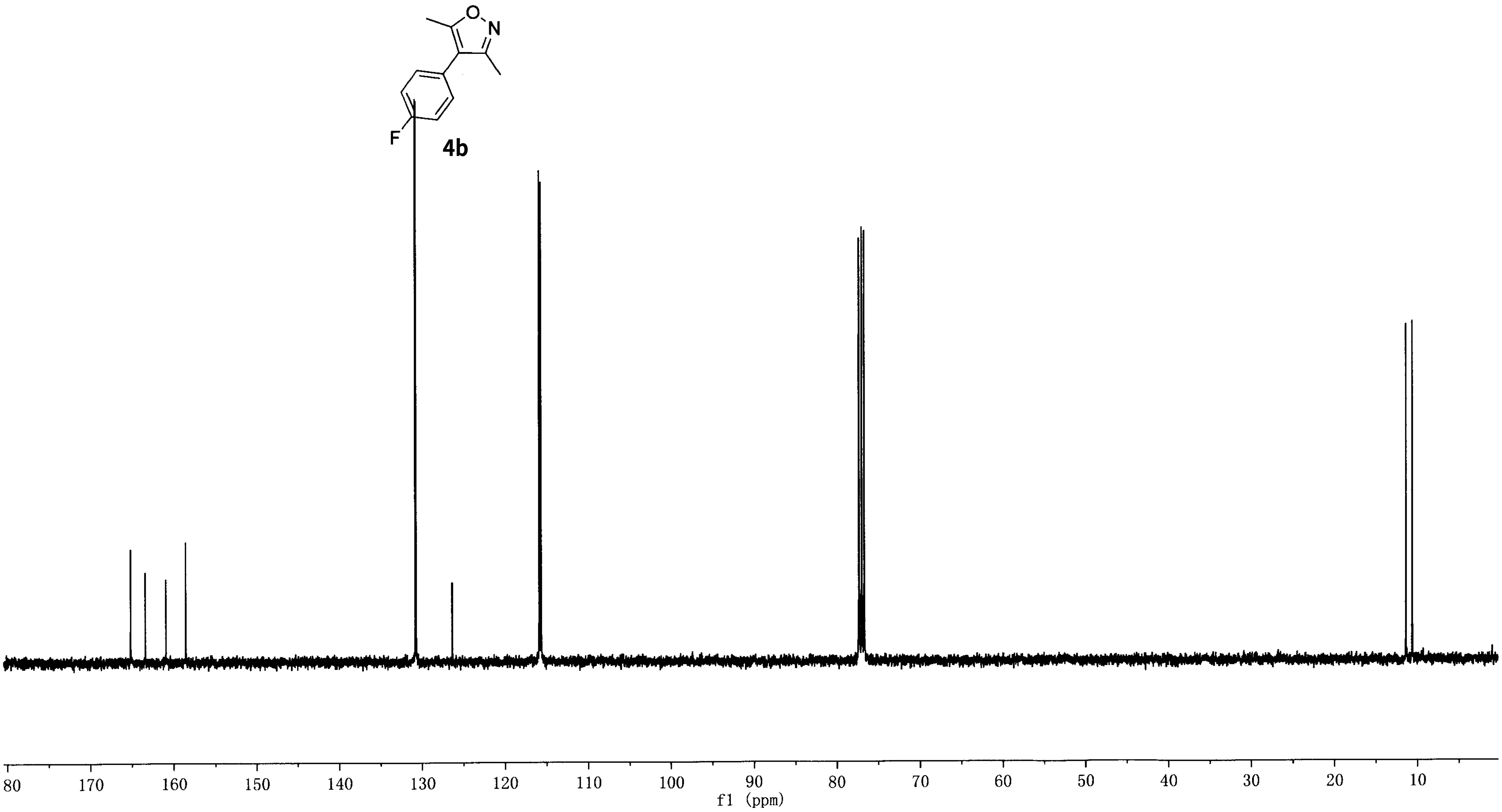
O
N
F
4b
80
170
160
150
140
130
120
110
100
90
80
70
60
50
40
30
20
10
f1 (ppm)

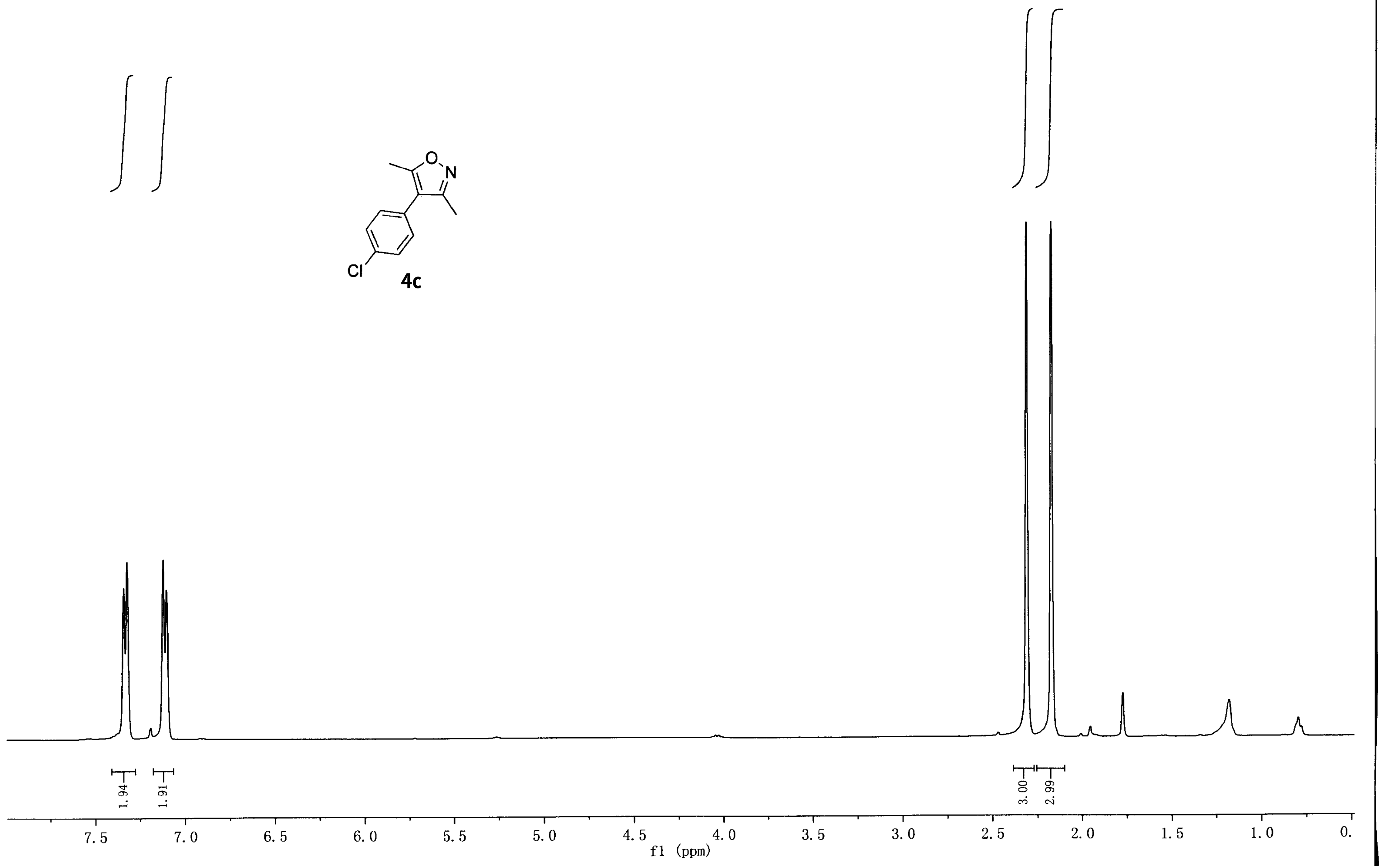

O
N
Cl
4c
1.94
1.91
3.00
2.99
7.5
7.0
6.5
6.0
5.5
5.0
4.5
4.0
3.5
3.0
2.5
2.0
1.5
1.0
0.
f1 (ppm)

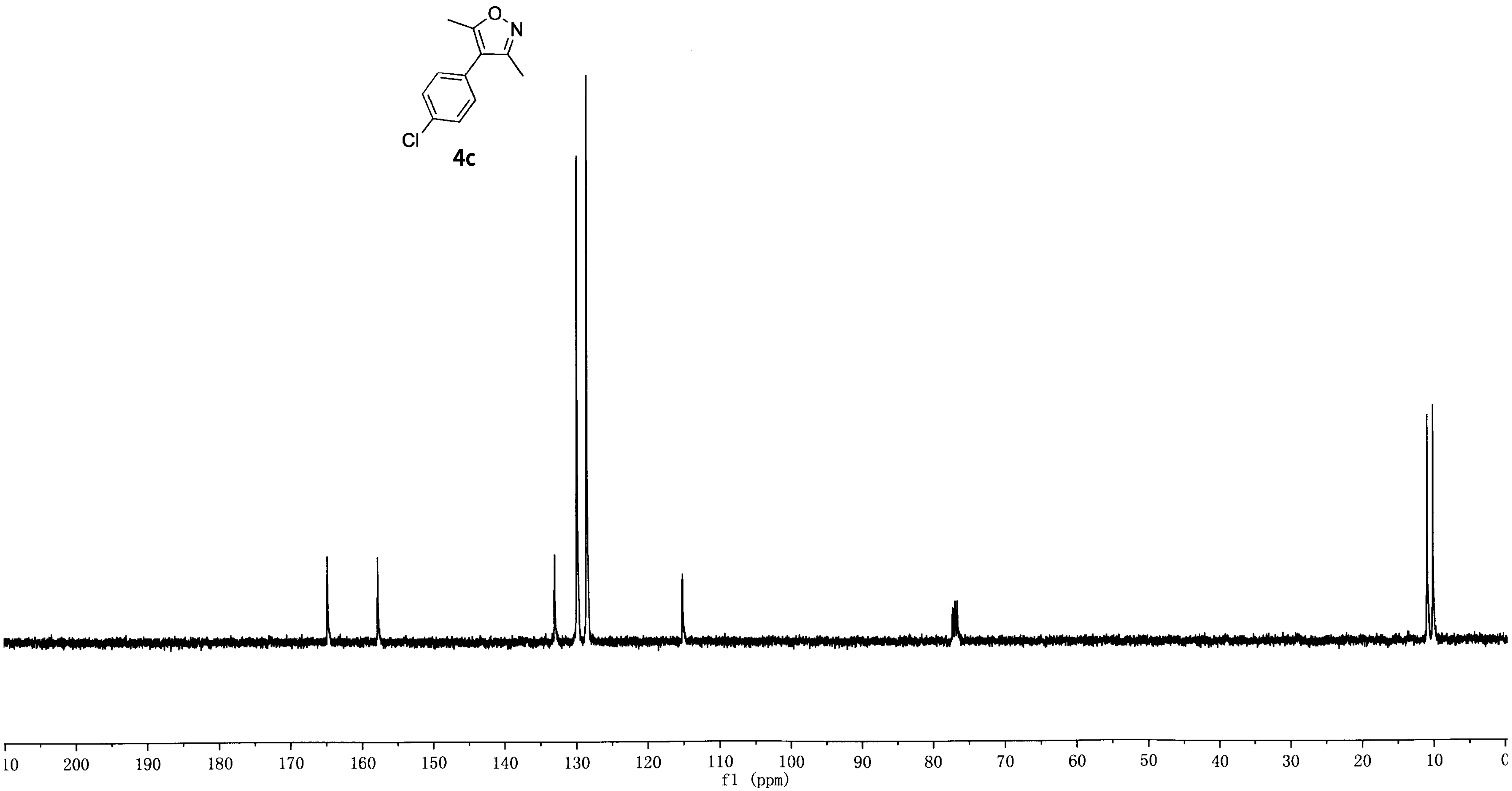

O
N
Cl
4c
10
200
190
180
170
160
150
140
130
120
110
100
90
80
70
60
50
40
30
20
10
0
f1 (ppm)

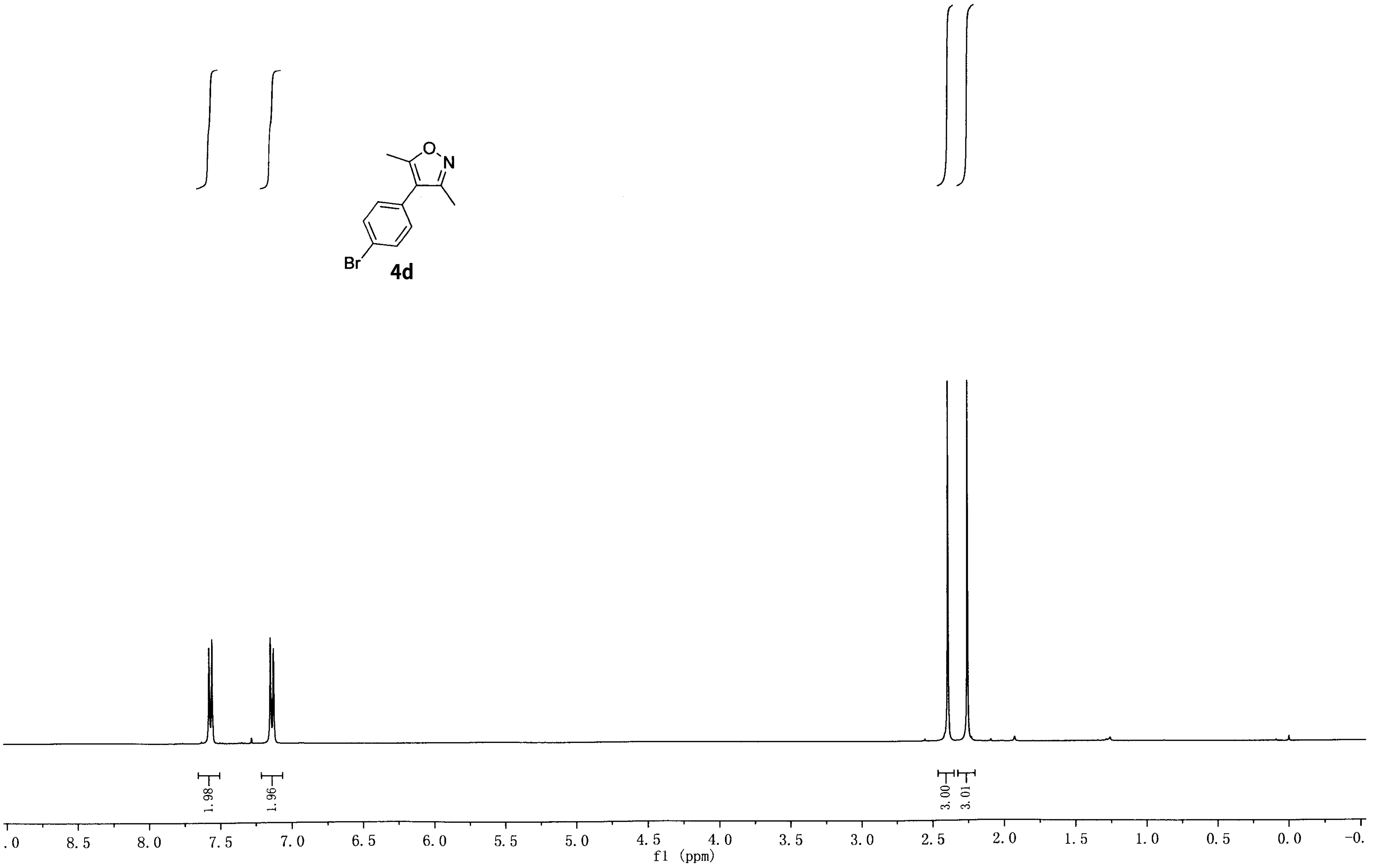

O
N
Br
4d
1.98
1.96
3.00
3.01
.0
8.5
8.0
7.5
7.0
6.5
6.0
5.5
5.0
4.5
4.0
3.5
3.0
2.5
2.0
1.5
1.0
0.5
0.0
-0.
f1 (ppm)

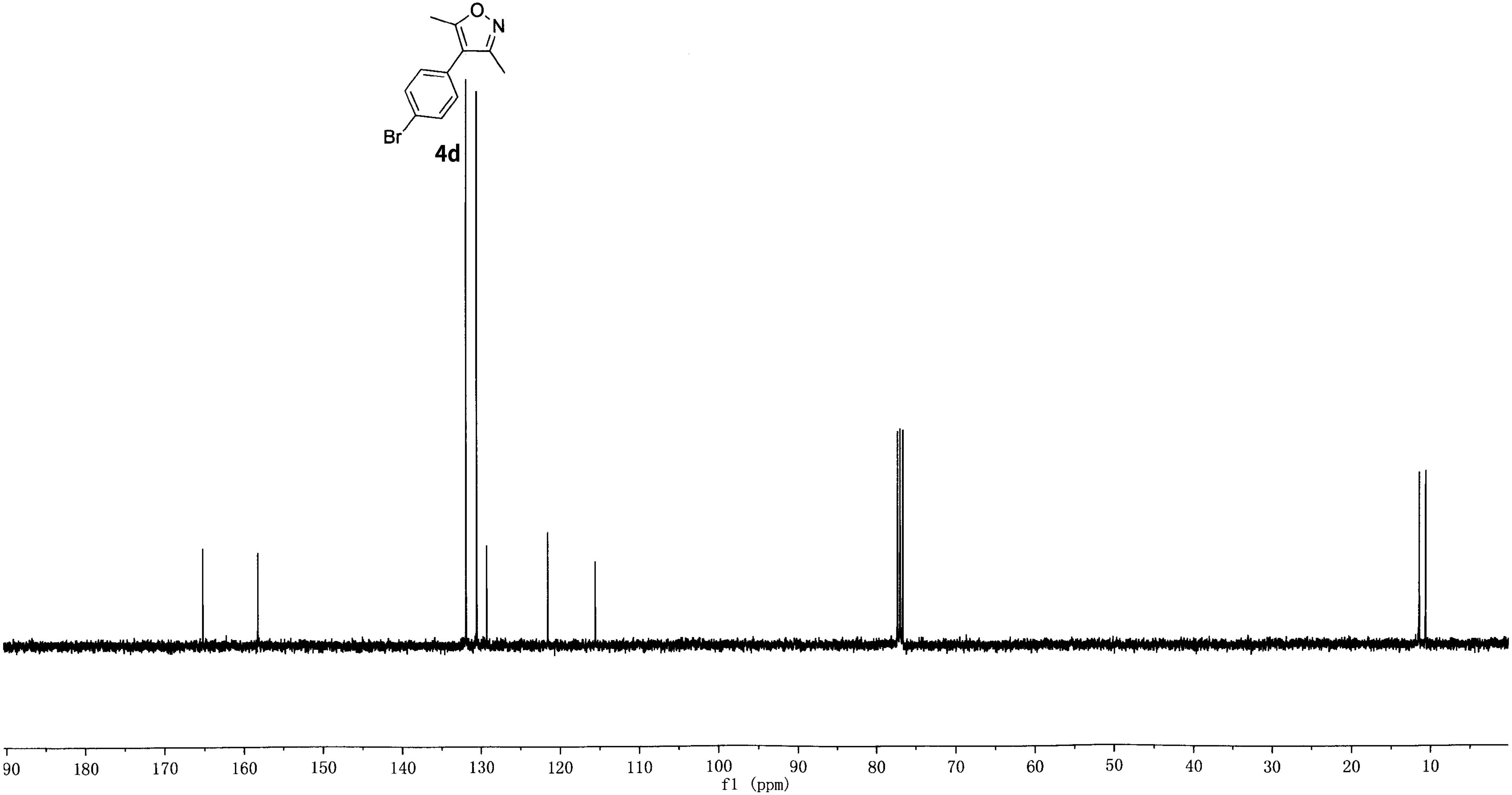
O
N
Br
4d
90
180
170
160
150
140
130
120
110
100
90
80
70
60
50
40
30
20
10
f1 (ppm)

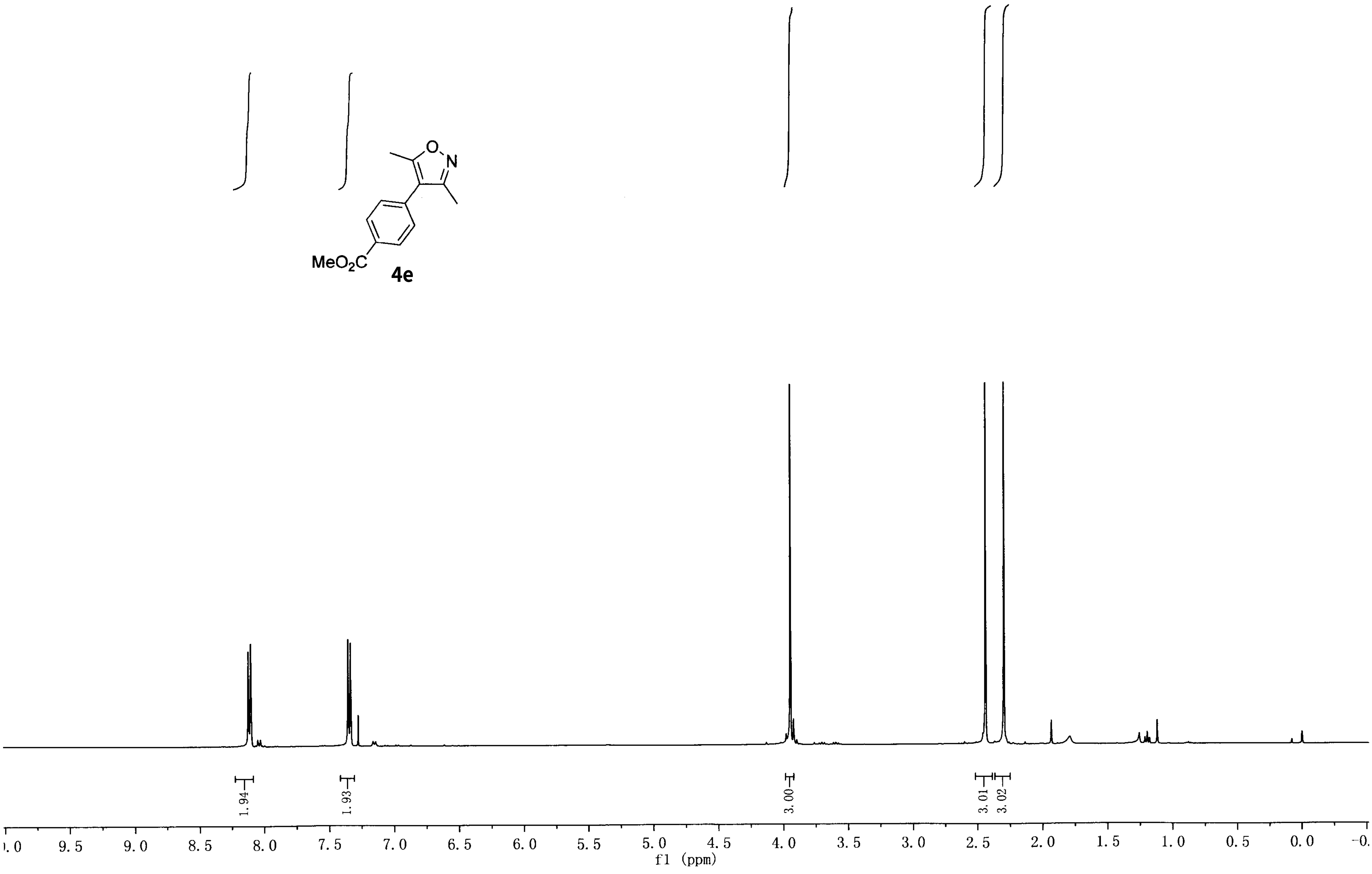

O
N
MeO2C
4e
1.94
1.93
3.00
3.01
3.02
9.5
9.0
8.5
8.0
7.5
7.0
6.5
6.0
5.5
5.0
4.5
4.0
3.5
3.0
2.5
2.0
1.5
1.0
0.5
0.0
f1 (ppm)

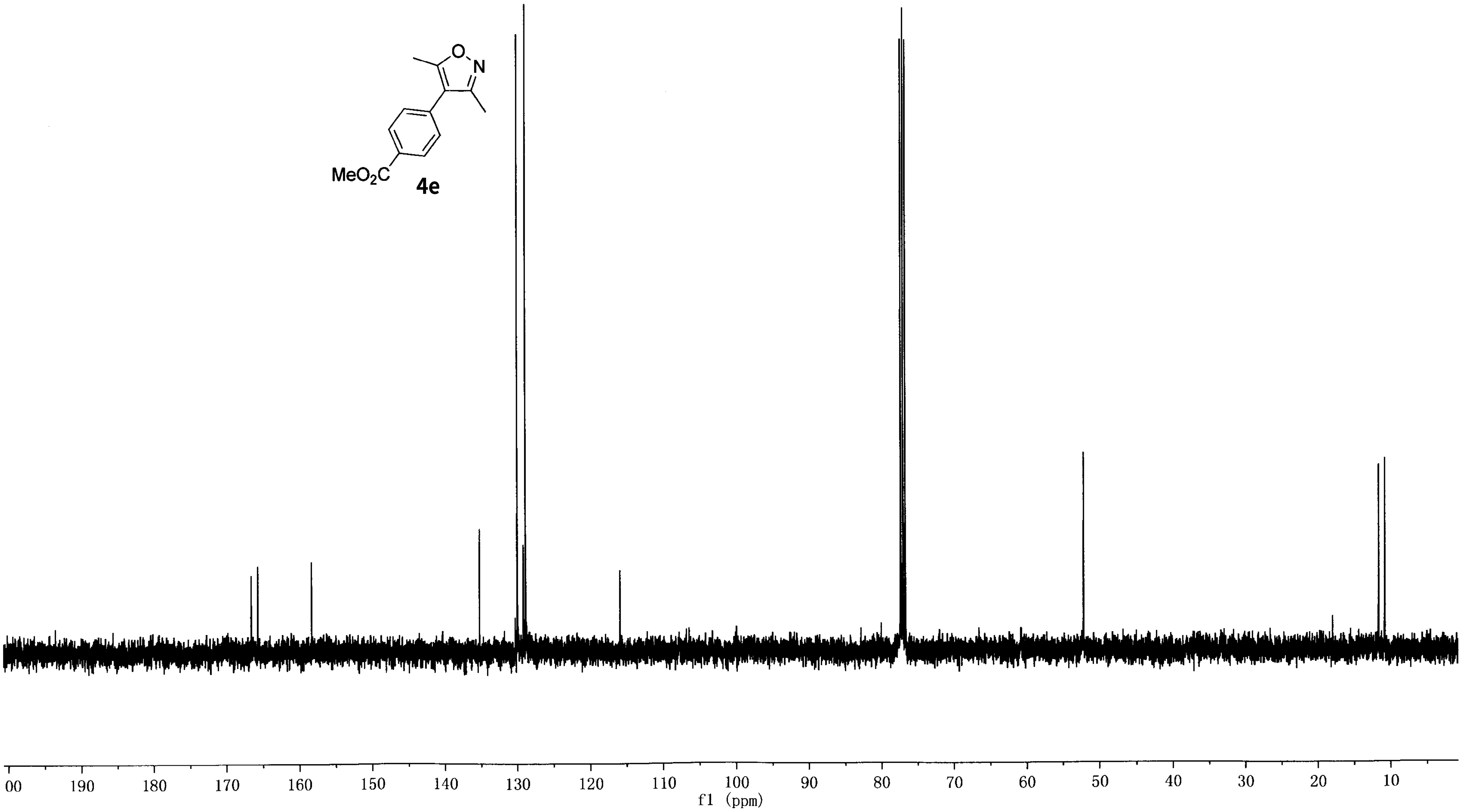

O
N
MeO2C
4e
00
190
180
170
160
150
140
130
120
110
100
90
80
70
60
50
40
30
20
10
f1 (ppm)

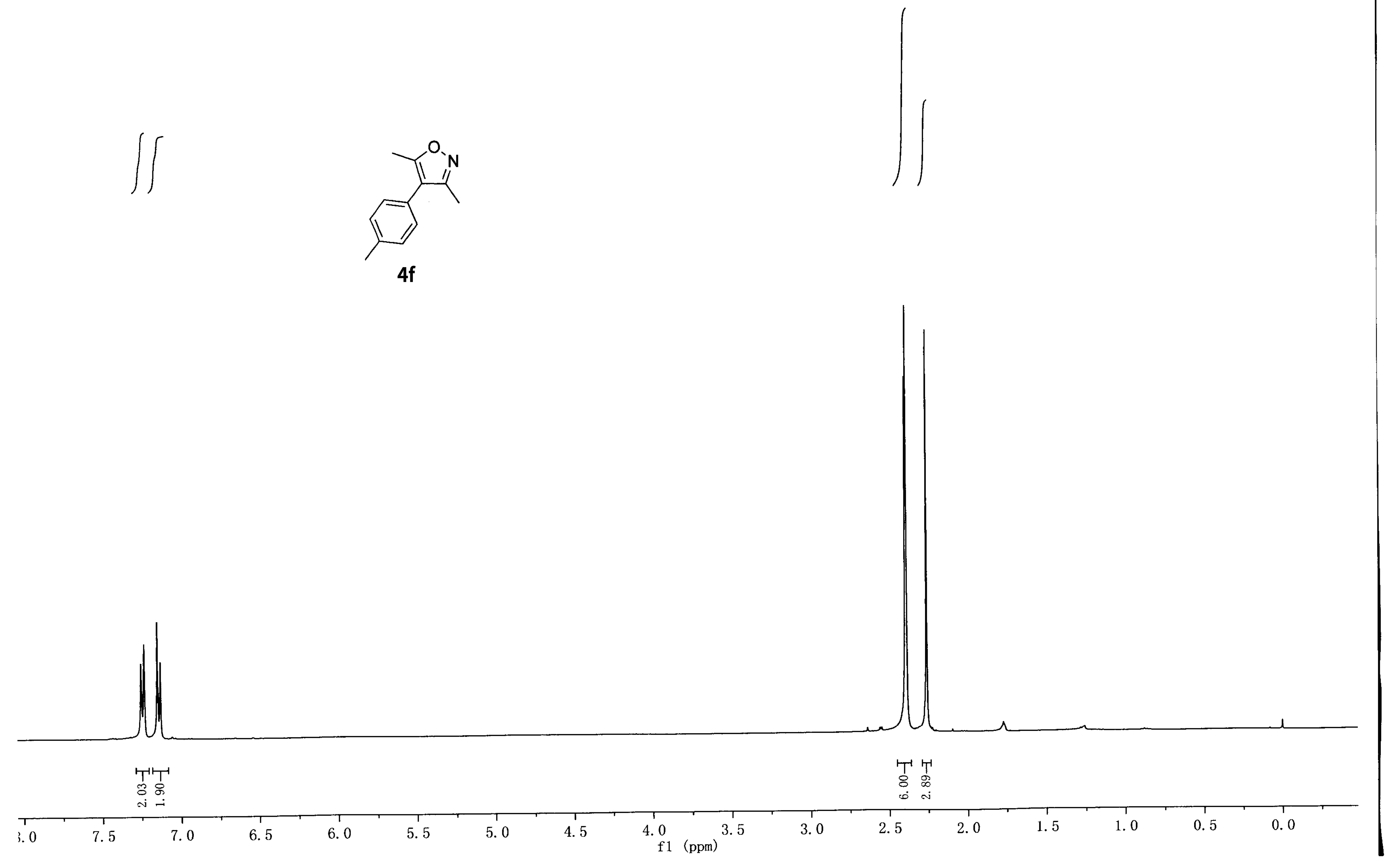

4f
2.03
1.90
6.00
2.89
8.0
7.5
7.0
6.5
6.0
5.5
5.0
4.5
4.0
3.5
3.0
2.5
2.0
1.5
1.0
0.5
0.0
f1 (ppm)

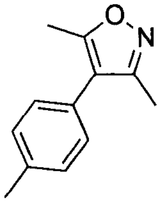
O
N

4f

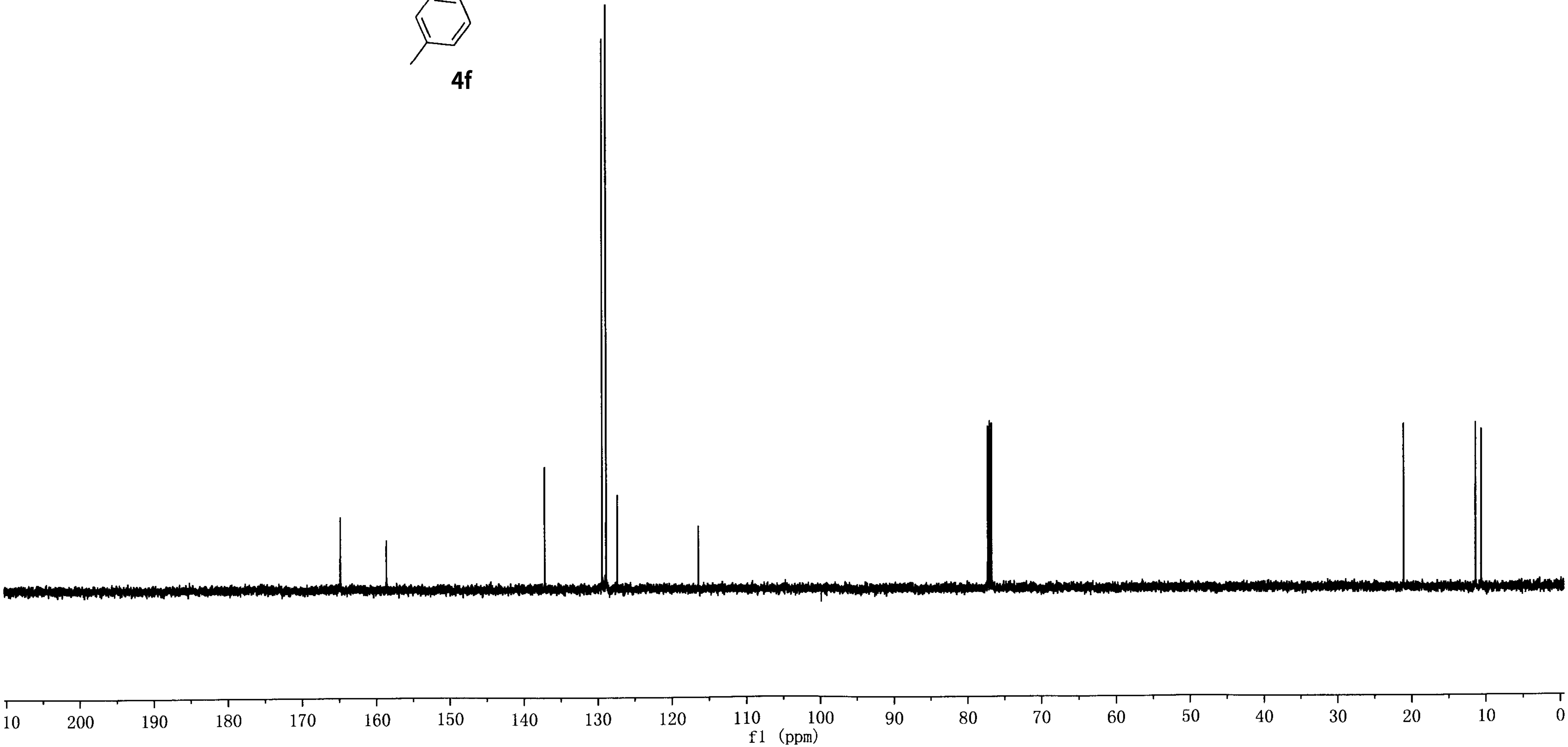
10 200 190 180 170 160 150 140 130 120 110 100 90 80 70 60 50 40 30 20 10 0
f1 (ppm)

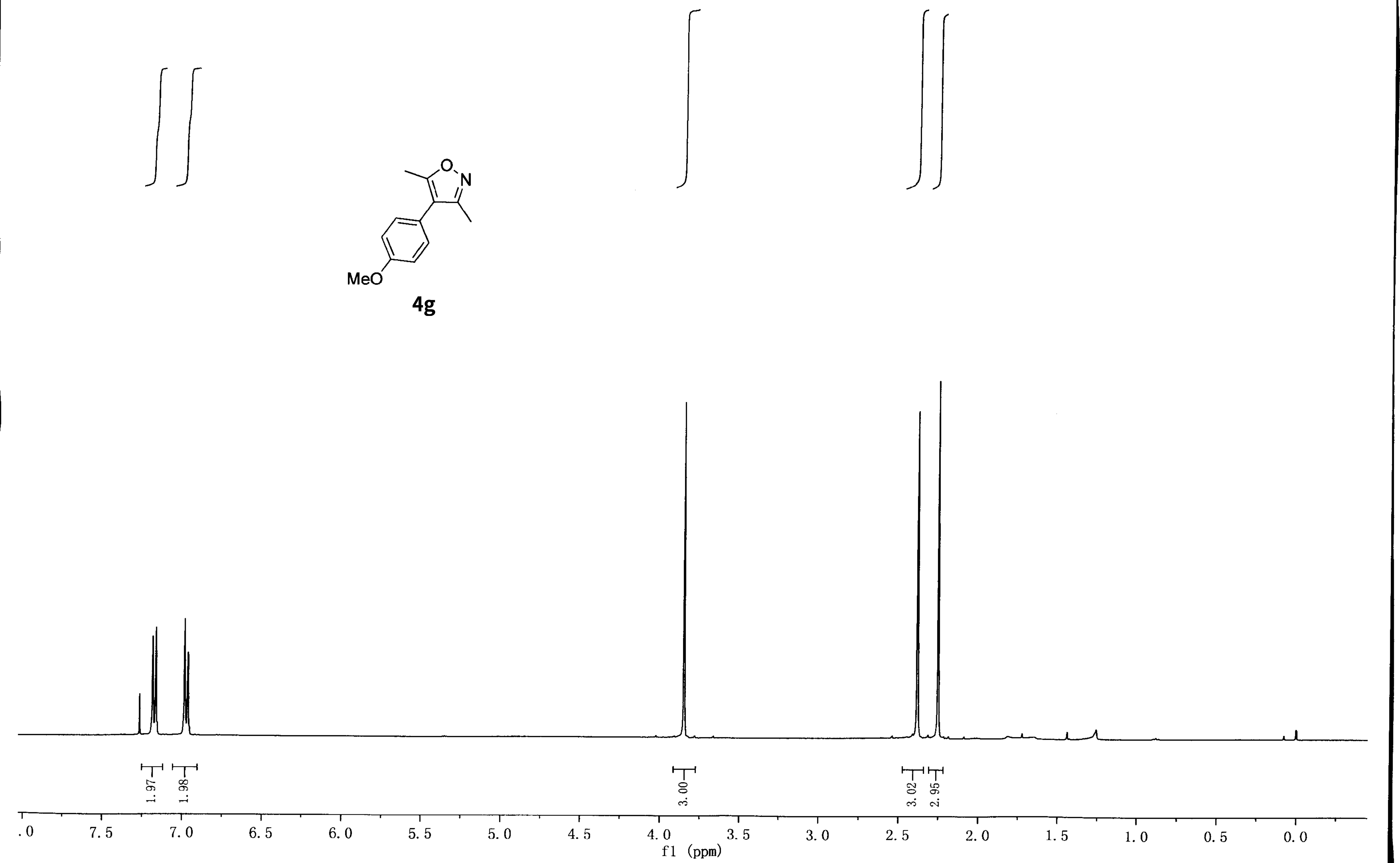
O
N
MeO
4g
1.97
1.98
3.00
3.02
2.95
.0 7.5 7.0 6.5 6.0 5.5 5.0 4.5 4.0 3.5 3.0 2.5 2.0 1.5 1.0 0.5 0.0
f1 (ppm)

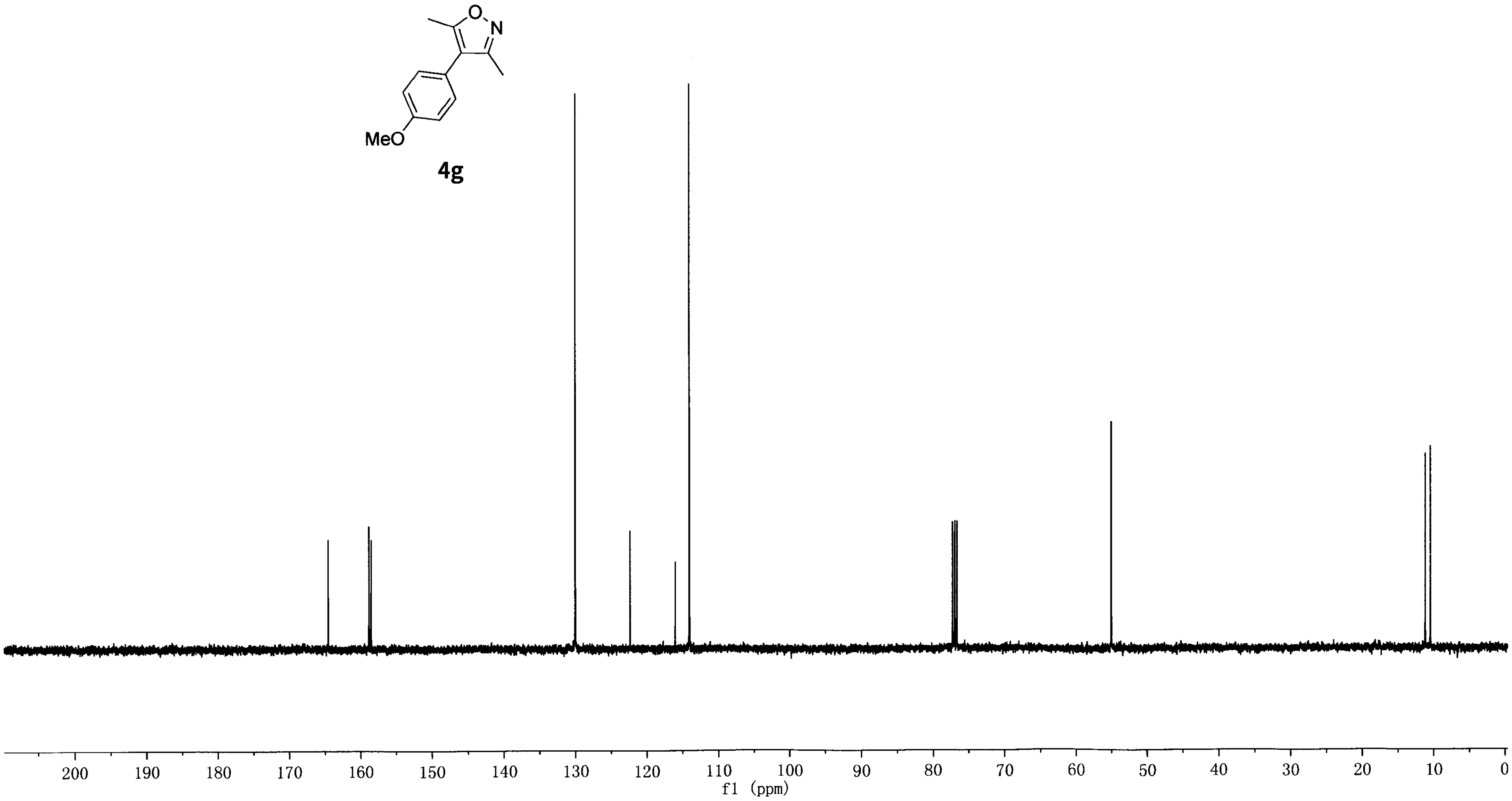
O
N
MeO
4g
200
190
180
170
160
150
140
130
120
110
100
90
80
70
60
50
40
30
20
10
0
f1 (ppm)

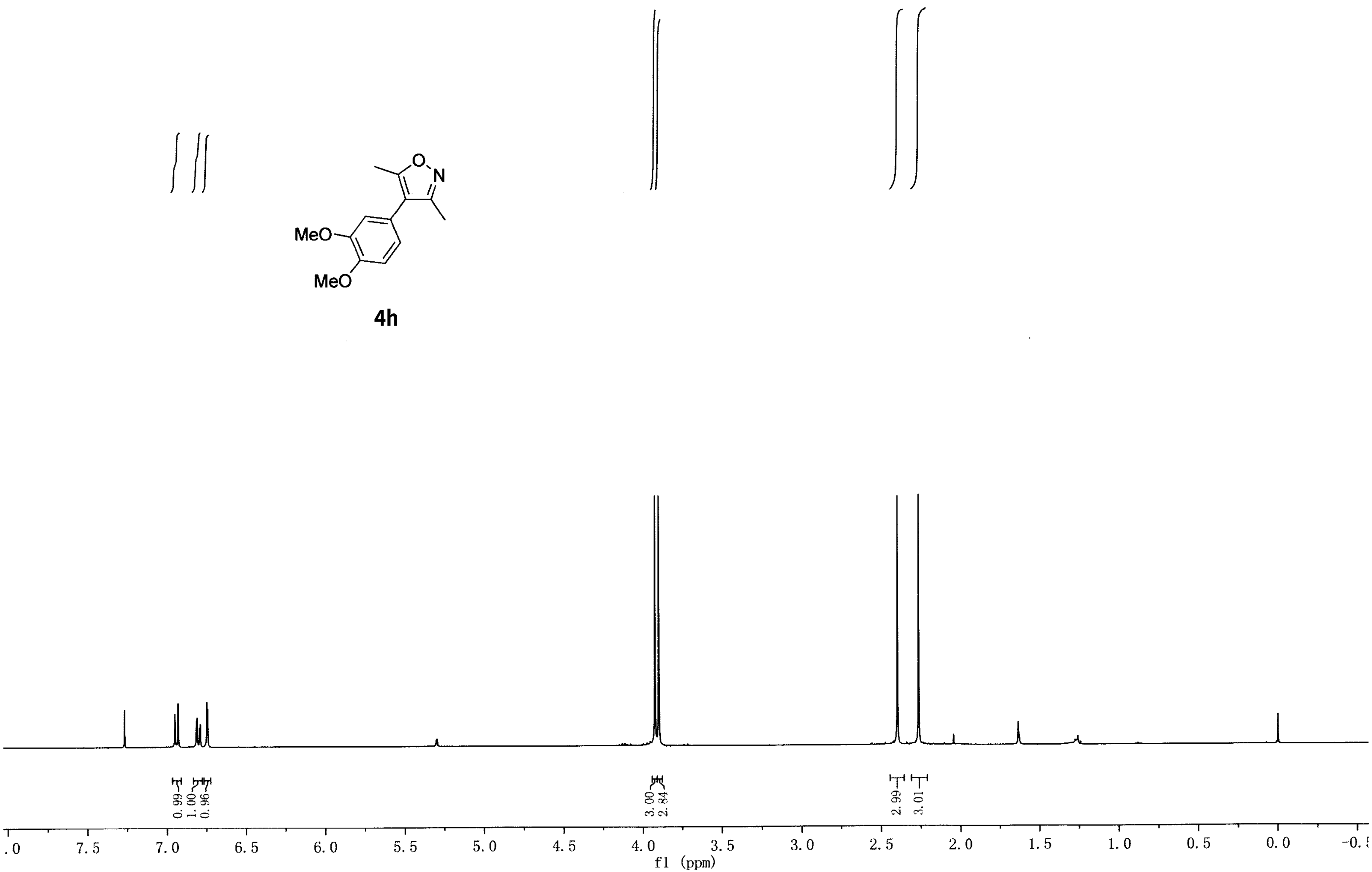

MeO
MeO
O
N
0.99
1.00
0.96
3.00
2.84
2.99
3.01
.0
7.5
7.0
6.5
6.0
5.5
5.0
4.5
4.0
3.5
3.0
2.5
2.0
1.5
1.0
0.5
0.0
-0.5
f1 (ppm)

4h

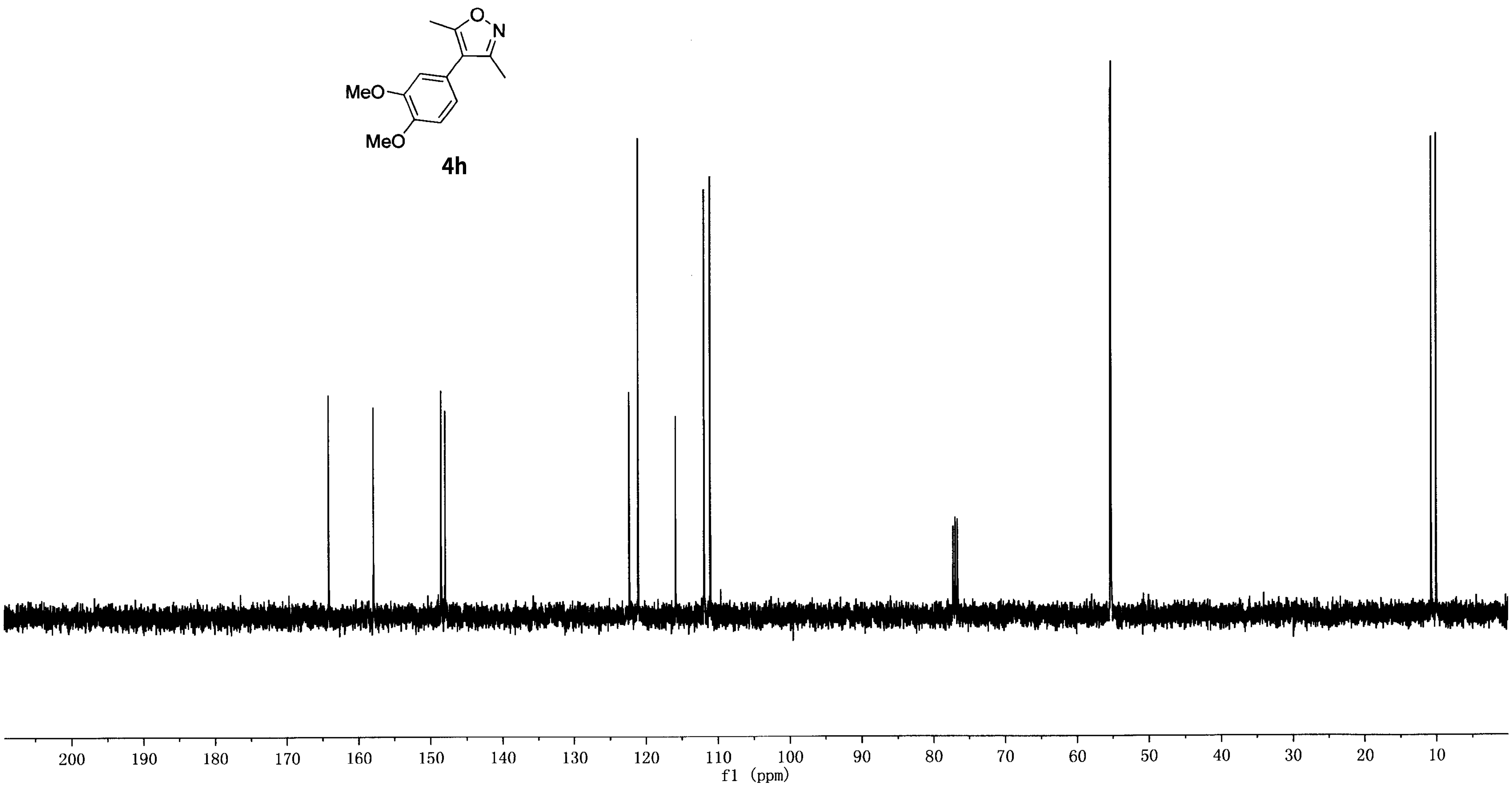
O
N
MeO
MeO
4h
200
190
180
170
160
150
140
130
120
110
100
90
80
70
60
50
40
30
20
10
f1 (ppm)

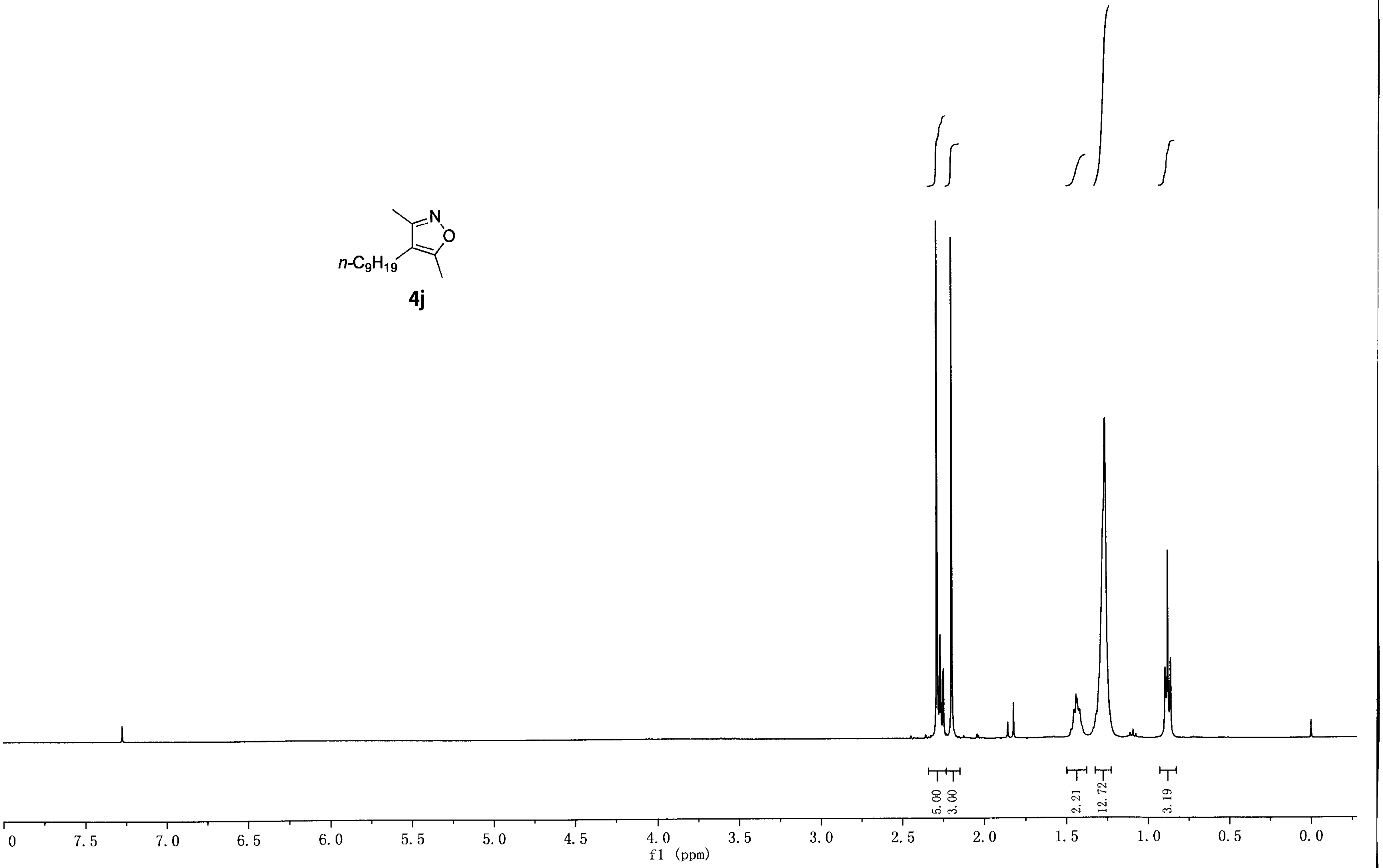

n-C9H19
N
O
4j
5.00
3.00
2.21
12.72
3.19
0
7.5
7.0
6.5
6.0
5.5
5.0
4.5
4.0
3.5
3.0
2.5
2.0
1.5
1.0
0.5
0.0
f1 (ppm)

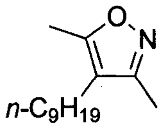

4j

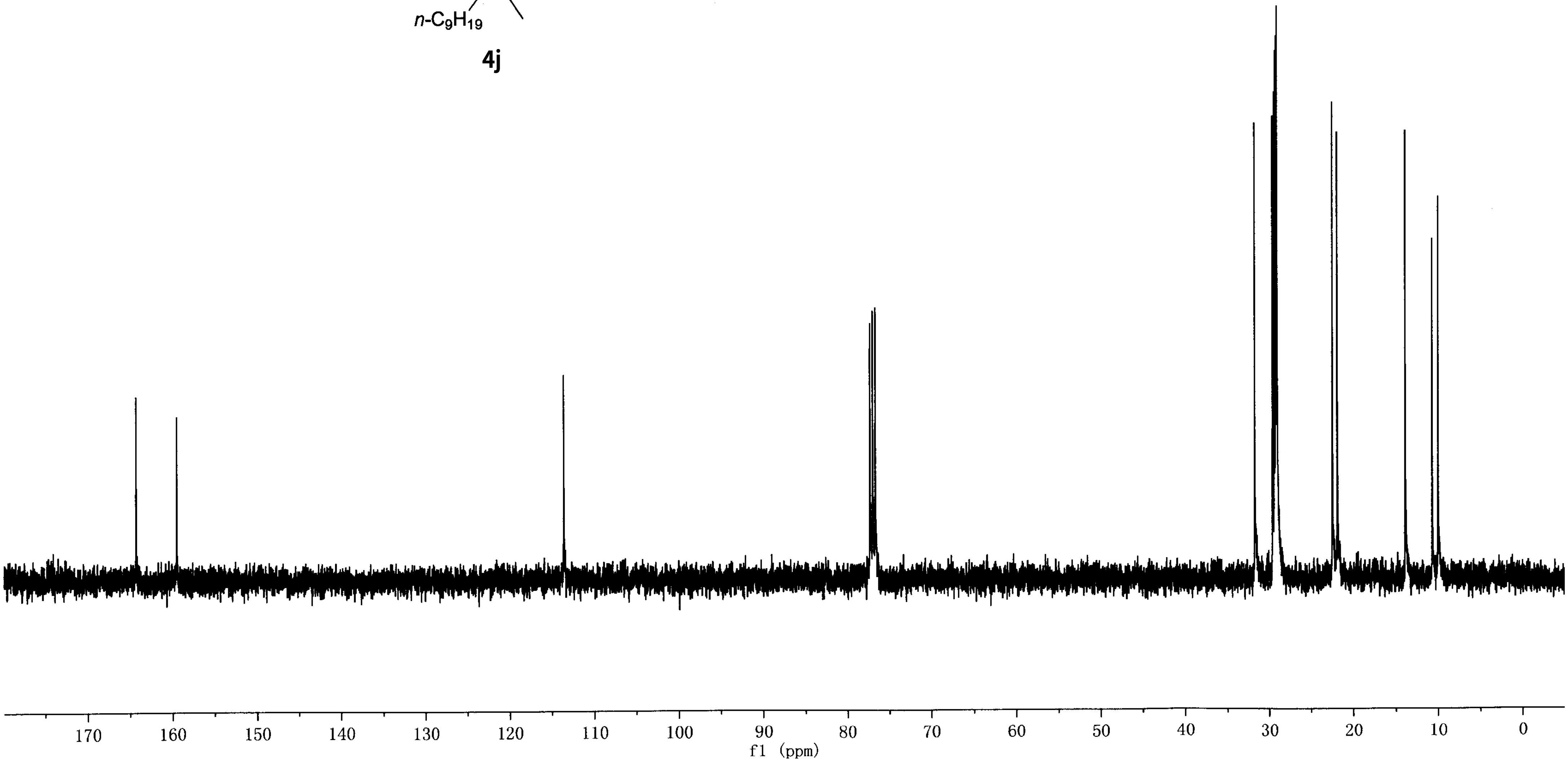

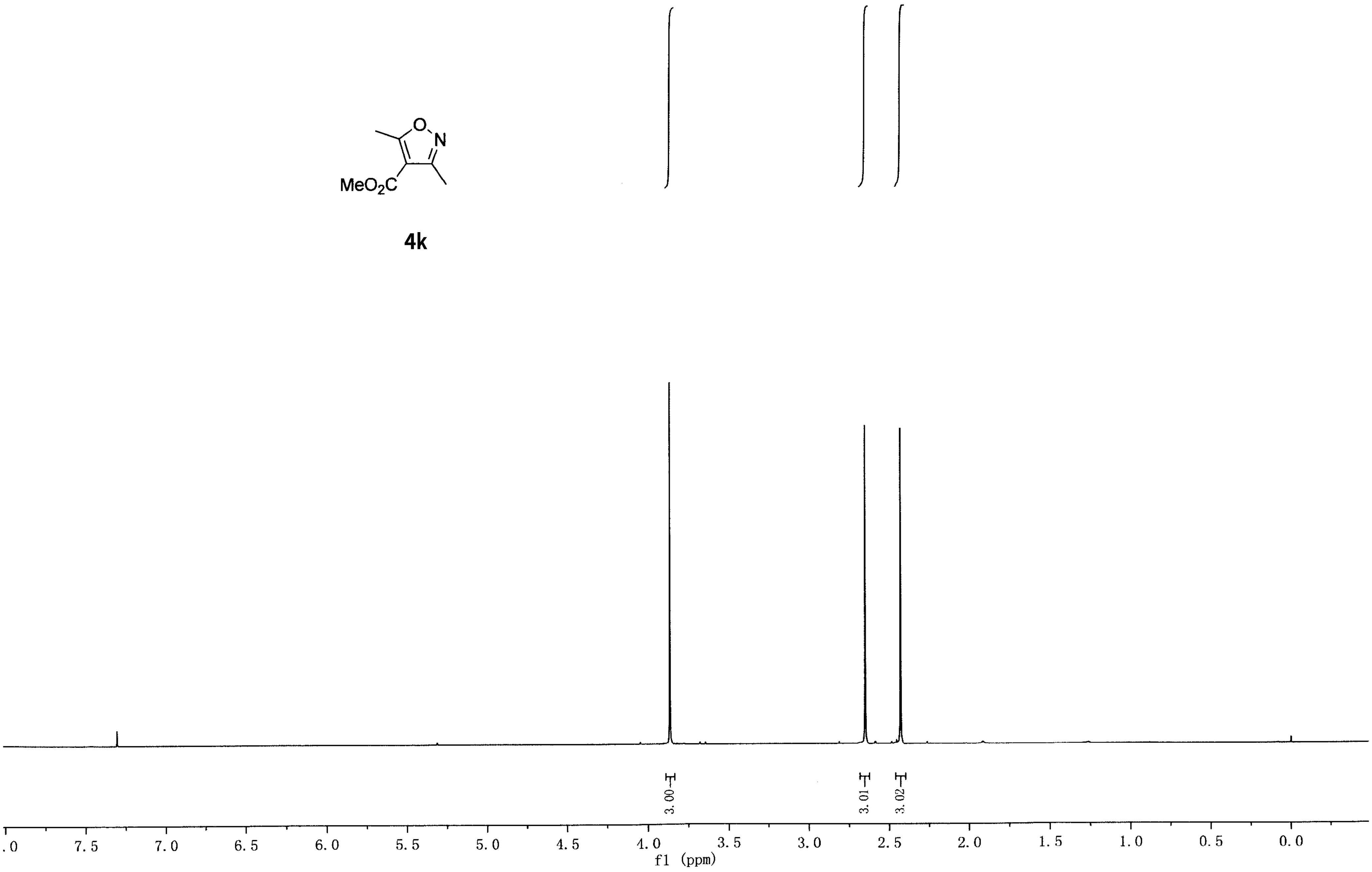

MeO2C
O
N
4k
3.00
3.01
3.02
.0
7.5
7.0
6.5
6.0
5.5
5.0
4.5
4.0
3.5
3.0
2.5
2.0
1.5
1.0
0.5
0.0
f1 (ppm)

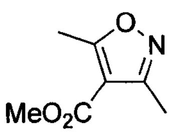

MeO2C
O
N

4k

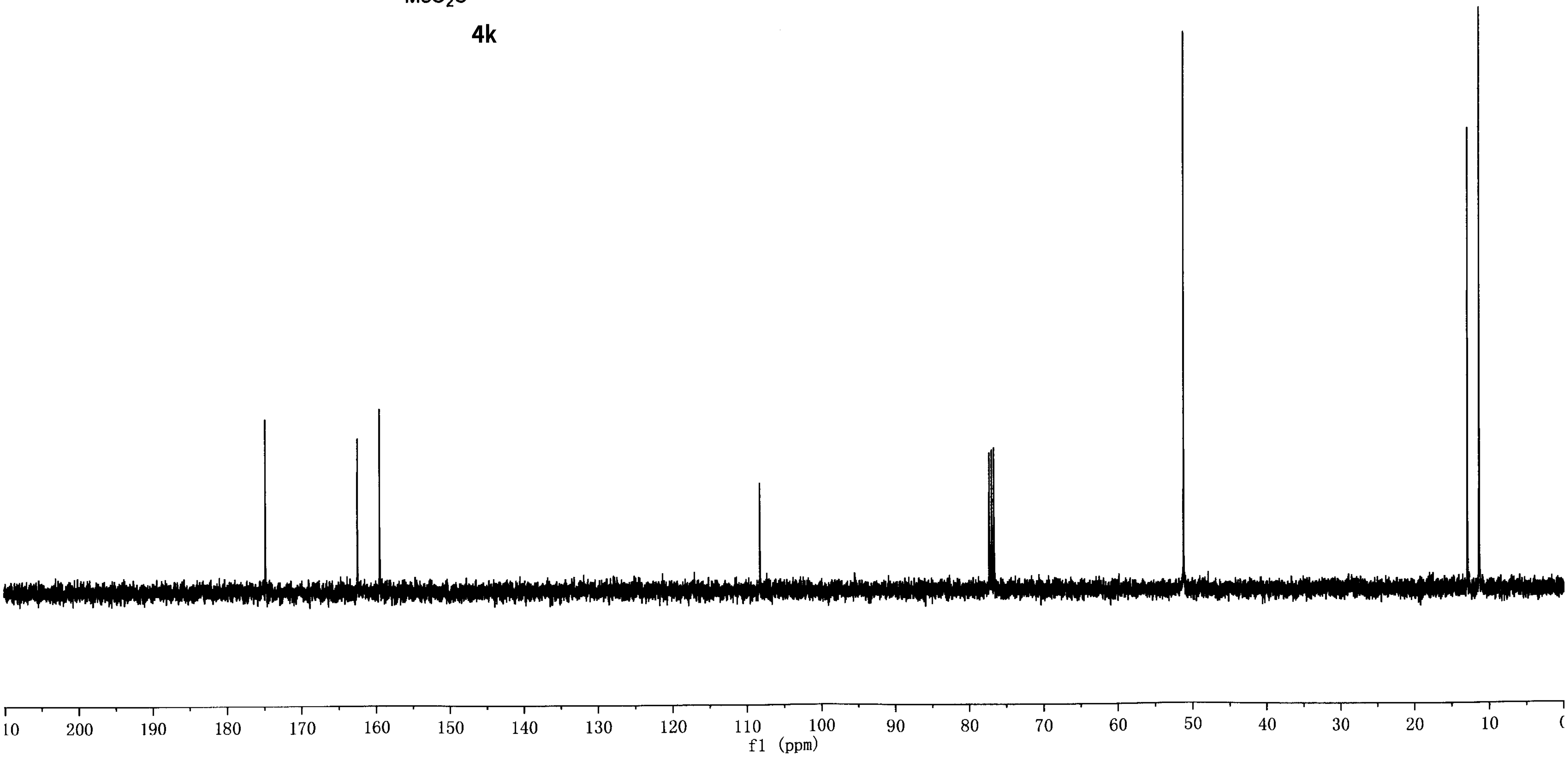

10
200
190
180
170
160
150
140
130
120
110
100
90
80
70
60
50
40
30
20
10
f1 (ppm)

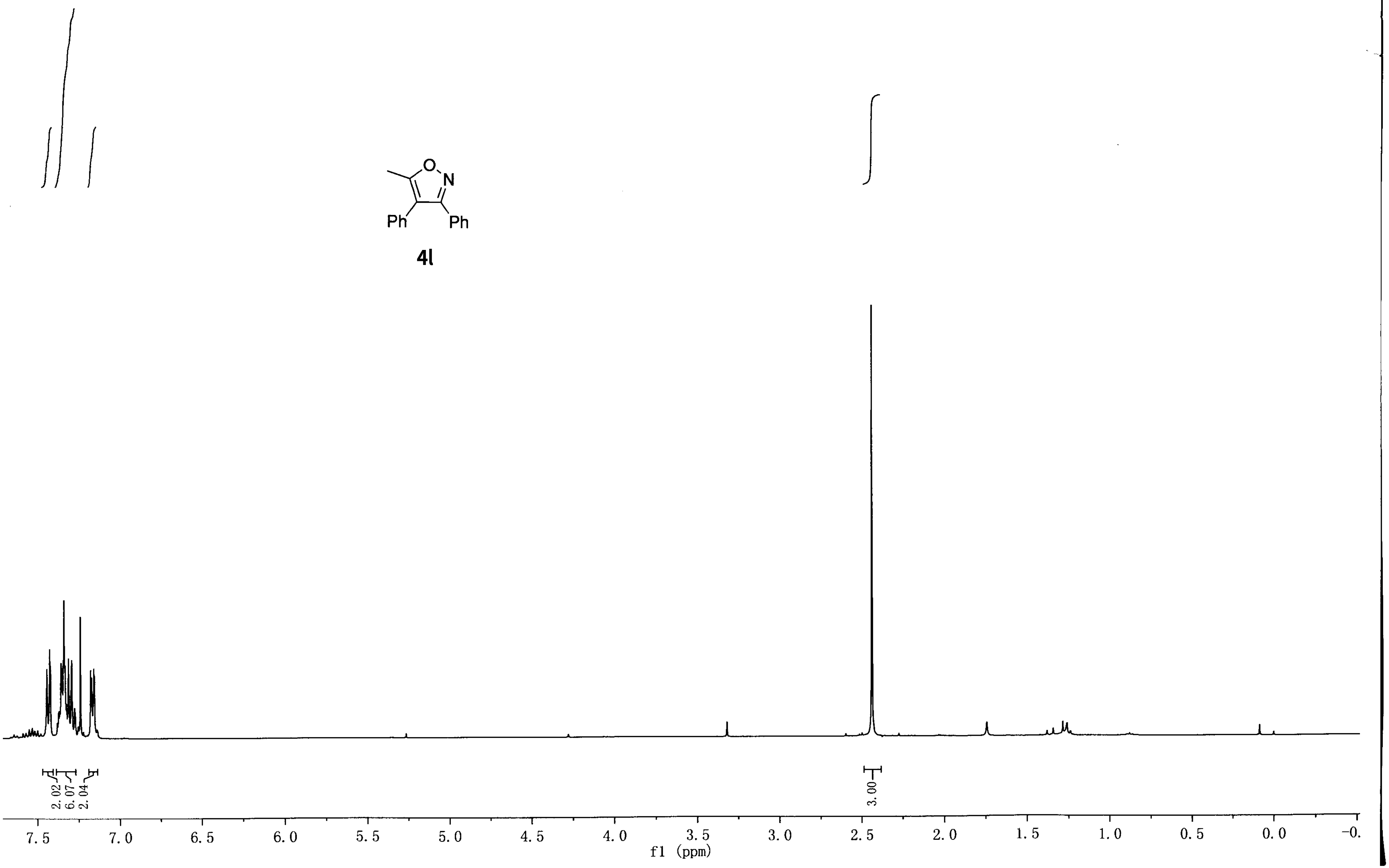

O
N
Ph
Ph
4l
2.02
6.07
2.04
3.00
7.5
7.0
6.5
6.0
5.5
5.0
4.5
4.0
3.5
3.0
2.5
2.0
1.5
1.0
0.5
0.0
-0.
f1 (ppm)

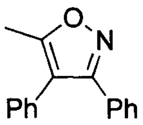
O
N
Ph
Ph

4l

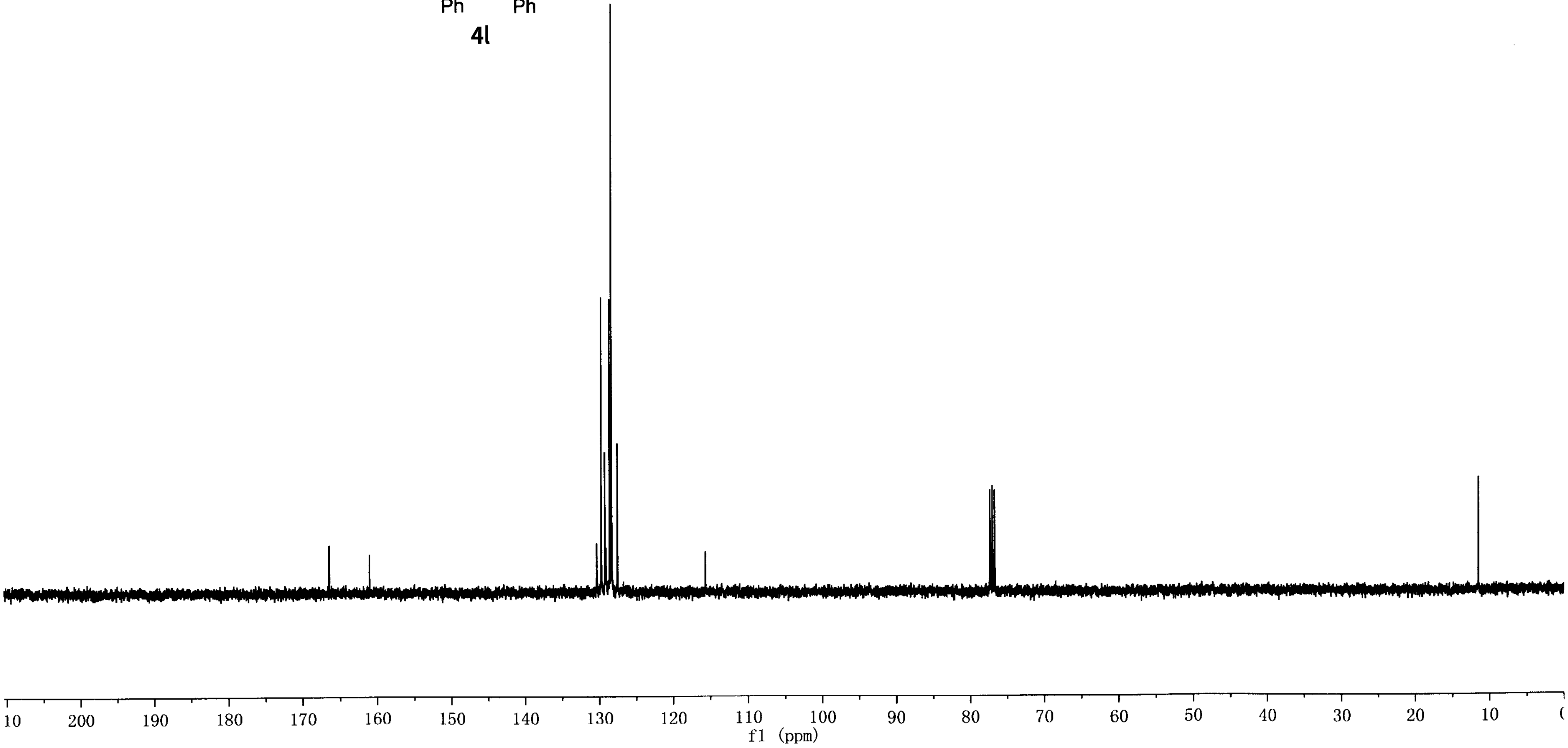
10 200 190 180 170 160 150 140 130 120 110 100 90 80 70 60 50 40 30 20 10
f1 (ppm)

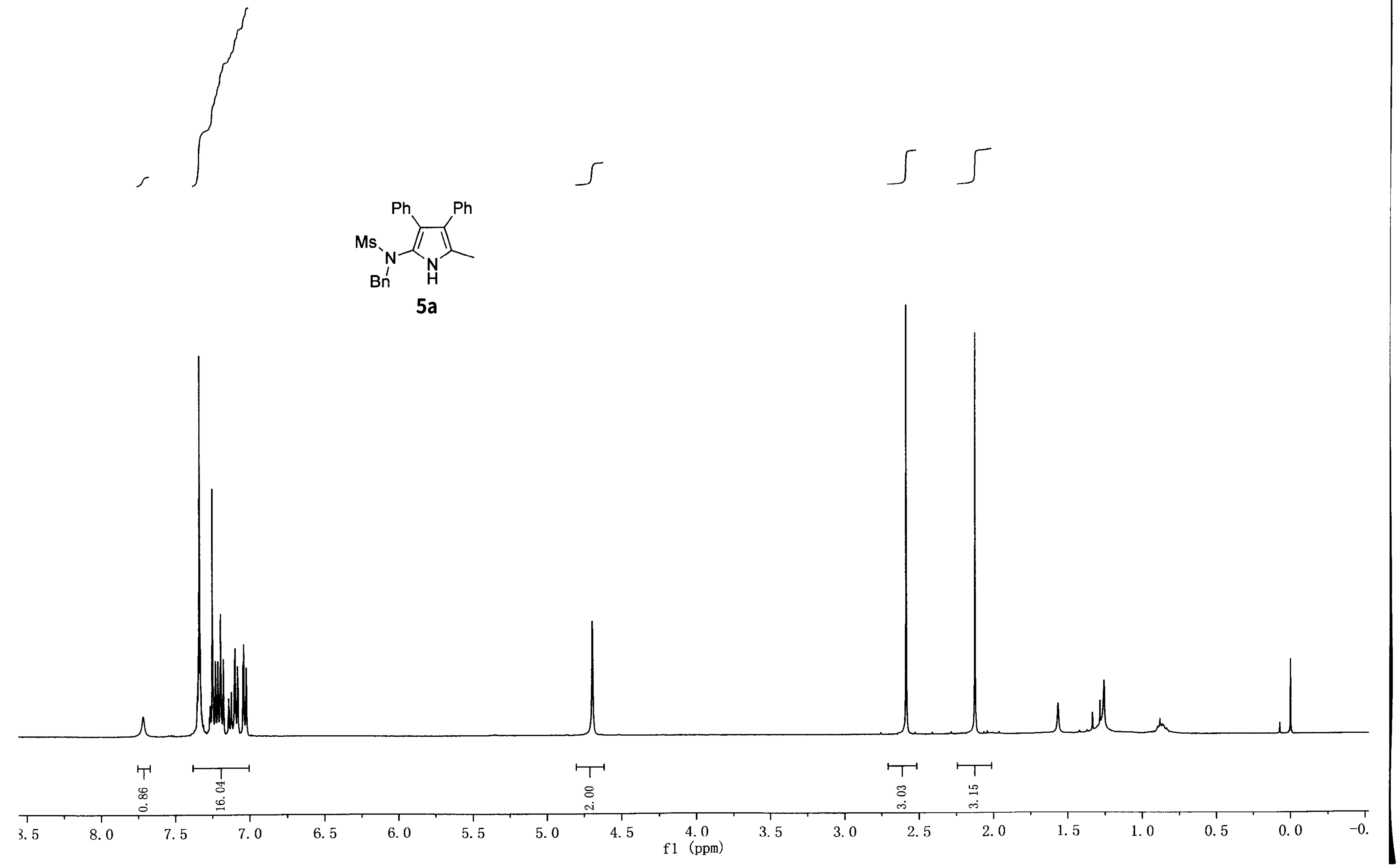
Ph
Ph
Ms
N
Bn
N
H
5a
0.86
16.04
2.00
3.03
3.15
8.0
7.5
7.0
6.5
6.0
5.5
5.0
4.5
4.0
3.5
3.0
2.5
2.0
1.5
1.0
0.5
0.0
f1 (ppm)

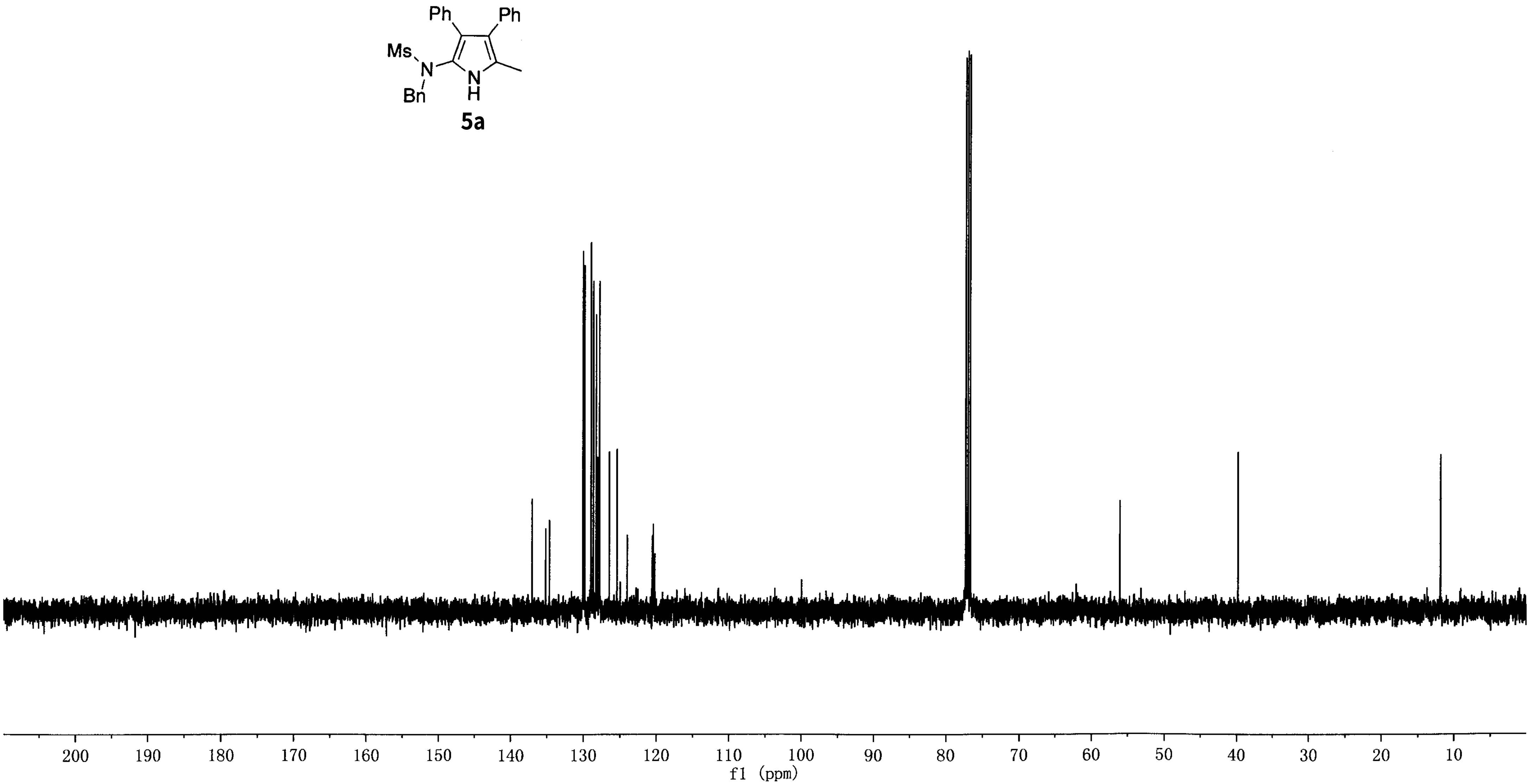

Ph
Ph
Ms
N
Bn
N
H
5a
200
190
180
170
160
150
140
130
120
110
100
90
80
70
60
50
40
30
20
10
f1 (ppm)

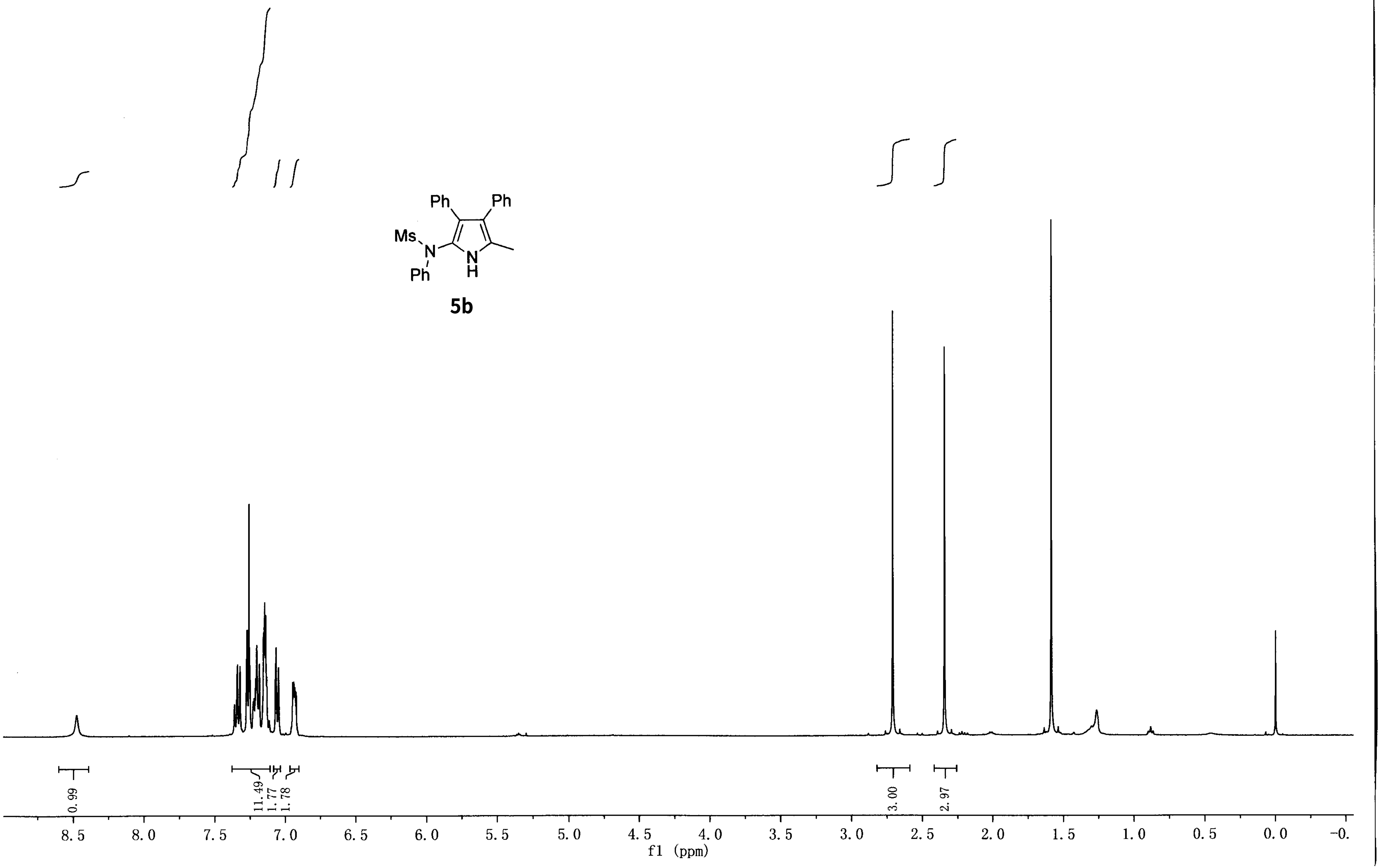

Ph
Ph
Ms
N
N
H
Ph
5b
0.99
11.49
1.77
1.78
3.00
2.97
8.5
8.0
7.5
7.0
6.5
6.0
5.5
5.0
4.5
4.0
3.5
3.0
2.5
2.0
1.5
1.0
0.5
0.0
-0.
f1 (ppm)

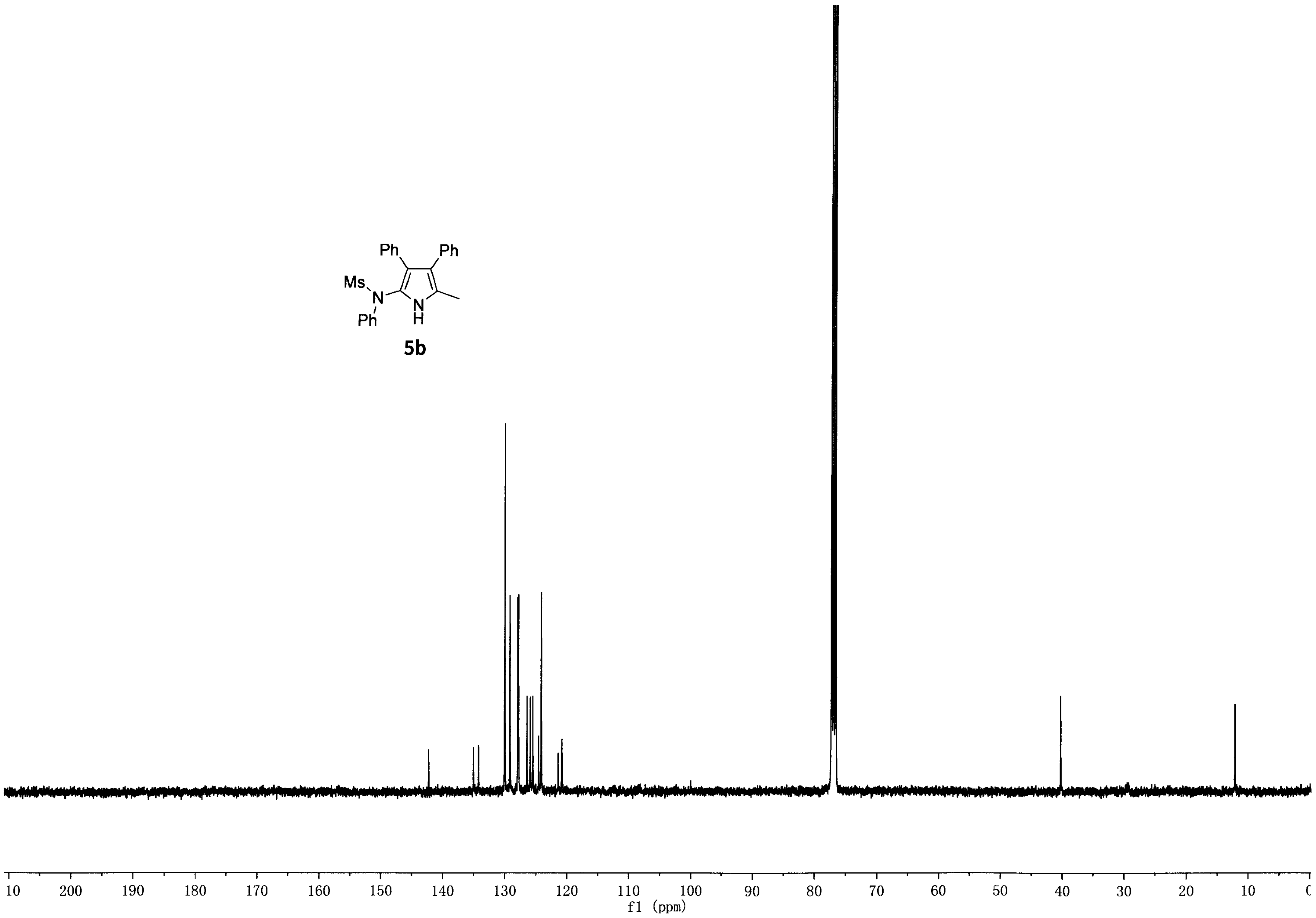
Ph
Ph
Ms
N
Ph
N
H
5b
10
200
190
180
170
160
150
140
130
120
110
100
90
80
70
60
50
40
30
20
10
f1 (ppm)

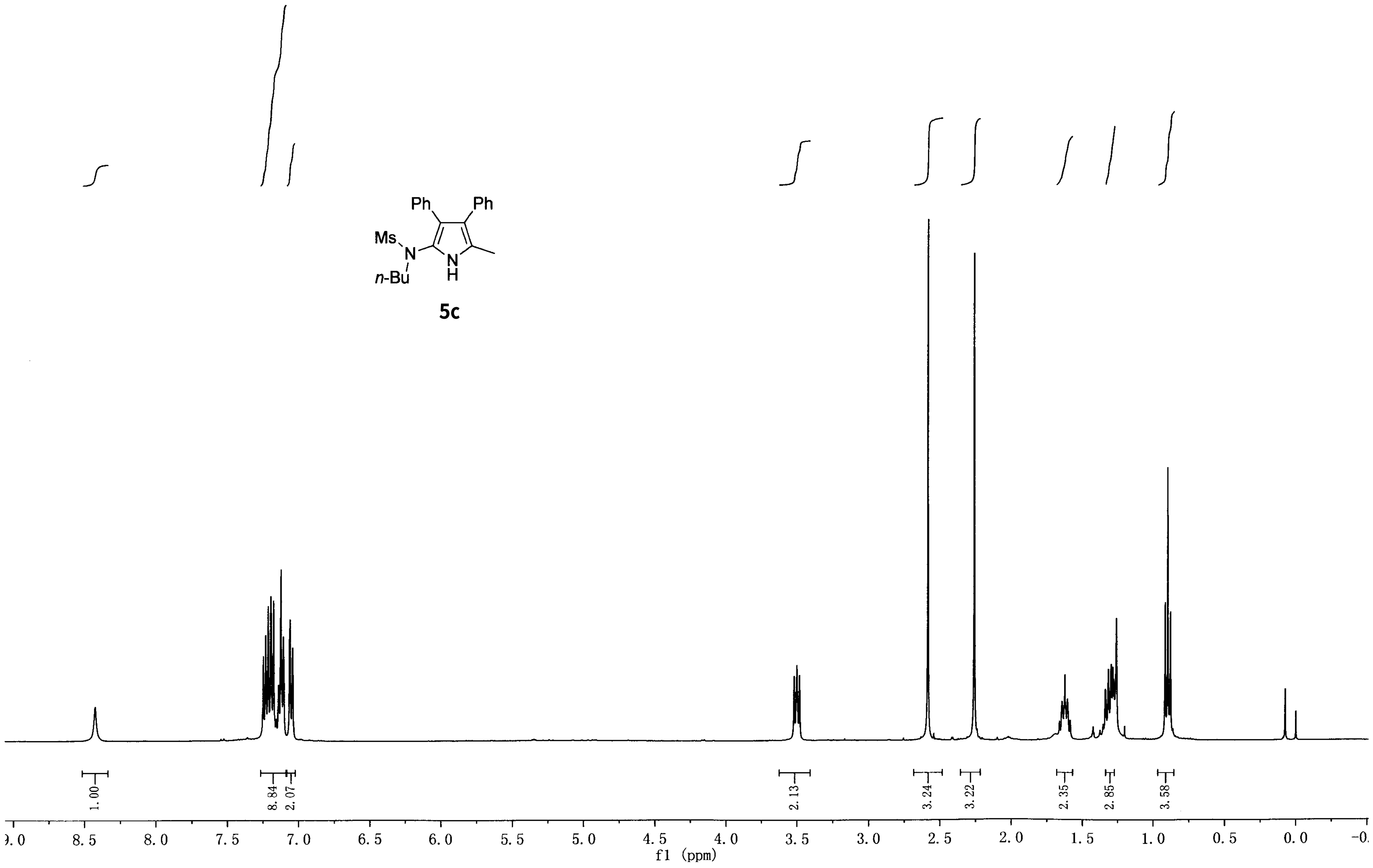

Ph
Ph
Ms
N
n-Bu
N
H
5c
1.00
8.84
2.07
2.13
3.24
3.22
2.35
2.85
3.58
9.0 8.5 8.0 7.5 7.0 6.5 6.0 5.5 5.0 4.5 4.0 3.5 3.0 2.5 2.0 1.5 1.0 0.5 0.0 -0.
f1 (ppm)

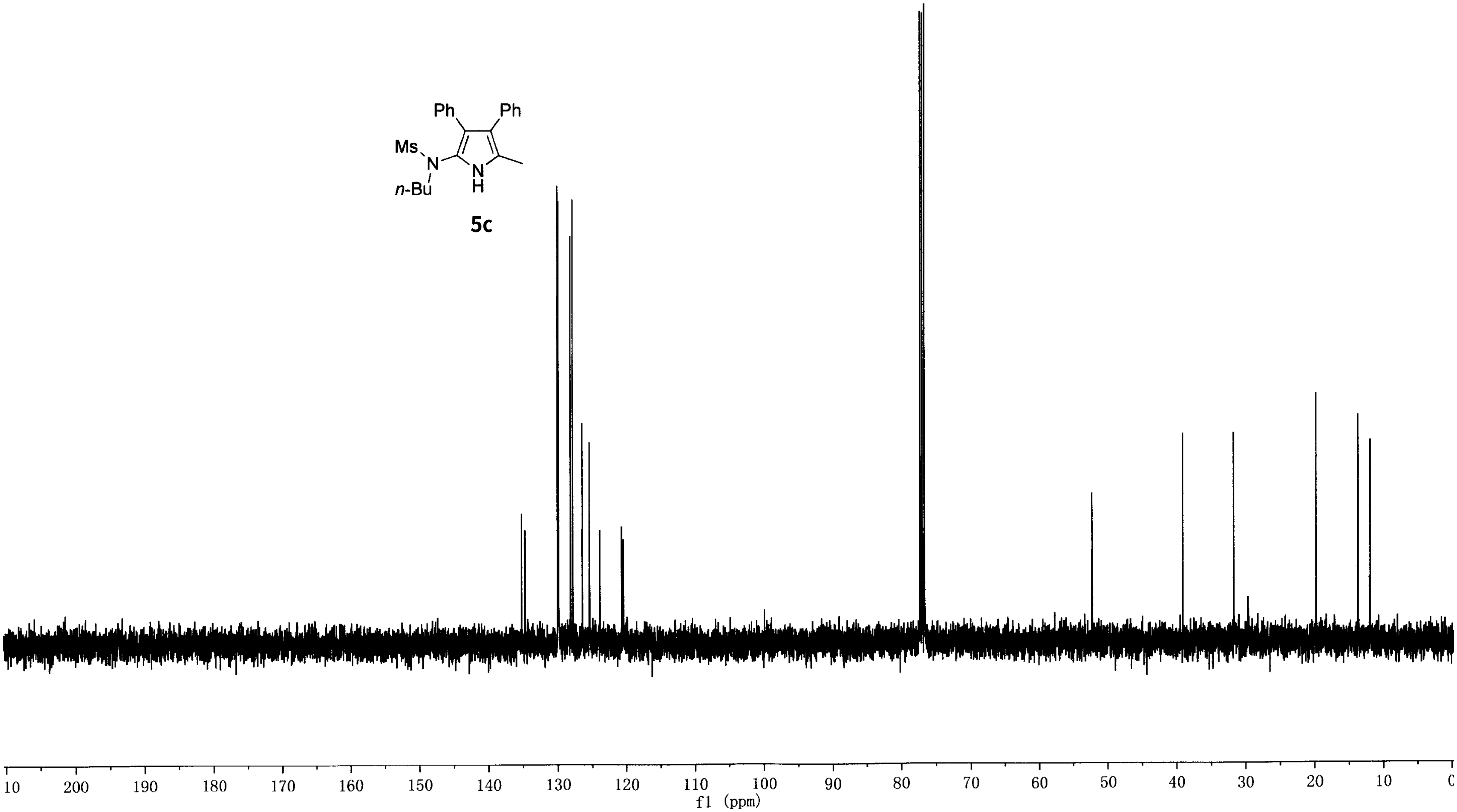
Ph
Ph
Ms
N
N
H
n-Bu
5c
10 200 190 180 170 160 150 140 130 120 110 100 90 80 70 60 50 40 30 20 10
f1 (ppm)

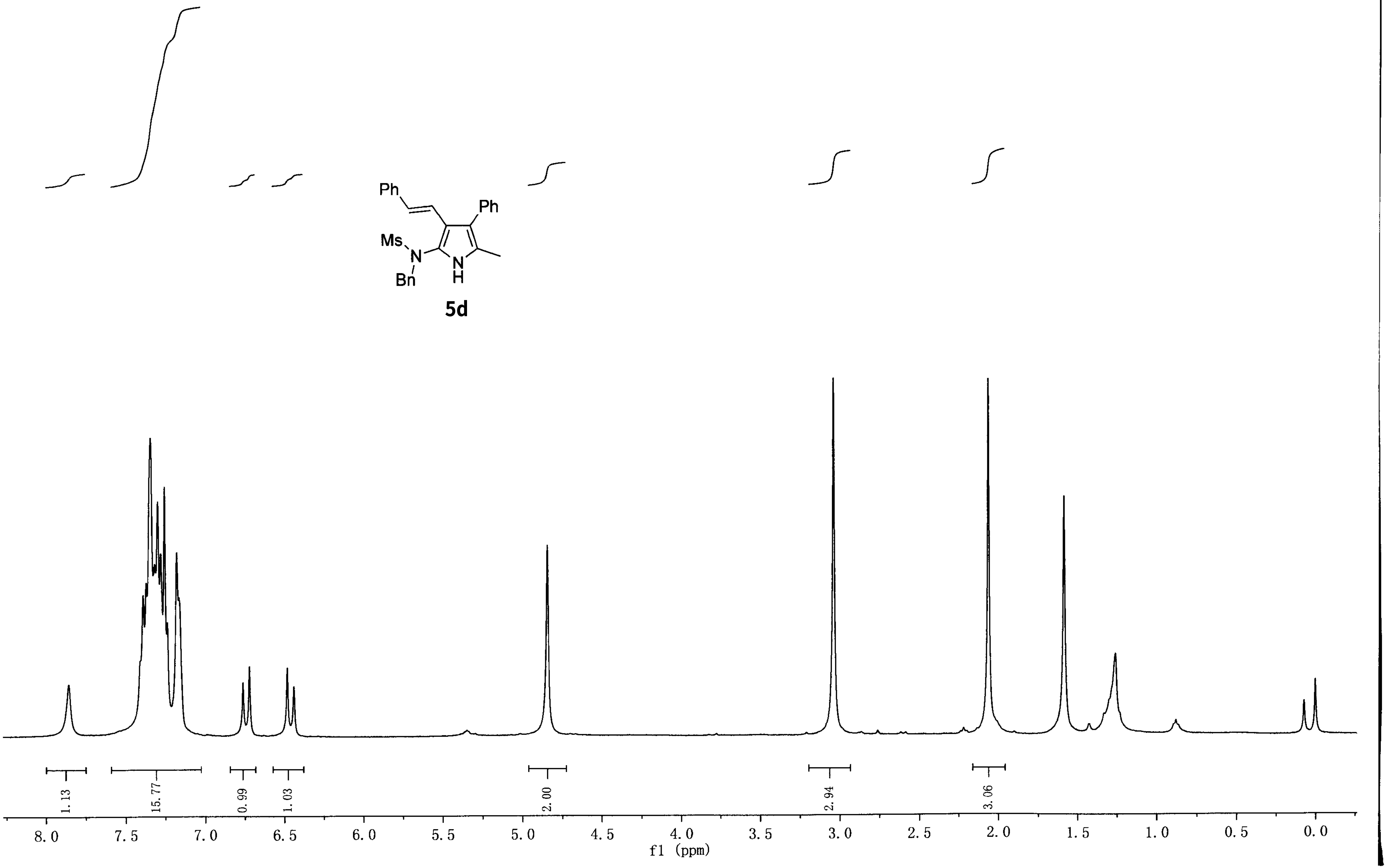

Ph
Ph
Ms
N
Bn
N
H
5d
1.13
15.77
0.99
1.03
2.00
2.94
3.06
8.0
7.5
7.0
6.5
6.0
5.5
5.0
4.5
4.0
3.5
3.0
2.5
2.0
1.5
1.0
0.5
0.0
f1 (ppm)

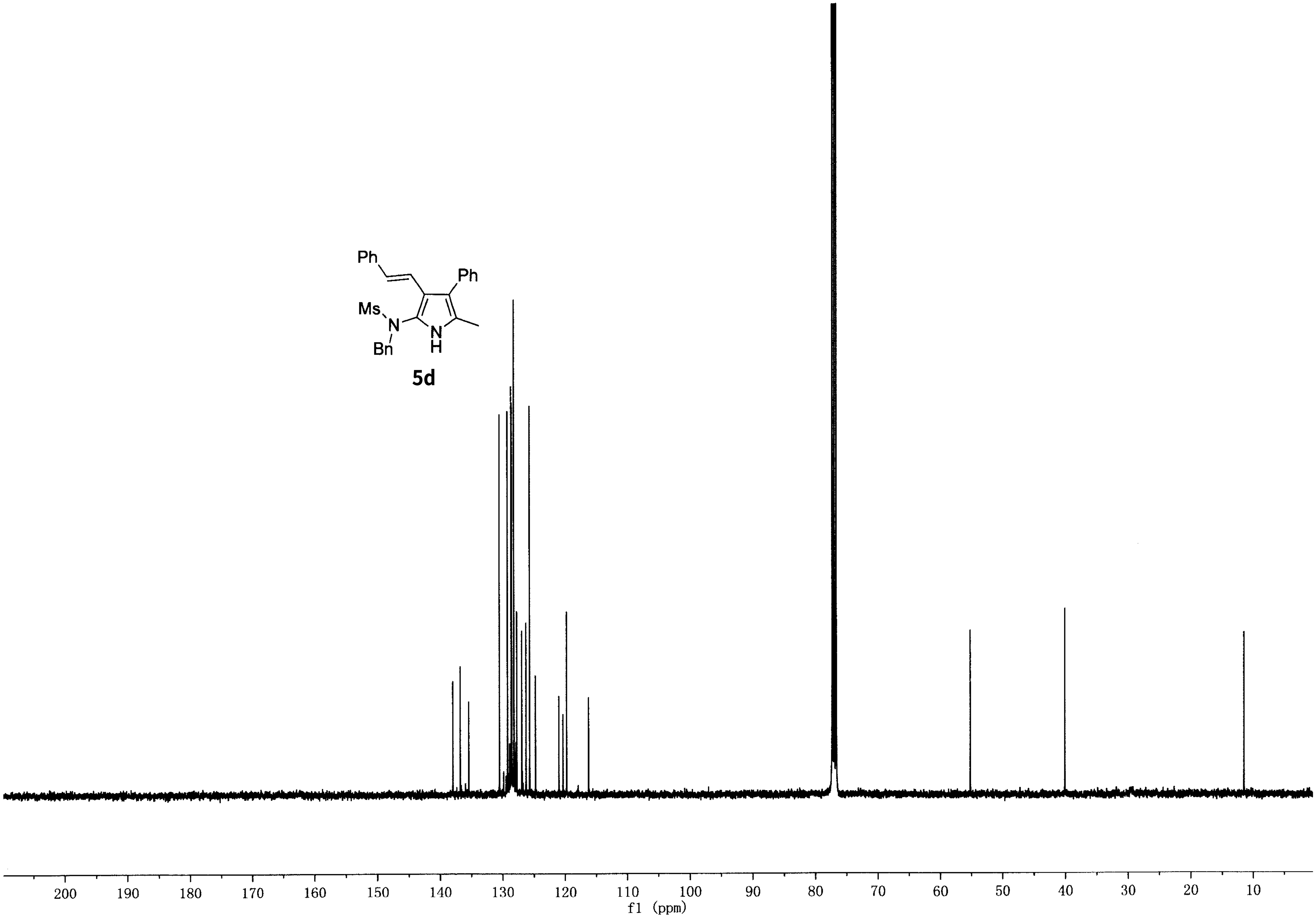

Ph
Ph
Ms
N
Bn
N
H
5d
200
190
180
170
160
150
140
130
120
110
100
90
80
70
60
50
40
30
20
10
f1 (ppm)

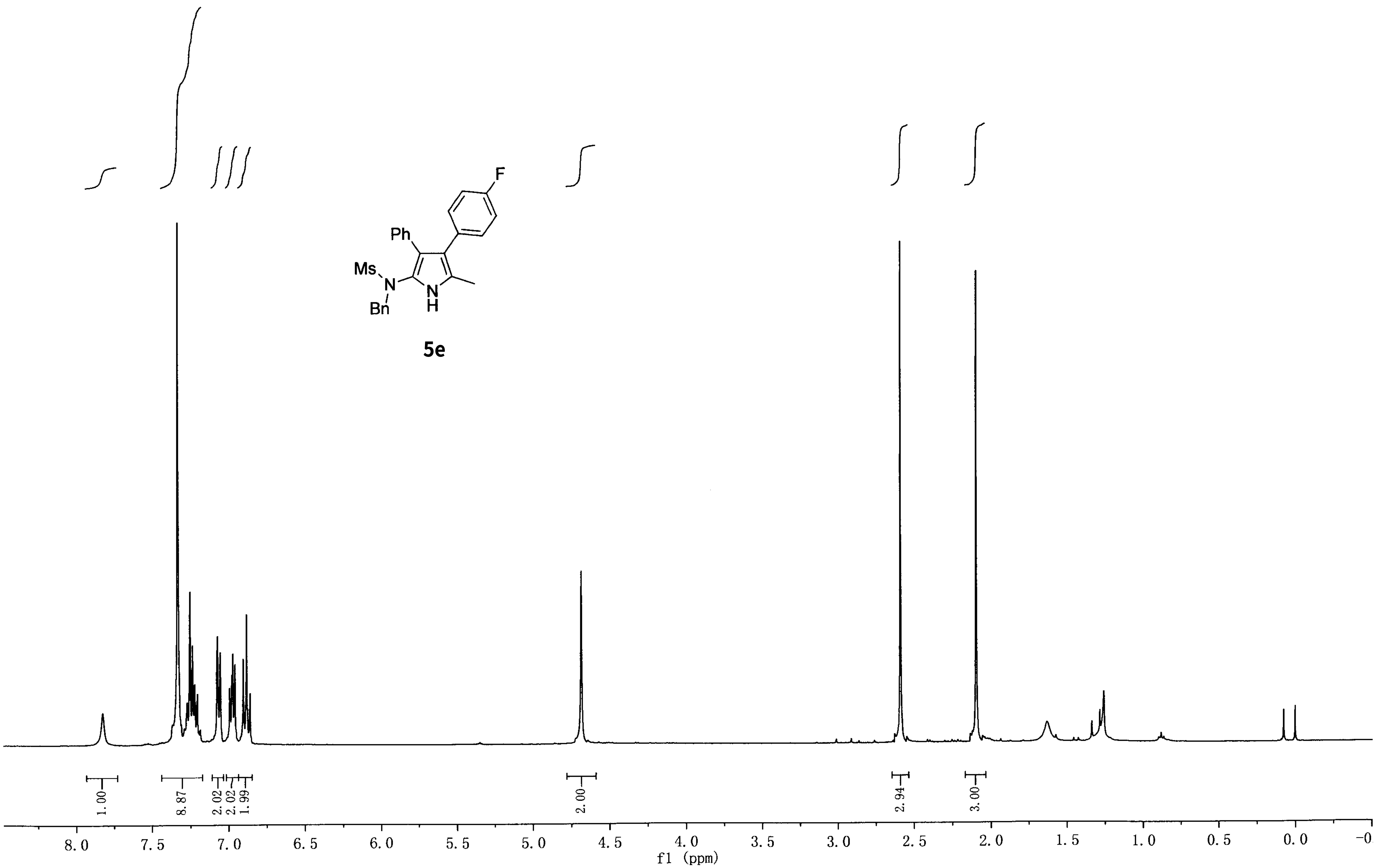
F
Ph
Ms
N
Bn
N
H
5e
1.00
8.87
2.02
2.02
1.99
2.00
2.94
3.00
8.0
7.5
7.0
6.5
6.0
5.5
5.0
4.5
4.0
3.5
3.0
2.5
2.0
1.5
1.0
0.5
0.0
-0
f1 (ppm)

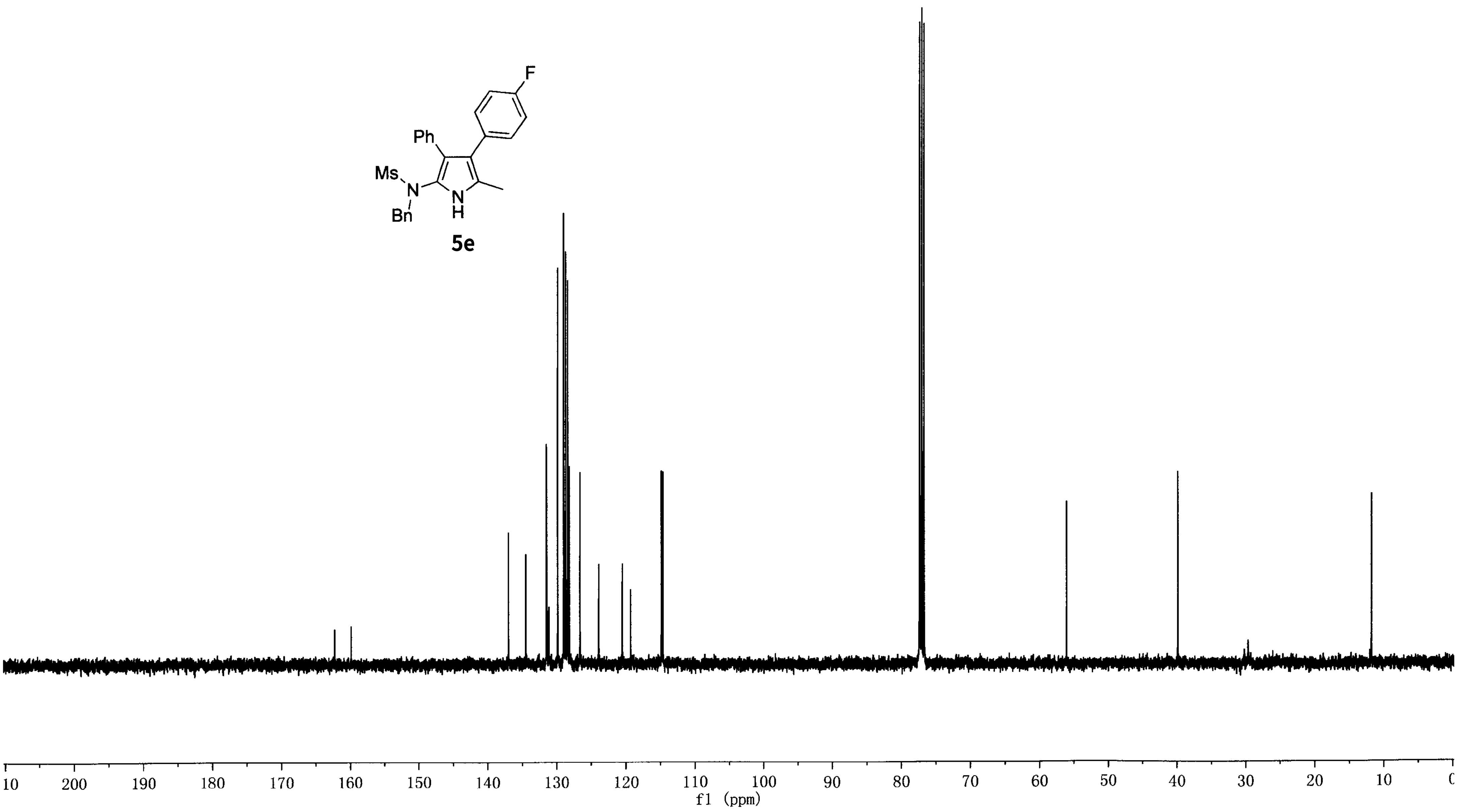
F
Ph
Ms
N
Bn
N
H
5e
10 200 190 180 170 160 150 140 130 120 110 100 90 80 70 60 50 40 30 20 10 (
f1 (ppm)

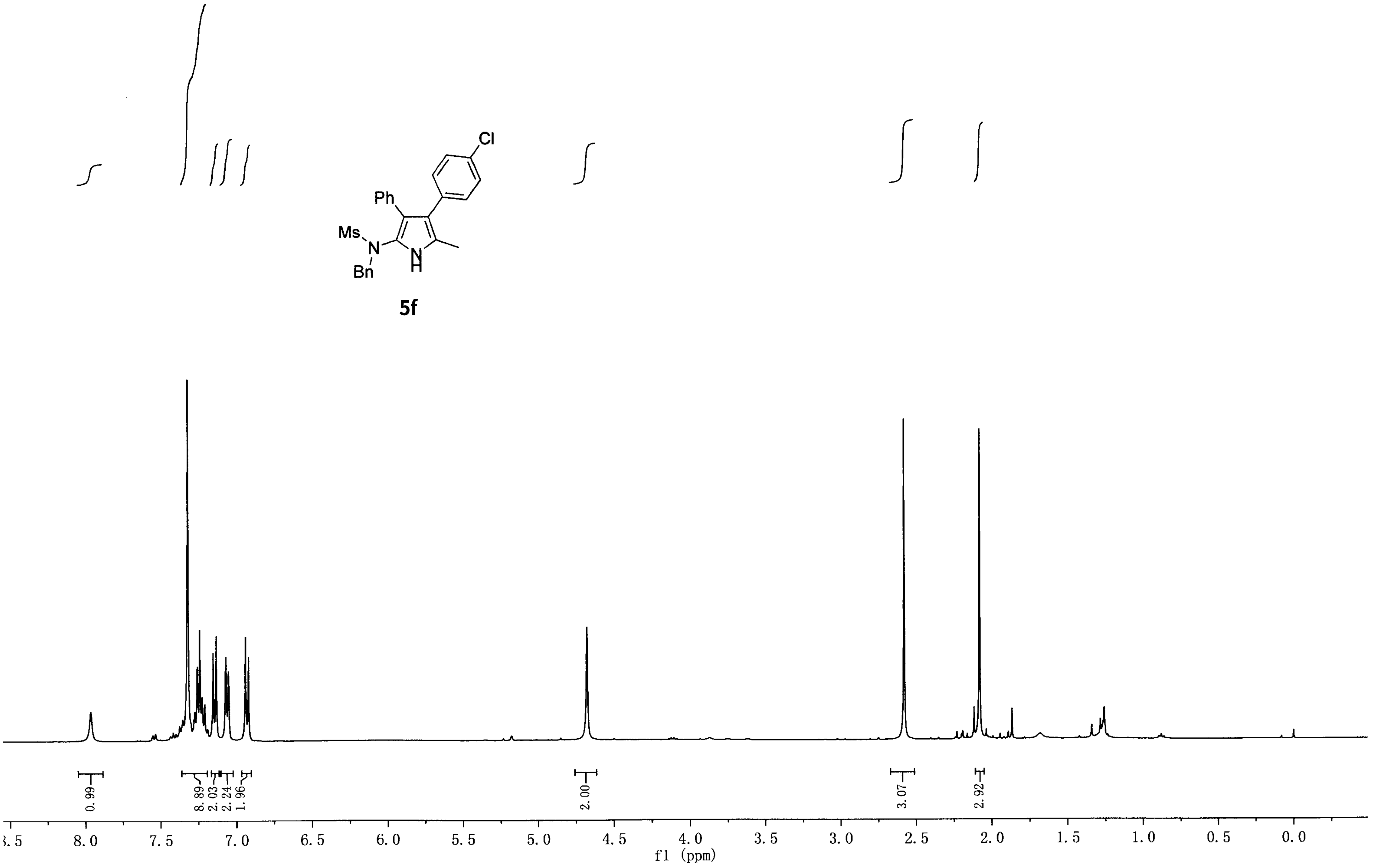
Cl
Ph
Ms
N
Bn
N
H
5f
0.99
8.89
2.03
2.24
1.96
2.00
3.07
2.92
8.0
7.5
7.0
6.5
6.0
5.5
5.0
4.5
4.0
3.5
3.0
2.5
2.0
1.5
1.0
0.5
0.0
f1 (ppm)

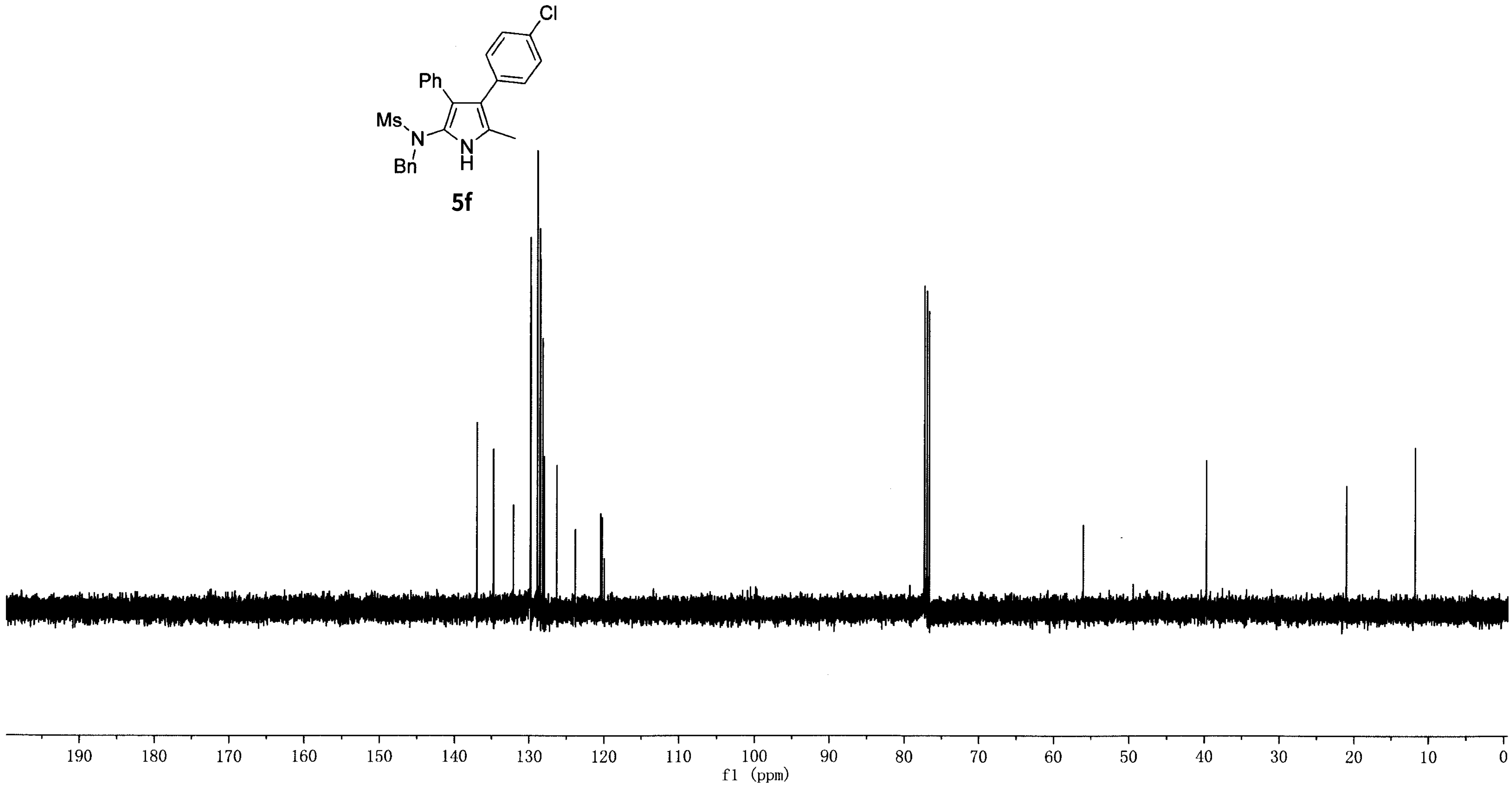
Cl
Ph
Ms
N
Bn
N
H
5f
190 180 170 160 150 140 130 120 110 100 90 80 70 60 50 40 30 20 10 0
f1 (ppm)

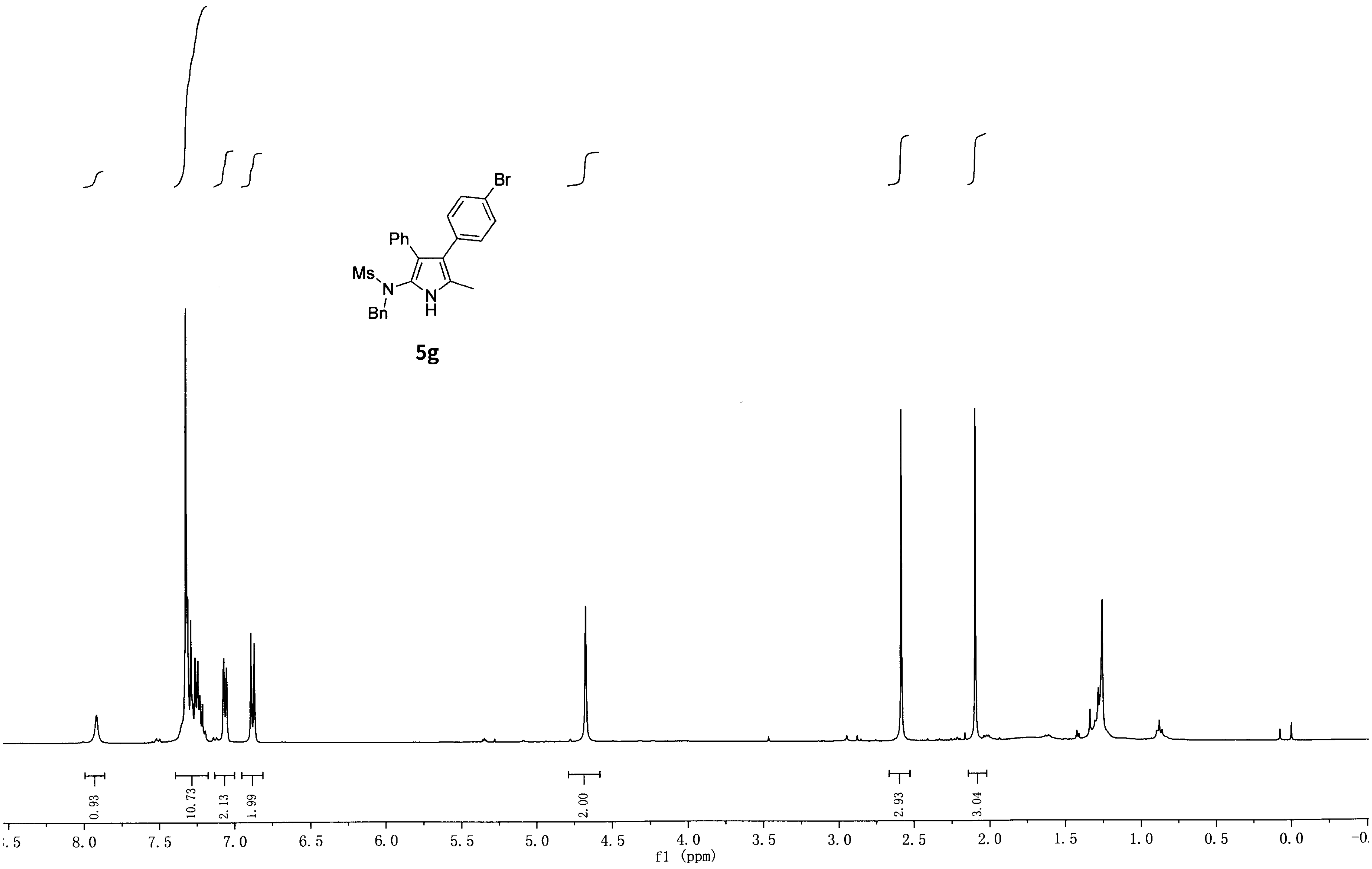

Br
Ph
Ms
N
Bn
N
H
5g
0.93
10.73
2.13
1.99
2.00
2.93
3.04
8.0
7.5
7.0
6.5
6.0
5.5
5.0
4.5
4.0
3.5
3.0
2.5
2.0
1.5
1.0
0.5
0.0
f1 (ppm)

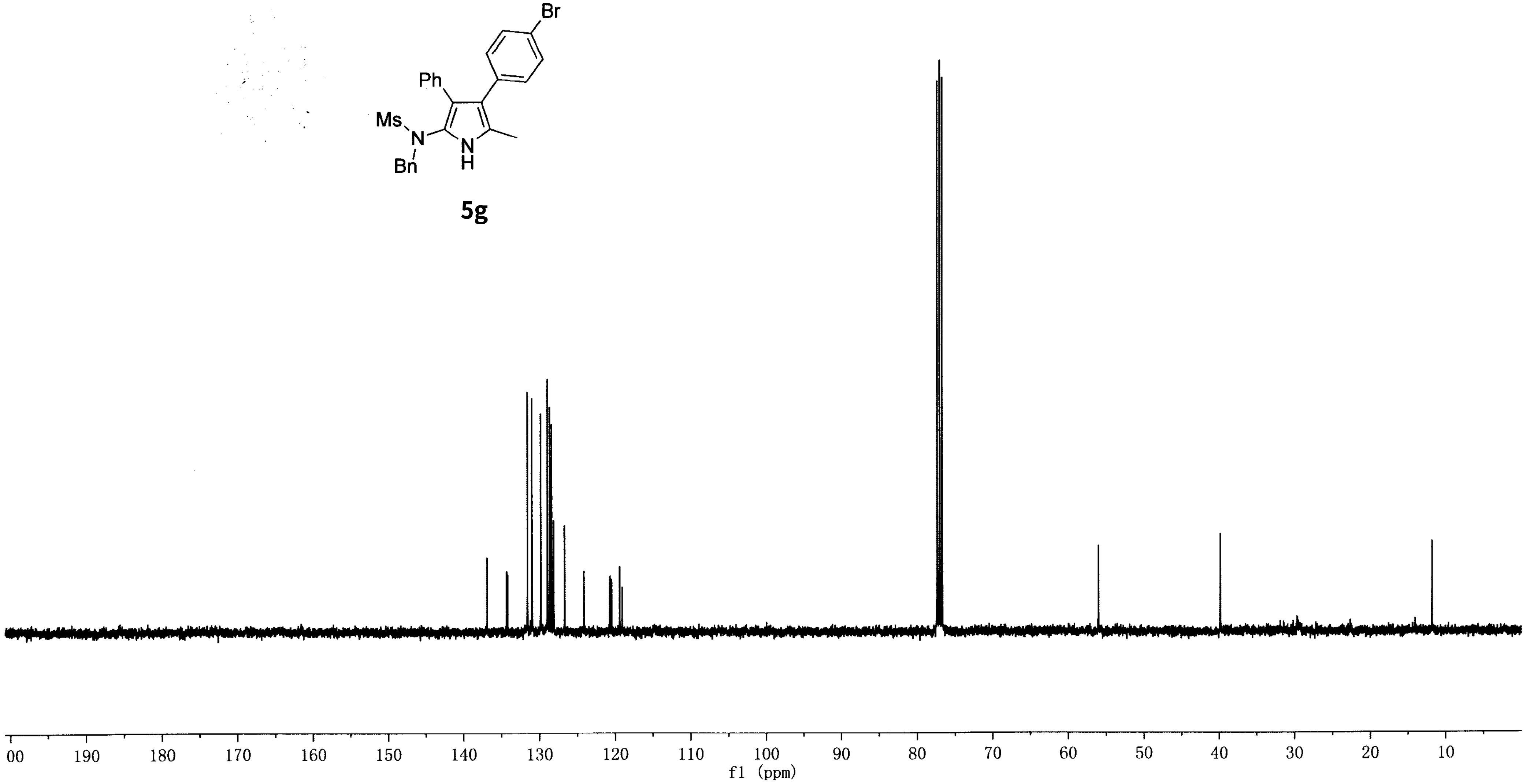
Br
Ph
Ms
N
Bn
N
H

5g
00 190 180 170 160 150 140 130 120 110 100 90 80 70 60 50 40 30 20 10
f1 (ppm)

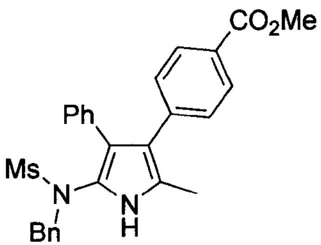
CO2Me
Ph
Ms
N
Bn
N
H

5h

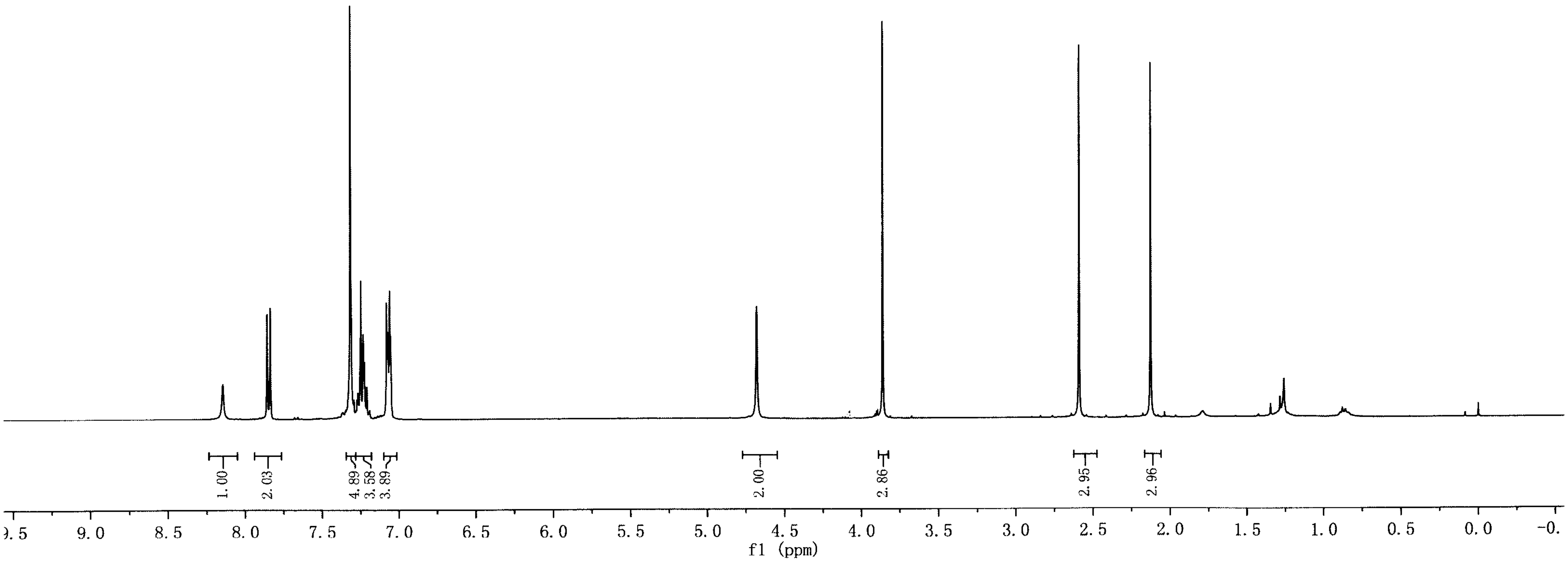
1.00
2.03
4.89
3.58
3.89
2.00
2.86
2.95
2.96
9.0
8.5
8.0
7.5
7.0
6.5
6.0
5.5
5.0
4.5
4.0
3.5
3.0
2.5
2.0
1.5
1.0
0.5
0.0
f1 (ppm)

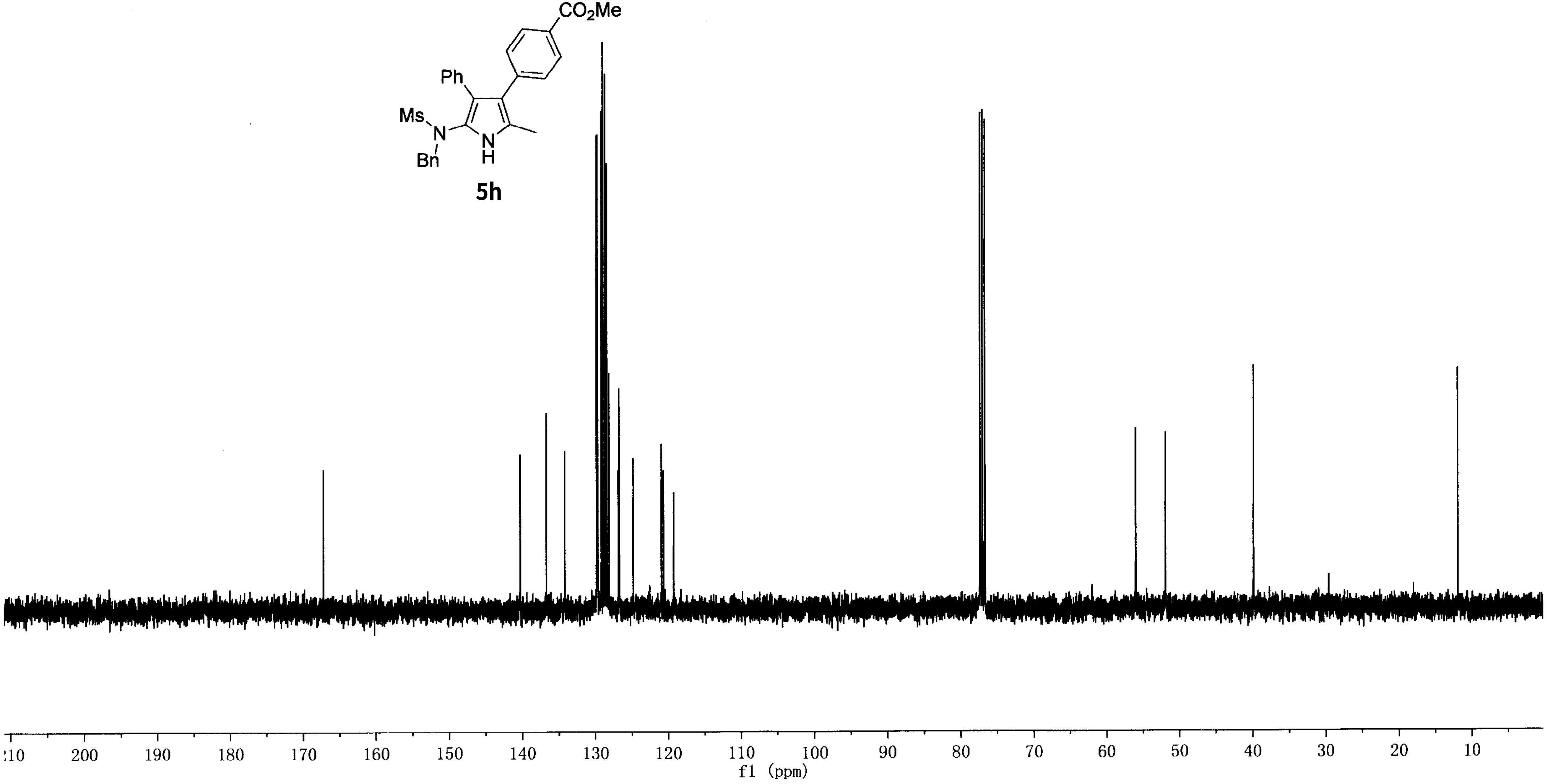

CO2Me
Ph
Ms
N
Bn
N
H
5h
210
200
190
180
170
160
150
140
130
120
110
100
90
80
70
60
50
40
30
20
10
f1 (ppm)

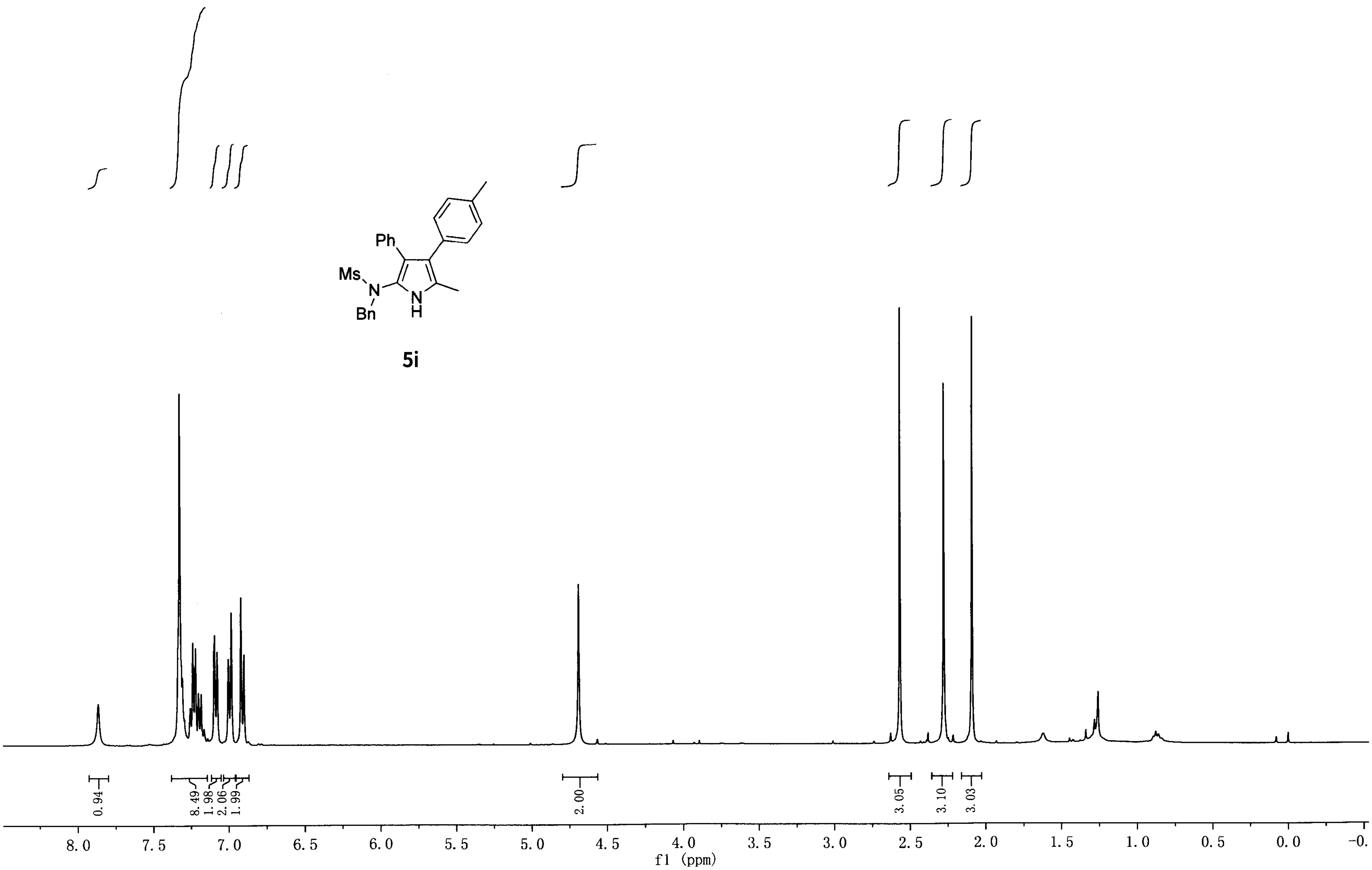
Ph
Ms
N
Bn
N
H
5i
0.94
8.49
1.98
2.06
1.99
2.00
3.05
3.10
3.03
8.0 7.5 7.0 6.5 6.0 5.5 5.0 4.5 4.0 3.5 3.0 2.5 2.0 1.5 1.0 0.5 0.0 -0.
f1 (ppm)

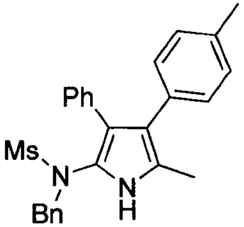
Ph
Ms
N
Bn
N
H

5i

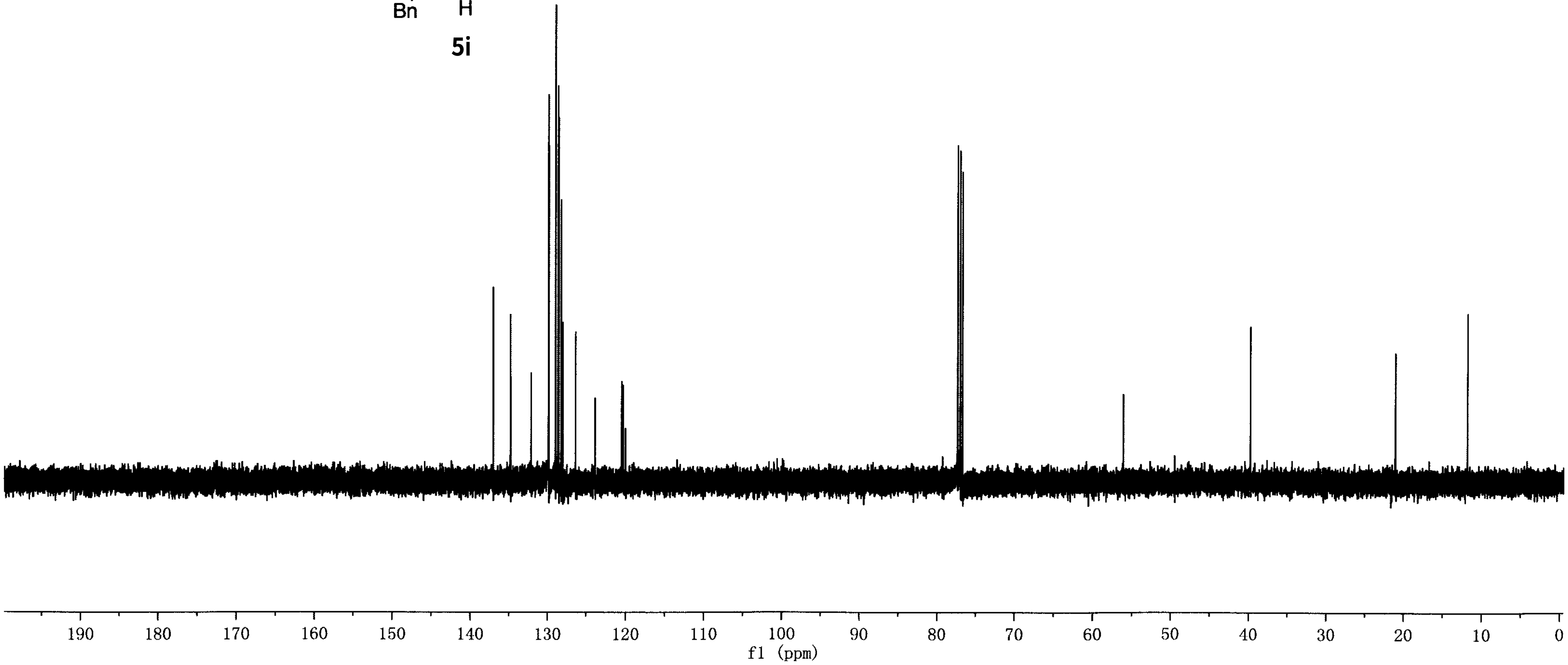
190 180 170 160 150 140 130 120 110 100 90 80 70 60 50 40 30 20 10 0
f1 (ppm)

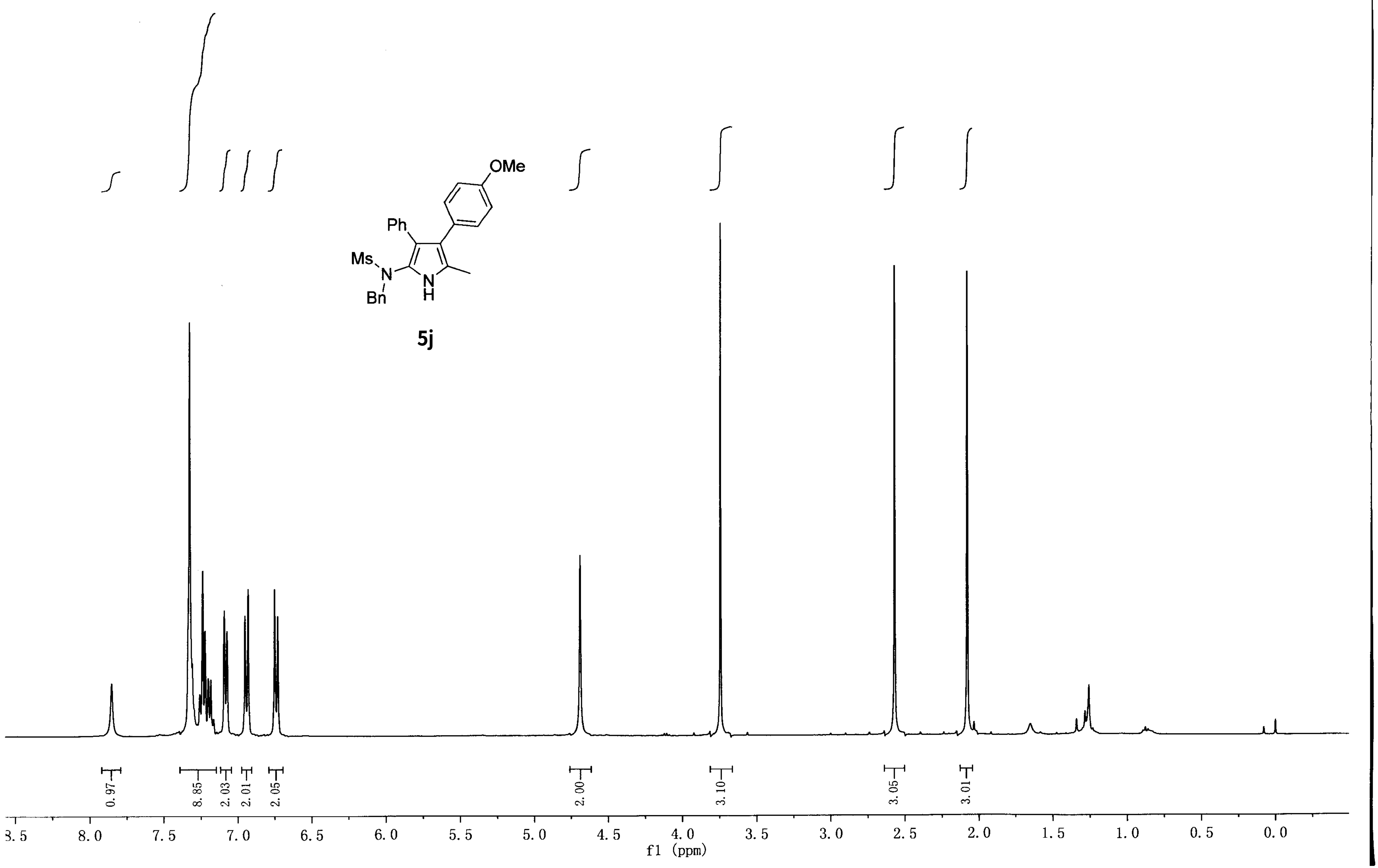

OMe
Ph
Ms
N
Bn
N
H
5j
0.97
8.85
2.03
2.01
2.05
2.00
3.10
3.05
3.01
8.5
8.0
7.5
7.0
6.5
6.0
5.5
5.0
4.5
4.0
3.5
3.0
2.5
2.0
1.5
1.0
0.5
0.0
f1 (ppm)

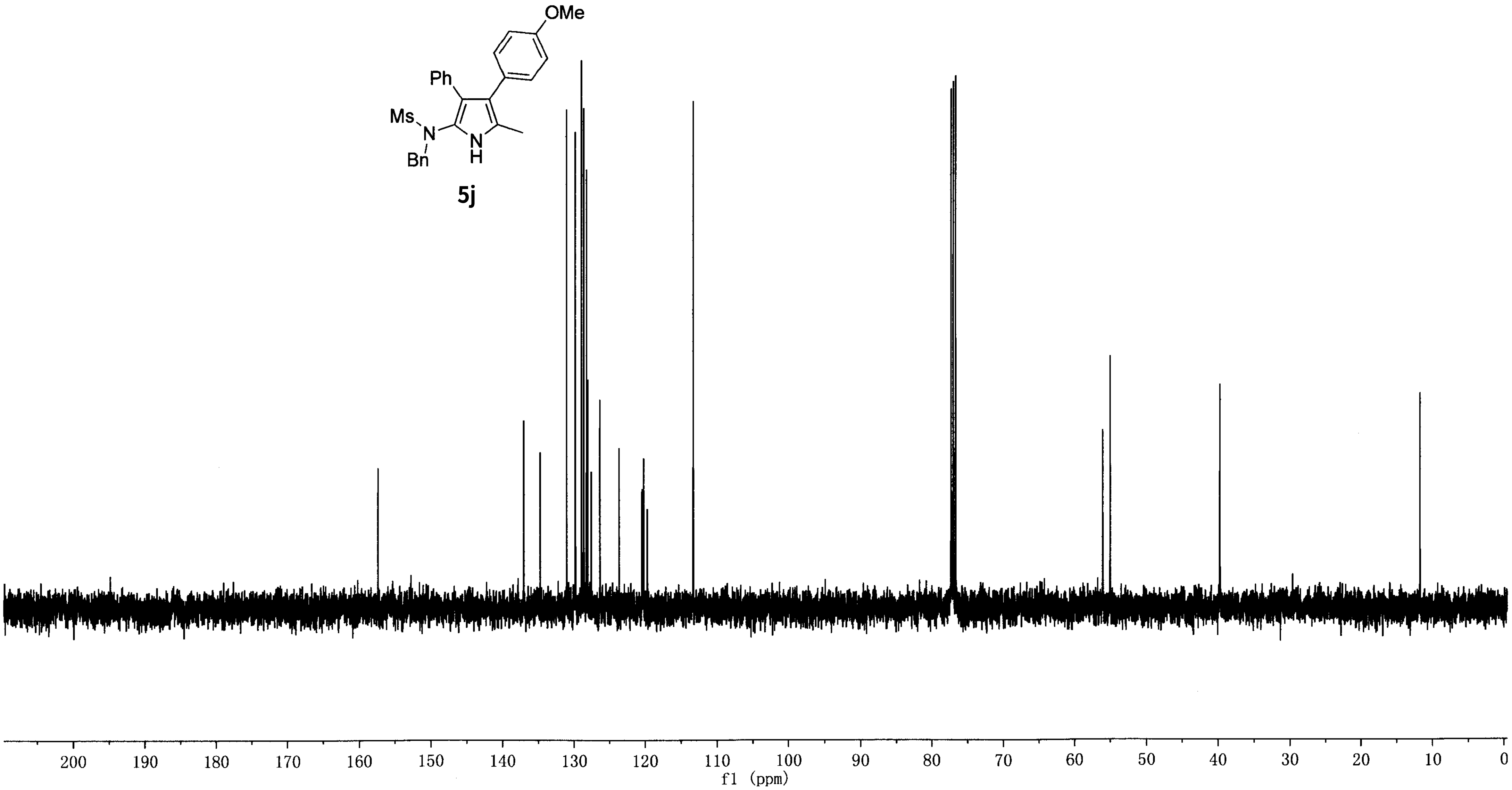
OMe
Ph
Ms
N
Bn
N
H
5j
200 190 180 170 160 150 140 130 120 110 100 90 80 70 60 50 40 30 20 10 0
f1 (ppm)

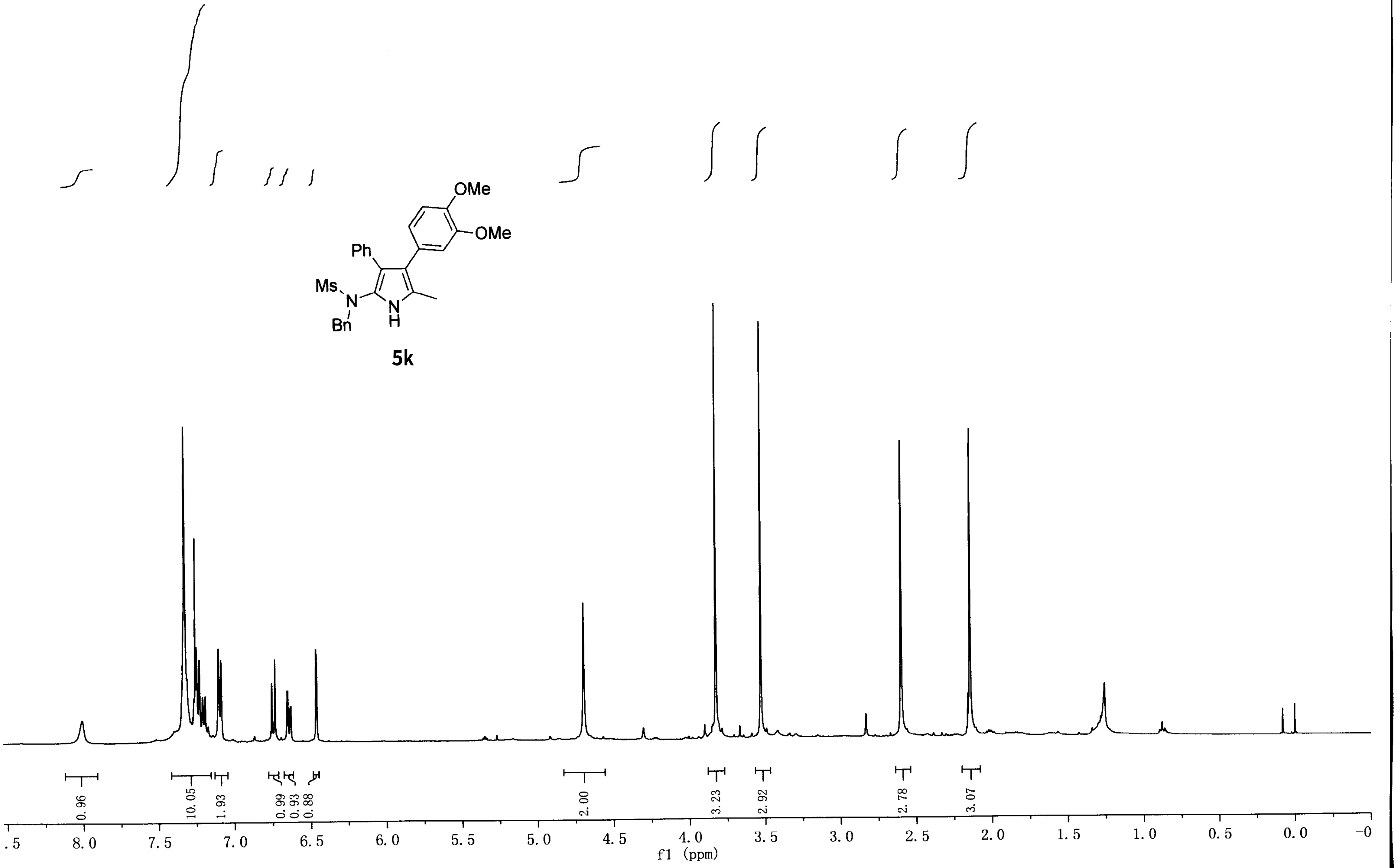
OMe
OMe
Ph
Ms
N
Bn
N
H
5k
0.96
10.05
1.93
0.99
0.93
0.88
2.00
3.23
2.92
2.78
3.07
.5
8.0
7.5
7.0
6.5
6.0
5.5
5.0
4.5
4.0
3.5
3.0
2.5
2.0
1.5
1.0
0.5
0.0
-0
f1 (ppm)

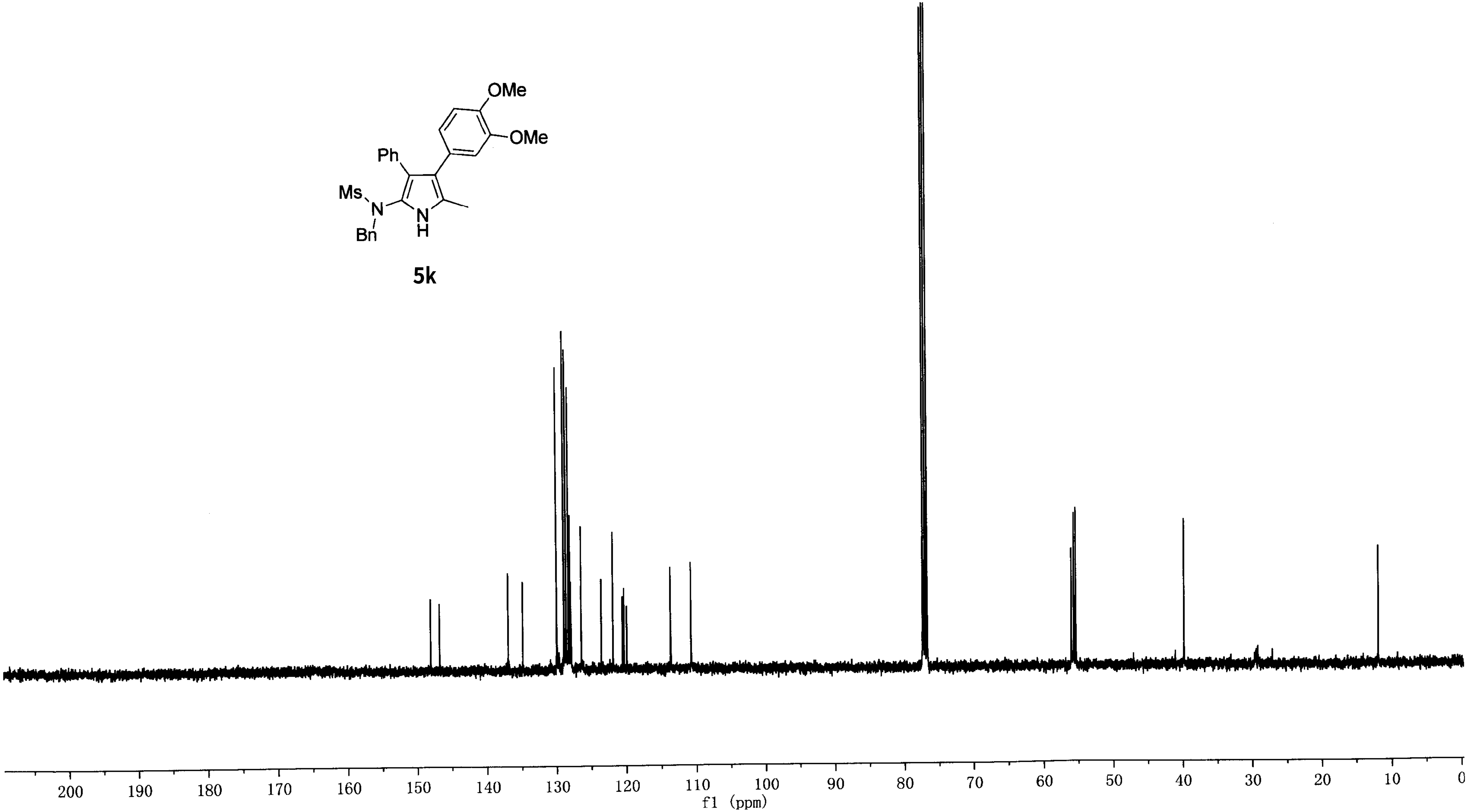
OMe
OMe
Ph
Ms
N
Bn
N
H
200
190
180
170
160
150
140
130
120
110
100
90
80
70
60
50
40
30
20
10
0
f1 (ppm)

5k

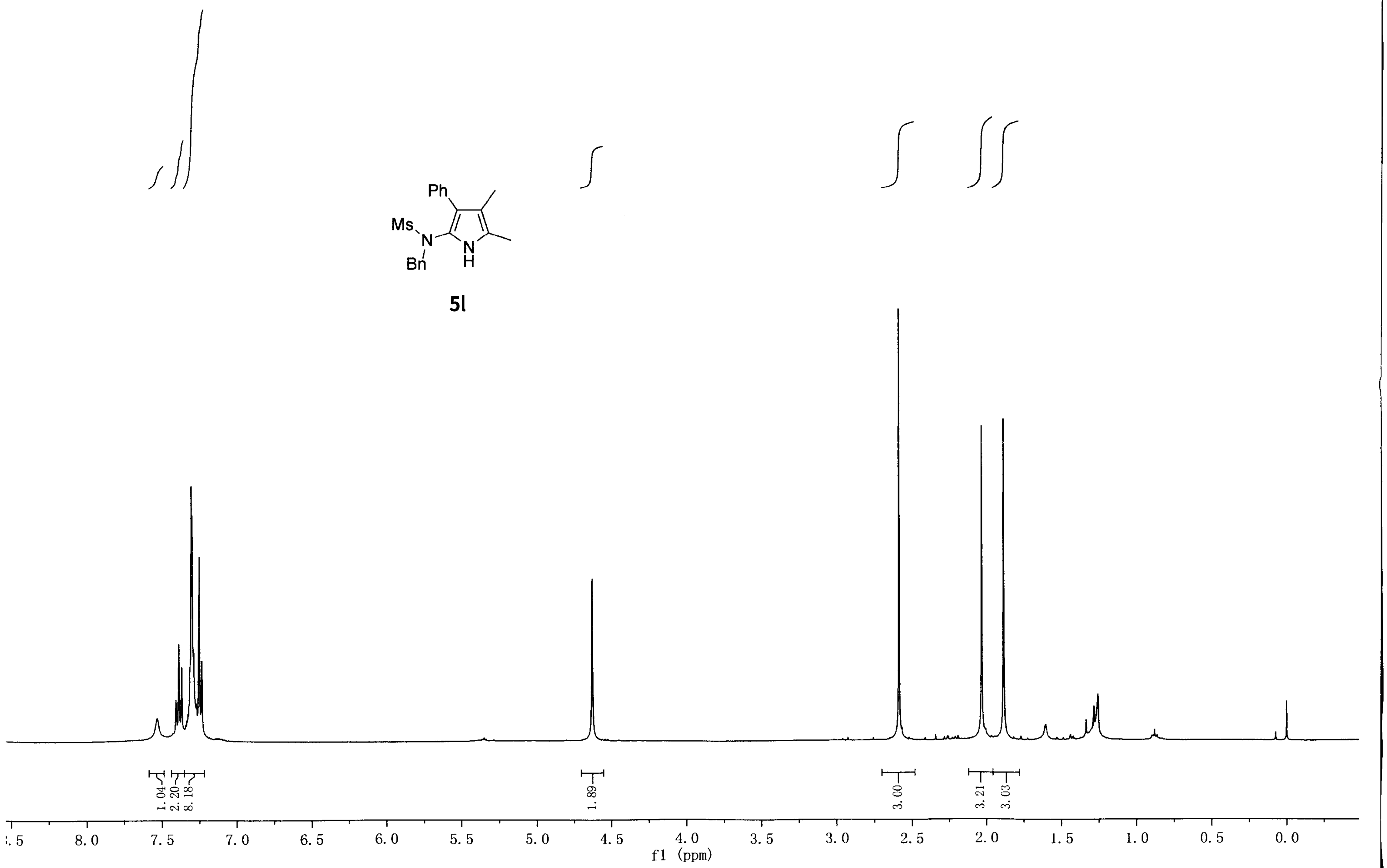
Ph
Ms
N
Bn
N
H
5l
1.04
2.20
8.18
1.89
3.00
3.21
3.03
8.0
7.5
7.0
6.5
6.0
5.5
5.0
4.5
4.0
3.5
3.0
2.5
2.0
1.5
1.0
0.5
0.0
f1 (ppm)

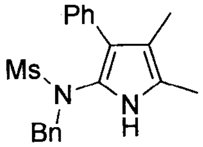
Ph
Ms
N
Bn
N
H

5l

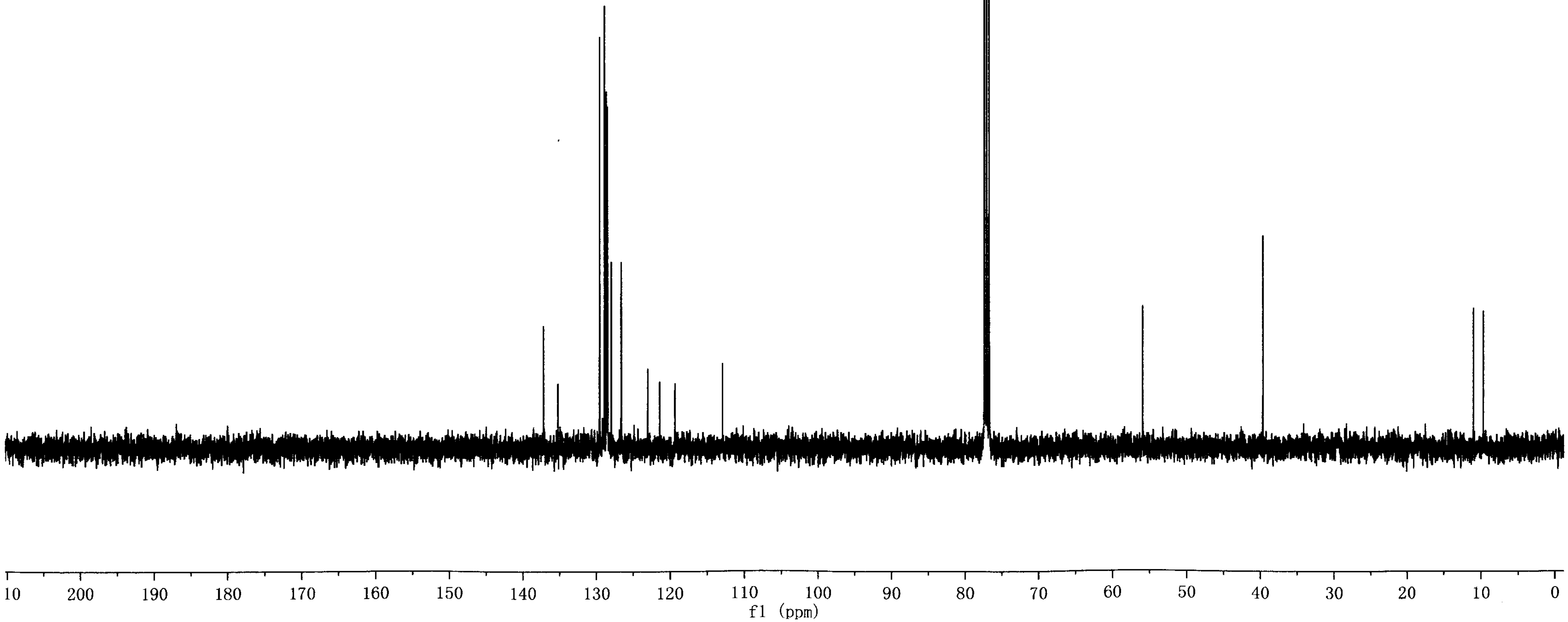
10
200
190
180
170
160
150
140
130
120
110
100
90
80
70
60
50
40
30
20
10
0
f1 (ppm)

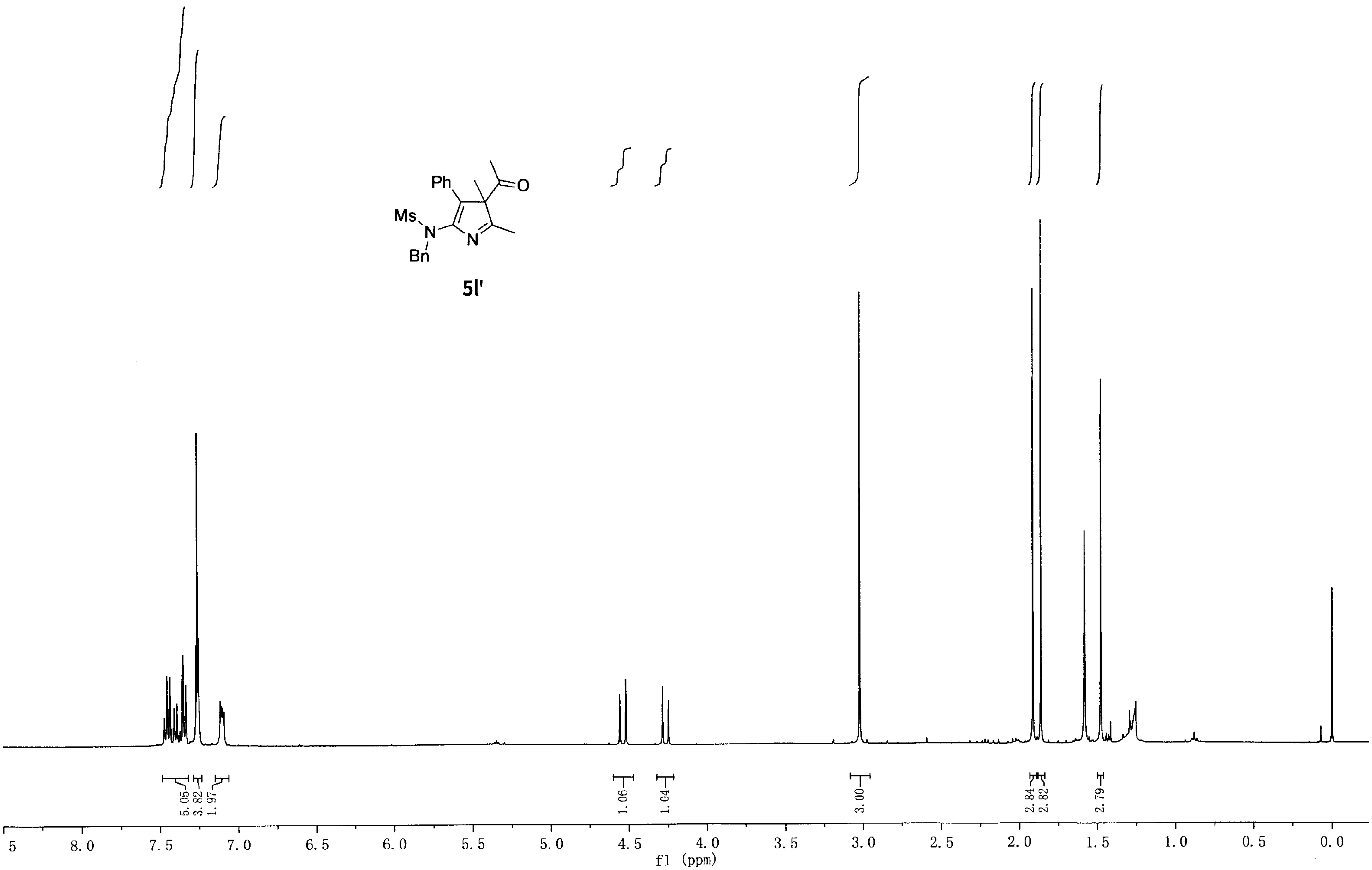

Ph
Ms
N
Bn
N
O
5.05
3.82
1.97
1.06
1.04
3.00
2.84
2.82
2.79
5
8.0
7.5
7.0
6.5
6.0
5.5
5.0
4.5
4.0
3.5
3.0
2.5
2.0
1.5
1.0
0.5
0.0
f1 (ppm)

5l'

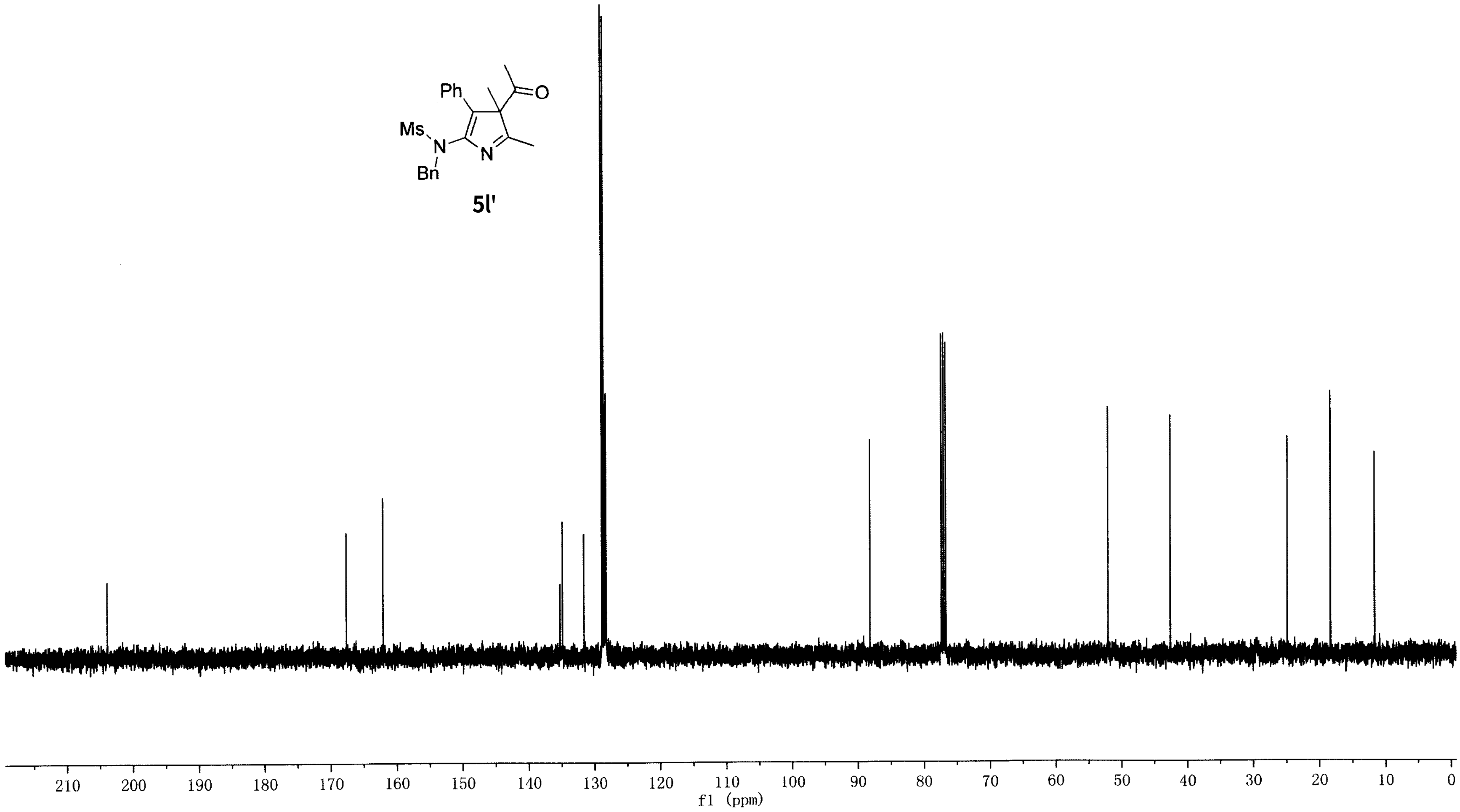
Ph
Ms
N
Bn
N
O
5l'
210
200
190
180
170
160
150
140
130
120
110
100
90
80
70
60
50
40
30
20
10
0
f1 (ppm)

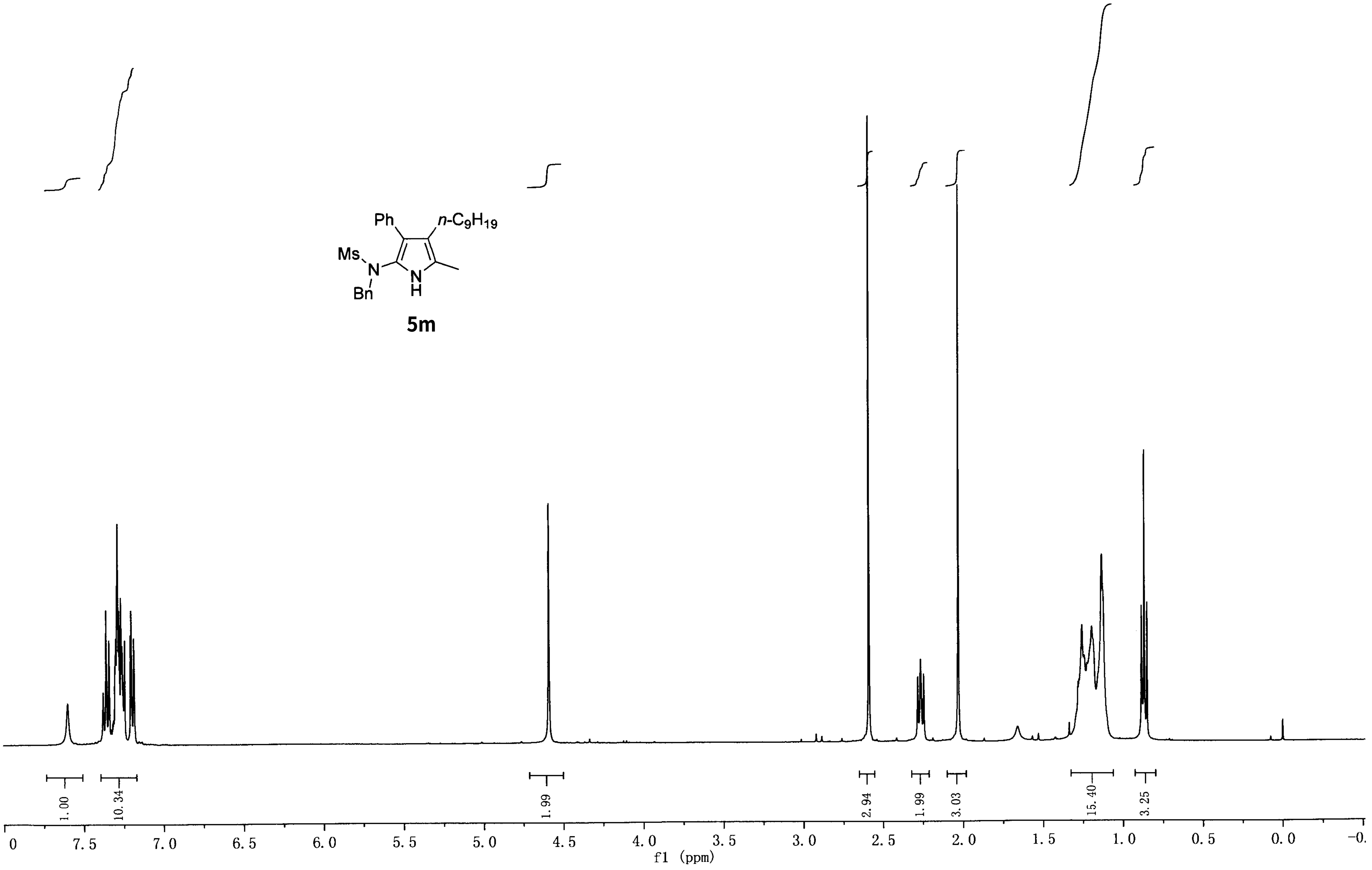

Ph
n-C9H19
Ms
N
Bn
N
H
5m
1.00
10.34
1.99
2.94
1.99
3.03
15.40
3.25
0
7.5
7.0
6.5
6.0
5.5
5.0
4.5
4.0
3.5
3.0
2.5
2.0
1.5
1.0
0.5
0.0
-0.
f1 (ppm)

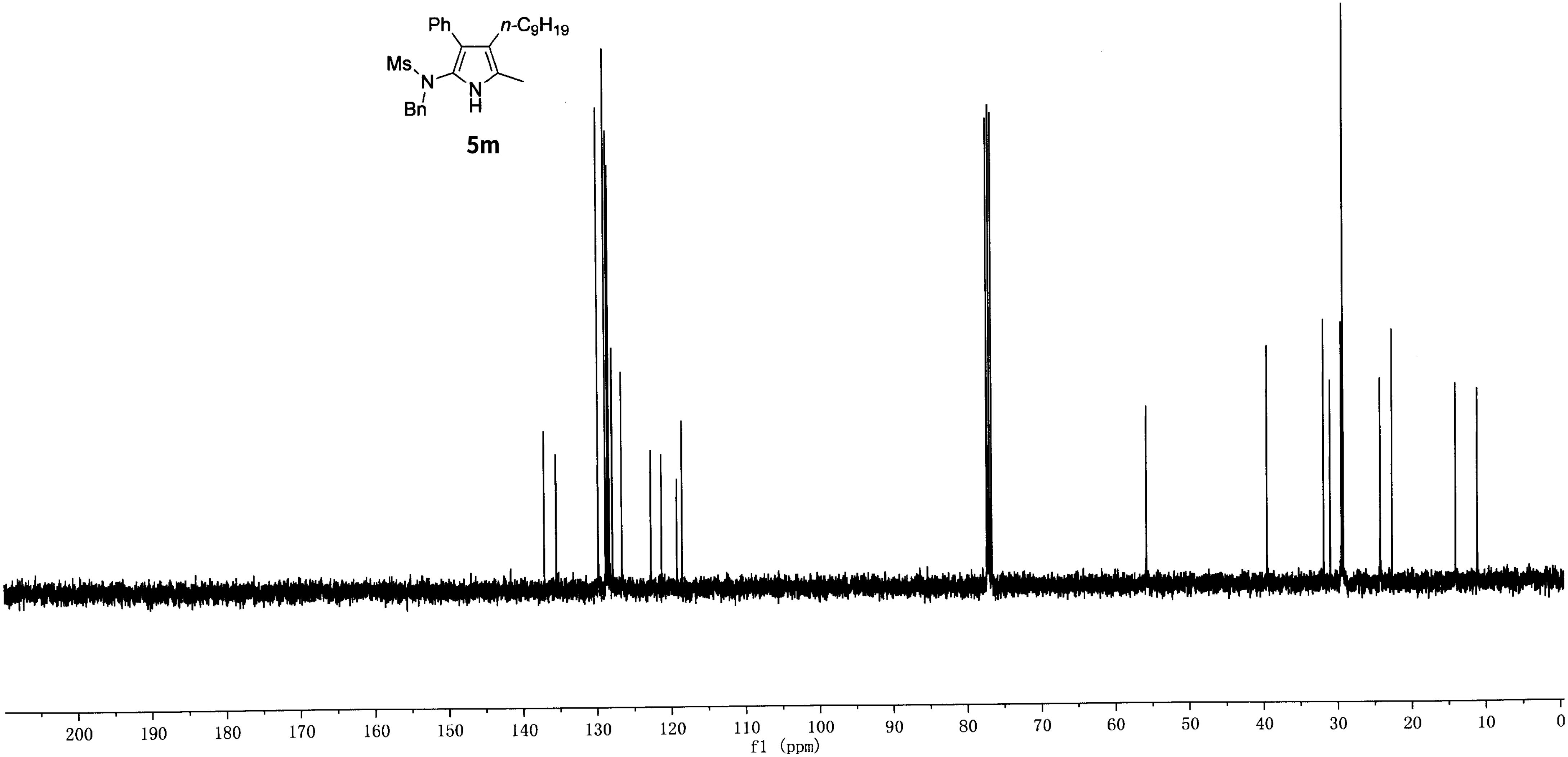

Ph
n-C9H19
Ms
N
Bn
N
H
200
190
180
170
160
150
140
130
120
110
100
90
80
70
60
50
40
30
20
10
0
f1 (ppm)

5m

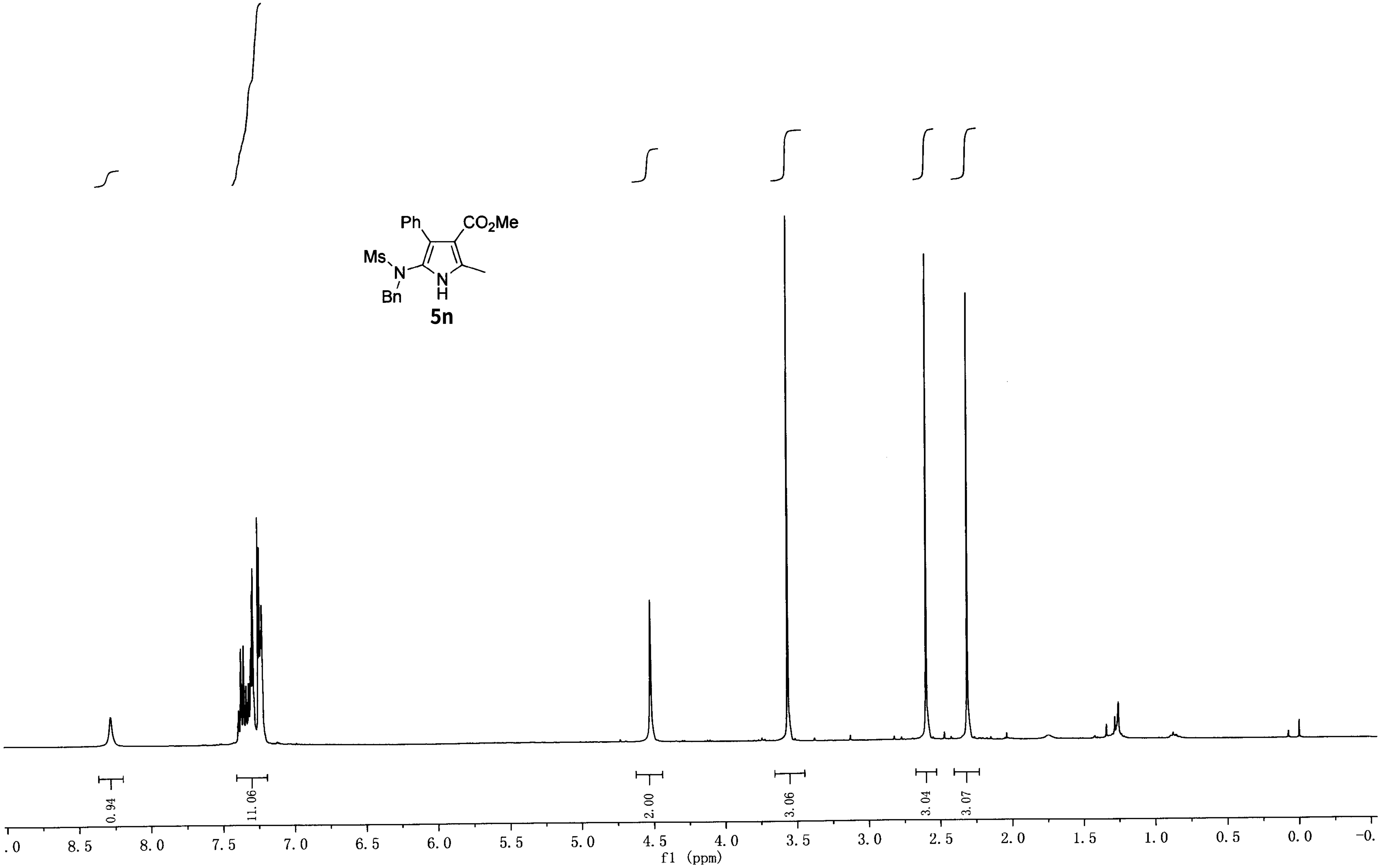
Ph
CO2Me
Ms
N
Bn
N
H
5n
0.94
11.06
2.00
3.06
3.04
3.07
.0
8.5
8.0
7.5
7.0
6.5
6.0
5.5
5.0
4.5
4.0
3.5
3.0
2.5
2.0
1.5
1.0
0.5
0.0
-0.
f1 (ppm)

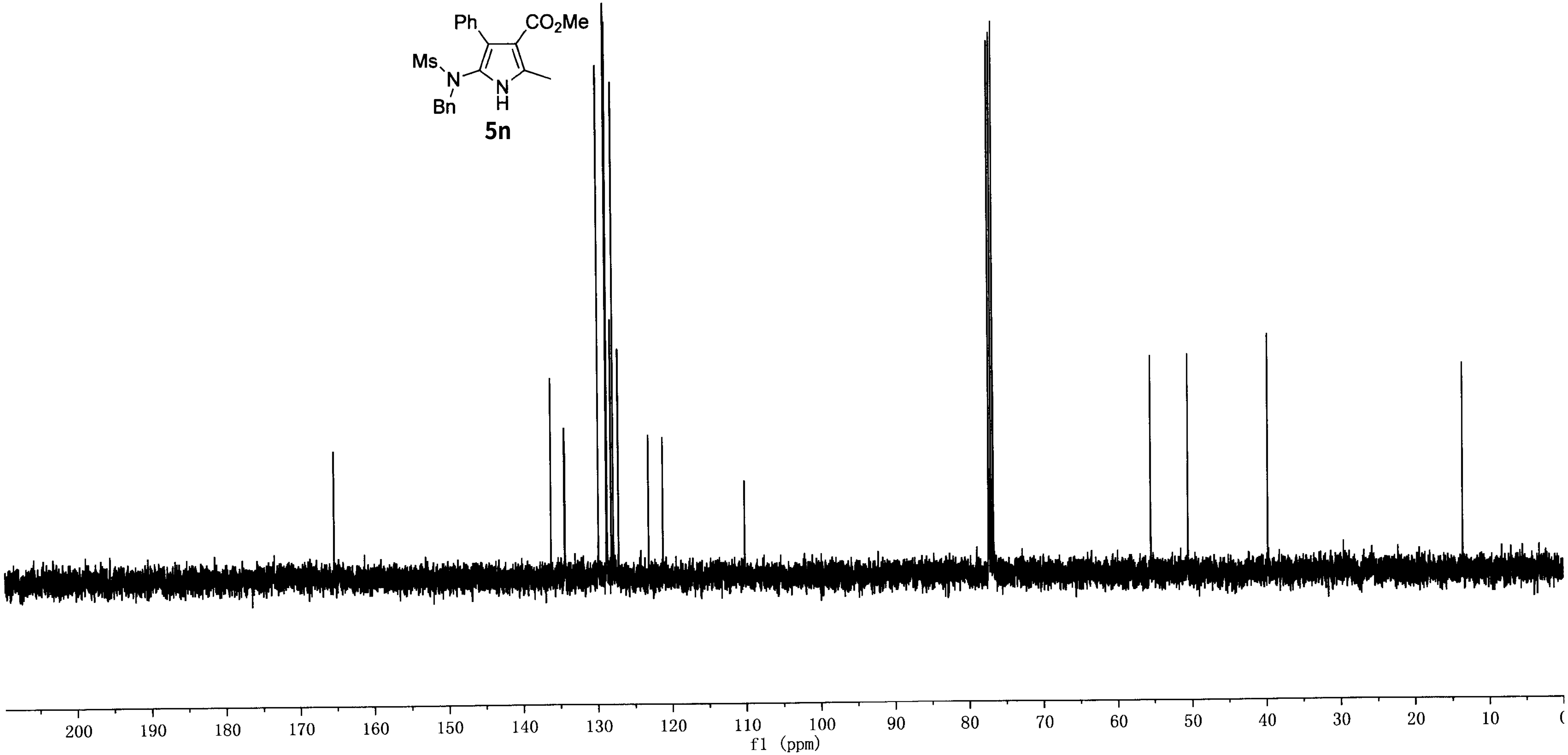
Ph
CO2Me
Ms
N
Bn
N
H
5n
200 190 180 170 160 150 140 130 120 110 100 90 80 70 60 50 40 30 20 10
f1 (ppm)

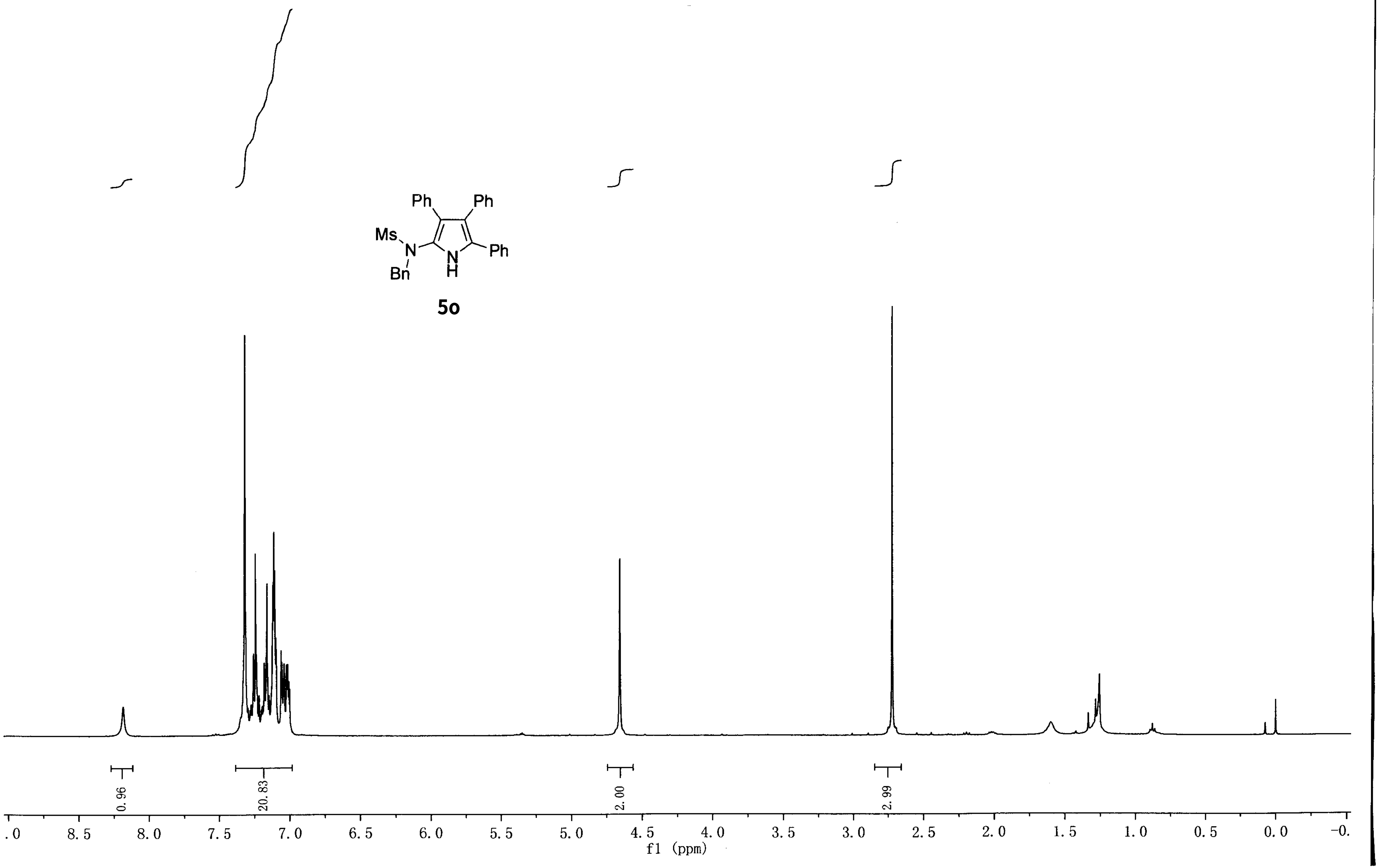

Ph
Ph
Ms
N
Bn
N
H
Ph
5o
0.96
20.83
2.00
2.99
.0
8.5
8.0
7.5
7.0
6.5
6.0
5.5
5.0
4.5
4.0
3.5
3.0
2.5
2.0
1.5
1.0
0.5
0.0
-0.
f1 (ppm)

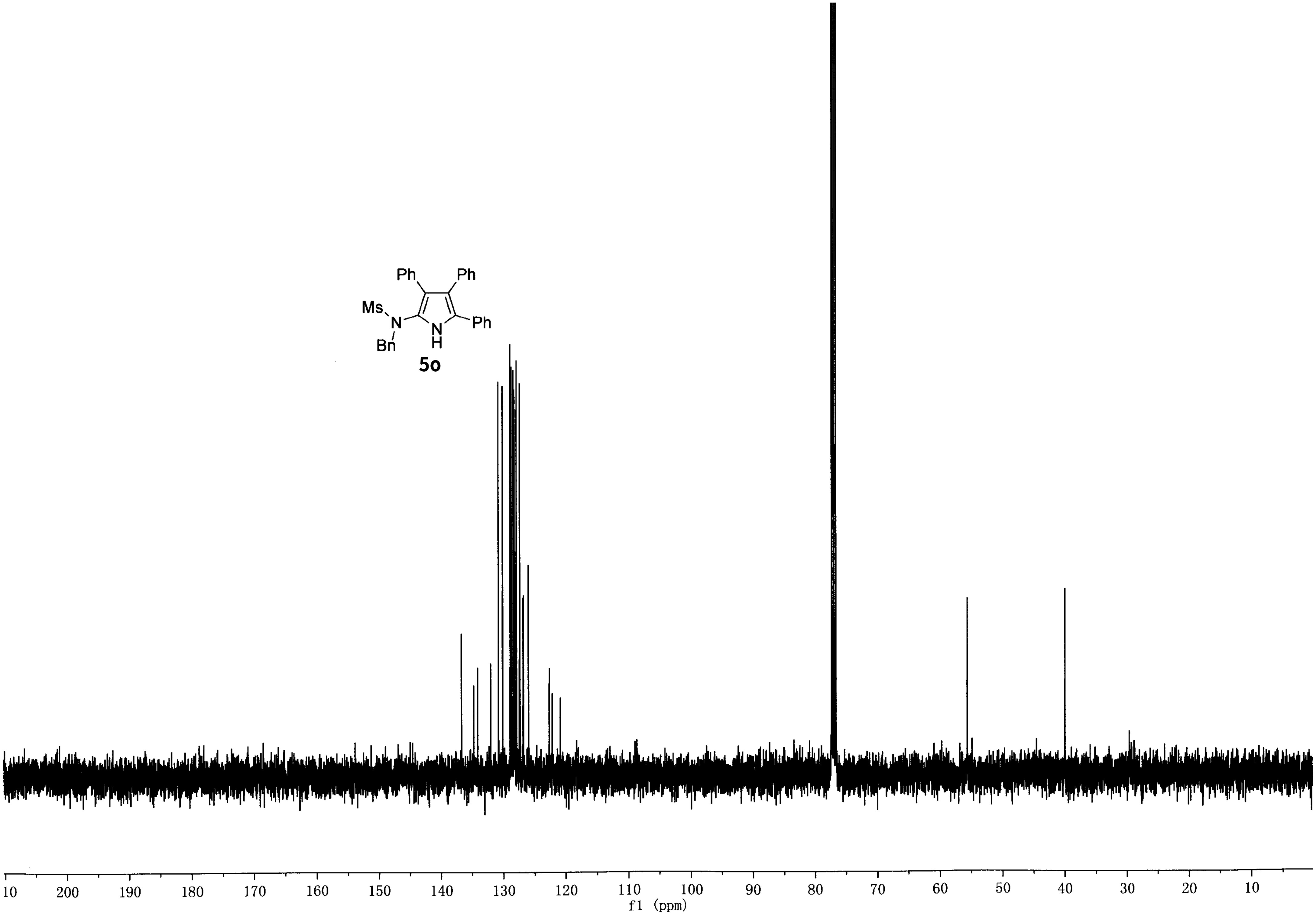
Ph
Ph
Ms
N
Ph
N
H
Bn
5o
10
200
190
180
170
160
150
140
130
120
110
100
90
80
70
60
50
40
30
20
10
f1 (ppm)

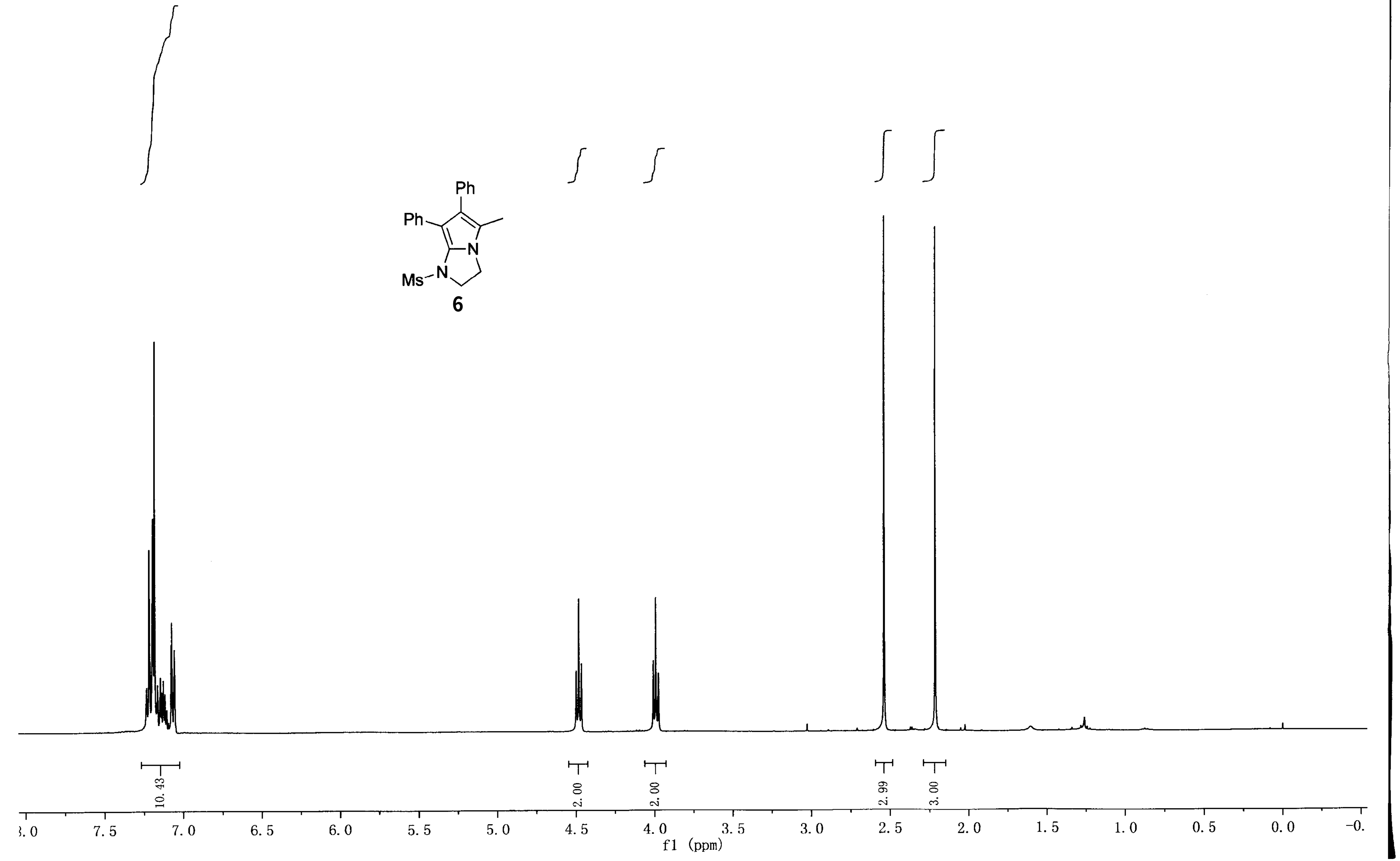

Ph
Ph
N
Ms
N
6
10.43
2.00
2.00
2.99
3.00
7.5
7.0
6.5
6.0
5.5
5.0
4.5
4.0
3.5
3.0
2.5
2.0
1.5
1.0
0.5
0.0
f1 (ppm)

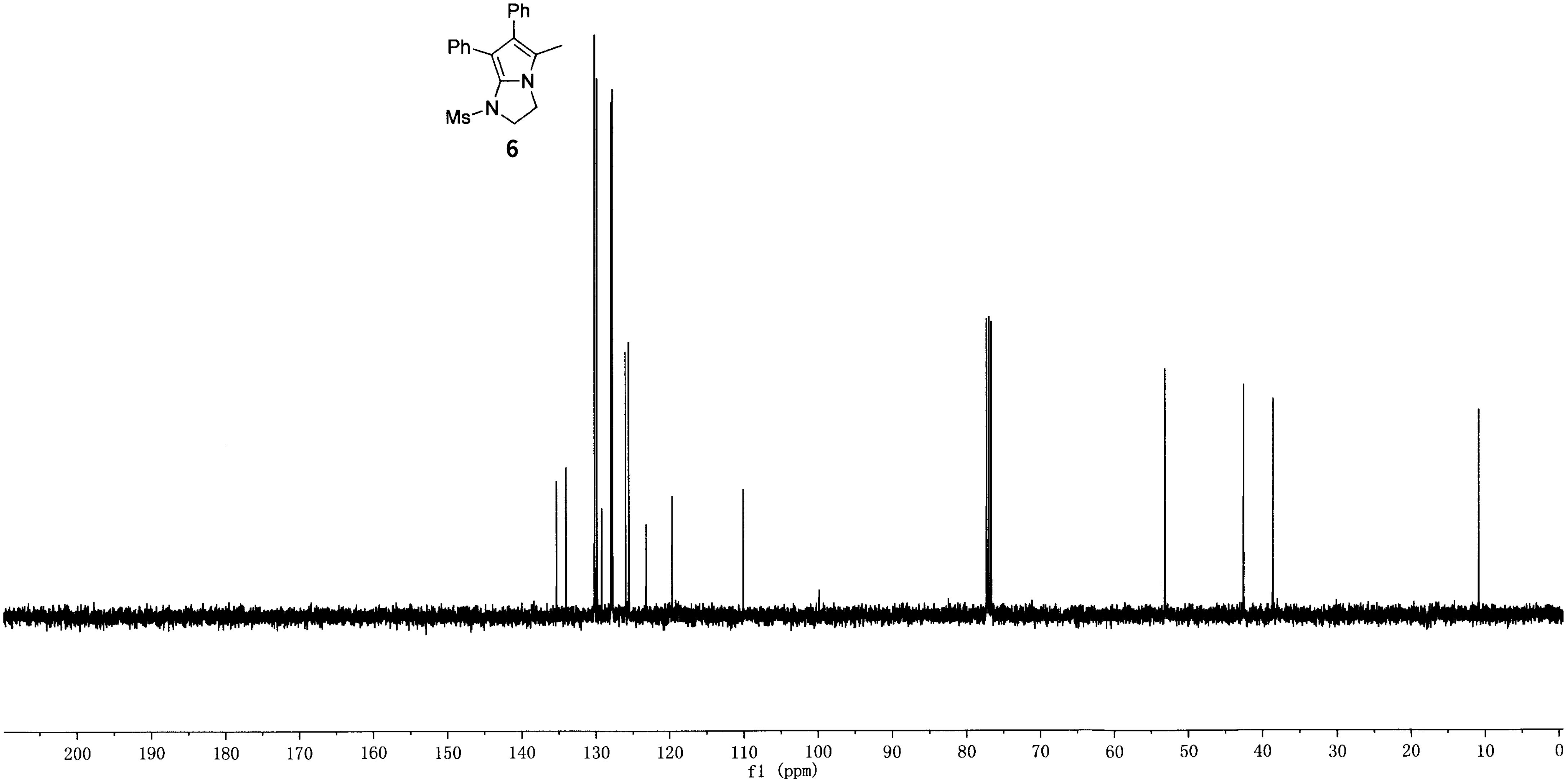
Ph
Ph
N
Ms
N
6
200 190 180 170 160 150 140 130 120 110 100 90 80 70 60 50 40 30 20 10 0
f1 (ppm)

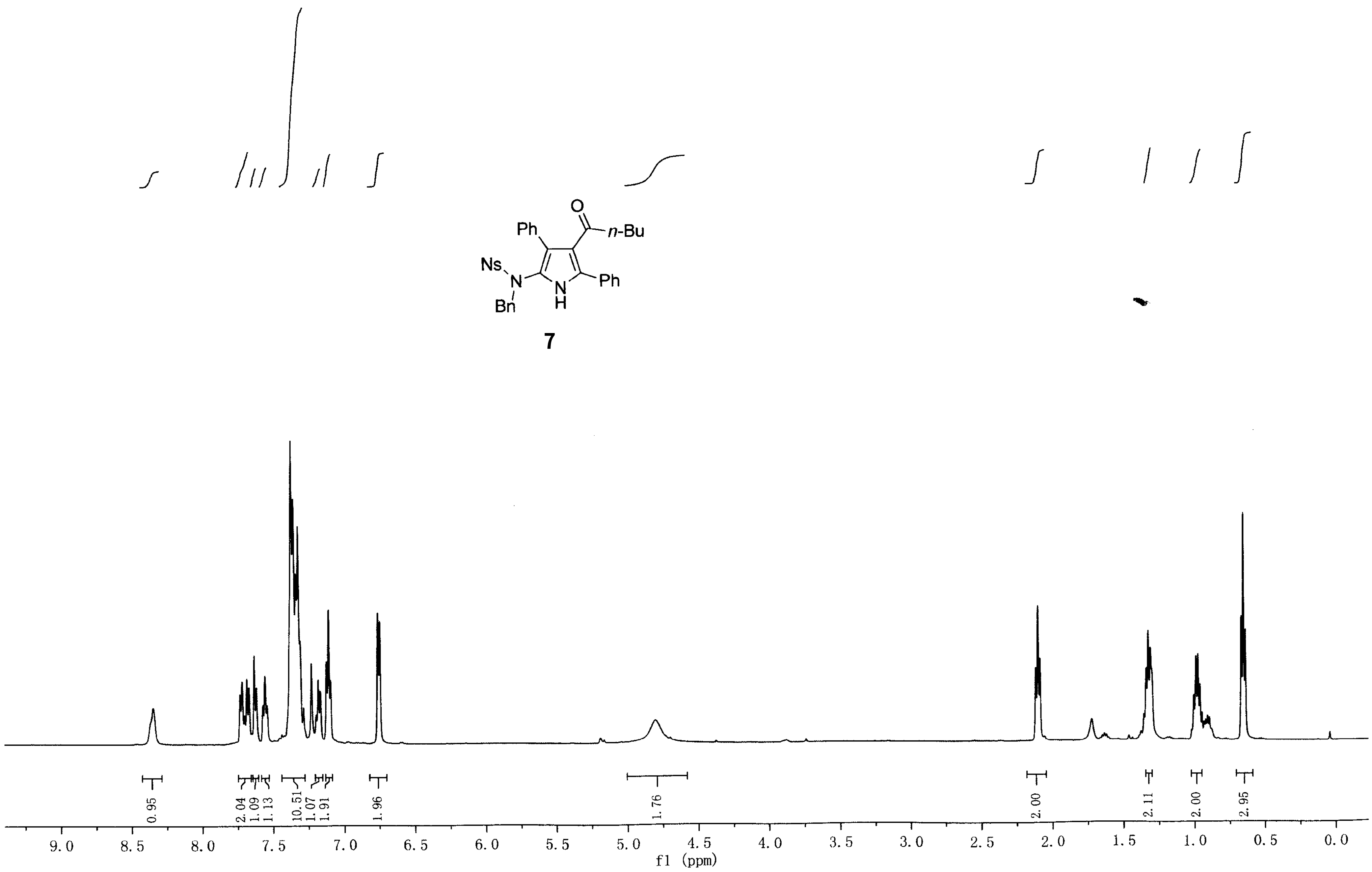

Ph
O
n-Bu
Ns
N
Ph
Bn
N
H
7
0.95
2.04
1.09
1.13
10.51
1.07
1.91
1.96
1.76
2.00
2.11
2.00
2.95
9.0
8.5
8.0
7.5
7.0
6.5
6.0
5.5
5.0
4.5
4.0
3.5
3.0
2.5
2.0
1.5
1.0
0.5
0.0
f1 (ppm)

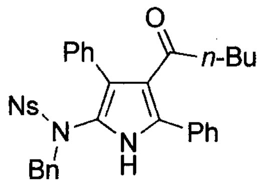
Ph
O
n-Bu
Ns
N
Bn
N
H
Ph

7

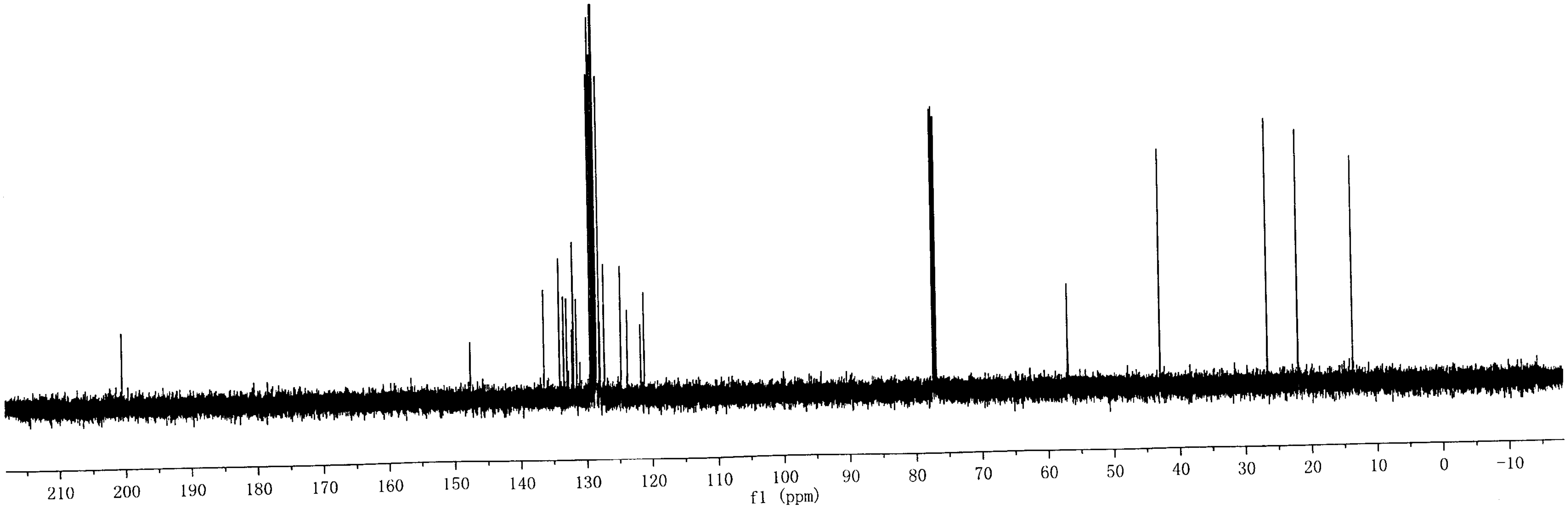
210
200
190
180
170
160
150
140
130
120
110
100
90
80
70
60
50
40
30
20
10
0
-10
f1 (ppm)

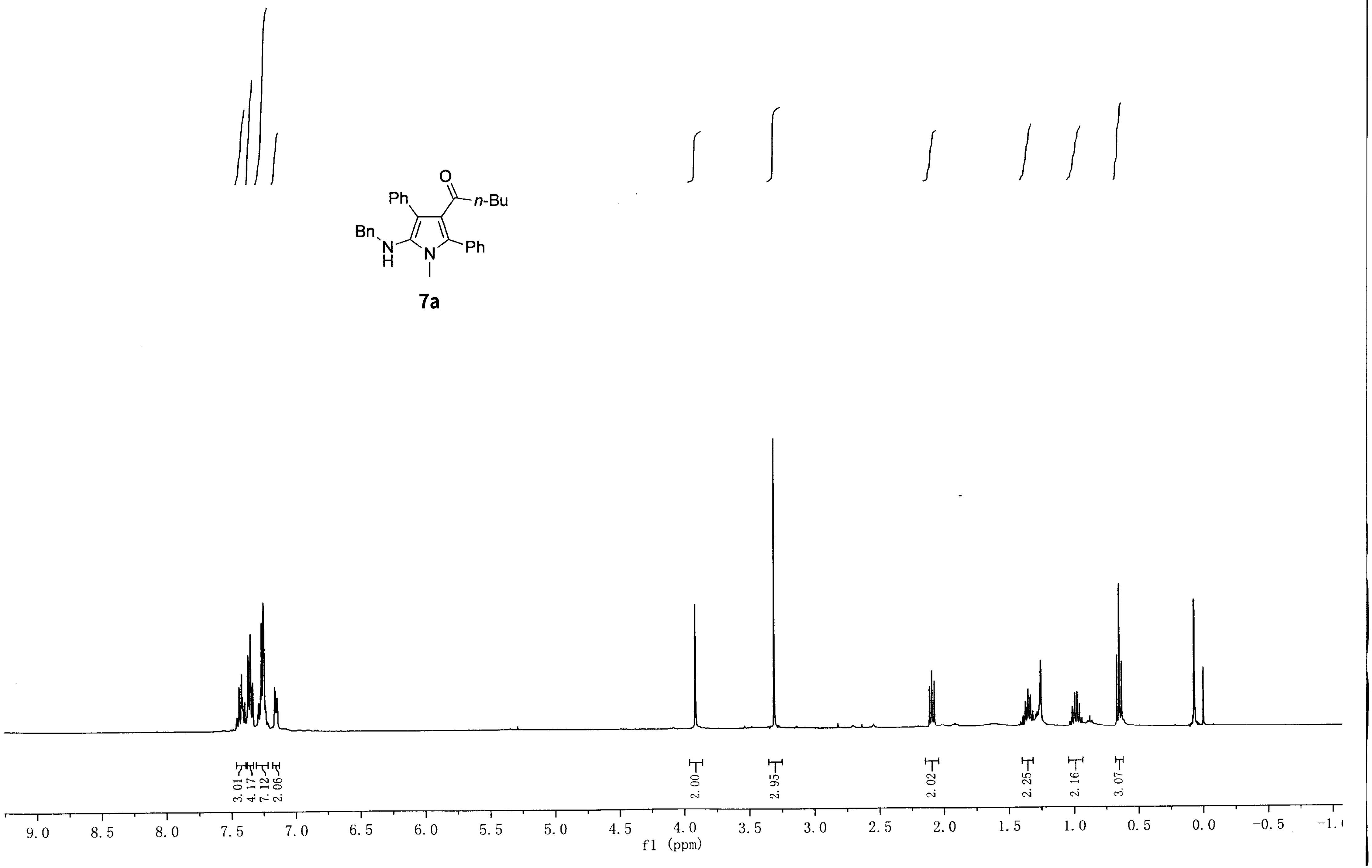

Ph
O
n-Bu
Bn
N
H
N
Ph
7a
3.01
4.17
7.12
2.06
2.00
2.95
2.02
2.25
2.16
3.07
9.0 8.5 8.0 7.5 7.0 6.5 6.0 5.5 5.0 4.5 4.0 3.5 3.0 2.5 2.0 1.5 1.0 0.5 0.0 −0.5 −1.
f1 (ppm)

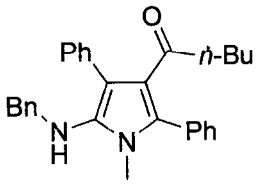

O
Ph
n-Bu
Bn
N
H
N
Ph

7a

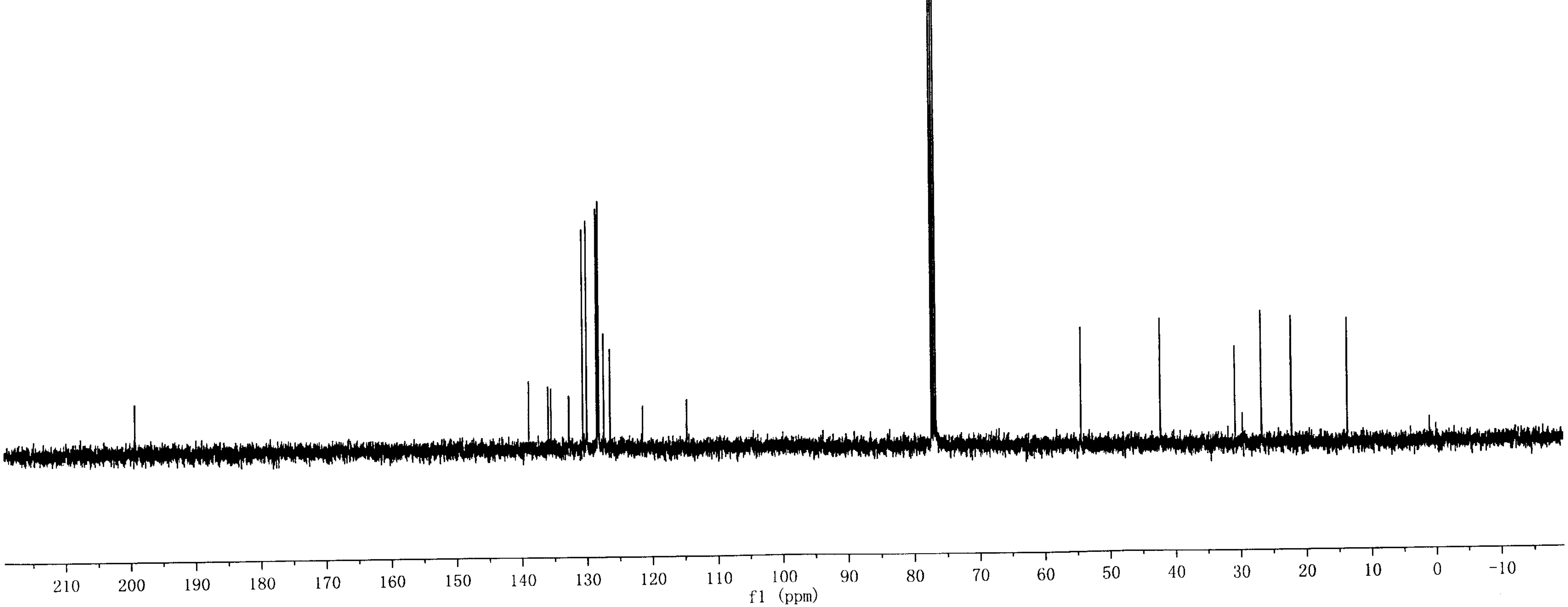

210
200
190
180
170
160
150
140
130
120
110
100
90
80
70
60
50
40
30
20
10
0
-10
f1 (ppm)

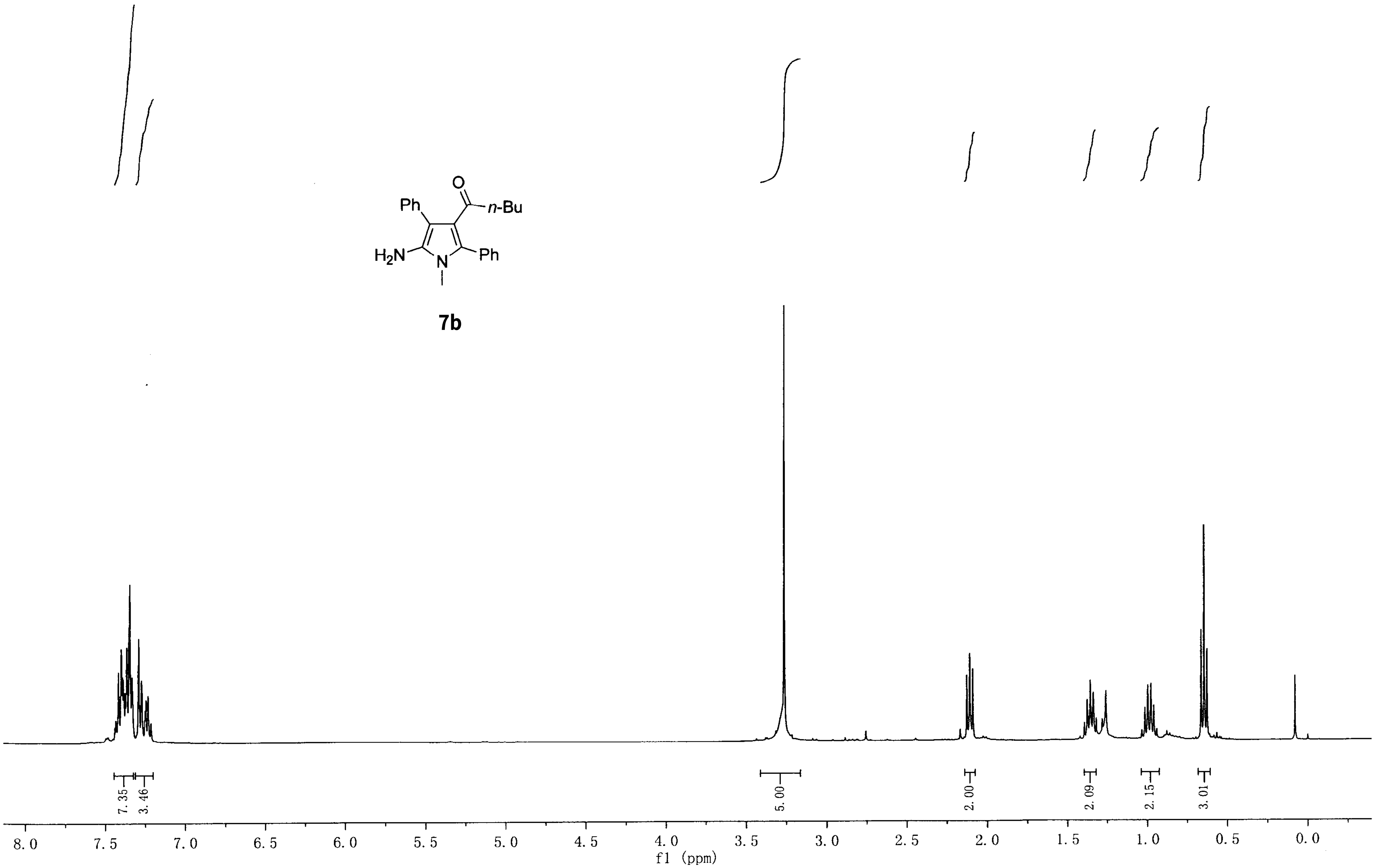

Ph
O
n-Bu
H2N
N
Ph
7b
7.35
3.46
5.00
2.00
2.09
2.15
3.01
8.0
7.5
7.0
6.5
6.0
5.5
5.0
4.5
4.0
3.5
3.0
2.5
2.0
1.5
1.0
0.5
0.0
f1 (ppm)

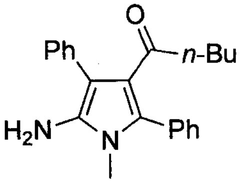

7b

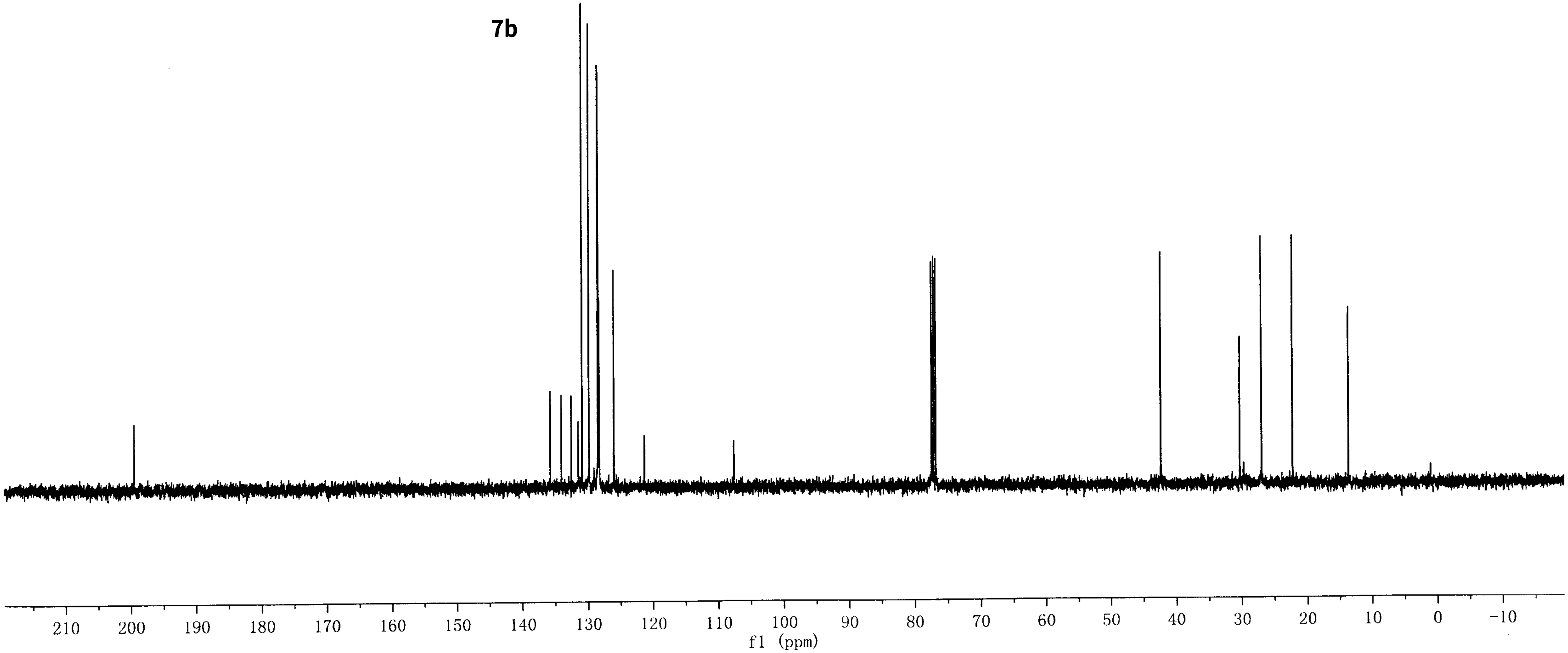
210
200
190
180
170
160
150
140
130
120
110
100
90
80
70
60
50
40
30
20
10
0
−10
f1 (ppm)

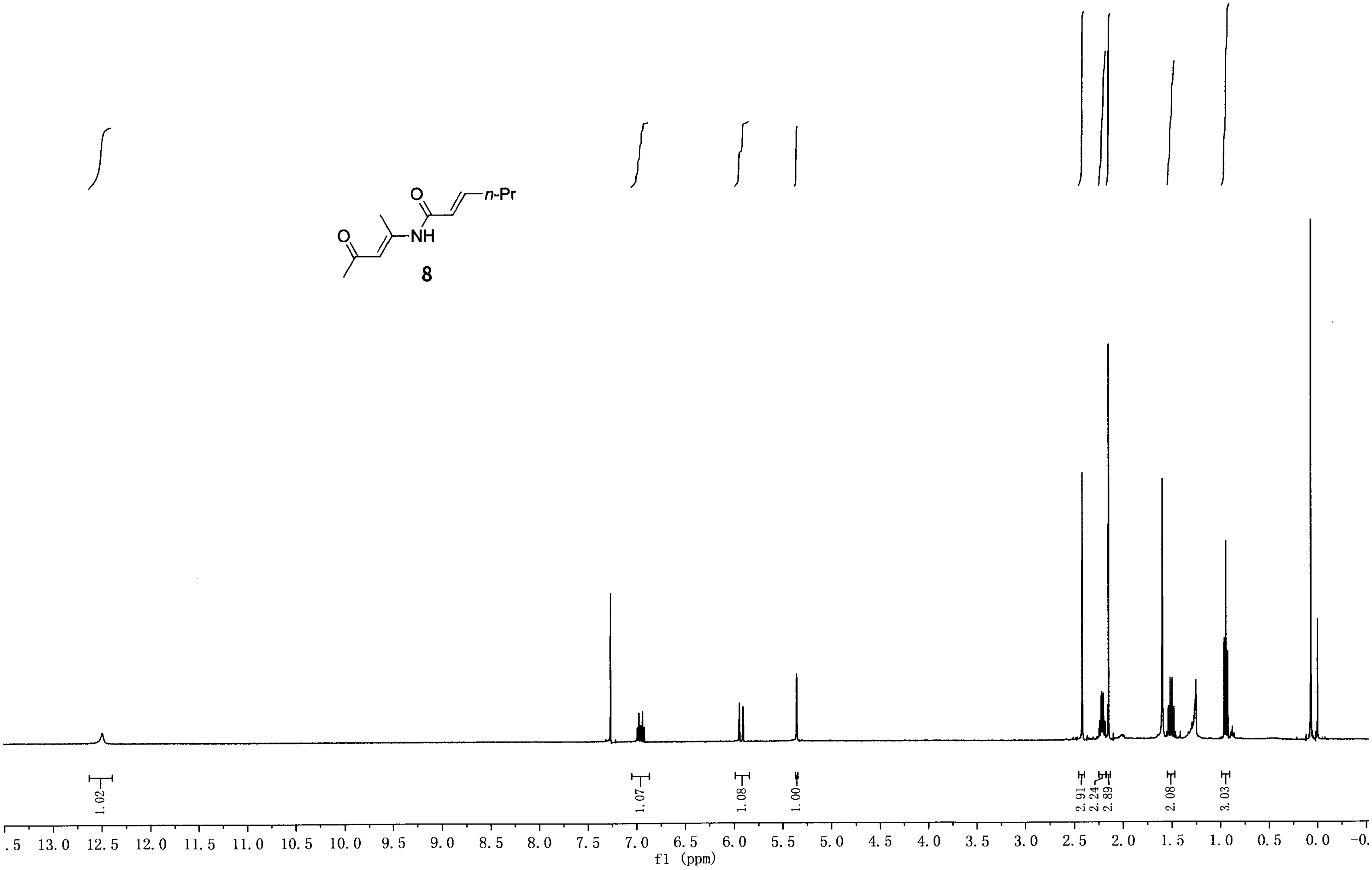
O
O
NH
n-Pr
8
1.02
1.07
1.08
1.00
2.91
2.24
2.89
2.08
3.03
.5 13.0 12.5 12.0 11.5 11.0 10.5 10.0 9.5 9.0 8.5 8.0 7.5 7.0 6.5 6.0 5.5 5.0 4.5 4.0 3.5 3.0 2.5 2.0 1.5 1.0 0.5 0.0 -0.
f1 (ppm)

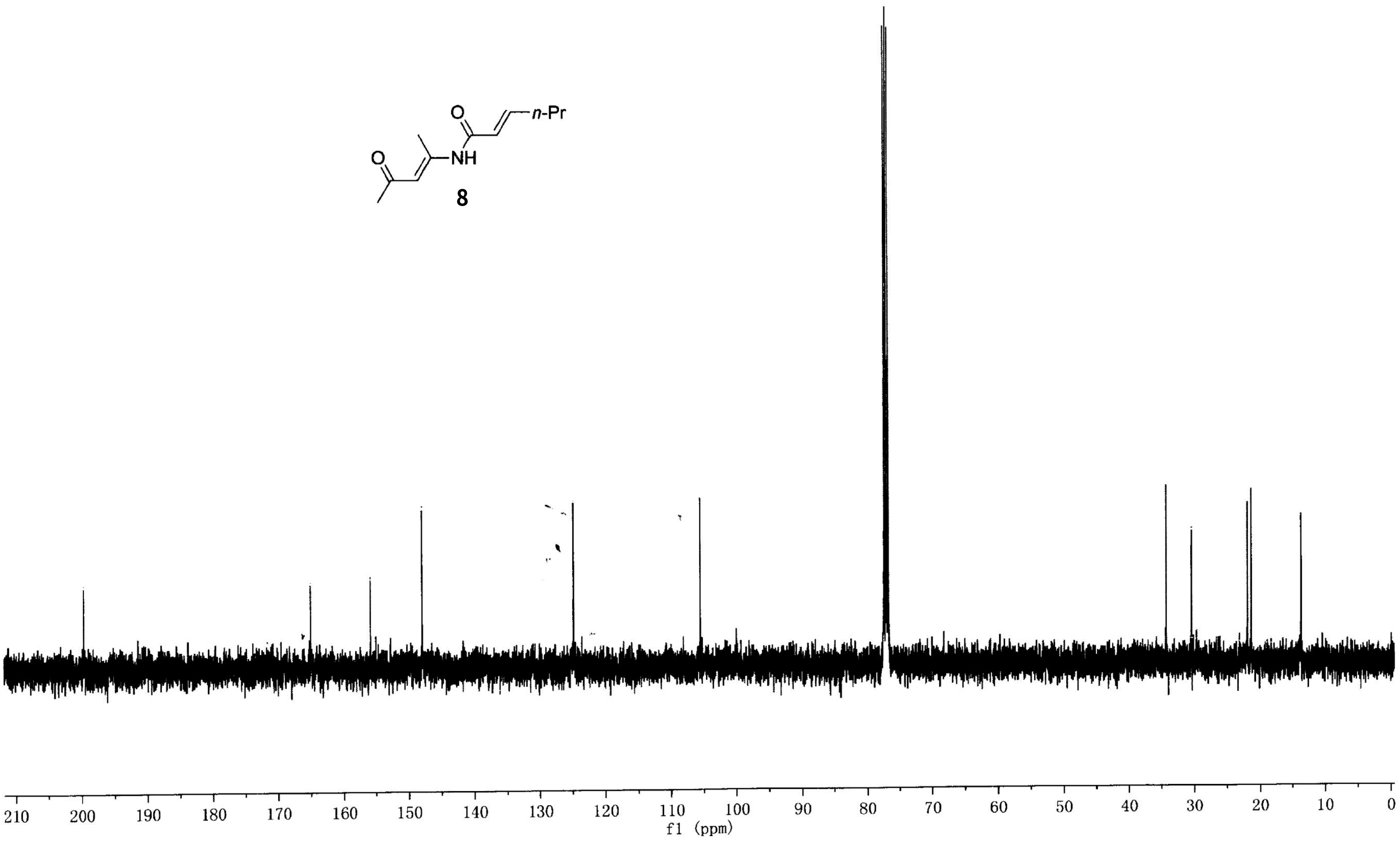

n-Pr
NH
O
O
8
210
200
190
180
170
160
150
140
130
120
110
100
90
80
70
60
50
40
30
20
10
0
f1 (ppm)
